# The CBI-M framework for characterization of traumatic brain injury: Evaluation of performance using data from the CENTER-TBI and TRACK-TBI prospective cohort studies

**DOI:** 10.1371/journal.pmed.1004945

**Published:** 2026-10-20

**Authors:** Dana Pisică, Ana Mikolić, John K. Yue, Michael A. McCrea, Kristen Dams-O’Connor, Véronique De Keyser, Sonia Jain, Amy J. Markowitz, David K. Menon, Virginia Newcombe, Ewout W. Steyerberg, Xiaoying Sun, Hester F. Lingsma, Geoffrey T. Manley, Andrew I. R. Maas, on behalf of the CENTER-TBI and TRACK-TBI Investigators

**Affiliations:** 1 Department of Public Health, Erasmus MC - University Medical Center Rotterdam, Rotterdam, The Netherlands; 2 Department of Neurosurgery, Erasmus MC - University Medical Center Rotterdam, Rotterdam, The Netherlands; 3 Department of Neurological Surgery, University of California, San Francisco, California, United States of America; 4 Brain and Spinal Injury Center, Zuckerberg San Francisco General Hospital, San Francisco, California, United States of America; 5 Department of Neurosurgery, Medical College of Wisconsin, Milwaukee, Wisconsin, United States of America; 6 Department of Rehabilitation and Human Performance, Icahn School of Medicine at Mount Sinai, New York, New York, United States of America; 7 Departments of Neuroscience and Neurology, Icahn School of Medicine at Mount Sinai, New York, New York, United States of America; 8 Department of Neurosurgery, Antwerp University Hospital, Edegem, Belgium; 9 Biostatistics Research Center, Herbert Wertheim School of Public Health and Longevity Science, University of California, San Diego, California, United States of America; 10 Department of Medicine, University of Cambridge, Cambridge, United Kingdom; 11 Julius Center for Health Sciences and Primary Care, University Medical Center Utrecht, Utrecht, The Netherlands; 12 Department of Translational Neuroscience, Faculty of Medicine and Health Science, University of Antwerp, Antwerp, Belgium; Barts and the London School of Medicine & Dentistry Queen Mary University of London, UNITED KINGDOM OF GREAT BRITAIN AND NORTHERN IRELAND

## Abstract

**Background:**

The CBI-M Framework, incorporating Clinical, Biomarker, Imaging, and Modifier pillars, was recently proposed to provide a comprehensive characterization of patients with acute traumatic brain injury (TBI). Such detailed characterization reflects the clinical and biological heterogeneity of TBI, and is considered essential to inform appropriate clinical management. Still unclear is the extent to which CBI-M captures clinically relevant heterogeneity in relation to diagnosis, clinical care pathways, and longer-term outcome. Prior to broad implementation into clinical practice and research, its diagnostic and prognostic performance needs to be ascertained. In this study, we aim to explore how CBI-M and its constituent pillars relate to diagnosis, clinical care pathways, and outcome in two large contemporary TBI cohorts.

**Methods and findings:**

We analyzed data of patients with acute TBI enrolled in the European-based CENTER-TBI (*N* = 4,509) and US-based TRACK-TBI (*N* = 2,552) prospective observational studies. We assessed associations of the CBI-M Framework, its pillars, and pillar components with key clinical endpoints: diagnosis (traumatic intracranial injury on computed tomography); care pathways (hospital admission, intensive care admission, intracranial pressure monitoring, and major cranial surgery); and outcomes at 6 months post-injury (mortality, Glasgow Outcome Scale-Extended (GOSE), and health-related quality of life (HRQoL)). Discriminatory performance was calculated using the AUC and explained variability expressed with Nagelkerke’s pseudo-R^2^. Prognostic performance was compared to the established IMPACT-Lab and CRASH-CT prediction models in their respective target populations. For diagnostic and care path endpoints full CBI-M models achieved excellent discrimination (AUC CENTER-TBI: from 0.83 to 0.95; TRACK-TBI: from 0.88 to 0.98), and high proportions of explained variability (pseudo-R^2^ CENTER-TBI: from 0.41 to 0.72; TRACK-TBI: from 0.55 to 0.80). Contributions of individual pillars varied by endpoint, with the Biomarker pillar contributing most for CT-defined abnormalities and the Imaging pillar to major cranial surgery. For mortality and unfavorable outcome (GOSE ≤ 4) similarly strong performance was observed: CENTER-TBI: AUC 0.89 (95% Confidence intervals (CI) [0.88,0.90]) and 0.92 (95% CI [0.91,0.94]) respectively; pseudo-R^2^ 0.53 (95% CI [0.50,0.57]) and 0.55 (95% CI [0.51,0.59]) respectively; TRACK-TBI: AUC 0.91 (95% CI [0.89,0.93]) and 0.95 (95% CI [0.93,0.97]) respectively; pseudo-R^2^ 0.57 (95% CI [0.52,0.62] and 0.57 (95% CI [0.50, 0.64] respectively), whereas performance for HRQoL was modest. Compared to the IMPACT-Lab and CRASH-CT models in their respective target populations, pseudo-R^2^ values suggest that the full CBI-M model was more strongly associated with 6-month mortality and unfavorable outcomes. Methodological limitations include the restriction to analysis of data from high-income settings, limiting generalizability, the absence of three elements of the modifier pillar in the datasets accessed, and risk of possible reverse causality in some analyses.

**Conclusions:**

CBI-M provides a robust multimodal characterization of acute TBI that aligns closely with clinical decision-making and outcomes. Further prospective validation and optimization are justified and needed to inform implementation of the CBI-M Framework in clinical practice and research.

## Author summary

**Why was this study done?:**

- Traumatic brain injury (TBI) is a complex and heterogeneous condition.
- The complexity of the disease is poorly captured by the traditional approach to classification, which is based only on clinical severity and groups patients into three categories of mild, moderate, or severe.
- A new framework (CBI-M) has been proposed to better describe TBI using clinical features, blood biomarkers, imaging findings, and modifiers.
- It is not yet known whether this framework meaningfully reflects how patients are diagnosed, treated, and recover in real-world clinical settings.

**What did the researchers do and find?:**

- We analyzed data from over 7,000 patients with TBI enrolled in two large international studies (CENTER-TBI and TRACK-TBI).
- We found that the CBI-M framework closely aligned with clinical decision-making, including diagnosis, hospital care pathways, and outcomes at 6 months.
- Different components of the framework contributed in different ways: clinical, biomarker, and imaging features were most relevant for early care decisions, while patient-related factors were more important for longer-term recovery and quality of life.

**What do these findings mean?:**

- These results provide empirical support for the potential clinical relevance of the CBI-M framework, and support further development towards clinical and research use.
- Whilst its applicability across all ranges of clinical severity is a strength, some elements of a pillar may be more relevant in less severely injured patients and others in more severely injured patients.
- Steps towards clinical implementation should include prospective validation, combined with refining and weighting of pillar elements.
- Limitations of the study include its retrospective nature, the restriction to data from high-income countries and lack of data on three basic components of the modifier pillar.

## Abstract 3

Dana Pisică, Ana Mikolić, John K. Yue and colleagues find that the CBI-M Framework is associated with clinically relevant endpoints for patients with traumatic brain injury, including diagnosis, hospital care pathways, and outcomes at six months. Further optimisation and validation is needed before implementation in clinical practice and research.

## Introduction

Traumatic brain injury (TBI) has the highest incidence of all common neurological disorders, and poses a substantial public health burden. Approximately 50–60 million people sustain a TBI each year, with an estimated annual cost to the global economy of around US$400 billion [1]. This heterogeneous disease, with multiple phenotypes, is now recognized not only as an acute event, but also as a chronic condition with long-term consequences [2]. Detailed characterization is essential to inform appropriate care, enable individualized management and to improve on diagnosis and outcome prediction in TBI. The traditional approach to classification of clinical severity of acute TBI is based on unidimensional assessment by the Glasgow Coma Scale (GCS) score, categorizing patients as “mild” (GCS 13–15), “moderate” (GCS 9–12), or “severe” (GCS 3–8). While widely used, this approach captures only a limited aspect of brain injury severity and fails to reflect the underlying biological heterogeneity. People with lived experience of TBI object to being labeled as mild, moderate, or severe [3–5]. A novel multimodal framework (“CBI-M”) was recently proposed to provide a more comprehensive characterization of patients with TBI [3]. This new Framework builds upon the GCS with four pillars containing multiple and more nuanced features: Clinical (C), Biomarker (B), Imaging (I), and Modifiers (M). The Framework was developed through an international initiative launched in 2023 by the U.S. National Institutes of Health-National Institute of Neurological Disorders and Stroke (NIH-NINDS) to develop a more precise, multimodal framework for TBI characterization and nomenclature, applicable across the TBI severity continuum. The initiative was based on consensus and expert opinion, informed by extensive literature searches, involving 94 TBI experts from 14 countries, and a panel of people with lived experience of TBI. Although conceptually appealing, the empirical performance of the CBI-M Framework, both as an integrated construct and through its individual pillars, has not yet been evaluated in large, contemporary clinical cohorts. In particular, it remains unclear to what extent CBI-M captures clinically relevant heterogeneity in relation to diagnosis, clinical care pathways, and longer-term outcomes.

The aim of the present study was to provide empirical evidence on how the CBI-M Framework functions as a tool for refined characterization of patients with TBI. We quantify relationships of the full CBI-M Framework and its individual pillars with clinically important endpoints related to TBI diagnosis, care pathways, and outcome in two large contemporary cohorts. We hypothesized that the CBI-M full model and its individual pillars would be associated with diagnostic, care pathway and 6-month outcome endpoints in both cohorts, as quantified by measures of model performance.

## Methods

### Study design and population

We analyzed data from two contemporary prospective longitudinal observational multi-center studies. The Collaborative European NeuroTrauma Effectiveness Research in Traumatic Brain Injury (CENTER-TBI; ClinicalTrials.gov NCT02210221) Core Study enrolled patients from 65 trauma centers across 18 countries in Europe and Israel between December 2014 and December 2017 [6,7]. The Transforming Research and Clinical Knowledge in Traumatic Brain Injury (TRACK-TBI; ClinicalTrials.gov NCT02119182) Study enrolled patients from 18 Level 1 trauma centers in the United States (US) between February 2014 and July 2018 [8]. Both studies enrolled patients who presented to participating centers within 24 hours (h) of injury with a clinical diagnosis of TBI, an indication for computed tomography (CT) scanning according to local protocols and no severe pre-existing neurological disorders that could confound outcome assessment. For both studies, ethical approval was obtained by each participating center and written informed consent was provided by all enrolled patients or their legally authorized representatives.

Analyses were planned in the autumn of 2024 by the CENTER-TRACK Working Group at the initiative of NIH-NINDS. The statistical approach was refined during weekly CENTER-TRACK Working Group meetings. The analyses were updated in June 2025 to reflect the final version of the proposed new framework. No data-driven changes to analyses were made. At the reviewers’ request, Area under the Receiver Operating Characteristic curves (AUC), optimism-correction, and confidence intervals were added to the main tables and figures. To explore the potential reverse causality where biomarker levels may reflect the surgical procedure rather than predicting the need for surgery, as suggested by the reviewers, we performed sensitivity analyses of the association between biomarker levels and intracranial surgery in patients whose biomarker levels were sampled before versus after surgery. We also explored changes in biomarker levels before and after surgery by paired Wilcoxon test in the subset of patients with available paired data. No AI tools were used in the generation of this manuscript. The authors retain full responsibility for the final manuscript.

### Data collection and management

#### Pillar components.

Details regarding collection, handling, and storage of clinical data in the CENTER-TBI Core Study [6,7] and in the TRACK-TBI study [9] have been previously reported. For the current study, we extracted the available basic components of the Clinical, Biomarker, Imaging, and Modifier pillars of the CBI-M Framework [3]. Basic pillar components and their availability in the CENTER- and TRACK-TBI datasets are presented in S1 Table. For the Biomarker pillar, we analyzed the first blood sample obtained within 24 hours of injury. Samples were collected as part of research and assay results were not available to treating clinicians for decision-making. For the Imaging pillar, we analyzed findings from the first clinical head CT, reported centrally according to the NIH-NINDS TBI Imaging Common Data Elements [10].

#### Endpoints.

We analyzed clinical endpoints pertaining to: 1) diagnosis: presence of acute traumatic intracranial injury on the first head CT, 2) care pathway: admission to hospital, admission to the intensive care unit (ICU), intracranial pressure (ICP) monitoring in patients admitted to the ICU, and major cranial surgery within 72 hour of injury (craniotomy or decompressive craniectomy for hematoma evacuation and/or cerebral edema), and 3) clinical outcomes at 6 months post-injury, including mortality, the Glasgow Outcome Scale-Extended (GOSE) [11,12], and health-related quality of life (HRQoL) in survivors.

The GOSE [11,12] assesses the level of functioning compared to pre-injury level on a scale from death (1) to upper good recovery (8) by in-person/telephone interviews or postal questionnaires. Missing 6-month GOSE scores were imputed based on trajectories of GOSE obtained at 2 weeks, 3 months, and 12 months post-TBI using a Markov multi-state model, adjusted for age [13]. Where patients presented for at least one GOSE assessment (at any of these time points), missing outcomes at other time points were assumed to be missing at random (MAR). The GOSE was scored to capture the symptoms, disabilities, and consequences of the participant’s overall injury burden, inclusive of TBI and systemic injuries [12]. For analysis, the GOSE was dichotomized as unfavorable outcome (GOSE < 5) and incomplete recovery (GOSE < 8). HRQoL was assessed using the Quality of Life after Brain Injury-Overall Scale (QoLIBRI- OS) [14]. A total score < 52 was considered indicative of impaired HRQoL [15].

### Data analyses

Distributions of pillar components and endpoints were reported as absolute and relative frequencies for categorical variables and medians and 25th and 75th percentiles (p25-p75) for continuous variables. For regression analyses, biomarker values were log-transformed and standardized to z-scores, and analyses included interactions between biomarker sampling time and biomarker values [16].

Pairwise correlations between pillar components and endpoints were assessed in available cases with Spearman’s rank correlation coefficient (rho, possible range −1 to –1). To visualize the strength of these relationships, coefficients were plotted in heatmaps.

The primary analyses were performed after imputation of missing pillar components. Multiple imputation was performed within each study based on all pillar components, endpoints and IMPACT [17] and CRASH [18] predictors with the MICE package in R (S2 Table) [19]. Missing endpoints were imputed as part of the algorithm but were not used in the analyses. Sensitivity analyses were performed on available cases (including imputed GOSE endpoints [13]).

The performance of each pillar was examined in multivariable single-pillar logistic regression models containing all available basic components within a pillar, allowing for nonlinearity (modeled with restricted cubic splines with 3 knots) of all continuous predictors, and in cumulative multivariable logistic regression models. Models were refined in terms of nonlinearity of continuous predictors and interaction terms between biomarkers and sampling time separately within each dataset. For cumulative models, we added the linear predictors of single-pillar models in consecutive order. We started from the Clinical pillar model (C model), and then sequentially added other pillars: first the Biomarker pillar (C + B model), followed by Imaging (C + B + I model), and finally, the full CBI-M model, including the Modifier pillar. Model performance was evaluated in terms of discrimination and overall model fit, quantified with the AUC and Nagelkerke’s pseudo-R^2^, respectively. Confidence intervals (CIs) and optimism in model performance were derived from 1,000 bootstrap samples drawn with replacement. An AUC of 0.5 indicates discrimination equal to chance, while an AUC of 1.0 indicates perfect discrimination. Nagelkerke pseudo-R^2^ is a likelihood-based measure of model fit for logistic regression that we used as a proxy for the proportion of outcome variability explained by the model. Values can range between 0 and 1, with higher values indicating better model fit. The measure is well suited to compare the performance of different models in the same dataset. Partial pseudo-R^2^ statistics, representing the improvement in model fit attributable to pillar or component, were calculated and visualized in sunburst plots, for each pillar within the CBI-M model and for each pillar component within single pillar models, aiming to provide insight into unique explanatory contributions. Subgroup analysis was performed for “mild TBI” (GCS 13–15) and “moderate to severe TBI” (GCS 3–12).

The performance of cumulative models for predicting mortality and unfavorable outcome at 6 months post-injury was compared to that of established prediction models (IMPACT-Lab model [17] and CRASH-CT [18] model). All models were re-fitted in IMPACT and CRASH target subpopulations within the CENTER-TBI and TRACK-TBI datasets for a fair comparison to CBI-M models.

This study was conducted and reported in accordance with the Strengthening the Reporting of Observational Studies in Epidemiology (STROBE) statement (S3 Table) [20]. Analyses were performed using R version 4.3.3 [21], and RStudio [22] version 2023.12.1.402. No AI tools were used in the generation of this manuscript. The authors retain full responsibility for the final manuscript.

### Ethics statement

CENTER-TBI was registered with ClinicalTrials.gov (NCT02210221). Informed written or oral consent by patients or legal representatives was obtained according to local regulation. The list of sites, ethics committees, approval numbers, and approval dates can be found at: https://www.center-tbi.eu/project/ethical-approval [accessed 07/13/2026]. Clinical data were accessed via the Neurobot platform (RRID/SCR_017004, core data, version 3.0). TRACK-TBI was registered with ClinicalTrials.gov (NCT02119182) and was approved by the Institutional Review Board of the University of California, San Francsico (Protocol Approval No. 12-09465). All participants or their legally authorized representative provided written or oral informed consent. The study protocols strictly adhered to the ethical principles outlined in the Declaration of Helsinki and the Belmont Report.

## Results

### Study populations and patient characteristics

CENTER-TBI enrolled 4,509 and TRACK-TBI 2,552 participants with TBI, respectively. Compared with TRACK-TBI participants, CENTER-TBI participants were older, had more severe clinical characteristics, with a higher frequency of intracranial and extracranial injuries (Table 1). Based on initial GCS, 2,957 (68%), 389 (9%), and 986 (23%) CENTER-TBI participants and 2000 (80%), 123 (5%), and 362 (15%) TRACK-TBI participants were classified as having mild, moderate, or severe TBI, respectively. Blood-based biomarkers were sampled at a median of 13.3 h [p25-p75: 6.3,19.1] and 15.8h [p25, p75: 9.6,20.7] post-injury in CENTER-TBI and TRACK-TBI, respectively. The most frequent intracranial lesion was traumatic subarachnoid hemorrhage (Table 1). By 6 months, unfavorable outcome (GOSE < 5) was reported in 971/3811 (25.5%) CENTER-TBI and 325/2122 (15.3%) TRACK-TBI participants, including 473/3811 (12.4%) CENTER-TBI and 126/2122 (5.9%) TRACK-TBI participants who died (S4 Table).

**Table 1:** Distribution of pillar components in CENTER-TBI and TRACK-TBI.

	CENTER-TBI		TRACK-TBI	
	Overall	% Missing	Overall	% Missing
***N***	**4,509**		**2,552**	
**Clinical pillar**				
Baseline GCS Sum score (median [p25, p75])	15.0 [10.0, 15.0]	3.9	15.0 [14.0, 15.0]	2.6
Baseline GCS Eye score (median [p25, p75])	4.0 [2.0, 4.0]	2.1	4.0 [4.0, 4.0]	3.2
Baseline GCS Motor score (median [p25, p75])	6.0 [5.0, 6.0]	2.4	6.0 [6.0, 6.0]	4.4
Baseline GCS Verbal score (median [p25, p75])	5.0 [2.0, 5.0]	3.6	5.0 [4.0, 5.0]	10.2
Baseline pupils (%)		5.8		12.8
Both reactive	3,802 (89.5)		2,078 (93.4)	
One reactive	164 (3.9)		42 (1.9)	
Both unreactive	281 (6.6)		105 (4.7)	
**Biomarker pillar**				
logGFAP (median [p25, p75])^a^	8.2 [6.4, 9.7]	28.5	6.1 [4.6, 7.5]	4.8
logUCH-L1 (median [p25, p75])^a^	4.6 [3.6, 5.8]	28.7	5.4 [4.7, 6.2]	4.8
logS100B (median [p25, p75])^b^	−1.9 [−2.5, −1.1]	28.1	−2.0 [−2.6, −1.3]	13.7
Time to sampling (median [p25, p75])	13.3 [6.3, 19.1]	27.9	15.8 [9.6, 20.7]	10.3
**Imaging pillar**				
No abnormalities detected^c^ (%)	1,520 (37.2)	9.4	1,242 (50.8)	4.2
Skull fracture (%)	1,529 (37.4)	9.4	41 (1.7)	7.8
Epidural hematoma (%)	463 (11.3)	9.4	189 (7.8)	4.5
Subdural hematoma (%)	1,272 (31.1)	9.4	675 (27.7)	4.5
Traumatic subarachnoid hemorrhage (%)	1852 (45.3)	9.4	850 (34.9)	4.5
Contusion or intracerebral hemorrhage (%)	1,338 (32.7)	9.4	501 (20.6)	4.5
TAMVI^d^ (%)	336 (8.2)	9.4	217 (8.9)	4.5
Intraventricular hemorrhage (%)	491 (12.0)	9.4	120 (4.9)	4.5
Mass effect (%)	778 (19.0)	9.4	239 (9.8)	4.5
Total lesion volume ≥ 25 cm^3^ (%)	770 (18.8)	9.4	228 (9.4)	4.5
**Modifier pillar**				
Low velocity mechanism of injury (%)	2,145 (50.1)	5.1	991 (38.9)	0.3
Major extracranial injury (%)	1,642 (36.4)	0	530 (20.8)	0
Nonaccidental injury cause^e^ (%)	293 (6.7)	2.7	171 (6.7)	0.6
Hypoxia^f^ (%)	299 (7.0)	5.6	154 (6.0)	0
Hypotension^g^ (%)	297 (6.9)	4.7	153 (6.0)	0
Early posttraumatic seizures^h^	197 (4.5)	2.9	86 (3.4)	0
Age (median [p25, p75])	50 [30, 66]	0	38 [26, 55]	0
Male sex (%)	3,023 (67.0)	0	1,767 (69.2)	0
Medical history (%)	2,406 (55.0)	2.9	1,458 (57.2)	0
Mental health condition (%)	601 (13.8)	3.5	563 (22.1)	0
Developmental history (%)	57 (1.3)	3.8	190 (7.4)	0
TBI history (%)	402 (9.7)	7.8	467 (20.6)	11.0

Abbreviations: GCS, Glasgow Coma Scale; GFAP, glial fibrillary acidic protein; S100B, S100 calcium-binding protein B; p, percentiles; TAMVI, traumatic axonal microvascular injury; TBI, traumatic brain injury; UCH-L1, ubiquitin C-terminal hydrolase L1.

^a^CENTER-TBI: measured with an ultrasensitive immunoassay using digital array technology (Single Molecule Arrays, SiMoA) - based Human Neurology 4 - Plex B assay (N4PB) run on the SR-X benchtop assay platform (Quanterix Corporation, Lexington, Massachusetts, USA) at the University of Florida (Gainesville, Florida, USA), TRACK-TBI: measured with a sandwich enzyme-linked immunoassay with electrochemical detection of the resulting enzyme signal optimized for plasma, using either a hand-held point-of-care platform (i-STAT; Abbott Laboratories, Abbott Park, Illinois, USA) or a core clinical laboratory platform (ARCHITECT; Abbott Laboratories, Abbott Park, Illinois, USA).

^b^CENTER-TBI: measured with a clinical-use automated system, using an electrochemiluminescence immunoassay kit (ECLIA) (Elecsys S100) run on the e 602 module of Cobas 8,000 modular analyzer (Roche Diagnostics, Germany) at the University of Pecs (Pecs, Hungary), TRACK-TBI: measured similarly to CENTER-TBI using an electrochemiluminescence immunoassay (Elecsys S100; Roche Diagnostics, Germany) run on the Cobas analyzer (Roche Diagnostics, Germany).

^c^Abnormality includes isolated skull fracture.

^d^Diffuse axonal injury/traumatic axonal injury and punctate hemorrhages.

^e^Proxy for Stressful circumstances.

^f^Defined as a documented PaO2 < 8 kPa (60 mmHg) and/or SaO2 < 90% or if the patient did not have documented hypoxia by PaO2 or SaO2, but there was a clinical suspicion, as evidenced by for example cyanosis, apnea, or respiratory distress.

^g^Defined as a documented systolic BP < 90 mm Hg (adults) or if the patient did not have a documented blood pressure, but was reported to be in shock or have an absent brachial pulse (not related to injury of the extremity).

^f^Defined as seizures occurring before or on presentation to hospital.

### Correlations between pillar components within and across pillars and endpoints

Correlations between components within and across the Clinical, Biomarker and Imaging pillars were generally strong, whereas correlations within the Modifier pillar ranged from negligible to moderate (Fig 1, coefficients in S1 Fig). Correlation patterns were consistent across both studies. Given the strong correlations between the GCS sum score and its components (range 0.80 to 0.96), the GCS sum score was used in the subsequent multivariable analyses.

**Fig 1:**
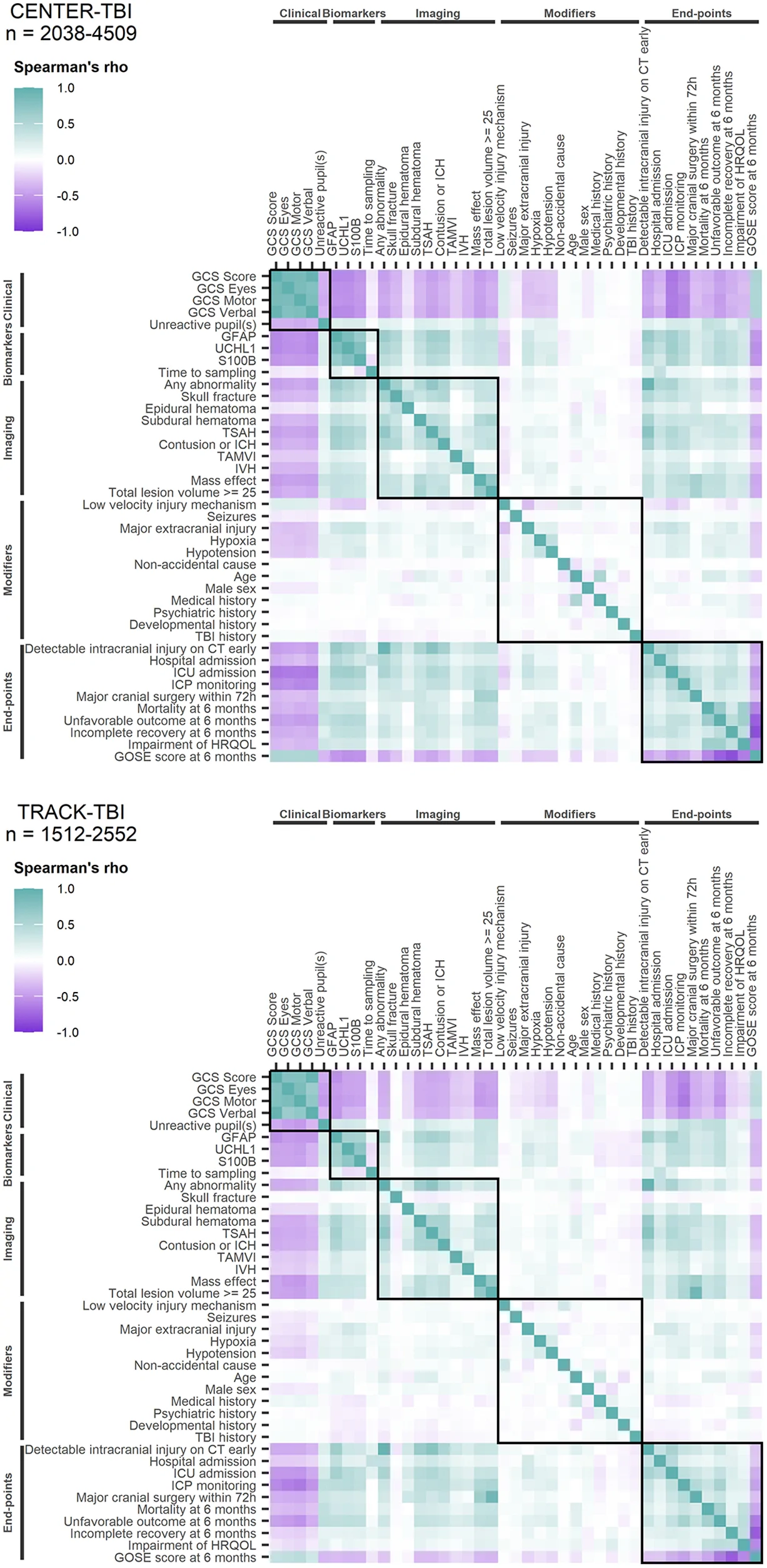
Heatmap of pairwise correlation coefficients between available pillar components and endpoints in CENTER-TBI and TRACK-TBI. Darker color indicates stronger correlations. Abbreviations: GCS, Glasgow Coma Scale; GFAP, glial fibrillary acidic protein; GOSE, Glasgow Outcome Scale – Extended; HRQoL, health-related quality of life; ICH, intracerebral hemorrhage; ICP, intracranial pressure; ICU, intensive care unit; IVH, intraventricular hemorrhage; S100B, S100 calcium-binding protein B; TAMVI, diffuse axonal injury/traumatic axonal injury or punctate hemorrhages; TBI, traumatic brain injury; TSAH, traumatic subarachnoid hemorrhage; UCH-L1, ubiquitin C-terminal hydrolase L1.

### Performance for diagnostic and care path endpoints

The Clinical pillar alone showed high discrimination and explained a large proportion of variability in ICU admission (CENTER-TBI: AUC 0.86 (95% CI [0.85,0.87]); pseudo-R^2^ = 0.57 (95% CI [0.54,0.59]); TRACK-TBI: AUC 0.76 (95% CI [0.74,0.77]); pseudo-R^2^ 0.40 (95% CI [0.37,0.44])). The Biomarker pillar showed strong performance for CT evidence of traumatic intracranial injury (CENTER-TBI: AUC 0.89 (95% CI [0.88,0.90]); pseudo-R^2^ 0.57 (95% CI [0.54,0.60]); TRACK-TBI: AUC 0.86 (95% CI [0.85,0.88]); pseudo-R^2^ 0.50 (95% CI [0.47,0.54]) and for hospital admission (CENTER-TBI: AUC 0.91 (95% CI [0.90,0.92]); pseudo-R^2^ 0.53 (95% CI [0.50,0.57]); TRACK-TBI: AUC 0.87 (95% CI [0.85,0.89]); pseudo-R^2^ 0.45 (95% CI [0.42,0.49]). The Imaging pillar was strongly associated with major cranial surgery within 72 h (CENTER-TBI: AUC 0.93 (95% CI [0.92,0.94]]; pseudo-R^2^ 0.55 (95% CI [0.52,0.59]); TRACK-TBI: AUC 0.98 (95% CI [0.97,0.99]); pseudo-R^2^ 0.80 (95% CI [0.75,0.85])). The Modifier pillar showed poor to fair discrimination for all diagnostic and care pathway endpoints (CENTER-TBI AUC from 0.60 to 0.77; TRACK-TBI from 0.66 to 0.74) with limited explained variability (CENTER-TBI pseudo-R^2^ from 0.03 to 0.29; TRACK-TBI from 0.06 to 0.22).

In cumulative models, adding the Biomarker, Imaging, and Modifier pillars sequentially to the Clinical pillar incrementally improved performance (Table 2, Figs 2 and S2). The Biomarker pillar added substantial incremental value beyond the Clinical pillar for CT abnormalities (CENTER-TBI: AUC 0.90 (95% CI [0.89,0.91]) versus 0.76 (95% CI [0.75,0.78]); pseudo-R^2^ 0.59 (95% CI [0.56,0.62]) versus 0.32 (95% CI [0.30,0.35]); TRACK-TBI: AUC 0.87 (95% CI [0.86,0.89]) versus 0.71 95% CI [0.70,0.73]); pseudo-R^2^ 0.52 (95% CI [0.49,0.56]) versus 0.27 (95% CI [0.23,0.30])), and hospital admission (CENTER-TBI: AUC 0.93 (95% CI [0.92,0.93]) versus 0.78 (95% CI [0.76,0.79]); pseudo-R^2^ 0.58 (95% CI [0.55,0.61]) versus 0.31 (95% CI [0.29,0.33]); TRACK-TBI: AUC 0.87 (95% CI [0.86,0.89]) versus 0.66 (95% CI [0.64,0.68]); pseudo-R^2^ 0.47 (95% CI [0.43,0.51]) versus 0.15 (95% CI [0.14,0.18])). Adding the Imaging pillar to the C and B pillars yielded the largest incremental gains for major cranial surgery (CENTER-TBI: AUC 0.93 (95% CI [0.92,0.94]) versus 0.80 (95% CI [0.79,0.82]); pseudo-R^2^ 0.55 (95% CI [0.52,0.59]) versus 0.24 (95% CI [0.21,0.27]); TRACK-TBI: AUC 0.98 (95% CI [0.97,0.99]) versus 0.89 (95% CI [0.87,0.91]); pseudo-R^2^ 0.80 (95% CI [0.74,0.85]) versus 0.36 (95% CI [0.31,0.41])). The Modifier pillar provided relatively little incremental value for the diagnostic and care pathway endpoints beyond the C + B + I model. When considered together within full CBI-M models, each pillar contributed unique explanatory information beyond the other pillars across all endpoints. In particular, the Biomarker pillar contributed strongly for CT abnormalities and hospital admission, and the Imaging pillar for the endpoint major cranial surgery (Fig 3, S5 Table). Sunburst plots (Fig 3) confirmed the different contributions of pillars across the various endpoints, regardless of the sequence in which pillars were added. Full CBI-M models demonstrated consistently high performance across all diagnostic and care pathway endpoints (CENTER-TBI: AUC from 0.83 to 0.95; pseudo-R^2^ from 0.41 to 0.72; TRACK-TBI: AUC: from 0.88 to 0.98; pseudo-R^2^ = from 0.55 to 0.80; Table 2). The estimated optimism in model performance was small (S6 Table). Results for proportion of explained variability (pseudo-R^2^) were consistent in sensitivity analyses on available cases (S5 Table, S3 and S4 Figs) and largely consistent in subgroup analysis by GCS severity (S5 Fig).

**Table 2:** Discrimination (AUC) and proportion of explained variability (pseudo-R^2^) with 95% confidence intervals [95% CI] for endpoints for cumulative models, adding one pillar at a time, after imputation of missing pillar components.

	CENTER-TBI						TRACK-TBI				
	N Events/ sample	Metric [95% CI]	C pillar	C + B pillars	C + B + I pillars	Full model (CBI-M)	N Events/sample	C pillar	C + B pillars	C + B + I pillars	Full model (CBI-M)
**Diagnostic/Care Path**											
**CT Abnormalities** ^**a**^	2,434/4,086	AUC	0.76 [0.75,0.78]	0.90 [0.89,0.91]		0.90 [0.89,0.91]	1,163/2,445	0.71 [0.70,0.73]	0.87 [0.86,0.89]		0.88 [0.87,0.90]
		**Pseudo-R** ^**2**^	**0.32 [0.30,0.35]**	**0.59 [0.56,0.62]**		**0.59 [0.56,0.62]**		**0.27 [0.23,0.30]**	**0.52 [0.49,0.56]**		**0.55 [0.52,0.59]**
**Hospital Admission**	3,660/4,508	AUC	0.78 [0.76,0.79]	0.93 [0.92,0.93]	0.94 [0.93,0.94]	0.95 [0.94,0.95]	2032/2,552	0.66 [0.64,0.68]	0.87 [0.86,0.89]	0.88 [0.87,0.90]	0.91 [0.90,0.92]
		**Pseudo-R** ^**2**^	**0.31 [0.29,0.33]**	**0.58 [0.55,0.61]**	**0.61 [0.58,0.64]**	**0.65 [0.62,0.68]**		**0.15 [0.14,0.18]**	**0.47 [0.43,0.51]**	**0.49 [0.45,0.53]**	**0.55 [0.52,0.59]**
**ICU Admission**	2,137/4,508	AUC	0.86 [0.85,0.87]	0.93 [0.92,0.93]	0.93 [0.93,0.94]	0.94 [0.94,0.95]	1,160/2,552	0.76 [0.74,0.77]	0.89 [0.88,0.90]	0.90 [0.89,0.92]	0.92 [0.91,0.93]
		**Pseudo-R** ^**2**^	**0.57 [0.54,0.59]**	**0.67 [0.65,0.69]**	**0.69 [0.67,0.71]**	**0.72 [0.70,0.74]**		**0.40 [0.37,0.44]**	**0.58 [0.55,0.61]**	**0.62 [0.59,0.65]**	**0.65 [0.63,0.68]**
**ICP Monitoring**	920/ 2,114	AUC	0.73 [0.71,0.75]	0.78 [0.76,0.80]	0.81 [0.80,0.83]	0.83 [0.81,0.85]	321/1,160	0.87 [0.86,0.89]	0.91 [0.89,0.92]	0.93 [0.92,0.95]	0.94 [0.92,0.95]
		**Pseudo-R** ^**2**^	**0.23 [0.19,0.27]**	**0.31 [0.28,0.36]**	**0.38 [0.34,0.42]**	**0.41 [0.38,0.46]**		**0.51 [0.46,0.56]**	**0.58 [0.53,0.63]**	**0.65 [0.60,0.70]**	**0.65 [0.61,0.70]**
**Major cranial Surgery < 72 h**	573/4,491	AUC	0.78 [0.76,0.80]	0.80 [0.79,0.82]	0.93 [0.92,0.94]	0.93 [0.92,0.94]	213/2,552	0.87 [0.84,0.89]	0.89 [0.87,0.91]	0.98 [0.97,0.99]	0.98 [0.97,0.99]
		**Pseudo-R** ^**2**^	**0.22 [0.19,0.25]**	**0.24 [0.21,0.27]**	**0.55 [0.52,0.59]**	**0.56 [0.52,0.59]**		**0.34 [0.29,0.40]**	**0.36 [0.31,0.41]**	**0.80 [0.74,0.85]**	**0.80 [0.74,0.85]**
**Outcome at 6 months** **post-injury**											
**Mortality**	472/3,810	AUC	0.83 [0.81,0.85]	0.88 [0.86,0.90]	0.90 [0.88,0.91]	0.92 [0.91,0.94]	126/2,122	0.86 [0.82,0.89]	0.92 [0.89,0.94]	0.93 [0.90,0.95]	0.95 [0.93,0.97]
		**Pseudo-R** ^**2**^	**0.32 [0.28,0.36]**	**0.42 [0.38,0.46]**	**0.47 [0.43,0.51]**	**0.55 [0.51,0.59]**		**0.31 [0.25,0.38]**	**0.48 [0.41,0.56]**	**0.51 [0.44,0.58]**	**0.57 [0.50,0.64]**
**Unfavorable Outcome** ^**b**^	970/3,810	AUC	0.80 [0.79,0.82]	0.86 [0.84,0.87]	0.87 [0.86,0.89]	0.89 [0.88,0.90]	325/2,122	0.84 [0.81,0.86]	0.89 [0.87,0.91]	0.90 [0.88,0.92]	0.91 [0.89,0.93]
		**Pseudo-R** ^**2**^	**0.34 [0.31,0.37]**	**0.45 [0.41,0.48]**	**0.48 [0.45,0.52]**	**0.53 [0.50,0.57]**		**0.39 [0.34,0.45]**	**0.50 [0.45,0.55]**	**0.53 [0.48,0.58]**	**0.57 [0.52,0.62]**
**Incomplete recovery** ^**c**^	2,422/3,810	AUC	0.73 [0.71,0.74]	0.79 [0.78,0.81]	0.80 [0.78,0.81]	0.81 [0.80,0.83]	1,514/2,122	0.62 [0.60,0.65]	0.69 [0.66,0.72]	0.69 [0.67,0.72]	0.72 [0.70,0.75]
		**Pseudo-R** ^**2**^	**0.24 [0.21,0.27]**	**0.33 [0.30,0.35]**	**0.34 [0.31,0.36]**	**0.36 [0.33,0.39]**		**0.09 [0.07,0.12]**	**0.14 [0.11,0.17]**	**0.15 [0.12,0.18]**	**0.19 [0.16,0.22]**
**Impairment of HRQoL** ^**d**^	511/2,323	AUC	0.57 [0.55,0.60]	0.62 [0.59,0.65]	0.62 [0.59,0.65]	0.67 [0.65,0.70]	479/1,618	0.50 [0.48,0.54]	0.60 [0.57,0.63]	0.62 [0.59,0.65]	0.70 [0.67,0.73]
		**Pseudo-R** ^**2**^	**0.02 [0.01,0.04]**	**0.05 [0.03,0.08]**	**0.05 [0.03,0.08]**	**0.10 [0.07,0.13]**		**0.00 [0.00,0.01]**	**0.04 [0.02,0.07]**	**0.05 [0.03,0.08]**	**0.15 [0.11,0.20]**

^a^CT Abnormalities: detectable intracranial injury on CT early; ^b^ Unfavorable outcome: Glasgow Outcome Scale – Extended score < 5;

^c^Incomplete recovery: Glasgow Outcome Scale – Extended score < 8;

^d^Impairment of HRQoL: Quality of Life after Brain Injury – Overall Scale total score < 52.

Abbreviations: CT: Computed tomography; HRQoL: health-related quality of life; ICP: intracranial pressure; ICU: intensive care unit.

**Fig 2:**
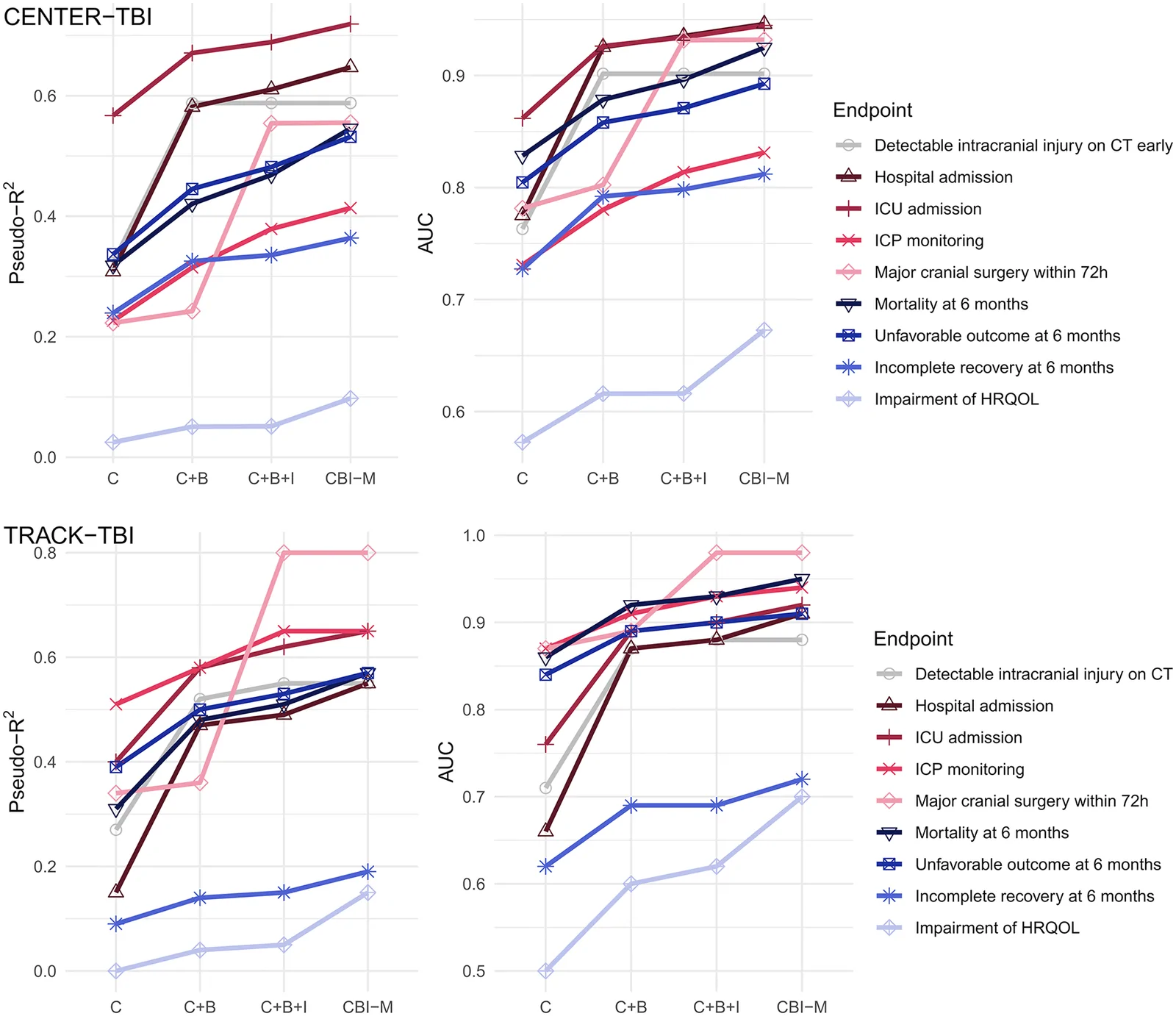
Model performance for cumulative pillar models in CENTER-TBI and TRACK-TBI. Cumulative models were obtained by including the linear predictors of pillar models, not the individual pillar components themselves, as independent variables in multivariable models, starting with the Clinical pillar model (C) and adding one additional pillar at a time. Pillars were added in a fixed order: Clinical (C), Clinical + Biomarker (CB), Clinical + Biomarker + Imaging (CBI), and finally Clinical + Biomarker + Imaging + Modifier (CBI-M). For the diagnostic endpoint, the imaging pillar was excluded from cumulative models. Confidence intervals are reported in Table 2.

**Fig 3:**
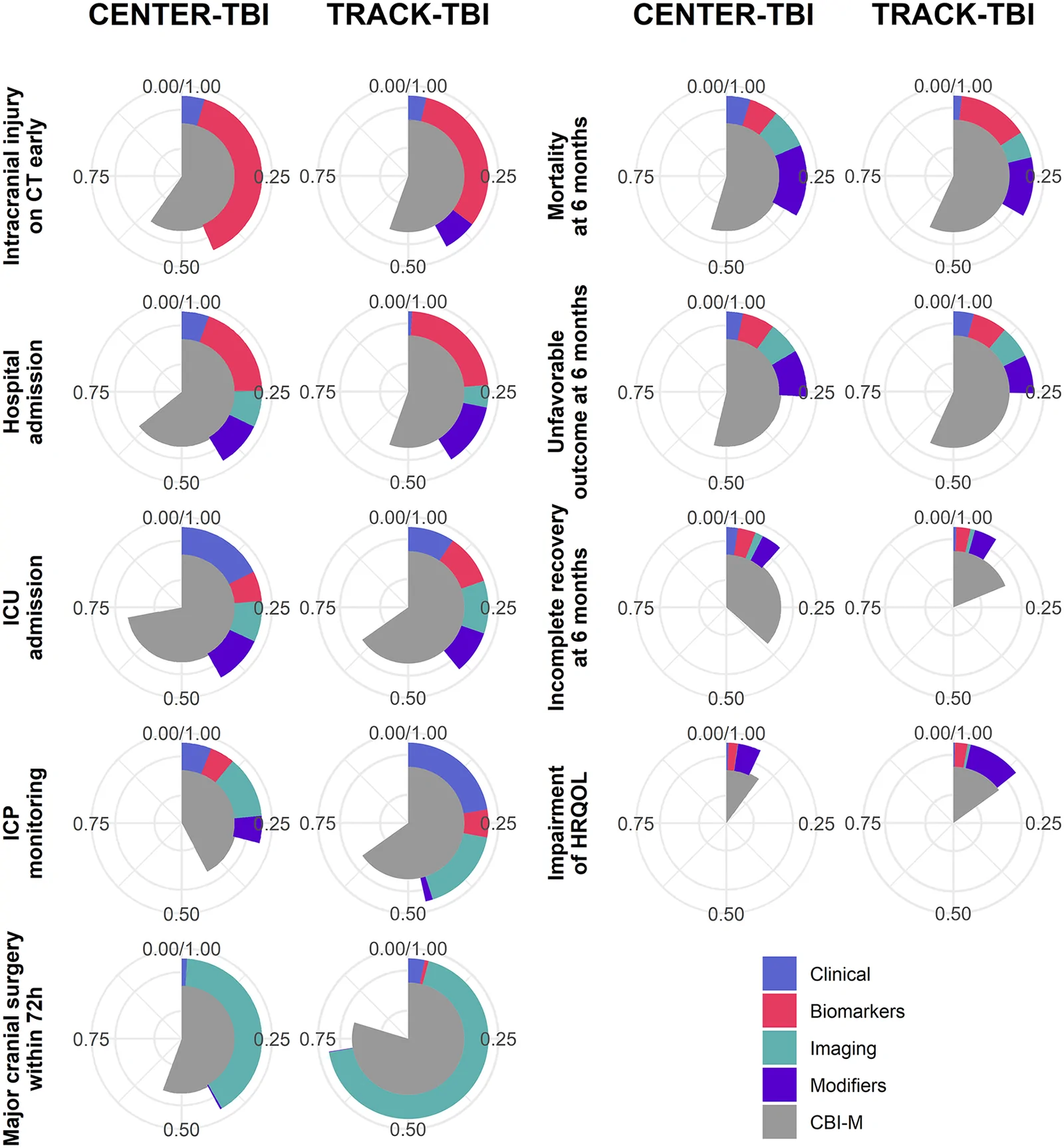
Independent prognostic value of individual pillars within the full CBI-M model for diagnostic, care, path, and outcome endpoints in CENTER-TBI and TRACK-TBI. Sunburst plots of partial pseudo-R^2^s, after imputation of missing pillar components (values with 95% confidence intervals are reported in S5 Table). Each circle represents the total observed variation in the corresponding endpoint (100%). The area of gray inner sectors is proportional to the endpoint variation “explained” by the CBI-M model (Nagelkerke pseudo-R^2^). The area of outer sectors is proportional to the additional unique contribution of each pillar beyond that of the other pillars within the CBI-M model (partial pseudo-R^2^). For example, for the endpoint major cranial surgery within 72 h, when the Imaging pillar is added to a model already containing the Clinical, Biomarker, and Modifier pillars, a substantial amount of previously unexplained variation is “explained” (large green outer section); in contrast, when the Biomarker pillar is added to a model already containing the Clinical, Imaging, and Modifier pillars, almost no additional variation is explained, leading to a virtual null partial pseudo-R^2^ (no red outer sector).

Sensitivity analyses of the associations between the Biomarker pillar and major intracranial surgery in CENTER-TBI patients with biomarkers sampled before (*n* = 84/414) and after surgery (*n* = 330/414) indicated somewhat better discrimination for future surgery (happening after sampling, up to 72 h after injury) than for past surgery (having happened before biomarker sampling, < 24 h of injury; S7 Table). In 55 patients from CENTER-TBI with biomarker levels available before and after surgery, levels of S100B and UCHL-1 after surgery were on average lower compared to pre-surgery, while GFAP remained similar (S7 Table).

### Performance for outcome endpoints

Overall, full CBI-M models reached high predictive performance for commonly reported outcome endpoints in TBI, including mortality, unfavorable outcome and incomplete recovery at 6 months post-injury (CENTER-TBI: AUC from 0.81 to0.92; pseudo-R^2^ from 0.36 to 0.55; TRACK-TBI: AUC from 0.72 to 0.95; pseudo-R^2^ from 0.19 to 0.57), but not for impairment of HRQoL (CENTER-TBI: AUC 0.67 (95% CI [0.65,0.70]); pseudo-R^2^ 0.10 (95% CI [0.07,0.13]); TRACK-TBI: AUC 0.70 (95% CI [0.67,0.73]); pseudo-R^2^ 0.15 (95% CI [0.11,0.20])) (Table 2). Single pillar models also showed high performance for 6-month mortality and unfavorable outcome (CENTER-TBI: AUC from 0.76 to 0.86; pseudo-R^2^ from 0.23 to 0.39; TRACK-TBI: AUC from 0.76 to 0.91; pseudo-R^2^ from 0.21 to 0.46; S6 Fig), but less so for impairment of HRQoL (CENTER-TBI: AUC from 0.57 to 0.64; pseudo-R^2^ from 0.02 to 0.07; TRACK-TBI: AUC from 0.50 to 0.68; pseudo-R^2^ from 0.00 to 0.12), and incomplete recovery (GOSE < 8) in TRACK-TBI (AUC from 0.62 to 0.68; pseudo-R^2^ from 0.08 to 0.13). Cumulative models showed incremental improvement in performance, especially in terms of proportion of explained variability (Table 2, Fig 2). Despite a modest performance of the Modifier pillar compared with other single-pillar models, its unique independent contribution within full CBI-M models for 6-month outcome was substantial (Fig 3). The Modifier pillar provided the largest independent contribution for impairment of HRQoL (partial pseudo-R^2^ 0.05 (95% CI [0.03,0.07]) in CENTER-TBI; 0.11 (95% CI [0.07,0.15]) in TRACK-TBI) (Fig 3, S5 Table, S6 Fig). In the subgroup GCS 13–15, the Modifier pillar explained more variability in unfavorable outcome and impaired HRQoL than any other pillar (S5 Fig).

### Comparison of CBI-M to IMPACT and CRASH prognostic models

The full CBI-M model showed stronger associations with 6-month mortality and unfavorable outcome compared to both the IMPACT-Lab and CRASH-CT models in their respective target populations (Fig 4). Compared with the IMPACT Lab model, the CBI-M pseudo-R^2^ for mortality increased from 0.36 (95% CI [0.32,0.43]) to 0.57 (95% CI [0.51,0.62]) in CENTER-TBI and from 0.47 (95% CI [0.40,0.59]) to 0.62 (95% CI [0.53,0.72]) in TRACK-TBI. For unfavorable outcome, pseudo-R^2^ increased from 0.29 (95% CI [0.25,0.36]) to 0.49 (95% CI [0.43,0.54]) in CENTER-TBI and from 0.44 (95% CI [0.37,0.55]) to 0.55 (95% CI [0.47,0.64]) in TRACK-TBI. Compared with the CRASH-CT model, the CBI-M pseudo-R^2^ for 6-month mortality increased from 0.30 (95% CI [0.26, 0.36]) to 0.55 (95% CI [0.50,0.60]) in CENTER-TBI and from 0.50 (95% CI [0.43,0.0.59]) to 0.60 (95% CI [0.53,0.69]) in TRACK-TBI. For unfavorable outcome, the pseudo-R^2^ increased from 0.25 (95% CI [0.22,0.30] to 0.52 (95% CI [0.47,0.56] in CENTER TBI and from 0.54 (95% CI [0.49,0.62] to 0.61 (95% CI [0.54,0.67]) in TRACK-TBI. For CENTER-TBI, the increase in pseudo-R^2^ and AUC was significant for all 6-month outcomes, as confidence intervals did not overlap (Figs 4; S7).

**Fig 4:**
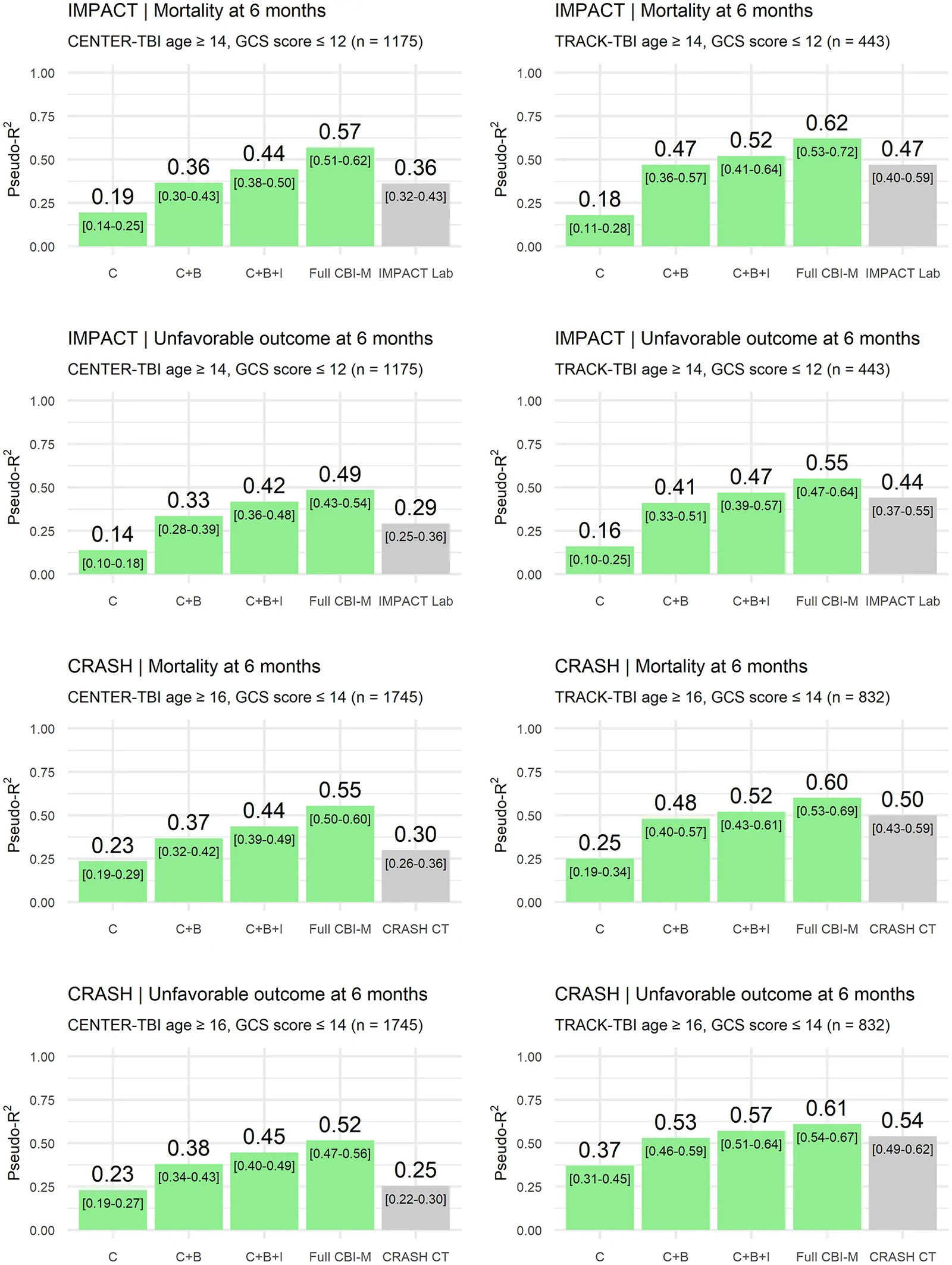
Prognostic value of CBI-M Cumulative Models, adding one pillar at a time, compared with IMPACT Lab and CRASH CT models. Bar plots of Nagelkerke pseudo-R^2^s [95% confidence intervals], after imputation of missing predictors/pillar components. Models were fitted in the IMPACT and CRASH cohorts in CENTER-TBI (left columns) and TRACK-TBI (right columns). IMPACT Lab model: age, Glasgow Coma Scale - motor score, pupils, hypoxia, hypotension, computed tomography (CT) classification, traumatic subarachnoid hemorrhage on CT, epidural mass on CT, glucose (3-20 mmol/L), hemoglobin (6-17 g/dL). CRASH CT model: age, Glasgow Coma Scale score, pupils, major extracranial injury, CT findings: petechial hemorrhages, obliteration of the third ventricle or basal cisterns, traumatic subarachnoid hemorrhage, midline shift, nonevacuated hematoma.

## Discussion

Robust associations between the CBI-M Framework and clinically important endpoints spanning diagnosis, care pathways, and 6-month outcomes were found in two large, contemporary international cohorts across the full spectrum of TBI severity. The relative contribution of each pillar varied by endpoint, consistent with the intended premise of CBI-M as a multimodal characterization framework. For example, the Biomarker pillar was strongly associated with CT abnormalities, while the Imaging pillar showed the strongest association with major cranial surgery. Several analyzed endpoints, including ICU admission, ICP monitoring, and major cranial surgery, are strongly driven in routine clinical practice by variables already embedded within the CBI-M framework, particularly clinical status and imaging findings. Associations with these care pathways likely reflect established clinical decision-making processes, but aptly illustrate the potential relevance of the CBI-M framework to clinical practice. The strong associations between CBI-M pillars and their components with outcome endpoints further support its potential clinical relevance and informational richness of this characterization approach. Our analysis was, however, not designed as a prognostic study. We focused on overall associations and performance, but did not address calibration as we did not aim to develop a prognostic model and considered that beyond the scope of the current exploratory analyses. Not assessing calibration may, however, be considered a limitation. Although we found that the full CBI-M model was more strongly associated with 6-month mortality and unfavorable functional outcome compared to validated TBI prognostic models (i.e., IMPACT [17], CRASH [18]), these comparisons are intended to illustrate potential explanatory capacity rather than to definitively establish direct clinical superiority over existing prognostic tools. Further dedicated modeling, including feature selection and thorough validation is required to derive a more compact CBI-M prognostic model. Such a model should preserve performance while remaining feasible, ideally across all severities, and not only targeted at specific subpopulations, as is the case for the IMPACT and CRASH models.

Differences in case-mix were noted between the CENTER-TBI and TRACK-TBI cohorts: participants in CENTER-TBI were older, had more severe clinical characteristics and more intracranial and extracranial injuries, likely contributing to a higher mortality compared with TRACK-TBI. TRACK-TBI participants had fewer skull fractures and more frequently reported a history of prior TBI. These differences likely reflect at least in part, differences in ascertainment: prior TBI history was captured in TRACK-TBI by a structured assessment tool [23], while skull fractures in CENTER-TBI were mostly scored on 3D bony reconstructions of CT images, which were not routinely performed in TRACK-TBI. While different platforms for biomarker assays were used, this did not affect the results, as analyses were performed in each study separately, using standardized log-transformed values.

Across both datasets, relatively strong correlations were observed within and between the Clinical, Biomarker, and Imaging pillars, while correlations with, and within the Modifier pillar were lower. This pattern reflects how the Framework encompasses two distinct elements of TBI complexity: what the injury brings to the patient and what the patient brings to the injury. Our results suggest that patient-related variables have relatively weak pairwise correlations with the selected endpoints, with the exception of age for 6-month outcome. Although the Modifier pillar contains some injury-related factors, patient-related factors may act to modify the risks associated with injury-related characteristics for specific outcomes, while not necessarily having large independent effect sizes. This conceptual difference between the C, B, I pillars and the Modifier pillar is also captured by the hyphen in designating the CBI-M Framework. Given the primary relevance of the Modifier pillar to recovery and outcome, its components may not all need to be collected directly at presentation. Sunburst plots of partial pseudo-R^2^s, as shown in Fig 3 and S5 Table, showed an independent contribution of the M pillar within the CBI-M model for predicting 6-month outcome, particularly for health-related quality of life (HRQoL), underscoring the importance of this pillar. These results, together with our previous work [24], indicate a potential for using elements from the M-pillar for prediction of HRQoL.

High correlations between clinical presentation and imaging findings are harnessed by clinical decision rules for CT imaging, aimed at limiting unnecessary radiation exposure, overcrowding in the emergency department and high costs in patients with minimal symptomatology [25–27]. Since 2013, the blood-based biomarker S100B has been included in Scandinavian guidelines for CT imaging in the emergency department, while in the United States, the combination of GFAP and UCH-L1 received Food and Drug Administration clearance in 2018 as a test to assist clinicians in determining the need for a CT scan [28,29]. Subsequently, the combination of GFAP and UCH-L1 has also been included in French [30], Spanish [31], and European consensus guidelines [32]. Strong correlations between levels of these three blood biomarkers and generally small independent contributions within Biomarker pillar models suggest some redundancy (S4 Fig), indicating that any of these three biomarkers might suffice in clinical practice. The timing of blood sampling for biomarker assays, which was relatively late in both CENTER-TBI and TRACK-TBI, should be taken into consideration when interpreting these results. As shown in the sunburst plots of S4 Fig, the sampling time had a substantial independent (within-pillar) contribution to explaining hospital admission.

Following the publication introducing the CBI-M Framework, much support has been obtained [33–36], but critiques have also been leveled. Feng and colleagues [37] highlighted, and we acknowledge, that blood-based brain biomarkers are not yet widely available in clinical practice, and that in many cases, such as patients with a deterioration of consciousness, low GCS or signs of brain herniation, urgent CT scanning is indicated and should take priority over the Biomarker pillar. We agree and emphasize that the sequence of pillar assessment should be informed by the clinical condition, in particular, the Clinical pillar. Nevertheless, our findings indicate that biomarker values, which may be obtained later, remain prognostically relevant, including for patients with more severe injuries who require urgent CT scanning.

Brennan and Teasdale [38] called attention to the complexity of the CBI-M Framework, compared with the GCS, and stated that the expert consensus approach underpinning the development of the CBI-M Framework will need to be confirmed by analysis-driven evidence of benefit. They suggest that ease of use and reproducibility should be considered alongside mathematical assessment of performance.

Here, we provide the requested empirical support for the CBI-M Framework. Emerging AI-driven clinical platforms already synthesize complex clinical and imaging data to guide stroke care and with the addition of biomarkers can be readily adapted to develop CBI-M-based TBI care [39].

The different associations of CBI-M pillars and their components with different endpoints have important implications for settings in which complete data from all pillar components of the CBI-M Framework cannot be obtained [40–42]. The modular nature of the CBI-M Framework is a strength, and although there is some redundancy across pillars with some highly associated variables, each pillar also captures unique and complementary information. It is important to note that the sequence of pillar assessments is not fixed, and should be informed by the clinical situation. This is particularly relevant to the M-pillar where some components can be assessed later, as they are likely more relevant to the later recovery course than to the acute management. We also recognize that biomarker assays are not yet broadly available; however, rapid advances in assay technology and point-of-care testing are expected to substantially improve their accessibility, feasibility, and potential for clinical integration in the foreseeable future.

The current results confirm the high potential of the CBI-M Framework, but further work is required prior to broad implementation. Ongoing work that will support broad implementation includes framework optimization (reduction of required components), refinement for specific contexts of use, adaptation to subpopulations, weighting of pillars for the development of numerical scoring systems, and applying the framework for dynamic characterization and prediction across acute, subacute, and chronic stages of TBI. Prospective studies will be required to definitively establish clinical utility and to determine how CBI-M-informed characterization should be operationalized in everyday practice and research.

Our results provide substantial insights regarding the potential usefulness of the CBI-M Framework for characterizing TBI, but it is important to recognize several limitations. Our findings are based on European/North American cohorts mainly treated in level 1 trauma centers. The performance of the Framework may differ in other settings. We analyzed the Framework as originally proposed by the expert panel and refrained from in-depth component and pillar analyses aimed at refinement, variable selection, pillar weighting, and extension or reduction of basic components. The relatively large number of variables included in the full CBI-M models may need to be reduced for practical applicability. Overfitting was limited, as indicated by the small optimism in the AUC and pseudo-R^2^ values estimated for our modeling strategy (i.e., building cumulative models by incorporating linear predictors of single-pillar models, instead of individual predictors). Moreover, results were consistent across the CENTER-TBI and TRACK-TBI cohort*s*. Blood samples were mostly obtained after CT scanning, and biomarkers analyzed many months after enrollment for research purposes—thus, biomarker values could not inform care paths, and statistical associations presented herein may underestimate their relevance for clinical decision-making. In surgically treated patients, reverse causality may have been present, as the first biomarker samples were obtained after surgery in most patients (330/414 in CENTER-TBI, 15/193 in TRACK-TBI), and increased biomarker levels after surgery may then have been influenced by surgical manipulation. In a small subset of 55 patients with samples available before and after surgery, average within-subject changes do not indicate post-operative biomarker elevations. Nonetheless, the effect of surgical intervention on biomarker elevations deserves further dedicated investigation, accounting for lesion phenotype and surgical invasivity (e.g., extra/intra-axial manipulation). We also recognize the current barriers to clinical implementation of the Biomarker pillar. These include lack of standardization across assay platforms, insufficient evidence on cost-effectiveness, and lack of reimbursement by insurance companies. Further work is required to explore effects of age, sex, and time to sampling prior to broad clinical implementation, and to expand the use of these biomarkers beyond their current approved use for CT triage in patients with a GCS 13–15.

Although we included 86% [31/36] of the CBI-M basic components in our analysis, information on six basic components was missing in at least one of the datasets. For the Imaging pillar, this concerned the components “other” and “chronic or incidental lesions.” For the Modifier pillar, missing components were “stressful circumstances”, “language or communication barriers”, “social disadvantage or deprivation” and “no or partial health coverage”. “Stressful circumstances” was replaced by “nonaccidental cause”. The lack of information on three basic components of the Modifier pillar and the use of a proxy variable for a fourth (e.g., nonaccidental injury for “stressful circumstances”) may have incompletely captured the intended constructs of the Modifier pillar, potentially attenuating its observed contribution in these analyses.

In cumulative models, incremental improvement in performance is dependent on our chosen sequence of adding pillars, aimed to reflect clinical workflow. Partial pseudo-R^2^ measures, as presented in Fig 3, capture the unique contribution of each pillar beyond the other three pillars, and complement those obtained in cumulative models. The consistency of results obtained on independent analyses of the CENTER-TBI and TRACK-TBI cohorts strongly supports the robustness of our findings.

In summary, the CBI-M Framework and its pillars are strongly associated with clinically relevant TBI endpoints pertaining to diagnosis, care pathways, and 6-month outcomes after TBI. The consistency of the findings across CENTER-TBI and TRACK-TBI supports the robustness of the CBI-M framework as a potential multimodal approach to TBI characterization across the injury spectrum. When used prognostically for 6-month outcome, the full CBI-M model was more strongly associated with 6-month mortality and unfavorable functional outcome compared to the IMPACT and CRASH models. Our findings support further optimization and prospective validation of the CBI-M Framework for characterization of patients with TBI as a next step towards implementation across clinical care and research settings.

## Supporting information

S1 Table Basic CBI-M pillar components and their availability in CENTER-TBI and TRACK-TBI datasets. (PDF)

S2 Table Multiple imputation. (PDF)

S3 Table STROBE statement. (PDF)

S4 Table Distribution of endpoints in CENTER-TBI and TRACK-TBI. (PDF)

S5 Table Comparison of (partial) Nagelkerke pseudo-R^2^s as displayed in the sunburst plots of Fig 3 between available case analysis and analysis in imputed dataset. (PDF)

S6 Table Optimism in discrimination (area under the curve) and proportion of explained variability (pseudo-R^2^) for the full CBI-M model in CENTER-TBI and TRACK-TBI, after imputation of missing pillar components. (PDF)

S7 Table Sensitivity analyses of the association between biomarkers and major intracranial surgery. (PDF)

S1 Fig Heatmap of pairwise correlation coefficients between pillar components and endpoints in available cases in CENTER-TBI and TRACK-TBI. (PDF)

S2 Fig Explained variability and discrimination for diagnostic and care path endpoints by Pillars and CBI-M model in CENTER-TBI and TRACK-TBI. (PDF)

S3 Fig Available case analysis: Explained variability for diagnostic, care paths and outcome endpoints by Pillars and CBI-M model in CENTER-TBI and TRACK-TBI. (PDF)

S4 Fig Independent prognostic value of CBI-M pillar components - Sunburst plots of partial pseudo-R^2^s – available case analysis. (PDF)

S5 Fig Explained variability in endpoints by pillars and CBI-M model in CENTER-TBI and TRACK-TBI, differentiated by TBI severity strata: Mild (GCS 13–15) versus Moderate/Severe (GCS 3–12). (PDF)

S6 Fig Explained variability and discrimination for outcome endpoints by Pillars and CBI-M model in CENTER-TBI and TRACK-TBI. (PDF)

S7 Fig Prognostic value of CBI-M Cumulative Models, adding one pillar at a time, compared with IMPACT Lab and CRASH CT models: Bar plot of AUCs, after imputation of missing predictors/pillar components. (PDF)

S1 Text List of contributors. (PDF)

S1 Protocol Study protocol CENTER-TBI. (PDF)

S2 Protocol TRACK-TBI U01 clinical protocol. (PDF)

## Abbreviations

CB: Clinical + Biomarker
CBI: Clinical + Biomarker + Imaging
CIs: confidence intervals
CT: computed tomography
GCS: Glasgow Coma Scale
GOSE: Glasgow Outcome Scale-Extended
HRQoL: health-related quality of life
ICP: intracranial pressure
ICU: intensive care unit
MAR: missing at random
TBI: traumatic brain injury
US: United States.
