# Supplementary material for: The CBI-M framework for characterization of traumatic brain injury: Evaluation of performance using data from the CENTER-TBI and TRACK-TBI prospective cohort studies
Source: PLoS Med. 2026 Oct 20;23(10):e1004945. doi: 10.1371/journal.pmed.1004945 (PMC13681731; doi:10.1371/journal.pmed.1004945)
Supplement: Supplementary file 1 [file pmed.1004945.s001.pdf]

S1 Table: Basic CBI-M pillar components and their availability in CENTER-TBI and TRACK-TBI datasets.

Basic Pillar Component	Available in CENTER-TBI and TRACK-TBI
**Clinical pillar**	
Glasgow Coma Scale (GCS) score	✓
GCS Eye score	✓
GCS Verbal score	✓
GCS Motor score	✓
Pupillary reactivity	✓
**Biomarker pillar**	
Glial fibrillary acidic protein (GFAP)	✓
Ubiquitin C-terminal hydrolase L1 (UCH-L1)	✓
S100 calcium-binding protein B (S100B)	✓
**Imaging pillar**	
Clinically not indicated	✓ (exclusion criterion)
No abnormalities detected	✓
Epidural haematoma	✓
Subdural haematoma	✓
Traumatic subarachnoid haemorrhage	✓
Contusion	✓
Intracerebral haematoma	✓
Traumatic axonal and microvascular injury	✓
Intraventricular haemorrhage	✓
Skull fracture	✓
Lesion volume	✓
Mass effect	✓
Other lesions (evidence of recent craniotomy, penetrating traumatic injury, ventriculomegaly, vascular)	✗
Chronic or incidental lesions	✗ (only available for CENTER-TBI)
**Modifier pillar**	
Mechanism of injury	✓
Comorbid extracranial injury	✓
Secondary insults (hypoxia, hypotension)	✓
Early post-traumatic seizure	✓
Stressful circumstances	✗ Non-accidental cause of injury used as proxy
Age	✓
Sex	✓
Medical history and medication	✓
Mental health condition	✓
Developmental history	✓
TBI history	✓
Language or communication barrier	✗
Social disadvantage or deprivation	✗
No or partial health-care insurance	✗
