# Supplementary material for: The CBI-M framework for characterization of traumatic brain injury: Evaluation of performance using data from the CENTER-TBI and TRACK-TBI prospective cohort studies
Source: PLoS Med. 2026 Oct 20;23(10):e1004945. doi: 10.1371/journal.pmed.1004945 (PMC13681731; doi:10.1371/journal.pmed.1004945)
Supplement: Supplementary file 2 [file pmed.1004945.s002.pdf]

S2 Table. Multiple imputation

**Multiple imputation**	
**Assumed missingness mechanism**	Missing at random (MAR)
**Variables used in the imputation model**	All pillar components (in case of composite variables- the variables used to derive them), all endpoints, all IMPACT and CRASH variables, auxiliary variables
Pillar components	All variables;<br><br>If composite variables- individual variables used to derive the composite e.g. total lesion volume ≥25 ml derived from total epidural/subdural/intraparenchymal hematoma volumes
IMPACT and CRASH variables	Glucose, hemoglobin, Marshall CT classification, obliteration of the third ventricle or basal cisterns
Endpoints	All endpoints
Auxiliary variables	History of stroke/TIA, history of seizures, history of neurodegenerative disorders/MS, alcohol intoxication
**Imputation models**	
Continuous	pmm
Binary	logreg
Other categorical	polyreg
**Number of imputed datasets**	5
