# Supplementary material for: The CBI-M framework for characterization of traumatic brain injury: Evaluation of performance using data from the CENTER-TBI and TRACK-TBI prospective cohort studies
Source: PLoS Med. 2026 Oct 20;23(10):e1004945. doi: 10.1371/journal.pmed.1004945 (PMC13681731; doi:10.1371/journal.pmed.1004945)
Supplement: Supplementary file 3 [file pmed.1004945.s003.pdf]

S3 Table: STROBE Statement—Checklist of items included in reports of cohort studies.

	Item No	Recommendation	Page No/ Section
**Title and abstract**	1	(*a*) Indicate the study's design with a commonly used term in the title or the abstract	Abstract
		(*b*) Provide in the abstract an informative and balanced summary of what was done and what was found	Abstract
**Introduction**			
Background/rationale	2	Explain the scientific background and rationale for the investigation being reported	Introduction-paragraph 1
Objectives	3	State specific objectives, including any prespecified hypotheses	Introduction-paragraph 2
**Methods**			
Study design	4	Present key elements of study design early in the paper	Methods- Study design and population
Setting	5	Describe the setting, locations, and relevant dates, including periods of recruitment, exposure, follow-up, and data collection	Methods- Study design and population; Data collection and management; Endpoints
Participants	6	(*a*) Give the eligibility criteria, and the sources and methods of selection of participants. Describe methods of follow-up	Methods- Study design and population; Endpoints
		(*b*) For matched studies, give matching criteria and number of exposed and unexposed	NA
Variables	7	Clearly define all outcomes, exposures, predictors, potential confounders, and effect modifiers. Give diagnostic criteria, if applicable	Methods- Endpoints
Data sources/ measurement	8*	For each variable of interest, give sources of data and details of methods of assessment (measurement). Describe comparability of assessment methods if there is more than one group	Methods- data collection and management, Table 1; S1 Table
Bias	9	Describe any efforts to address potential sources of bias	Methods- Data analyses
Study size	10	Explain how the study size was arrived at	NA

Quantitative variables	11	Explain how quantitative variables were handled in the analyses. If applicable, describe which groupings were chosen and why	Methods- Data analyses, paragraph 4
Statistical methods	12	(*a*) Describe all statistical methods, including those used to control for confounding	Methods- Data analyses, paragraphs 4 and 5
		(*b*) Describe any methods used to examine subgroups and interactions	Methods- Study Design and Population, paragraph 2; Data analyses, paragraphs 4 and 5
		(*c*) Explain how missing data were addressed	Methods- Data analyses, paragraph 3; S2 Table
		(*d*) If applicable, explain how loss to follow-up was addressed	Methods- Data analyses, paragraph 3
		(*e*) Describe any sensitivity analyses	Methods- Study Design and Population, paragraph 2; Data analyses, paragraphs 4 and 5
Participants	13*	(a) Report numbers of individuals at each stage of study—eg numbers potentially eligible, examined for eligibility, confirmed eligible, included in the study, completing follow-up, and analysed	Results- Study populations and patient characteristics
		(b) Give reasons for non-participation at each stage	NA
		(c) Consider use of a flow diagram	NA
Descriptive data	14*	(a) Give characteristics of study participants (eg demographic, clinical, social) and information on exposures and potential confounders	Results- Study populations and patient characteristics; Table 1
		(b) Indicate number of participants with missing data for each variable of interest	Table 1; S4 Table
		(c) Summarise follow-up time (eg, average and total amount)	Results- Study populations and patient characteristics

Outcome data	15*	Report numbers of outcome events or summary measures over time	S4 Table; Table 2
**Results**			
Main results	16	(*a*) Give unadjusted estimates and, if applicable, confounder-adjusted estimates and their precision (eg, 95% confidence interval). Make clear which confounders were adjusted for and why they were included	Results-Performance for diagnostic and care path endpoints, Performance for outcome endpoints, Comparison of CBI-M to IMPACT and CRASH prognostic models; Table 2, Figure 4, S7 Figure, S6 Table
		(*b*) Report category boundaries when continuous variables were categorized	Table 1
		(*c*) If relevant, consider translating estimates of relative risk into absolute risk for a meaningful time period	NA
Other analyses	17	Report other analyses done—eg analyses of subgroups and interactions, and sensitivity analyses	Results-Performance for diagnostic and care path endpoints, Performance for outcome endpoints; S5 Table S3, S4, S5 and S6 Figure
**Discussion**			
Key results	18	Summarise key results with reference to study objectives	Discussion-paragraph 1
Limitations	19	Discuss limitations of the study, taking into account sources of potential bias or imprecision. Discuss both direction and magnitude of any potential bias	Discussion-paragraphs 10,11,12

Interpretation	20	Give a cautious overall interpretation of results considering objectives, limitations, multiplicity of analyses, results from similar studies, and other relevant evidence	Discussion
