# Supplementary material for: The CBI-M framework for characterization of traumatic brain injury: Evaluation of performance using data from the CENTER-TBI and TRACK-TBI prospective cohort studies
Source: PLoS Med. 2026 Oct 20;23(10):e1004945. doi: 10.1371/journal.pmed.1004945 (PMC13681731; doi:10.1371/journal.pmed.1004945)
Supplement: Supplementary file 4 [file pmed.1004945.s004.pdf]

S4 Table: Distribution of endpoints in CENTER-TBI and TRACK-TBI.

	**CENTER-TBI**		**TRACK-TBI**	
**N Overall**	**4509**[a]	**% Missing**	**2552**	**% Missing**
**Diagnostic and care paths endpoints**				
Detectable traumatic intracranial injury on CT early (%)	2435 (59.6)	9.4	1163 (47.6)	4.2
Hospital admission (%)	3661 (81.2)	0	2032 (79.6)	0
ICU admission (%)	2138 (47.4)	0	1160 (45.5)	0
ICP monitoring[b] (%)	924 (42.6)	51.9	321 (27.7)	54.5[a]
Major cranial surgery within 72h (%)	574 (12.8)	0.4	213 (8.3)	0
**6-month outcome endpoints**				
GOSE[c] score (%)		15.5		16.8
Dead	473 (12.4)		126 (5.9)	
Vegetative state / Lower severe disability	320 (8.4)		155 (7.3)	
Upper severe disability	178 (4.7)		44 (2.1)	
Lower moderate disability	355 (9.3)		249 (11.7)	
Upper moderate disability	414 (10.9)		426 (20.1)	
Lower good recovery	683 (17.9)		514 (24.2)	
Upper good recovery	1388 (36.4)		608 (28.7)	
Mortality (%)	473 (12.4)	15.5	126 (5.9)	16.8
Unfavourable outcome, GOSE[c] score ≤ 4 (%)	971 (25.5)	15.5	325 (15.3)	16.8
Incomplete recovery, GOSE[c] score ≤ 7 (%)	2423 (63.6)	15.5	1514 (71.3)	16.8
Impairment of HRQoL, QoLIBRI OS total score < 52 (%)	511 (22.0)	48.5	479 (29.6)	36.6

Abbreviations: GOSE, Glasgow Outcome Scale – Extended; HRQoL, health-related quality of life; ICP, intracranial pressure; ICU, intensive care unit; QoLIBRI OS, Quality of Life after Brain Injury – Overall Scale.

[a]The main analysis was performed in patients with observed endpoints and imputed missing pillar components. In CENTER-TBI, the imputation algorithm failed for a single patient with almost all pillar components missing. Despite having recorded endpoints, this patient was excluded from the main analysis, which is why the sample sizes differ by one unit compared to this table.

[b]Only assessed as an endpoint in the subgroup of patients admitted to the intensive care unit;

[c]GOSE scores were assessed by in-person/telephone interviews or postal questionnaires. A clear distinction between GOSE scores 2 and 3 was not possible for assessments performed by postal questionnaire. As such, these 2 categories were combined. When GOSE ratings were not available for the 6-month time window, but ratings from other time points were available, missing values were imputed centrally using a multi-state model.
