# Supplementary material for: The CBI-M framework for characterization of traumatic brain injury: Evaluation of performance using data from the CENTER-TBI and TRACK-TBI prospective cohort studies
Source: PLoS Med. 2026 Oct 20;23(10):e1004945. doi: 10.1371/journal.pmed.1004945 (PMC13681731; doi:10.1371/journal.pmed.1004945)
Supplement: Supplementary file 5 [file pmed.1004945.s005.pdf]

S5. Table: Comparison of (partial) Nagelkerke pseudo-R^2s (95% Confidence Intervals) as displayed in the sunburst plots of Figure 3 between available case analysis and analysis in imputed dataset. Independent prognostic value of individual pillars within the full CBI-M model (partial pseudo-R^2) for diagnostic, care pathways and outcome endpoints in CENTER-TBI and TRACK-TBI.

	**CENTER-TBI**					**TRACK-TBI**				
	**Available cases**									
**Endpoint**	**CBIM**	**C**	**B**	**I**	**M**	**CBIM**	**C**	**B**	**I**	**M**
**Detectable intracranial injury on CT early**	0.57 (0.54,0.61)	0.04 (0.02,0.07)	0.37 (0.33,0.42)	N/A	0.00 (0.00,0.00)	0.56 (0.52,0.60)	0.04 (0.02,0.07)	0.33 (0.28,0.38)	N/A	0.08 (0.05,0.120
**Hospital admission**	0.68 (0.64,0.72)	0.06 (0.03,0.10)	0.20 (0.15,0.25)	0.09 (0.05,0.13)	0.13 (0.09,0.17)	0.51 (0.47,0.56)	0.01 (0.01,0.02)	0.20 (0.15,0.26)	0.04 (0.02,0.07)	0.13 (0.11,0.16)
**ICU admission**	0.73 (0.71,0.76)	0.18 (0.14,0.22)	0.06 (0.04,0.09)	0.10 (0.07,0.15)	0.12 (0.08,0.17)	0.62 (0.59,0.66)	0.07 (0.04,0.11)	0.09 (0.06,0.14)	0.12 (0.08,0.16),	0.10 (0.06,0.14)
**ICP monitoring**	0.51 (0.46,0.57)	0.08 (0.04,0.13)	0.07 (0.03,0.11)	0.16 (0.11,0.22)	0.06 (0.03,0.11)	0.72 (0.66,0.77)	0.25 (0.17,0.34)	0.05 (0.01,0.11)	0.27 (0.17,0.37)	0.01 (0,0.06)
**Major cranial surgery within 72h**	0.57 (0.52,0.63)	0.01 (0.00,0.03)	0.00 (0.00,0.01)	0.43 (0.37,0.49)	0.00 (0.00,0.01)	0.85 (0.81,0.91)	0.02 (0,0.1)	0.03 0,0.11)	0.78 (0.71,0.86)	0.00 (0,0.05)
**Mortality at 6 months**	0.51 (0.45,0.58)	0.04 (0.01,0.08)	0.08 (0.04,0.13)	0.08 (0.04,0.13)	0.15 (0.10,0.21)	0.52 (0.41,0.66)	0.01 0,0.06)	0.07 0.01,0.18)	0.10 0.02,0.22)	0.09 0.03,0.17)
**Unfavourable outcome at 6 months**	0.51 (0.46,0.56)	0.03 (0.01,0.06)	0.07 (0.04,0.11)	0.07 (0.04,0.10)	0.07 (0.04,0.11)	0.55 (0.47,0.62)	0.05 0.02,0.1)	0.03 0.01,0.07)	0.08 (0.04,0.14)	0.07 (0.03,0.13)
**Incomplete recovery at 6 months**	0.35 (0.31,0.40)	0.01 (0.00,0.03)	0.05 (0.03,0.07)	0.02 (0.01,0.03)	0.05 (0.03,0.07)	0.18 (0.14,0.22)	0.01 0,0.02)	0.03 0.01,0.05)	0.01 (0,0.02)	0.06 0.03,0.09)
**Impairment of HRQoL at 6 months**	0.13 (0.09,0.18)	0.01 (0.00,0.03)	0.01 (0.00,0.03)	0.00 (0.00,0.01)	0.07 (0.04,0.11)	0.16 (0.12,0.22)	0.00 (0,0.02)	0.03 (0.01,0.06)	0.01 (0,0.02)	0.11 (0.07,0.15)
	**Analysis in imputed dataset[a]**									
**Endpoint**	**CBIM**	**C**	**B**	**I**	**M**	**CBIM**	**C**	**B**	**I**	**M**
**Detectable intracranial injury on CT early**	0.60 (0.57,0.62)	0.05 (0.03,0.06)	0.39 (0.36,0.42)	N/A	0.00 (0.00,0.00)	0.55 (0.52,0.59)	0.04 (0.02,0.06)	0.31 (0.27,0.35)	N/A	0.07 (0.05,0.1)
**Hospital admission**	0.64 (0.61,0.67)	0.05 (0.04,0.08)	0.19 (0.16,0.23)	0.07 (0.05,0.10)	0.09 (0.07,0.12)	0.56 (0.52,0.59)	0.01 (0,0.02)	0.22 (0.18,0.27)	0.05 (0.03,0.08)	0.13 0.11,0.15)

**ICU admission**	0.72 (0.70,0.74)	0.18 (0.15,0.21)	0.06 (0.04,0.08)	0.08 (0.06,0.11)	0.10 (0.07,0.13)	0.66 (0.63,0.68)	0.09 (0.06,0.11)	0.09 (0.06,0.11)	0.12 (0.09,0.15)	0.09 (0.06,0.12)
**ICP monitoring**	0.42 (0.39,0.47)	0.06 (0.04,0.09)	0.05 (0.03,0.08)	0.13 (0.09,0.16)	0.05 (0.03,0.08)	0.65 (0.61,0.7)	0.23 (0.17,0.29)	0.04 (0.02,0.09)	0.17 (0.11,0.24)	0.02 (0,0.05)
**Major cranial surgery within 72h**	0.56 (0.52,0.59)	0.01 (0.00,0.02)	0.00 (0.00,0.00)	0.41 (0.37,0.45)	0.00 (0.00,0.01)	0.83 (0.79,0.87)	0.03 (0,0.08)	0.02 (0,0.07)	0.73 (0.67,0.8)	0.00 (0,0.01)
**Mortality at 6 months**	0.55 (0.51,0.59)	0.05 (0.03,0.07)	0.06 (0.04,0.09)	0.08 (0.05,0.11)	0.14 (0.11,0.18)	0.58 (0.52,0.65)	0.02 (0,0.06)	0.15 (0.09,0.23)	0.06 (0.02,0.12)	0.12 (0.06,0.19)
**Unfavourable outcome at 6 months**	0.54 (0.51,0.57)	0.03 (0.02,0.05)	0.07 (0.04,0.09)	0.07 (0.05,0.09)	0.09 (0.07,0.12)	0.57 (0.52,0.62)	0.04 0.02,0.07)	0.07 (0.03,0.1)	0.07 (0.04,0.11)	0.08 (0.05,0.11)
**Incomplete recovery at 6 months**	0.36 (0.34,0.40)	0.02 (0.01,0.04)	0.04 (0.02,0.05)	0.02 (0.01,0.03)	0.04 (0.03,0.06)	0.19 (0.16,0.22)	0.00 (0,0.02)	0.02 (0.01,0.04)	0.01 (0,0.03)	0.05 (0.03,0.07)
**Impairment of HRQoL at 6 months**	0.10 (0.07,0.13)	0.00 (0.00,0.01)	0.02 (0.01,0.04)	0.00 (0.00,0.01)	0.05 (0.03,0.07)	0.15 (0.11,0.19)	0.00 (0,0.01)	0.02 (0.01,0.04)	0.01 (0,0.02)	0.11 (0.07,0.15)

Abbreviations: CT, computed tomography; HRQoL, health-related quality of life; ICP, intracranial pressure; ICU, intensive care unit.

[a]Calculations performed in 1 imputed dataset, thus, CBI-M pseudo,R^2 differs slightly from the value in the rest of the analyses, where it was pooled over 5 imputed datasets.

The partial pseudo-R^2s values presented here underpin the sunburst plots in Figure 3. The partial pseudo-R^2s of pillars presented here and in Figure 3 are different from t he delta pseudo-R^2s that can be calculated between cumulative models in S2, S3, S6 Figs. Cumulative models are built progressively, starting from the C pillar and adding 1 extra pillar at each step (CB, then CBI, then CBI,M). Each pillar addition usually leads to a better fitting model (progressively higher grey bars, delta pseudo,R^2 usually >0 between adjacent models). The partial pseudo-R^2 on the other hand, represents the proportion of unexplained variability after applying a reduced 3-pillar model (BIM, CIM, CBM, CBI) that can be explained by the addition of the fourth missing pillar (leading to the full CBI-M model). As such, the partial pseudo,R^2 is meant to show the different independent contribution of each pillar across all endpoints, accounting for the contribution of the other 3 pillars in the CBI-M full model.
