# Supplementary material for: The CBI-M framework for characterization of traumatic brain injury: Evaluation of performance using data from the CENTER-TBI and TRACK-TBI prospective cohort studies
Source: PLoS Med. 2026 Oct 20;23(10):e1004945. doi: 10.1371/journal.pmed.1004945 (PMC13681731; doi:10.1371/journal.pmed.1004945)
Supplement: Supplementary file 6 [file pmed.1004945.s006.pdf]

S6 Table: Optimism in discrimination (area under the curve) and proportion of explained variability (pseudo-R^2) for the full CBI-M model in CENTER-TBI and TRACK-TBI, after imputation of missing pillar components.

Endpoint	CENTER-TBI					TRACK-TBI				
	N events/ sample	Area under the curve		Pseudo-R^2		N events/ sample	Area under the curve		Pseudo-R^2	
		Optimism[a]	Apparent (Corrected)	Optimism[a]	Apparent (Corrected)		Optimism[a]	Apparent (Corrected)	Optimism[a]	Apparent (Corrected)
Detectable intracranial injury on CT early[b]	2434/4086	0.002	0.90 (0.90)	0.006	0.59 (0.58)	1163/2445	0.000	0.88 (0.88)	0.000	0.55 (0.55)
Hospital admission	3660/4508	0.003	0.95 (0.94)	0.013	0.65 (0.64)	2032/2552	0.000	0.91 (0.91)	0.000	0.55 (0.55)
ICU admission	2137/4508	0.001	0.94 (0.94)	0.005	0.72 (0.71)	1160/2552	0.000	0.92 (0.92)	0.000	0.65 (0.65)
ICP monitoring	920/2114	0.008	0.83 (0.82)	0.024	0.41 (0.39)	321/1160	0.002	0.94 (0.93)	0.008	0.65 (0.64)
Major cranial surgery within 72h	573/4491	0.002	0.93 (0.93)	0.009	0.56 (0.55)	213/2552	0.004	0.98 (0.98)	0.034	0.80 (0.76)
Mortality at 6 months	472/3810	0.003	0.92 (0.92)	0.014	0.55 (0.53)	126/2122	0.001	0.95 (0.95)	0.005	0.57 (0.56)
Unfavorable outcome at 6 months	970/3810	0.003	0.89 (0.89)	0.011	0.53 (0.52)	325/2122	0.001	0.91 (0.91)	0.002	0.57 (0.57)
Incomplete recovery at 6 months	2422/3810	0.005	0.81 (0.81)	0.012	0.36 (0.35)	1514/2122	0.002	0.72 (0.72)	0.005	0.19 (0.18)
Impairment of HRQOL at 6 months	511/2323	0.023	0.67 (0.65)	0.033	0.10 (0.06)	479/1618	0.002	0.70 (0.70)	0.004	0.15 (0.15)

Abbreviations: CT, computed tomography; ICP, intracranial pressure; ICU, intensive care unit; HRQoL, health-related quality of life.

[a]Optimism in discrimination (area under the curve) and overall fit (pseudo-R^2) for the full CBI-M model was estimated by bootstrapping using 1000 bootstrap samples drawn with replacement from the multiply imputed dataset.

[b]For the diagnostic endpoint, the imaging pillar was excluded (CB-M instead of CBI-M model).

To reduce model complexity, the full CBI-M model (as well as the other reduced CB, CBI cumulative models) was built using the linear predictors of pre-specified pillar-models. This strategy was chosen to reduce the number of estimated degrees of freedom of cumulative models and hereby reduce the risk of overfitting. As such, even if each pillar model is re-fit within each bootstrap resample, the full CBI-M model's optimism remains relatively small.
