# Supplementary material for: The CBI-M framework for characterization of traumatic brain injury: Evaluation of performance using data from the CENTER-TBI and TRACK-TBI prospective cohort studies
Source: PLoS Med. 2026 Oct 20;23(10):e1004945. doi: 10.1371/journal.pmed.1004945 (PMC13681731; doi:10.1371/journal.pmed.1004945)
Supplement: Supplementary file 7 [file pmed.1004945.s007.pdf]

S7 Table. Sensitivity analyses of the association between biomarkers and major intracranial surgery

<table>
<tr><th colspan="5">Association between biomarker levels and major intracranial surgery in patients from CENTER-TBI with available biomarker levels and sampling time</th></tr>
<tr><td colspan="2">Major intracranial surgery</td><td>Yes</td><td>No</td><td>AUC [95% confidence interval]</td></tr>
<tr><td colspan="2">Regardless of sampling time[1]</td><td>414</td><td>2825</td><td>0.75 [0.73 - 0.77]</td></tr>
<tr><td colspan="2">Biomarker sampling before surgery[2]</td><td>84</td><td>2825</td><td>0.80 [0.78 - 0.86]</td></tr>
<tr><td colspan="2">Biomarker sampling after surgery[3]</td><td>330</td><td>2825</td><td>0.75 [0.74 - 0.78]</td></tr>
<tr><th colspan="5">Changes in biomarker levels before and after surgery in a subset of patients from CENTER-TBI with available biomarkers before and after surgery (n=55/84)[2]</th></tr>
<tr><td>Biomarker</td><td colspan="3">Within-subject change
median [percentile 25th, percentile 75th]</td><td>paired Wilcoxon test on
log biomarker values
p value</td></tr>
<tr><td>GFAP</td><td colspan="3">-1 ng/ml
[p.25, p75: -19, 12]</td><td>0.973</td></tr>
<tr><td>S100B</td><td colspan="3">-0.16 ng/ml
[p.25, p75: -0.50, 0]</td><td><0.001</td></tr>
<tr><td>UCH-L1</td><td colspan="3">-106 pg/ml
[p.25, p75: -421, 36]</td><td><0.001</td></tr>
</table>

[1] First biomarker sampling occurring anytime within 24h after injury;

[2] Surgery happening after the first biomarker sampling, up to 72h after injury;

[3] Surgery having happened before the first biomarker sampling.
