# Supplementary material for: The CBI-M framework for characterization of traumatic brain injury: Evaluation of performance using data from the CENTER-TBI and TRACK-TBI prospective cohort studies
Source: PLoS Med. 2026 Oct 20;23(10):e1004945. doi: 10.1371/journal.pmed.1004945 (PMC13681731; doi:10.1371/journal.pmed.1004945)
Supplement: Supplementary file 8 [file pmed.1004945.s008.pdf]

S1 Figure: Heatmap of pairwise correlation coefficients between pillar components and endpoints in available cases in CENTER-TBI (upper panel) and TRACK-TBI (lower panel). *Darker colour indicates stronger correlations.*

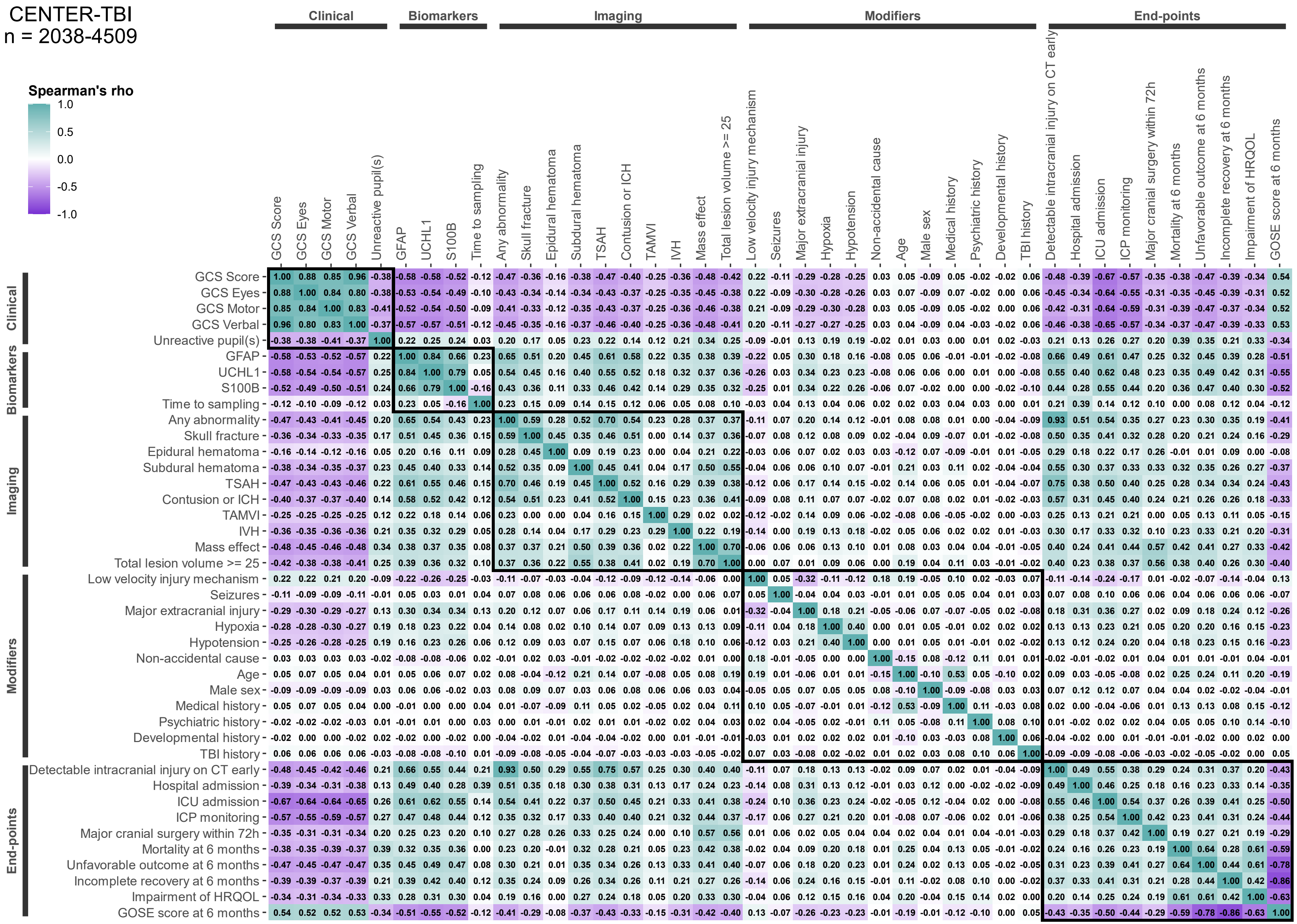

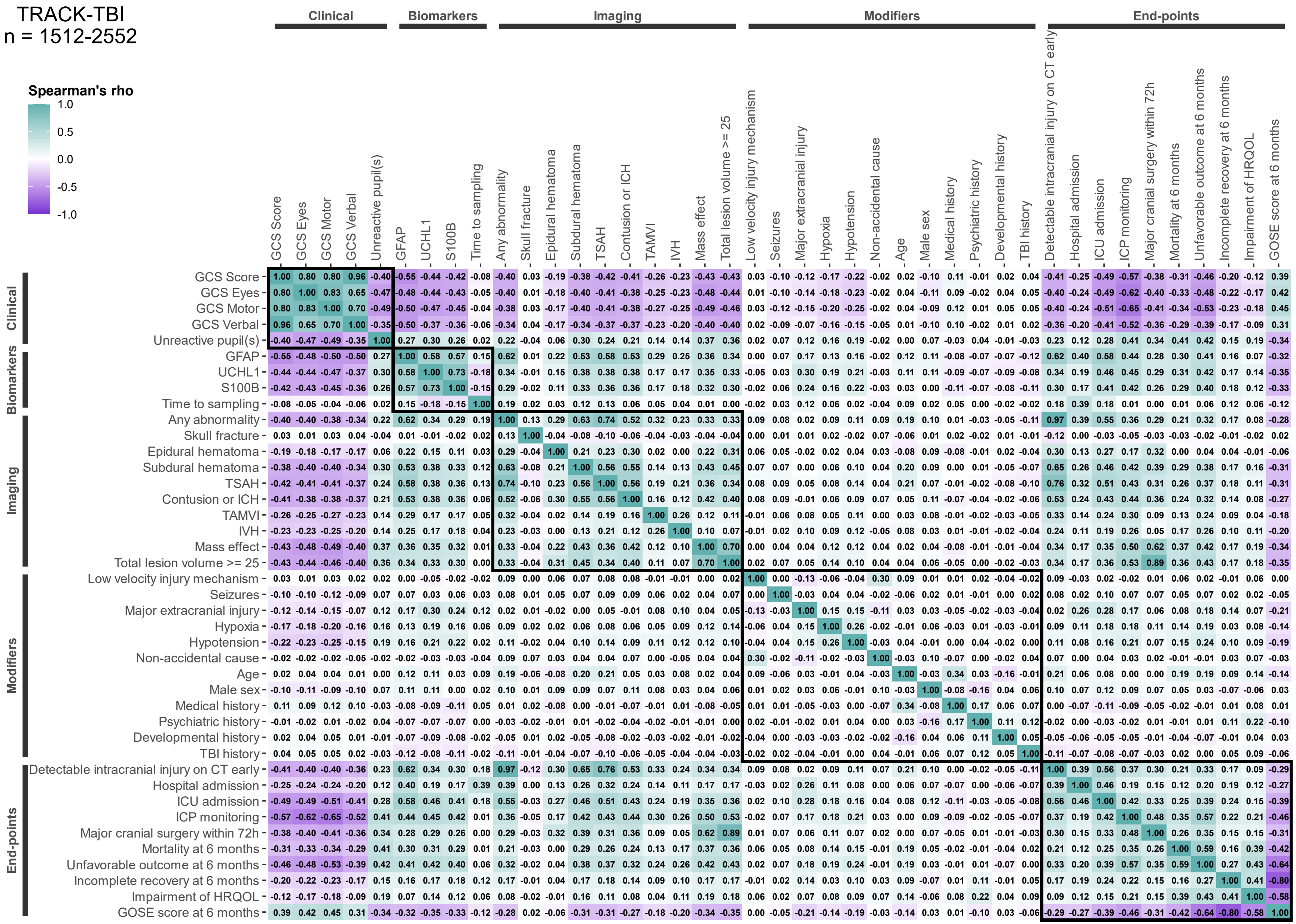

Abbreviations: GCS, Glasgow Coma Scale; GFAP, glial fibrillary acidic protein; GOSE, Glasgow Outcome Scale – Extended; HRQoL, health-related quality of life; ICH, intracerebral haemorrhage; ICP, intracranial pressure; ICU, intensive care unit; IVH, intraventricular haemorrhage; S100B, S100 calcium-binding protein B; TAMVI, diffuse axonal injury/traumatic axonal injury or punctate haemorrhages; TBI, traumatic brain injury; TSAH, traumatic subarachnoid haemorrhage; UCH-L1, ubiquitin C-terminal hydrolase L1.
