# Supplementary material for: The CBI-M framework for characterization of traumatic brain injury: Evaluation of performance using data from the CENTER-TBI and TRACK-TBI prospective cohort studies
Source: PLoS Med. 2026 Oct 20;23(10):e1004945. doi: 10.1371/journal.pmed.1004945 (PMC13681731; doi:10.1371/journal.pmed.1004945)
Supplement: Supplementary file 9 [file pmed.1004945.s009.pdf]

S2 Figure: Explained variability and discrimination for diagnostic and care path endpoints by Pillars and CBI-M model in CENTER-TBI and TRACK-TBI – Bar plots of Nagelkerke pseudo-R^2 and area under the curve measures of single-pillar models and cumulative models, adding one Pillar at a time, after imputation of missing Pillar components.

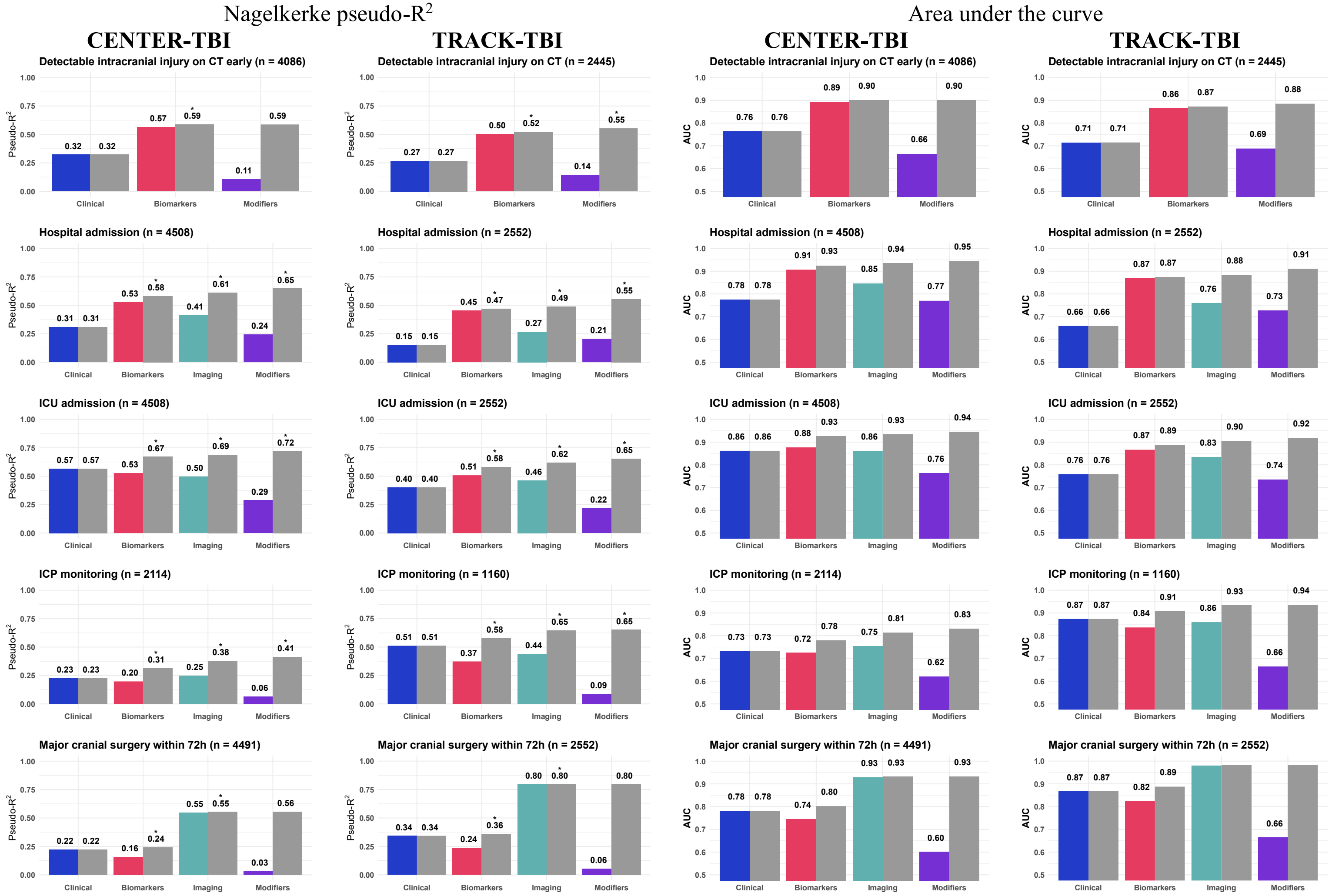

Coloured bars: single pillar models. Grey bars: cumulative models.

In cumulative models (grey bars), pillars were added in a fixed order: clinical (C), clinical+biomarker (CB), clinical+biomarker+imaging (CBI) and finally clinical+biomarker+imaging+ modifier (CBI-M). Cumulative models were obtained by including the linear predictors of pillar models as independent variables in multivariable models, starting with the clinical pillar model (C) and adding one extra pillar at a time (CB, CBI, and CBI-M). Improvement in model fit with the addition of each extra pillar linear predictor (CB vs. C, CBI vs. CB, CBI-M vs. CBI) was tested with the likelihood ratio test (1 degree of freedom). Significant improvement (likelihood ratio test p-value < 0.05) is indicated with an asterix above the corresponding more complex model. For the diagnostic endpoint, the imaging pillar was excluded from cumulative models.
