# Supplementary material for: The CBI-M framework for characterization of traumatic brain injury: Evaluation of performance using data from the CENTER-TBI and TRACK-TBI prospective cohort studies
Source: PLoS Med. 2026 Oct 20;23(10):e1004945. doi: 10.1371/journal.pmed.1004945 (PMC13681731; doi:10.1371/journal.pmed.1004945)
Supplement: Supplementary file 10 [file pmed.1004945.s010.pdf]

S3 Figure: Available case analysis: Explained variability for diagnostic, care paths and outcome endpoints by pillars and CBI-M model in CENTER-TBI and TRACK-TBI – Bar plots of Nagelkerke pseudo-R^2 measures of single-pillar models and cumulative models, adding one pillar at a time in available cases.

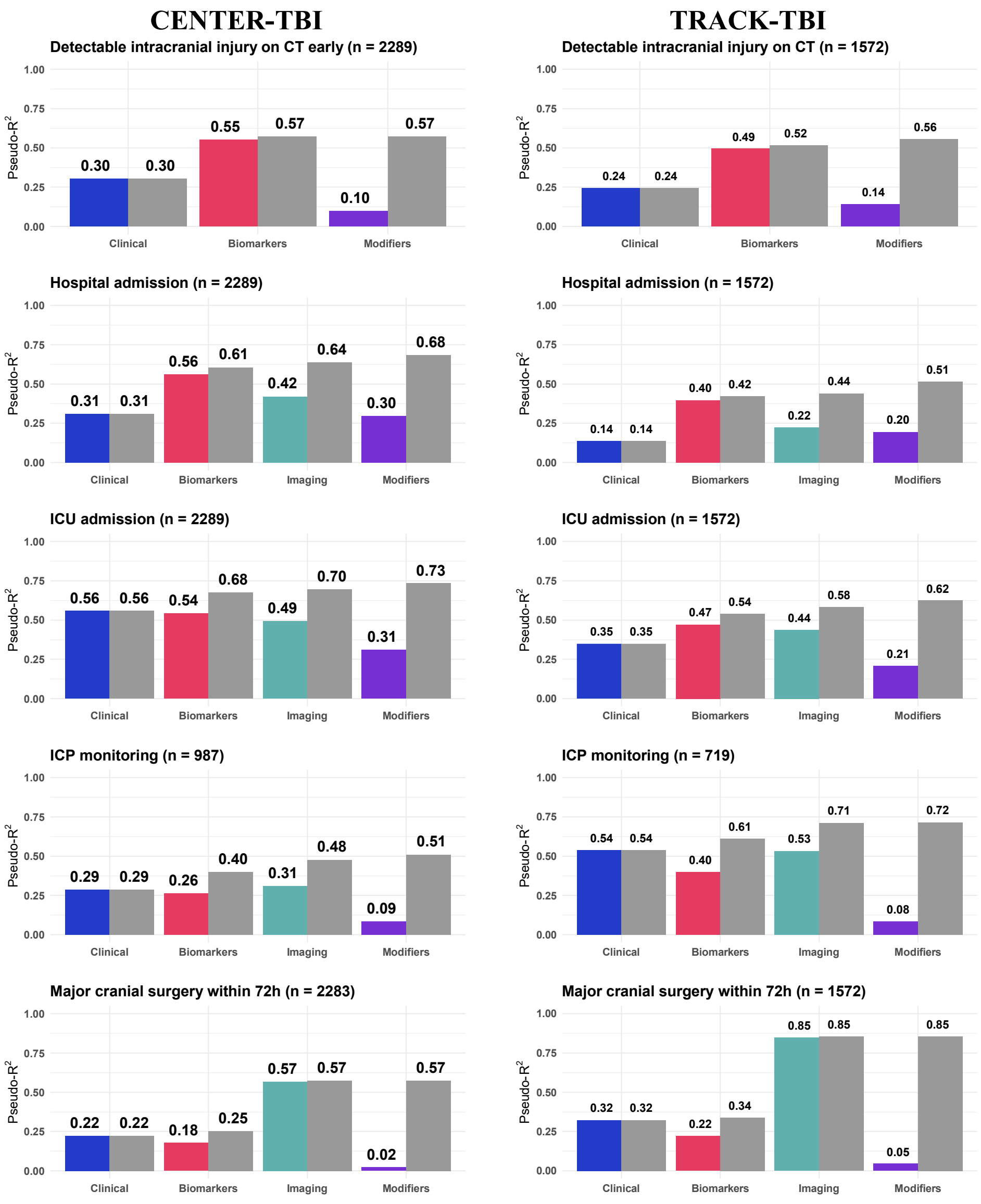

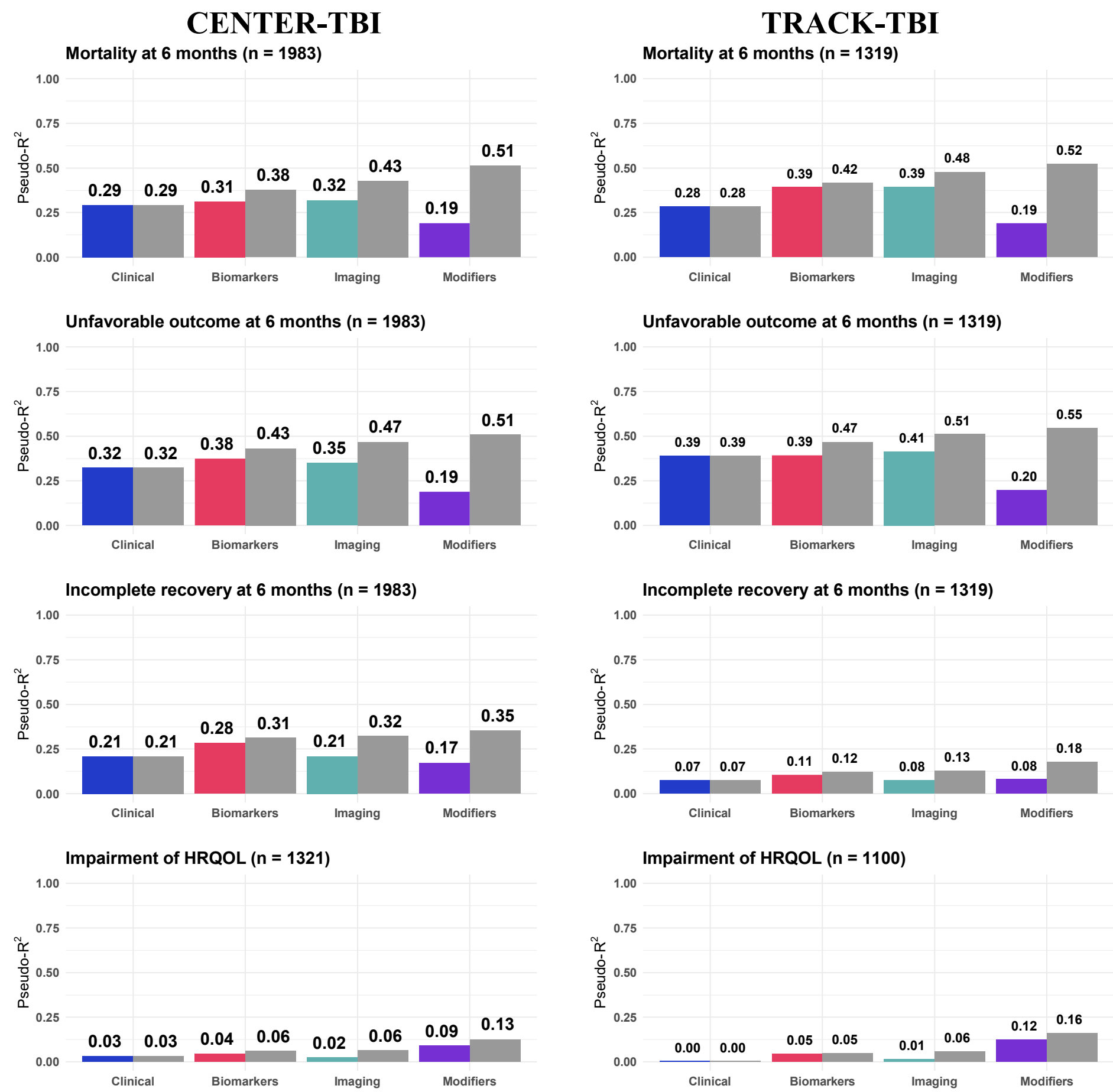

Legend: Coloured bars: single pillar models. Grey bars: cumulative models.
In cumulative models (grey bars), pillars were added in a fixed order: clinical (C), clinical+biomarker (CB), clinical+biomarker+imaging (CBI) and finally clinical+biomarker+imaging+modifier (CBI-M). Cumulative models were obtained by including the linear predictors of pillar models as independent variables in multivariable models, starting with the clinical pillar model (C) and adding one extra pillar at a time (CB, CBI, and CBI-M). For the diagnostic endpoint, the imaging pillar was excluded from cumulative models.
