# Supplementary material for: The CBI-M framework for characterization of traumatic brain injury: Evaluation of performance using data from the CENTER-TBI and TRACK-TBI prospective cohort studies
Source: PLoS Med. 2026 Oct 20;23(10):e1004945. doi: 10.1371/journal.pmed.1004945 (PMC13681731; doi:10.1371/journal.pmed.1004945)
Supplement: Supplementary file 11 [file pmed.1004945.s011.pdf]

S4 Figure: Independent prognostic value of CBI-M pillar components – Sunburst plots of partial pseudo-R^2s - available case analysis.

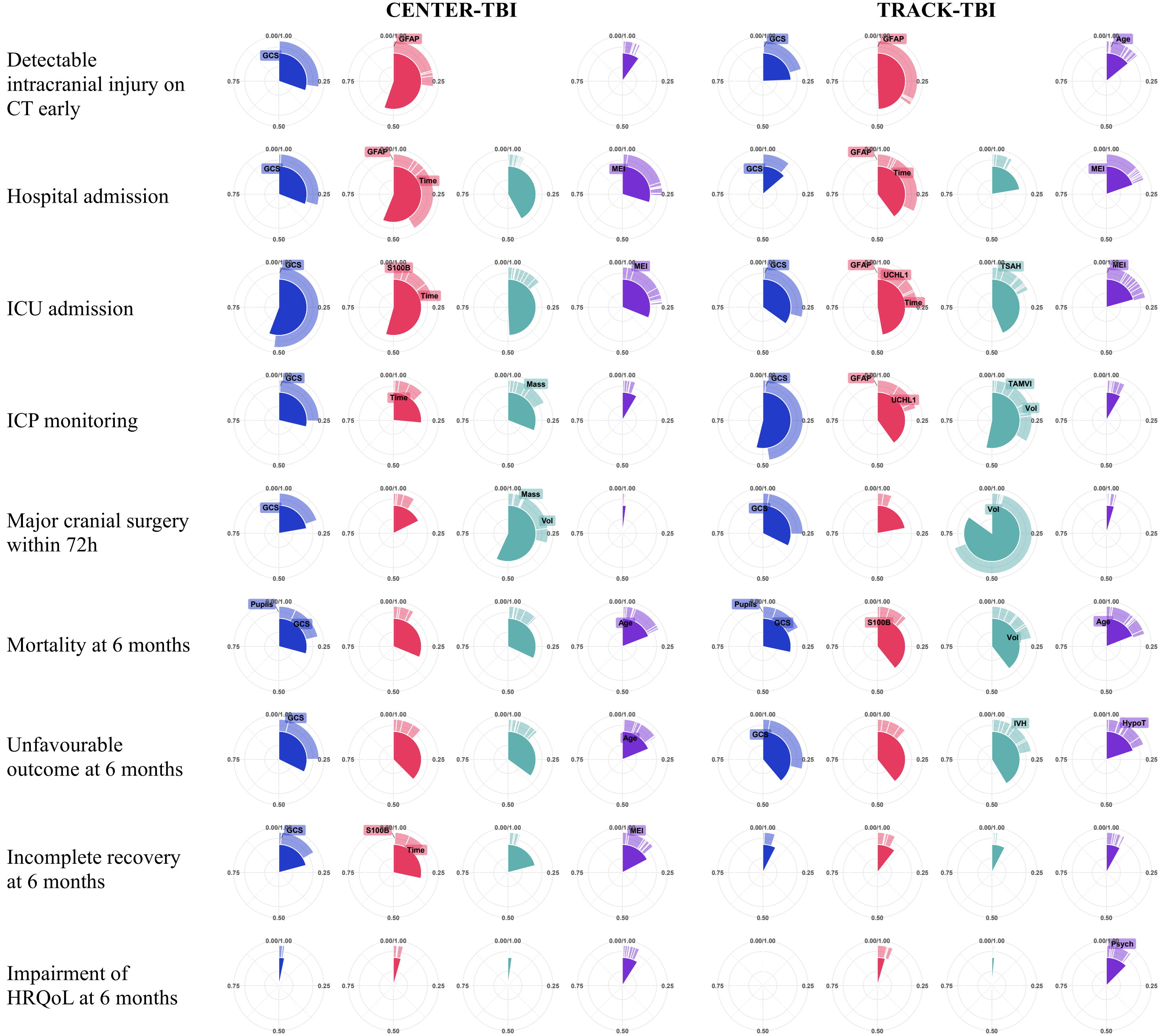

Legend:

The darker inner section is proportional to the explained variability by the C, B, I or M pillar model (pseudo-R^2). Outer sections are proportional to the independent additional contribution of each component beyond all other components in the pillar (partial pseudo-R^2).

Generally, individual components showed distinct additional contributions beyond other components across the different endpoints. Some components showed a strong unique contribution beyond other components, such as GFAP for the diagnostic endpoint and MEI for hospital admission.
