# Supplementary material for: The CBI-M framework for characterization of traumatic brain injury: Evaluation of performance using data from the CENTER-TBI and TRACK-TBI prospective cohort studies
Source: PLoS Med. 2026 Oct 20;23(10):e1004945. doi: 10.1371/journal.pmed.1004945 (PMC13681731; doi:10.1371/journal.pmed.1004945)
Supplement: Supplementary file 12 [file pmed.1004945.s012.pdf]

S5 Figure: Explained variability in endpoints by pillars and CBI-M model in CENTER-TBI and TRACK-TBI, differentiated by TBI severity strata: Mild (GCS 13-15) vs Moderate/Severe (GCS 3-12) - Bar plots of Nagelkerke pseudo-R^2 measures of single-pillar models and cumulative models, adding one pillar at a time, fitted in severity subgroups after imputation of missing pillar components.

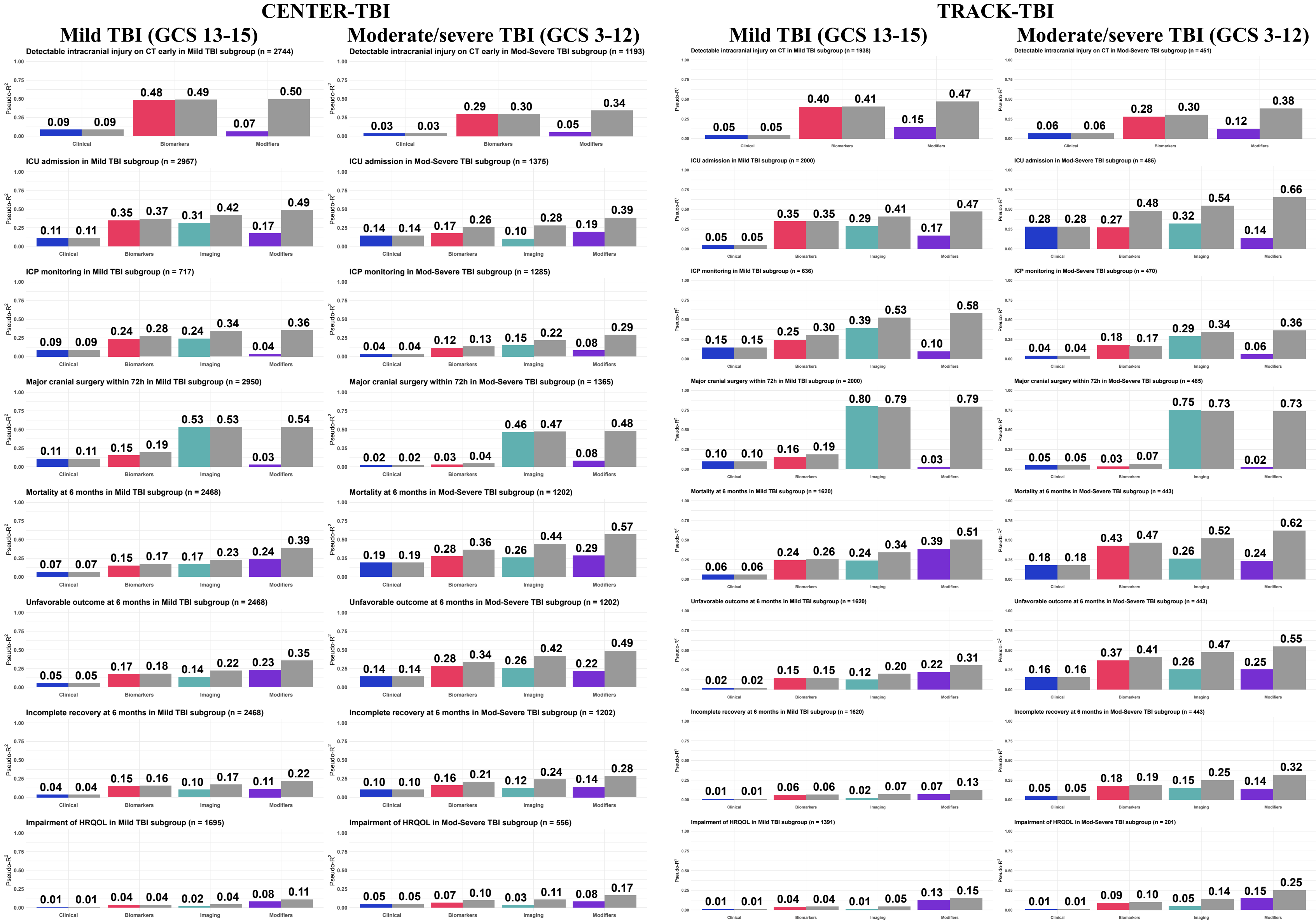

Coloured bars: single pillar models. Grey bars: cumulative models.

In cumulative models (grey bars), pillars were added in a fixed order: clinical (C), clinical+biomarker (CB), clinical+biomarker+imaging (CBI) and finally clinical+biomarker+imaging+ modifier (CBI-M). Cumulative models were obtained by including the linear predictors of pillar models as independent variables in multivariable models, starting with the clinical pillar model (C) and adding one extra pillar at a time (CB, CBI, and CBI-M). For the diagnostic endpoint, the imaging pillar was excluded from cumulative models.

Analyses were performed when at least 10 outcome events and 10 non-events were observed in each severity subgroup (the endpoint hospital admission was not analysed because in each study <10 patients with GCS 3-12 were not admitted).
