# Supplementary material for: The CBI-M framework for characterization of traumatic brain injury: Evaluation of performance using data from the CENTER-TBI and TRACK-TBI prospective cohort studies
Source: PLoS Med. 2026 Oct 20;23(10):e1004945. doi: 10.1371/journal.pmed.1004945 (PMC13681731; doi:10.1371/journal.pmed.1004945)
Supplement: Supplementary file 14 [file pmed.1004945.s014.pdf]

S7 Figure: Prognostic value of CBI-M Cumulative Models, adding one pillar at a time, compared with IMPACT Lab[1] and CRASH CT[2] models, when fitted in the IMPACT and CRASH cohorts in CENTER-TBI (left columns) and TRACK-TBI (right columns) - Bar plots of area under the curve metrics [95% confidence intervals], after imputation of missing predictors/pillar components.

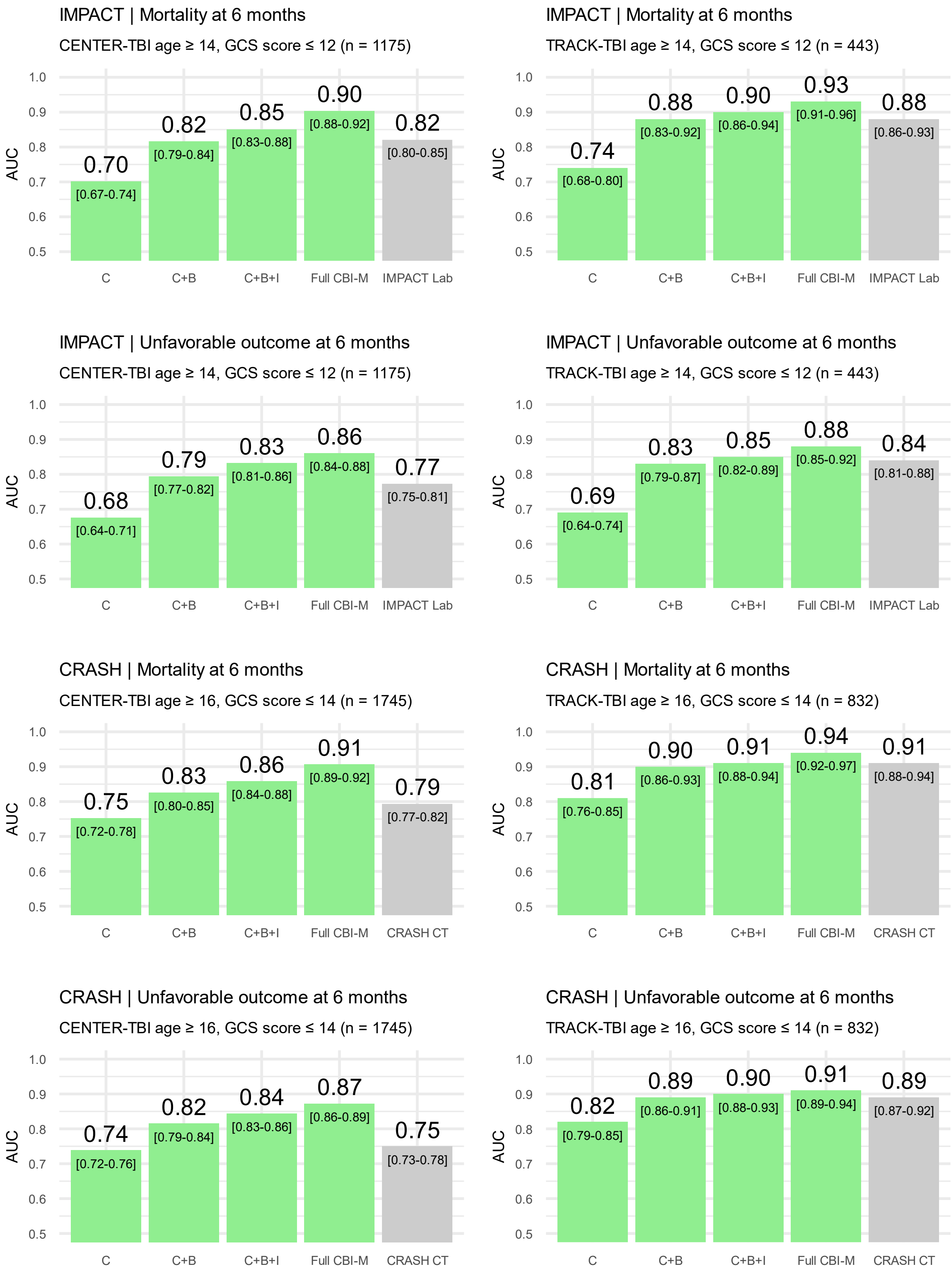

[1]Age, Glasgow Coma Scale - motor score, pupils, hypoxia, hypotension, computed tomography (CT) classification, traumatic subarachnoid hemorrhage on CT, epidural mass on CT, glucose (3-20 mmol/L), hemoglobin (6-17 g/dL);
[2]Age, Glasgow Coma Scale score, pupils, major extracranial injury, CT findings: petechial hemorrhages, obliteration of the third ventricle or basal cisterns, traumatic subarachnoid hemorrhage, midline shift, non-evacuated hematoma.
