# Supplementary material for: The CBI-M framework for characterization of traumatic brain injury: Evaluation of performance using data from the CENTER-TBI and TRACK-TBI prospective cohort studies
Source: PLoS Med. 2026 Oct 20;23(10):e1004945. doi: 10.1371/journal.pmed.1004945 (PMC13681731; doi:10.1371/journal.pmed.1004945)
Supplement: Supplementary file 15 [file pmed.1004945.s015.pdf]

List of Contributors

The CENTER-TBI investigators

Cecilia Åkerlund[1], Krisztina Amrein[2], Nada Andelic[3], Lasse Andreassen[4], Audny Anke[5], Anna Antoni[6], Gérard Audibert[7], Philippe Azouvi[8], Maria Luisa Azzolini[9], Ronald Bartels[10], Pál Barzó[11], Romuald Beauvais[12], Ronny Beer[13], Bo-Michael Bellander[14], Antonio Belli[15], Habib Benali[16], Maurizio Berardino[17], Luigi Beretta[9], Morten Blaabjerg[18], Peter Bragge[19], Alexandra Brazinova[20], Vibeke Brinck[21], Joanne Brooker[22], Camilla Brorsson[23], Andras Buki[24], Monika Bullinger[25], Manuel Cabeleira[26], Alessio Caccioppola[27], Emiliana Calappi [27], Maria Rosa Calvi[9], Peter Cameron[28], Guillermo Carbayo Lozano[29], Marco Carbonara[27], Simona Cavallo[17], Giorgio Chevallard[30], Arturo Chieregato[30], Giuseppe Citerio[31, 32], Hans Clusmann[33], Mark Coburn[34], Jonathan Coles[35], Jamie D. Cooper[36], Marta Correia[37], Amra Čović [38], Nicola Curry[39], Endre Czeiter[40], Marek Czosnyka[26], Claire Dahyot-Fizelier[41], Paul Dark[42], Helen Dawes[43], Véronique De Keyser[44], Vincent Degos[16], Francesco Della Corte[45], Hugo den Boogert[10], Bart Depreitere[46], Đula Đilvesi [47], Abhishek Dixit[48], Emma Donoghue[22], Jens Dreier[49], Guy-Loup Dulière[50], Ari Ercole[48], Patrick Esser[43], Erzsébet Ezer[51], Martin Fabricius[52], Valery L. Feigin[53], Kelly Foks[54], Shirin Frisvold[55], Alex Furmanov[56], Pablo Gagliardo[57], Damien Galanaud[16], Dashiell Gantner[28], Guoyi Gao[58], Pradeep George[59], Alexandre Ghuysen[60], Lelde Giga[61], Ben Glocker[62], Jagoš Golubovic[47], Pedro A. Gomez [63], Johannes Gratz[64], Benjamin Gravesteijn[65], Francesca Grossi[45], Russell L. Gruen[66], Deepak Gupta[67], Juanita A. Haagsma[65], Iain Haitsma[68], Raimund Helbok[69,70], Eirik Helseth[71], Lindsay Horton [72], Jilske Huijben[65], Peter J. Hutchinson[73], Bram Jacobs[74], Stefan Jankowski[75], Mike Jarrett[21], Ji-yao Jiang[59], Faye Johnson[76], Kelly Jones[53], Mladen Karan[47], Angelos G. Kolias[73], Erwin Kompanje[77], Daniel Kondziella[52], Evgenios Kornaropoulos[48], Lars-Owe Koskinen[78], Noémi Kovács[79], Ana Kowark[80], Alfonso Lagares[63], Linda Lanyon[59], Steven Laureys[81], Fiona Lecky[82, 83], Didier Ledoux[81], Rolf Lefering[84], Valerie Legrand[85], Aurelie Lejeune[86], Leon Levi[87], Roger Lightfoot[88], Hester Lingsma[65], Andrew I.R. Maas[44,89], Ana M. Castaño-León[63], Marc Maegele[90], Marek Majdan[20], Alex Manara[91], Geoffrey Manley[92], Costanza Martino[93], Hugues Maréchal[50], Julia Mattern[94], Catherine McMahon[95], Béla Melegh[96], David Menon[48], Tomas Menovsky[44,89], Ana Mikolic[65], Benoit Misset[81], Visakh Muraleedharan[59], Lynnette Murray[28], Ancuta Negru[97], David Nelson[1], Virginia Newcombe[48], Daan Nieboer[65], József Nyirádi[2], Otesile Olubukola[82], Matej Oresic[98], Fabrizio Ortolano[27], Aarno Palotie[99, 100, 101], Paul M. Parizel[102], Jean-François Payen[103], Natascha Perera[12], Vincent Perlbarg[16], Paolo Persona[104], Wilco Peul[105], Anna Piippo-Karjalainen[106], Matti Pirinen[99], Dana Pisica[65], Horia Ples[97], Suzanne Polinder[65], Inigo Pomposo[29], Jussi P. Posti [107], Louis Puybasset[108], Andreea Radoi [109], Arminas Ragauskas[110], Rahul Raj[106], Malinka Rambadagalla[111], Isabel Retel Helmrich[65], Jonathan Rhodes[112], Sylvia Richardson[113], Sophie Richter[48], Samuli Ripatti[99], Saulius Rocka[110], Cecilie Roe[114], Olav Roise[115,116], Jonathan Rosand[117], Jeffrey V. Rosenfeld[118], Christina Rosenlund[119], Guy Rosenthal[56], Rolf Rossaint[80], Sandra Rossi[104], Daniel Rueckert[62] Martin Rusnák[120], Juan Sahuquillo[109], Oliver Sakowitz[94, 121], Renan Sanchez-Porras[121], Janos Sandor[122], Nadine Schäfer[84], Silke Schmidt[123], Herbert Schoechl[124], Guus Schoonman[125], Rico Frederik Schou[126], Elisabeth Schwendenwein[6], Charlie Sewalt[65], Ranjit D. Singh[105], Toril Skandsen[127, 128] , Peter Smielewski[26], Abayomi Sorinola[129], Emmanuel Stamatakis[48], Simon Stanworth[39], Robert Stevens[130], William Stewart[131], Ewout W. Steyerberg[65, 132, 133], Nino Stocchetti[134], Nina Sundström[135], Riikka Takala[136], Viktória Tamás[129], Tomas Tamosuitis[137], Mark Steven Taylor[20], Aurore Thibaut[81], Braden Te Ao[53], Olli Tenovuo[107], Alice Theadom[53], Matt Thomas[91], Dick Tibboel[138], Marjolein Timmers[77], Christos Tolias[139], Tony Trapani[28], Cristina Maria Tudora[97], Andreas Unterberg[94], Peter Vajkoczy [140], Shirley Vallance[28], Egils Valeinis[61], Zoltán Vámos[51], Mathieu van der Jagt[141], Gregory Van der Steen[44], Joukje van der Naalt[74], Jeroen T.J.M. van Dijck [105], Inge A. M. van Erp[105], Thomas A. van Essen[105], Wim Van Hecke[142], Caroline van Heugten[143], Ernest van Veen[65], Thijs Vande Vyvere[144], Roel P. J. van Wijk[105], Alessia Vargiolu[32], Emmanuel Vega[86], Kimberley Velt[65], Jan Verheyden[142], Paul M. Vespa[145], Anne Vik[127, 146], Rimantas Vilcinis[137], Victor Volovici[68], Nicole von Steinbüchel[38], Daphne Voormolen[65], Petar Vulekovic[47], Kevin K.W. Wang[147], Daniel Whitehouse[48], Eveline Wiegers[65], Guy Williams[48], Lindsay Wilson[72], Stefan Winzeck[48], Stefan Wolf[148], Zhihui Yang[117], Peter Ylén[149], Alexander Younsi[94], Marina Zeldovich[150], Frederick A. Zeiler[48,151], Veronika Zelinkova[20], Agate Ziverte[61] , Tommaso Zoerle[27]

[1] Department of Physiology and Pharmacology, Section of Perioperative Medicine and Intensive Care, Karolinska Institutet, Stockholm, Sweden
[2] János Szentágothai Research Centre, University of Pécs, Pécs, Hungary
[3] Division of Clinical Neuroscience, Department of Physical Medicine and Rehabilitation, Oslo University Hospital and University of Oslo, Oslo, Norway
[4] Department of Neurosurgery, University Hospital Northern Norway, Tromso, Norway
[5] Department of Physical Medicine and Rehabilitation, University Hospital Northern Norway, Tromso, Norway
[6] Trauma Surgery, Medical University Vienna, Vienna, Austria
[7] Department of Anesthesiology & Intensive Care, University Hospital Nancy, Nancy, France
[8] Raymond Poincare hospital, Assistance Publique – Hopitaux de Paris, Paris, France
[9] Department of Anesthesiology & Intensive Care, S Raffaele University Hospital, Milan, Italy
[10] Department of Neurosurgery, Radboud University Medical Center, Nijmegen, The Netherlands
[11] Department of Neurosurgery, University of Szeged, Szeged, Hungary
[12] International Projects Management, ARTTIC, Munchen, Germany
[13] Department of Neurology, Neurological Intensive Care Unit, Medical University of Innsbruck, Innsbruck, Austria
[14] Department of Neurosurgery & Anesthesia & intensive care medicine, Karolinska University Hospital, Stockholm, Sweden
[15] NIHR Surgical Reconstruction and Microbiology Research Centre, Birmingham, UK
[16] Anesthesie-Réanimation, Assistance Publique – Hopitaux de Paris, Paris, France
[17] Department of Anesthesia & ICU, AOU Città della Salute e della Scienza di Torino - Orthopedic and Trauma Center, Torino, Italy
[18] Department of Neurology, Odense University Hospital, Odense, Denmark
[19] BehaviourWorks Australia, Monash Sustainability Institute, Monash University, Victoria, Australia
[20] Department of Public Health, Faculty of Health Sciences and Social Work, Trnava University, Trnava, Slovakia
[21] Quesgen Systems Inc., Burlingame, California, USA
[22] Australian & New Zealand Intensive Care Research Centre, Department of Epidemiology and Preventive Medicine, School of Public Health and Preventive Medicine, Monash University, Melbourne, Australia
[23] Department of Surgery and Perioperative Science, Umeå University, Umeå, Sweden
[24] Department of Neurosurgery, Örebro University and University Hospital, Örebro, Sweden
[25] Department of Medical Psychology, Universitätsklinikum Hamburg-Eppendorf, Hamburg, Germany
[26] Brain Physics Lab, Division of Neurosurgery, Dept of Clinical Neurosciences, University of Cambridge, Addenbrooke's Hospital, Cambridge, UK
[27] Neuro ICU, Fondazione IRCCS Cà Granda Ospedale Maggiore Policlinico, Milan, Italy
[28] ANZIC Research Centre, Monash University, Department of Epidemiology and Preventive Medicine, Melbourne, Victoria, Australia
[29] Department of Neurosurgery, Hospital of Cruces, Bilbao, Spain
[30] NeuroIntensive Care, Niguarda Hospital, Milan, Italy
[31] School of Medicine and Surgery, Università Milano Bicocca, Milano, Italy
[32] NeuroIntensive Care Unit, Department Neuroscience, IRCCS Fondazione San Gerardo dei Tintori, Monza, Italy
[33]Department of Neurosurgery, Medical Faculty RWTH Aachen University, Aachen, Germany
[34] Department of Anesthesiology and Intensive Care Medicine, University Hospital Bonn, Bonn, Germany
[35] Department of Anesthesia & Neurointensive Care, Cambridge University Hospital NHS Foundation Trust, Cambridge, UK
[36] School of Public Health & PM, Monash University and The Alfred Hospital, Melbourne, Victoria, Australia
[37] Radiology/MRI department, MRC Cognition and Brain Sciences Unit, Cambridge, UK
[38] Institute of Medical Psychology and Medical Sociology, Universitätsmedizin Göttingen, Göttingen, Germany
[39] Oxford University Hospitals NHS Trust, Oxford, UK
[40] Department of Neurosurgery, Medical School, University of Pécs, Hungary and Neurotrauma Research Group, János Szentágothai Research Centre, University of Pécs, Hungary
[41] Intensive Care Unit, CHU Poitiers, Potiers, France
[42] University of Manchester NIHR Biomedical Research Centre, Critical Care Directorate, Salford Royal Hospital NHS Foundation Trust, Salford, UK
[43] Movement Science Group, Faculty of Health and Life Sciences, Oxford Brookes University, Oxford, UK
[44] Department of Neurosurgery, Antwerp University Hospital, Edegem, Belgium
[45] Department of Anesthesia & Intensive Care, Maggiore Della Carità Hospital, Novara, Italy

[46] Department of Neurosurgery, University Hospitals Leuven, Leuven, Belgium
[47] Department of Neurosurgery, Clinical centre of Vojvodina, Faculty of Medicine, University of Novi Sad, Novi Sad, Serbia
[48] Division of Anaesthesia, University of Cambridge, Addenbrooke's Hospital, Cambridge, UK
[49] Center for Stroke Research Berlin, Charité – Universitätsmedizin Berlin, corporate member of Freie Universität Berlin, Humboldt-Universität zu Berlin, and Berlin Institute of Health, Berlin, Germany
[50] Intensive Care Unit, CHR Citadelle, Liège, Belgium
[51] Department of Anaesthesiology and Intensive Therapy, University of Pécs, Pécs, Hungary
[52] Departments of Neurology, Clinical Neurophysiology and Neuroanesthesiology, Region Hovedstaden Rigshospitalet, Copenhagen, Denmark
[53] National Institute for Stroke and Applied Neurosciences, Faculty of Health and Environmental Studies, Auckland University of Technology, Auckland, New Zealand
[54] Department of Neurology, Erasmus MC, Rotterdam, the Netherlands
[55] Department of Anesthesiology and Intensive care, University Hospital Northern Norway, Tromso, Norway
[56] Department of Neurosurgery, Hadassah-hebrew University Medical center, Jerusalem, Israel
[57] Fundación Instituto Valenciano de Neurorrehabilitación (FIVAN), Valencia, Spain
[58] Department of Neurosurgery, Shanghai Renji hospital, Shanghai Jiaotong University/school of medicine, Shanghai, China
[59] Karolinska Institutet, INCF International Neuroinformatics Coordinating Facility, Stockholm, Sweden
[60] Emergency Department, CHU, Liège, Belgium
[61] Neurosurgery clinic, Pauls Stradins Clinical University Hospital, Riga, Latvia
[62] Department of Computing, Imperial College London, London, UK
[63] Department of Neurosurgery, Hospital Universitario 12 de Octubre, Madrid, Spain
[64] Department of Anesthesia, Critical Care and Pain Medicine, Medical University of Vienna, Austria
[65] Department of Public Health, Erasmus Medical Center-University Medical Center, Rotterdam, The Netherlands
[66] College of Health and Medicine, Australian National University, Canberra, Australia
[67] Department of Neurosurgery, Neurosciences Centre & JPN Apex trauma centre, All India Institute of Medical Sciences, New Delhi-110029, India
[68] Department of Neurosurgery, Erasmus MC, Rotterdam, the Netherlands
[69] Department of Neurology, Kepler University Hospital, Johannes Kepler University Linz, Linz, Austria.
[70] Clinical Research Institute for Neuroscience, Johannes Kepler University Linz, Linz, Austria
[71] Department of Neurosurgery, Oslo University Hospital, Oslo, Norway
[72] Division of Psychology, University of Stirling, Stirling, UK
[73] Division of Neurosurgery, Department of Clinical Neurosciences, Addenbrooke's Hospital & University of Cambridge, Cambridge, UK
[74] Department of Neurology, University of Groningen, University Medical Center Groningen, Groningen, Netherlands
[75] Neurointensive Care , Sheffield Teaching Hospitals NHS Foundation Trust, Sheffield, UK
[76] Salford Royal Hospital NHS Foundation Trust Acute Research Delivery Team, Salford, UK
[77] Department of Intensive Care and Department of Ethics and Philosophy of Medicine, Erasmus Medical Center, Rotterdam, The Netherlands
[78] Department of Clinical Neuroscience, Neurosurgery, Umeå University, Umeå, Sweden
[79] Hungarian Brain Research Program - Grant No. KTIA_13_NAP-A-II/8, University of Pécs, Pécs, Hungary
[80] Department of Anaesthesiology, University Hospital of Aachen, Aachen, Germany
[81] Cyclotron Research Center , University of Liège, Liège, Belgium
[82] Centre for Urgent and Emergency Care Research (CURE), Health Services Research Section, School of Health and Related Research (ScHARR), University of Sheffield, Sheffield, UK
[83] Emergency Department, Salford Royal Hospital, Salford UK
[84] Institute of Research in Operative Medicine (IFOM), Witten/Herdecke University, Cologne, Germany
[85] VP Global Project Management CNS, ICON, Paris, France
[86] Department of Anesthesiology-Intensive Care, Lille University Hospital, Lille, France
[87] Department of Neurosurgery, Rambam Medical Center, Haifa, Israel
[88] Department of Anesthesiology & Intensive Care, University Hospitals Southhampton NHS Trust, Southhampton, UK
[89] Department of Translational Neuroscience, Faculty of Medicine and Health Science, University of Antwerp, Antwerp, Belgium

[90] Cologne-Merheim Medical Center (CMMC), Department of Traumatology, Orthopedic Surgery and Sportmedicine, Witten/Herdecke University, Cologne, Germany
[91] Intensive Care Unit, Southmead Hospital, Bristol, Bristol, UK
[92] Department of Neurological Surgery, University of California, San Francisco, California, USA
[93] Department of Anesthesia & Intensive Care,M. Bufalini Hospital, Cesena, Italy
[94] Department of Neurosurgery, University Hospital Heidelberg, Heidelberg, Germany
[95] Department of Neurosurgery, The Walton centre NHS Foundation Trust, Liverpool, UK
[96] Department of Medical Genetics, University of Pécs, Pécs, Hungary
[97] Department of Neurosurgery, Emergency County Hospital Timisoara , Timisoara, Romania
[98] School of Medical Sciences, Örebro University, Örebro, Sweden
[99] Institute for Molecular Medicine Finland, University of Helsinki, Helsinki, Finland
[100] Analytic and Translational Genetics Unit, Department of Medicine; Psychiatric & Neurodevelopmental Genetics Unit, Department of Psychiatry; Department of Neurology, Massachusetts General Hospital, Boston, MA, USA
[101] Program in Medical and Population Genetics; The Stanley Center for Psychiatric Research, The Broad Institute of MIT and Harvard, Cambridge, MA, USA
[102] Department of Radiology, University of Antwerp, Edegem, Belgium
[103] Department of Anesthesiology & Intensive Care, University Hospital of Grenoble, Grenoble, France
[104] Department of Anesthesia & Intensive Care, Azienda Ospedaliera Università di Padova, Padova, Italy
[105] Dept. of Neurosurgery, Leiden University Medical Center, Leiden, The Netherlands and Dept. of Neurosurgery, Medical Center Haaglanden, The Hague, The Netherlands
[106] Department of Neurosurgery, Helsinki University Central Hospital
[107] Division of Clinical Neurosciences, Department of Neurosurgery and Turku Brain Injury Centre, Turku University Hospital and University of Turku, Turku, Finland
[108] Department of Anesthesiology and Critical Care, Pitié -Salpêtrière Teaching Hospital, Assistance Publique, Hôpitaux de Paris and University Pierre et Marie Curie, Paris, France
[109] Neurotraumatology and Neurosurgery Research Unit (UNINN), Vall d'Hebron Research Institute, Barcelona, Spain
[110] Department of Neurosurgery, Kaunas University of technology and Vilnius University, Vilnius, Lithuania
[111] Department of Neurosurgery, Rezekne Hospital, Latvia
[112] Department of Anaesthesia, Critical Care & Pain Medicine NHS Lothian & University of Edinburg, Edinburgh, UK
[113] Director, MRC Biostatistics Unit, Cambridge Institute of Public Health, Cambridge, UK
[114] Department of Physical Medicine and Rehabilitation, Oslo University Hospital/University of Oslo, Oslo, Norway
[115] Division of Orthopedics, Oslo University Hospital, Oslo, Norway
[116] Institue of Clinical Medicine, Faculty of Medicine, University of Oslo, Oslo, Norway
[117] Broad Institute, Cambridge MA Harvard Medical School, Boston MA, Massachusetts General Hospital, Boston MA, USA
[118] National Trauma Research Institute, The Alfred Hospital, Monash University, Melbourne, Victoria, Australia
[119] Department of Neurosurgery, Odense University Hospital, Odense, Denmark
[120] International Neurotrauma Research Organisation, Vienna, Austria
[121] Klinik für Neurochirurgie, Klinikum Ludwigsburg, Ludwigsburg, Germany
[122] Division of Biostatistics and Epidemiology, Department of Preventive Medicine, University of Debrecen, Debrecen, Hungary
[123] Department Health and Prevention, University Greifswald, Greifswald, Germany
[124] Department of Anaesthesiology and Intensive Care, AUVA Trauma Hospital, Salzburg, Austria
[125] Department of Neurology, Elisabeth-TweeSteden Ziekenhuis, Tilburg, the Netherlands
[126] Department of Neuroanesthesia and Neurointensive Care, Odense University Hospital, Odense, Denmark
[127] Department of Neuromedicine and Movement Science, Norwegian University of Science and Technology, NTNU, Trondheim, Norway
[128] Department of Physical Medicine and Rehabilitation, St.Olavs Hospital, Trondheim University Hospital, Trondheim, Norway
[129] Department of Neurosurgery, University of Pécs, Pécs, Hungary
[130] Division of Neuroscience Critical Care, John Hopkins University School of Medicine, Baltimore, USA
[131] Department of Neuropathology, Queen Elizabeth University Hospital and University of Glasgow, Glasgow, UK
[132] Dept. of Department of Biomedical Data Sciences, Leiden University Medical Center, Leiden, The Netherlands

[133] Julius Center for Health Sciences and Primary Care, University Medical Center Utrecht, Utrecht, The Netherlands
[134] Department of Pathophysiology and Transplantation, Milan University, and Neuroscience ICU, Fondazione IRCCS Cà Granda Ospedale Maggiore Policlinico, Milano, Italy
[135] Department of Radiation Sciences, Biomedical Engineering, Umeå University, Umeå, Sweden
[136] Perioperative Services, Intensive Care Medicine and Pain Management, Turku University Hospital and University of Turku, Turku, Finland
[137] Department of Neurosurgery, Kaunas University of Health Sciences, Kaunas, Lithuania
[138] Intensive Care and Department of Pediatric Surgery, Erasmus Medical Center, Sophia Children's Hospital, Rotterdam, The Netherlands
[139] Department of Neurosurgery, Kings college London, London, UK
[140] Neurologie, Neurochirurgie und Psychiatrie, Charité – Universitätsmedizin Berlin, Berlin, Germany
[141] Department of Intensive Care Adults, Erasmus MC– University Medical Center Rotterdam, Rotterdam, the Netherlands
[142] icoMetrix NV, Leuven, Belgium
[143] Movement Science Group, Faculty of Health and Life Sciences, Oxford Brookes University, Oxford, UK
[144] Radiology Department, Antwerp University Hospital and University of Antwerp, Edegem, Belgium
[145] Director of Neurocritical Care, University of California, Los Angeles, USA
[146] Department of Neurosurgery, St.Olavs Hospital, Trondheim University Hospital, Trondheim, Norway
[147] Department of Emergency Medicine, University of Florida, Gainesville, Florida, USA
[148] Department of Neurosurgery, Charité – Universitätsmedizin Berlin, corporate member of Freie Universität Berlin, Humboldt-Universität zu Berlin, and Berlin Institute of Health, Berlin, Germany
[149] VTT Technical Research Centre, Tampere, Finland
[150] Sigmund Freud University, Faculty of Psychotherapy Science, Vienna, Austria
[151] Section of Neurosurgery, Department of Surgery, Rady Faculty of Health Sciences, University of Manitoba, Winnipeg, MB, Canada

The TRACK-TBI investigators

Edilberto Amorim, MD[1], Kim Boase[2], Jason Barber, MS[2], Jeff Bazarian, MD MPH[3], Patrick Belton, MD[4], Yelena Bodien, PhD[5], Gretchen Brophy, PharmD[6], Randall Chesnut, MD[2], John D. Corrigan, PhD, ABPP[7] Ramon Diaz-Arrastia, MD PhD[8], Ann-Christine Duhaime, MD[9], Shawn Eagle, PhD[10], Richard Ellenbogen, MD[2] Adam R. Ferguson, PhD[1], Brandon Foreman, MD[11], Raquel Gardner, MD[12], Etienne Gaudette, PhD[13], Joseph Giacino, PhD[14], Shankar Gopinath, MD[15], Ramesh Grandhi, MD MS[16], J Claude Hemphill, MD[1], Russell Huie, PhD[1], Sonia Jain, PhD[17], Ruchira Jha, MD, MSc[18] C. Dirk Keene, MD PhD[2], Ryan Kitagawa, MD[19], Frederick K. Korley, MD, PhD[20], Natalie Kreitzer, MD[11], Vijay Krishnamoorthy, MD[21], Christine Mac Donald, PhD[2], Joan Machamer, MA[2], Debbie Madhok, MD[1], Geoffrey T Manley, MD PhD[1], Rebekah Mannix, MD MPH[22], Amy J. Markowitz[1], Thomas McAllister, MD[23], Michael McCrea, PhD[24], Randall Merchant, PhD[6], Pratik Mukherjee, MD PhD[1], Lindsay Nelson, PhD[24], Laura B. Ngwenya, MD, PhD[11], Amber Nolan, MD PhD[2], David Okonkwo, MD PhD[10], Ava Puccio, PhD[10], Christopher J. Roberts, MD PhD[24], Claudia Robertson, MD[25], Richard B Rodgers, MD[26], Eric Rosenthal, MD[27], Angelle Sander, PhD[25], Danielle Sandsmark, MD, PhD[8], Andrea Schneider, MD, PhD[8], David Schnyer, PhD[28], Seth Seabury, PhD[29], Jane Sharpless[10], Murray Stein, MD MPH[17], Gabriella Sugar, RN[1], Xiaoying 'Shelly' Sun, MS[17], Sabrina R. Taylor, PhD[1], Nancy Temkin, PhD[2], Abel Torres-Espin, PhD[30], Alex Valadka, MD[31], Mary Vassar, RN MS[1], Kevin Wang, PhD[32], Vincent Wang, MD PhD[33], John K. Yue, MD[1], Esther Yuh, MD PhD[1], Ross Zafonte, DO[34]

[1] University of California, San Francisco
[2] University of Washington
[3] University of Rochester
[4] University of Wisconsin-Madison
[5] Vanderbilt University
[6] Virginia Commonwealth University

[7] Ohio State University
[8] University of Pennsylvania
[9] MassGeneral Hospital for Children
[10] University of Pittsburgh
[11] University of Cincinnati
[12] Sheba Medical Center, Ramat Gan, Israel
[13] University of Toronto
[14] Spaulding Rehabilitation Hospital
[15] Baylor College of Medicine
[16] University of Utah
[17] University of California, San Diego
[18] Barrow Neurological Institute
[19] University of Texas Houston
[20] University of Michigan
[21] Duke University
[22] Boston Children's Hospital
[23] Indiana University
[24] Medical College of Wisconsin
[25] Baylor College of Medicine
[26] Goodman Campbell Brain and Spine
[27] Massachusetts General Hospital
[28] University of Rhode Island
[29] University of Southern California
[30] University of Waterloo
[31] UT Southwestern
[32] Morehouse School of Medicine
[33] UT Austin
[34] University of Missouri
