# Supplementary material for: The CBI-M framework for characterization of traumatic brain injury: Evaluation of performance using data from the CENTER-TBI and TRACK-TBI prospective cohort studies
Source: PLoS Med. 2026 Oct 20;23(10):e1004945. doi: 10.1371/journal.pmed.1004945 (PMC13681731; doi:10.1371/journal.pmed.1004945)
Supplement: Supplementary file 16 [file pmed.1004945.s016.pdf]

Study Protocol
Collaborative European NeuroTrauma Effectiveness research in TBI: a prospective longitudinal observational study

Short study title:	CENTER-TBI study
Protocol:	version 4.1
Version date:	03 July 2014
Coordinators:	Andrew IR Maas, Antwerp University Hospital<br>David K Menon, University of Cambridge
Funded:	by the European Union FP 7th Framework program (grant 602150)

Contract Research Organization: ICON plc, (Dublin, Ireland)

Data entry tool developed by: QuesGen (Burlington, CA, USA)

Neuroimaging repository: Icometrix, Antwerp, Belgium

Database and data analysis platform: Coordinated by the International Neuroinformatics Coordinating Facility (INCF) with additional support from One Mind for Research

Study synopsis

**Full study title**	Collaborative European NeuroTrauma Effectiveness research in TBI: a prospective longitudinal observational study
**Short study title**	CENTER-TBI study
**Study Design**	Longitudinal prospective observational cohort
**Study centers**	Approximately 80 centers from 21 countries in Europe and Israel
**Study population**	Patients with traumatic brain injury
**Funding source and international context**	Funded by the European Union FP7 framework program (grant 602150) with additional national and local institutional support.<br>CENTER-TBI is an InTBIR project (International Initiative for Traumatic Brain Injury Research: http://intbir.nih.gov/)<br>Funding of additional elements has been provided by the Hannelore Kohl Foundation (Germany) and by the non-profit organization One Mind For Research (directly to INCF).

Study objectives

The global aims of the study are to:

- Improve characterization and classification of TBI in Europe, with inclusion of emerging technologies.
- To identify the most effective clinical care and to provide high quality evidence in support of treatment recommendations and guidelines.

Secondary objectives:

- To collect high quality clinical and epidemiological data with repositories for neuro-imaging, DNA, and serum from patients with TBI.
- To refine and improve outcome assessment and develop health utility indices for TBI.
- To develop multidimensional approaches to characterisation and prediction of TBI.
- To define patient profiles which characterise homogenous subgroups of patients and predict efficacy of specific interventions ("Precision Medicine").
- To develop performance indicators for quality assurance and quality improvement in TBI care.
- To validate the common data elements (CDEs) for broader use in international settings.
- To develop an open source database compatible with FITBIR.
- To intensify networking activities and international collaborations in TBI.
- To disseminate study results and management recommendations for TBI to health care professionals, policy makers and consumers, aiming to improve health care for TBI at individual and population levels.
- To develop a "knowledge commons" for TBI, integrating CENTER-TBI outputs with systematic reviews

Study design

**Methodology**	This study will be a multicentre longitudinal prospective observational, cohort study conducted in 21 countries across Europe and Israel.<br>Data collection for CENTER-TBI will take place at 2 levels: CENTER-TBI registry (all patients) and CENTER-TBI Core data collection (patients meeting inclusion and exclusion criteria; detailed data collection).
**Number of subjects**	**CENTER-TBI core data study**: the planned total number of subjects will be 5400 equally distributed across three strata:<br>- *ER stratum*: patients seen and discharged from the ER<br>- *Admission stratum*: patients admitted to hospital but not to the ICU<br>- *ICU stratum*: patients admitted directly to the ICU<br><br>**CENTER-TBI registry**: 15.000-25,000
**Inclusion criteria for observational study**	1. Clinical diagnosis of TBI<br>2. Clinical indication for CT scan
**Inclusion criteria for core data study**<br><br>**Exclusion criteria for core data study**	1. Presentation within 24 hours of injury<br>2. Informed consent obtained according to local and national requirements<br><br>Severe pre-existing neurological disorder that would confound outcome assessments
**Extended studies**	Selected sites, meeting additional logistic requirements, will participate in extended data collection with regard to:<br><br>- MR imaging<br>- Extended coagulation and biomarker studies<br>- Acquisition of high resolution ICU monitoring data
**Study Interventions**	
**Therapeutic interventions**	None
**Diagnostic interventions**	- Registry: none<br>- Core study: blood sampling on presentation, in the ICU stratum during the clinical course and in all strata at follow up<br>- Follow up assessments
**Data collection**	
**Registry**	Only observational data collected, without formal consent, in the context of routine clinical care
**Core study**	Prospective collection of clinical data with informed consent.<br>Blood collection:<br>- Hematology<br>- Biochemistry<br>- Genotyping<br>- Biomarker analysis<br>- Coagulation studies<br>CT scan as part of routine clinical care<br>Outcome assessments

Study endpoints

Registry: Vital status, injury severity indices, and discharge destination

Core data collection:
Cross sectional comprehensive outcome assessments across the three strata of recruitments in all subjects at 6 months; these assessments will include health related quality of life, psychological and neuropsychological testing (CANTAB).

Longitudinal assessments of outcome by telephone,postal questionnaire and/or face-to-face visits will be performed at various time points in the three strata up to 24 months after injury (focus on more early outcome assessments in ER stratum and later assessments in admission in ICU strata).

Analysis directions in the context of the CENTER-TBI project

The registry and core data study will form the basis of the scientific analysis described in the Description of Work of the CENTER-TBI project (funded by the European Union FP 7 program grant 602150).
Analysis of the CENTER-TBI core data and CENTER-TBI registry will be in two directions:
1. Improved characterization of disease
2. Identification of best practice, addressing both variations in process and in clinical care, and including the use of stratified/personalized approaches.
Results of the analyses will be integrated with results of ongoing living systematic reviews in a process of knowledge transfer aiming to provide practical evidence based recommendations.

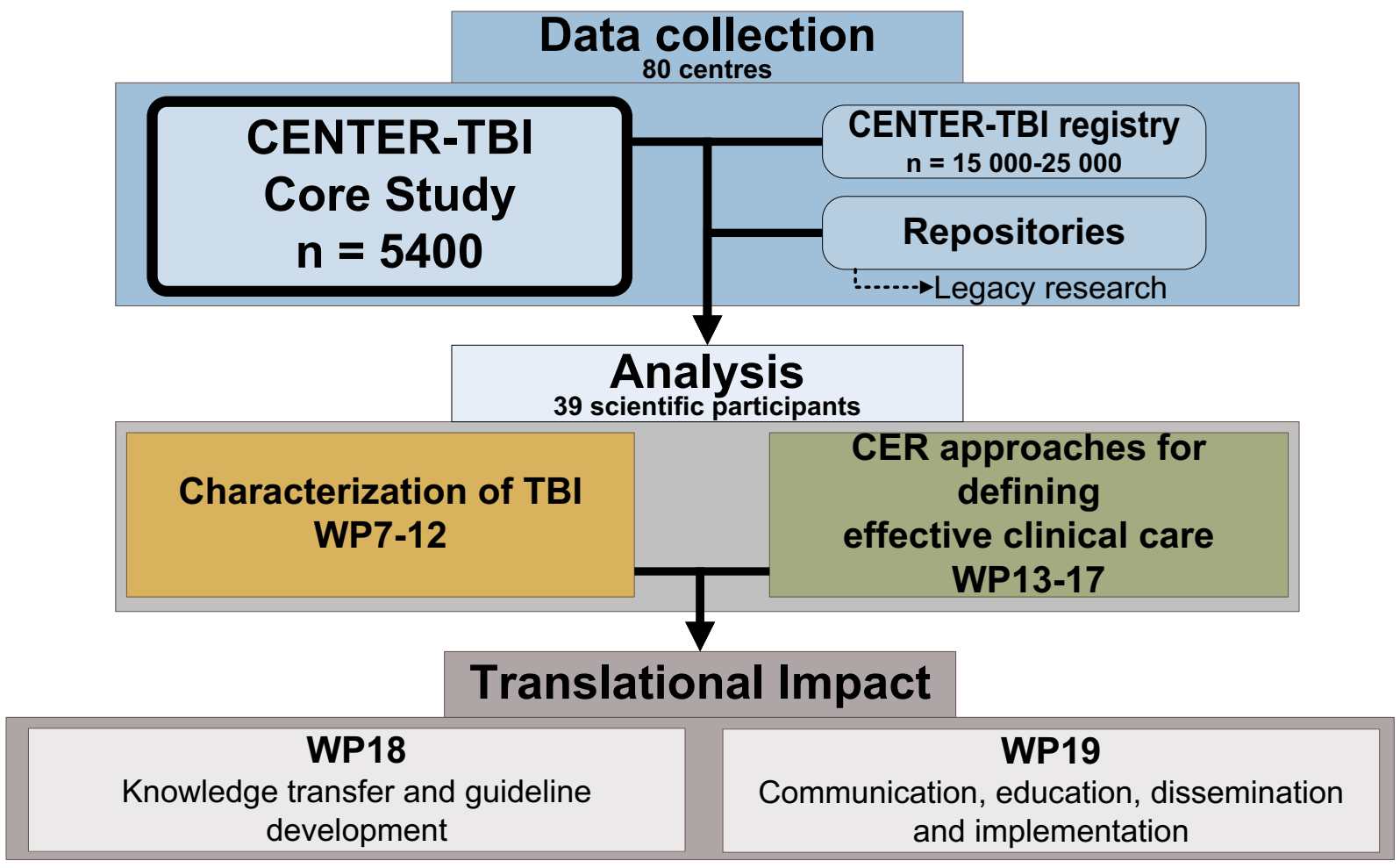

Sample size justification
A total sample size of 5400 patients is planned for the Core Data Study with enrolment of approximately 1800 per stratum.<br><br>This sample size estimate was based on:<br>• Practical logistic considerations<br>• Power calculations for the different strata, targeting comparative effectiveness analyses, assuming a between-centre and between-countries differences as previously observed in TBI research<br>• Postulated odds ratios for intervention effects of approximately 5% improvement in outcome.<br><br>Overall, these calculations provided a statistical power to detect odds ratios of ~1.2 associated with differences in process or intervention variables across the core dataset with a power of 80%; and somewhat larger odds ratios in each of the 3 individual strata.<br>In the registry we expect to be able to detect differences (predominantly in organizational or system variables) with an odds ratio of 1.2 with a power of 82%.
**Statistical analysis plan**
Statistical analyses for the Comparative Effectiveness Research (CER) questions will primarily apply random effects modeling, in which center is included at the highest level, and patients are considered clusters within centers. Confounding factors as measured at the individual patient and/or center level, will be considered.<br>Statistical analyses for better characterization of TBI will be mainly exploratory. Standard statistical descriptive and inferential techniques, as well as machine learning techniques will be used. Prognostic analyses will consider a range of variables, including genetic, biomarker, neuro-imaging and additional outcome assessments. Previously and newly developed prediction models will be validated by measures for model fit, discrimination, and calibration.

Overall goals and impact of CENTER-TBI

The CENTER-TBI project will contribute towards the overall goals of InTBIR, by identifying more effective and efficient treatment provision, thus improving outcome and reducing costs. The science in the project will provide methodological advances (including novel, multilingual translations of key outcome instruments), novel information on disease processes, treatment, outcome, and prognosis in TBI, identifying new therapeutic targets and therapies; while the CENTER-TBI repositories will ensure opportunities for legacy research. Thus, the project has the potential to improve current health care and its delivery at both population and individual levels, deliver early scientific advances that could improve the care of patients with TBI, and provide a rich investment for future biomedical and clinical research.

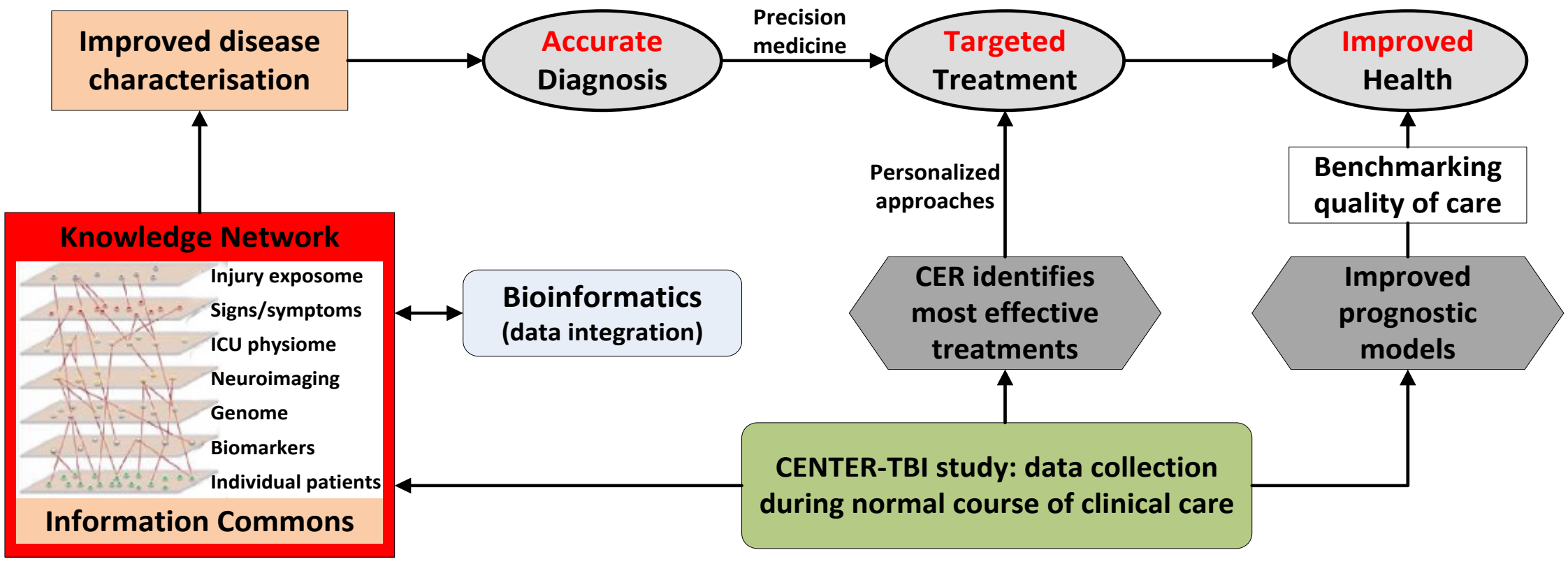

Figure 3.2: The CENTER-TBI participants represent a knowledge network that will analyze the clinical database and associated biorepositories. This highly granular "information commons", aided by novel bioinformatic approaches, will improve disease characterization, resulting in a new taxonomy of TBI (Precision Medicine). Clinical data will be subjected to CER analysis, both directly, and after refinement with precision medicine to identify more effective and targeted therapies. The increased data inputs will improve prognostic accuracy, allowing better benchmarking of care (Adapted from National Research Council).

Table of content

Schedule of events 9
Abbreviations 12
CENTER-TBI Management Committee 13
CENTER-TBI Scientific Advisory Board 14
CENTER-TBI Partner Organisations 15
1. Introduction and rationale 16
2. Concept of CENTER-TBI 17
3. Objectives 18
4. Study design 18
4.1 Statement of study design 18
4.2 Subject groups 18
4.3 Study sites 19
4.4 Number of subjects 19
4.5 Sample size calculation and planned statistical analyses 20
4.6 Study Duration 20
4.7 Inclusion – exclusion criteria 21
5. Subject procedures 21
5.1 CENTER-TBI registry 21
5.2 CENTER-TBI core study 21
5.3 Clinical: 21
5.4. Laboratory assays 22
6. Genetic studies 23
7. Neuro-imaging 23
8. Outcomes 24
9. Extended studies 26
9.1 MR imaging 26
9.2 Extended hemostatis studies 27
9.3 High resolution ICU monitoring 30
9.4 Electrocorticographic (EcoG) monitoring 30
9.5 Continuous EEG monitoring 31
10. Informed consent 32
10.1 Informed Consent Personnel 32
10.2 Location and Privacy 32
10.3 Consent 32

10. 4 Need for reconsent ... 33
10.5 Withdrawal from study ... 33
10.6 Declaration of Helsinki ... 33
10.7 ICH Guidelines for Good Clinical Practice ... 33
10.8 Data handling and record keeping ... 33
11. Subject risks and benefits ... 34
11.1 Foreseeable risks and their minimization ... 34
11.2 Potential benefits of proposed research ... 36
11.3 Benefits and impact of the CENTER-TBI project to patients, health care professionals and policy makers. ... 36
11.4 Adverse events ... 37
12. Data Management and Compliance ... 38
13. Clinical protocol maintenance ... 40
13.1 Protocol modifications ... 40
13.2 Protocol deviations ... 40
14. Publication ... 40
15. Insurance and liability ... 41
References ... 41

Schedule of events

Schedule of Assessments - ER Stratum

Visit Name	Acute	≤ 72 hours from injury	2-3 weeks	3 month	6 month
**Consent Subject/Consultee/Brief**	x[a]				
**Re-Consent**		x[b]	x[b]	x[b]	x[b]
**Emergency Room eCRF**	x				
**Post Discharge eCRF**			x	X	x
**Biomarker**	X (1800)		X (600)	X (200)	
**Genetics**	X (1800)				
**Routine Local Haemostasis**	X[f] (1800)				
**Extended Local Haemostasis**	X[g] (600)				
**Central Haemostasis**					
**Ultra Early MRI**		X[c] (200)			
**MRI**			X (600)	X[e] (250)	
**Neuropsychology Follow-Up**			X[d] (600)	X (600)	X (1150)
Questionnaire Follow-Up			X (1400)	X (1300)	X (1250)

a	Consent procedure will depend on capacity assessment
b	Only if required
c	Only in sites participating in UE MR arm
d	Only in patients undergoing MR studies
e	Target patients with abnormalities on previous MR<br>(patients who have a normal MR at 2-3 weeks will not be invited for repeat imaging)
f	Record routine clinical bloods if obtained in the eCRF
g	Only in sites participating in extended haemostatis arm

* Figures in brackets represent the number of subjects targeted for the procedure in that stratum (this accounts for funding constraints, mortality, loss to follow-up and feasibility)

Schedule of Assessments – Admission Stratum

Visit Name	Acute	Day 1-7	Day 10	Day 14	Day 21	Day 28	2-3 weeks	3 Month	6 month	12 month	24 month
**Consent Subject/Consultee/Brief**	x[a]										
**Re-Consent**		x[b]	x[b]	x[b]	x[b]	x[b]	x[b]	x[b]	x[b]	x[b]	x[b]
**Admission eCRF**	x	x[c]	x[c]	x[c]	x[c]	x[c]					
**Post Discharge eCRF**							x	x	x	x	x
**Biomarker**	X (1800)								X (1200)	X (300)	X (250)
**Genetics**	X (1800)										
**Routine Local Haemostasis**	X[i] (1800)										
**Extended Local Haemostasis**	X[j] (600)										
**Central Haemostasis**	X[j] (600)										
**Ultra Early MRI**	X[h] (200)										
**MRI**							X[e] (600)		X[f] (600)	X[g] (300)	X[g] (150)
**Neuropsychology Follow-Up**									X (1200)	X[d] (300)	X[d] (250)
**Questionnaire Follow-Up**								X (1450)	X (1300)	X[k] (1200)	X (250)

**a**	Consent procedure will depend on capacity assessment
**b**	Only if required
**c**	Only if an in-patient
**d**	Only in patients undergoing MR studies
**e**	Attempt to target patients who had UE MR at <72 hours
**f**	Attempt to target patients who had MR imaging at 2-3 weeks
**g**	Target patient who had MR imaging at 6 months
**h**	Only in site participating in UE MR arm
**i**	Record routine clinical bloods if obtained in the eCRF
**j**	Only in site participating in extended haemostatis studies
**k**	These patients will not have MR imaging

Schedule of Assessments – ICU Stratum

Visit Name	Acute	Post op	Day 1	Day 3	Day 4	Day 5	Day 6-7	Day 10	Day 14	Day 21	Day 28	2-3 wks	3 mth	6 mth	12 mth	24 mth
**Consent Subject/Consultee/Brief**	x[a]															
**Re-Consent**			x[b]	x[b]	x[b]	x[b]	x[b]	x[b]	x[b]	x[b]	x[b]	x[b]	x[b]	x[b]	x[b]	x[b]
**ICU eCRF**	x		x[c]	x[c]	x[c]	x[c]	x[c]	x[c]	x[c]	x[c]	x[c]					
**Post Discharge eCRF**												x	x	x	x	x
**Biomarker**	X (1800)		X (1000)	X (250)	X (250)	X (250)						X (600)		X (1200)	X (300)	X (250)
**Genetics**	X (1800)															
**Routine Local Haemostasis**	X (1800)	X (300)														
**Extended Local Haemostasis**	X (600)	X (300)	X (600)													
**Central Haemostasis**	X (600)	X (300)	X (600)													
**Ultra Early MRI**	X[h] (200)															
**MRI**												X[e] (600)		X[f] (600)	X[g] (300)	X[g] (150)
**HR-ICU**	x[j]															
**EcoG**	x[j]															
**EEG**	x[j]															
**Neuropsychology Follow-Up**														X (1200)	X[d] (300)	X[d] (250)
**Questionnaire Follow-Up**													X (1450)	X (1300)	X[k] (1200)	X (250)

**a**	Consent procedure will depend on capacity assessment
**b**	Only if required
**c**	Only if an in-patient
**d**	Only in patients undergoing MR studies
**e**	Attempt to target patients who had UE MR at <72 hours
**f**	Attempt to target patients who had MR imaging at 2-3 weeks
**g**	Target patient who had MR imaging at 6 months
**h**	Only in site participating in UE MR arm
**i**	Only in site participating in extended haemostatis studies
**j**	Only in centres participating in these extended studies
**k**	These patients will not have MR imaging

Abbreviations

ADNI	Alzheimer's Disease Neuroimaging Initiative
AIS	Abbreviated Injury Scale
BSI-18	Brief Symptom Inventory 18
CANTAB	Cambridge Neuropsychological Test Automated Battery
CDE	Common Data Elements
CENTER-TBI	Collaborative European NeuroTrauma Effectiveness Research in TBI
CER	Comparative Effectiveness Research
CRF	Case report Form
CT	Computed Tomography
EEG	Electroencephalography
ECoG	Electrocorticography
ER	Emergency Room
FITBIR	Federal Interagency Traumatic Brain Injury Research
GCS	Glasgow Coma Scale
GOSE	Glasgow Outcome Scale Extended
GUPI	Global unique Personal Identifier
ICU	Intensive Care Unit
InTBIR	International Initiative for Traumatic Brain Injury Research
ISS	Injury Severity Score
MR(I)	Magnetic Resonance Imaging
PCL-5	PTSD CheckList
PTSD	Post-Traumatic Stress Disorder
Qolibri	Quality of Life after Brain Injury
Qolibri-OS	Quality of Life after Brain Injury – Overall Scale
RAVLT	Rey Auditory Verbal Learning Test
RCT	Randomized Controlled Trial
ROTEM	Rotational thromboelastometry
RPQ	Rivermead Post Concussion Symptoms Questionnaire
SF 36	Short Form (36) Health Survey
TBI	Traumatic Brain Injury
TEG	Thromboelastography

CENTER-TBI Management Committee

Name	Title	Institution/Organisation
Professor Andrew IR Maas	Professor and Chairman, Department of Neurosurgery	University Hospital Antwerp
Professor David K Menon	Professor and Head, Department of Anaesthesia	University of Cambridge
Professor Ewout Steyerberg	Professor of Medical Decision Making	Erasmus MC, University Medical Centre Rotterdam
Professor Fiona Lecky	Clinical Professor of Emergency Medicine	The University of Sheffield
Dr Giuseppe Citerio	Director of Neuroanaesthesia and Neuro Intensive Care Unit	San Gerardo Hospital, Monza, Italy
Professor Sean K Hill	Scientific Director of the INCF	Karolinska Institutet, Sweden
Professor Geoffrey T Manley	Professor and Vice Chairman of Neurological Surgery	University of California, San Francisco
Dr Valerie Legrand	Vice President	ICON plc.
Annina Sorgner	Process Manager	GABO:mi

CENTER-TBI Scientific Advisory Board

Name	Title	Institution/Organisation
Professor David Hovda	Professor and Vice Chairman Research Affairs<br>Director, UCLA Brain Injury Research Centre	University of California, Los Angeles
Professor Gordon Murray	Professor of Medical Statistics	University of Edinburgh
Professor Dik Habbema	Professor of Medical Decision Sciences	Erasmus MC, University Medical Centre Rotterdam
Professor Jan Schwab	Physician and Group Leader, Department of Neurology and Experimental Neurology	Charité – Universitätsmedizin Berlin
Dr Joseph L Hellerstein	Senior Data Science Fellow and Affiliate Professor	University of Washington, Seattle
Dr Mary Baker MBE	Immediate Past President	European Brain Council
Patrick B Donohue	Founder and Chairman	The Sarah Jane Brain Project
Professor Michael J Bell	Professor of Critical Care Medicine and Neurological Surgery<br>Director, Paediatric Neurocritical Care and Neurotrauma Centre	University of Pittsburgh
Dr Christiane Druml	Vice Rector for Clinical Affairs and Executive Director of the Ethics Committee	Medical University of Vienna
Dr Marie-Noëlle Castel	Head of R&D Alliance Management Department	Sanofi-Aventis, Europe

CENTER-TBI Partner Organisations

Organisation Name	Organisation Details	CENTER-TBI Task
ICON PLC,<br>(Dublin, Ireland)	Contract Research Organisation	Support organisation, logistics, delivery and audit of study
QuesGen Systems, INC.<br>(Burlington, CA, USA)	Web-based data management systems provider	Develop data entry tool and global Unique ID (GUPI) system

1. Introduction and rationale

The **CENTER-TBI core data study** and **CENTER-TBI registry** form part of the **CENTER-TBI project**: *Collaborative European NeuroTrauma Effectiveness Research in TBI*, a large scale project funded by the European Union Framework 7 program (grant 602150). The research aims are to: 1) better characterize Traumatic Brain Injury (TBI) as a disease and describe it in a European context and 2) identify the most effective clinical interventions for managing TBI.

Each year approximately 2.5 million people will suffer from some form of TBI in Europe; of these 1 million will be admitted to hospital and 75.000 will die. TBI thus constitutes a major cause of death and disability, leading to great personal suffering to victims and relatives and huge direct and indirect costs to society. In the US, the annual burden of TBI has been estimated at over 60 billion USD in patients with severe TBI. The life time cots per case is estimated at 396.000 USD with disability and lost productivity costs outweighing medical and rehabilitation costs by a factor 4.

TBI is considered "*the most complex disease in our most complex organ*". It is characterized by great heterogeneity in terms of etiology, mechanisms, pathology, severity, and treatment, with widely varying outcomes. Falls and high velocity road traffic incidents cause different types of injury. TBI may consist of diffuse damage, contusional brain damage, or intracranial hematomas. Some structural abnormalities (particularly traumatic axonal injury) may be poorly detected by conventional imaging. The clinical severity of TBI ranges from minor (minimal complaints, no visible structural damage) to virtually unsurvivable injuries. We have found large differences in outcome between centres with up to a six fold higher risk in "poorer" vs. "better" centres after adjustment for chance effects and case mix (Lingsma et al 2011). We now also recognize that TBI is not just an acute event, but can trigger a chronic process, with progressive injury over hours, days, weeks, months, and even years (Masel & DeWitt 2010). Whilst basic research has increased our knowledge of the mechanisms involved, improvements in clinical management have not kept pace. Guidelines for the treatment of TBI are available (www.tbiguidelines.org; www.nice.org.uk) but the evidence underpinning these recommendations is weak. Moreover, current approaches to the characterization of disease severity and outcome are uni-dimensional and have not undergone refinement for more than three decades. Treatment generally follows a "one size fits all" approach and is not targeted to the needs of an individual. Clinical research in TBI is particularly challenging due to disease heterogeneity, and has been further hampered by dispersion of efforts with little collaboration between researchers in acute and post-acute settings, and by research that focuses on isolated disease mechanisms and tests highly specific neuroprotective agents in underpowered clinical trials. RCTs generally employ strict enrolment criteria in order to study the investigational intervention in the "cleanest" setting. The downside of this approach is that results are only valid in such selected subpopulations and that generalizability to the real world context is limited. Indeed, improvements in TBI care have come not from clinical trials, but rather from observational studies, expert guideline development and meta-analysis of individual patient data (Maas et al 2012). However, the large scale international observational studies on TBI in Europe and the USA that underpin these improvements date back at least 20 years (Rosenfeld et al 2012) and do not reflect current clinical care.

Recent advances in genomics, advanced neuro-imaging, and biomarker development provide unparalleled opportunities for refinements in clinical characterization, offering more accurate disease phenotyping. Improved disease characterization will aid *Precision Medicine*, a concept recently enunciated by the US National Academy of Science (National Research Council 2011). Such improved characterization and stratification will allow for more targeted therapies. Further, *Comparative effectiveness research* (CER) provides a promising framework to identify best practices and improve outcome after TBI. CER is the **generation** and **synthesis** of evidence that compares the benefits and harms of alternative methods to prevent, diagnose, treat, and monitor a clinical condition or to

improve the delivery of care. The purpose of CER is to assist consumers, clinicians, purchasers, and policy makers to make informed decisions that will improve health care at both the individual and population levels (Institute of Medicine 2009).

A basic concept of CER is to study differences in care and outcome in observational studies, thus turning natural variability into an asset. In CENTER-TBI, we will exploit the existing heterogeneity in structure, process and outcome to compare treatments and interventions that are standard practice in some centres and countries but not in others. Natural links exist between CER and individualized approaches, since CER aims to identify the best treatment for the individual patient, with a specific type of injury, severity, co-morbidities and other aspects that determine optimal treatment. We see a great potential for CER in TBI because of various unique features: First, there are large between-centre and between-country differences in both outcome and management. Second, robust risk adjustment models have been developed specifically for TBI, providing the possibility to adjust for patient characteristics that affect outcome. Third, advanced statistical models, including random effect models, are available to analyze differences between centres.

2. Concept of CENTER-TBI

The basic concept of this project is to **exploit the existing heterogeneity** in biology, care and outcome of TBI patients to discover underlying pathophysiology, to refine characterisation, and to identify effective clinical interventions. The **key driver** of our research plan is to collect data from a large number of European centres and sufficiently large cohort to enable CER analyses of differences in clinical care and management pathways in TBI. We will conduct a **prospective longitudinal non-randomized observational study**, recruiting a large core cohort of 5400 patients across the severity spectrum in TBI at ~80 sites from 21 countries over 18 months (**the CENTER-TBI Core Study**). We will follow their progress through the disease course with detailed data collection up to 2 years post-injury for the most severely injured patients, thus bridging the acute and post-acute phases. The CENTER-TBI population will be a unique and well characterized resource, accessible for longer term follow up with continued funding.

We will characterize centres with regard to their structural profile in order to explore effects of organizational aspects. Data collection in this large multinational study will be based upon the **common data elements (CDEs),** thus providing evidence context for further refinement and updating of the CDEs in an international setting, which will inform **global standardization of data collection in TBI**. The database structure will be compatible with FITBIR (Federal Interagency Traumatic Brain Injury Research). We will create and maintain **well curated biorepositories** for analysis by the participants and to provide for legacy research with future new methodologies or longer follow up of outcome (supported by future grant funding). The core cohort will be underpinned by comparison with a larger registry (**the CENTER-TBI registry**: n=15,000-25,000) based on pragmatic data collection of all patients with TBI seen in participating centres (to establish the internal generalizability of our study), and by comparison with national trauma registries (to establish the external generalizability of our findings).
The integrated results of the project will be brought together in a process of translational outputs. We aim for **real world approaches** to translating research outputs into practical information for patients, healthcare professionals and policy makers. We will develop and sustain an **international TBI knowledge community** that integrates results of the project with high quality 'living evidence reviews' of the current state of knowledge, aiming to continuously provide **evidence to underpin guidelines and treatment recommendations**.

The impact of CENTER-TBI will be enhanced by international collaborations within and beyond InTBIR. **TBI is a global problem and requires a global approach.** The CENTER-TBI database and repositories will be an invaluable resource for further research which we wish to encourage.

Data sharing policies, providing *open access*, modelled on the Alzheimer's Disease Neuro-imaging Initiative (ADNI) concept, will aim to broaden access to the data, encourage academic productivity, and accelerate outputs. CENTER-TBI participants and investigators will have equal access rights to the data.

3. Objectives

Our global aims are

- To improve characterization and classification of TBI in Europe, with inclusion of emerging technologies.
- To identify the most effective clinical care and to provide high quality evidence in support of treatment recommendations and guidelines.

Specific aims

1. To collect high quality clinical and epidemiological data with repositories for neuro-imaging, DNA, and serum from patients with TBI.
2. To refine and improve outcome assessment and develop health utility indices for TBI.
3. To develop multidimensional approaches to characterisation and prediction of TBI.
4. To define patient profiles which predict efficacy of specific interventions ("Precision Medicine").
5. To develop performance indicators for quality assurance and quality improvement in TBI care.
6. To validate the common data elements (CDEs) for broader use in international settings.
7. To develop an open source database compatible with FITBIR.
8. To intensify networking activities and international collaborations in TBI.
9. To disseminate study results and management recommendations for TBI to health care professionals, policy makers and consumers, aiming to improve health care for TBI at individual and population levels.
10. To develop a "knowledge commons" for TBI, integrating CENTER-TBI outputs into systematic reviews.

4. Study design

4.1 Statement of study design

Longitudinal prospective observational cohort study.

4.2 Subject groups

The study will consist of 2 parts: **CENTER-TBI core data study** (n=5400) and **CENTER-TBI registry** (n=15,000-25,000).

The **CENTER-TBI core data collection** will be stratified upon enrolment into 3 clinical groups differentiated by clinical care path:

- *ER stratum*: patients evaluated in the ER and discharged (n=1800)

- *Admission stratum*: patients admitted to the hospital but not to ICU (n=1800)
- *ICU stratum*: patients admitted directly from ER or other hospital to the ICU (n=1800)

We aim for an equal balance in numbers between the strata: approximately 1800 patients per stratum.

THE **CENTER-TBI registry** will serve 2 important purposes: 1) assessing representativeness of the **CENTER-TBI core study** and 2) providing opportunities for comparative effectiveness analysis of organization of care. Elementary data from all patients excluded from the core data collection for whatever reason, but who do have a clinical diagnosis of TBI and undergo CT scanning will be recorded in the registry.

4.3 Study sites

Approximately 80 centres across 21 countries from Europe and Israel will participate in CENTER-TBI. With a large number of centres participating, it is to be expected that some changes may occur over the course of the project. Updated information will be provided on the CENTER-TBI website: www.center-tbi.eu

Country	Number of sites
**Austria**	3
**Belgium**	6
**Bosnia**	2
**Bulgaria**	2
**Germany**	6
**Denmark**	2
**Finland**	1
**France**	7
**Hungary**	3
**Israel**	2
**Italy**	10
**Lithuania**	3
**Moldavia**	1
**Netherlands**	5
**Norway**	3
**Romania**	3
**Serbia**	1
**Slovakia**	1
**Spain**	3
**Sweden**	2
**UK**	13

4.4 Number of subjects

CENTER-TBI core study:
5400 patients differentiated into 3 equal strata of approximately 1800. Balance in numbers between the strata will be aimed for, but sites will be allowed to arrange recruitment strategies to best suit their local requirements. We would anticipate a far larger number of eventual subjects in the ER and Admission strata than in the ICU stratum. Options for achieving balance would be to limit the

recruitment in the ER and admission strata to certain days per week or certain periods of time. It would be essential to maintain balance of recruitment across the days of the week.

A maximum cap of enrolment will be implemented per centre in order to prevent overrepresentation. The cap is currently fixed at a maximum of 100 patients per stratum with a total number per centre of no more than 250.

CENTER-TBI registry:
No target recruitment number has been set for the CENTER-TBI registry. We anticipate inclusion of approximately 15.000 to 25.000 subjects.

4.5 Sample size calculation and planned statistical analyses

The sample size estimate was based on:

- Practical logistic considerations
- Power calculations for the different strata, targeting comparative effectiveness analyses, assuming a between-centre and between-country heterogeneity as identified in previous research (expressed by variance parameter from a random effects model, Tau^2 of 0.431)
- Postulated odds ratios for intervention effects of approximately 5% improvement in outcome.

Overall, these calculations provided a statistical power to detect odds ratios of ~1.2 associated with differences in process or intervention variables across the core dataset with a power of 80%; and require somewhat larger odds ratios in each of the three individual strata.
In the registry we expect to be able to detect differences (predominantly in organizational or system variables) with an odds ratio of 1.2 with a power of 82%.

The planned analyses are described in detail in the description of work for CENTER-TBI as approved by the European Commission.
Statistical analyses for the Comparative Effectiveness Research (CER) questions will primarily apply random effects modelling, in which center is included at the higher level, and patients are considered clustered within centers. In some analyses, higher levels of clustering will also be considered, e.g. country, or European region; or lower levels, e.g. physicians within hospitals. Confounding factors as measured at the individual patient and/or center level, will be considered extensively, and will be targeted to the specific research question.
Statistical analyses for better characterization of TBI will be exploratory, aiming to better understand the complexity of the disease and to discover new associations. In addition to standard statistical descriptive and inferential techniques, we will also employ novel machine learning techniques as appropriate.
Prognostic analyses will consider a range of variables, including genetic, demographic and clinical data, physiological signals, imaging results, and biomarkers as predictors of early endpoints and physiologic derangement (e.g. raised ICP), and late outcome, including mortality, functional outcome, quality of life and neuropsychological performance. Previously and newly developed prediction models will be validated by comparison of observed to predicted outcome risks, with predictive performance summarized by measures for model fit, discrimination, and calibration.

4.6 Study Duration

Recruitment for CENTER-TBI is planned over an 18 months period with a maximum follow-up duration of 2 years. Recruitment will end when the target numbers are reached. In the event of slower recruitment, the recruitment period may be extended.

4.7 Inclusion – exclusion criteria

CENTER-TBI core study:

Main criteria for inclusion:

1. Clinical diagnosis of TBI
2. Clinical indication for CT scan
3. Presentation within 24 hours of injury
4. Informed consent obtained according to local and national requirements

Exclusion criteria:

Severe pre-existing neurological disorder that would confound outcome assessments

CENTER-TBI registry:

Main criteria for inclusion:

1. Clinical diagnosis of TBI
2. Clinical indication for CT scan

No other inclusion or exclusion criteria apply for the Registry dataset.

5. Subject procedures

5.1 CENTER-TBI registry:

Data collection will be elementary and based on retrospective extraction form clinical record of data which are routinely clinically collection. No specific study interventions will be performed.

5.2 CENTER-TBI core study:

Recruitment

- *ER stratum*: patients seen and discharged from the ER
- *Admission stratum*: patients admitted to hospital but not to the ICU
- *ICU stratum*: patients admitted directly to the ICU

5.3 Clinical:

The following broad categories of clinical data variable types will be prospectively collected from all enrolled patients through medical record and personal interview:

- Baseline demographics e.g. age, gender, race, ethnicity, handedness.
- Baseline socioeconomics e.g. education, employment, living situation, types of support.
- Baseline medical history by system including substance abuse and prior TBI, and medications.
- Mechanism of injury, location, and surrounding circumstances.
- Pre-hospital clinical course variables e.g. vital signs, transport times, GCS score.
- Abbreviated Injury Scale (AIS) score and Injury Severity Score (ISS).
- Brain CT report including presence of skull fracture and intracranial abnormalities.
- Emergency department clinical course e.g. vital signs, GCS, fluids, labs, toxicology, complications.
- Hospital admission clinical course e.g. daily vital signs, GCS, fluids, labs, complications, medications.
- Hospital surgeries and neuromonitoring.

- Reasons for clinical decisions
- Physician based satisfaction with care and prognostic estimates
- Admission and discharge dates and times throughout full clinical course.
- Discharge destination and acute care outcome evaluation.

Clinical data collection will be prospective. Source data can include: patient medical records (paper and electronic), ambulance records, on-line test results, information held on clinical systems. Electronic CRFs will use secure, encrypted connections to the eCRF data. All data will be confidential and stored in locked areas to which only authorized study personnel have access. Records will be coded with a Global Unique Personal Identifier (GUPI) as early as possible so that names and other identifying information will not be linked to personal or sensitive data, in compliance with existing regulations.

5.4. Laboratory assays

In all patients blood samples will be drawn upon presentation for routine laboratory testing as dictated by standard clinical procedures. Details of assays will be captured in the CRF. For study purposes, patients from all strata will additionally have 19 ml of blood drawn <24 hours of injury for biomarker and genetic analysis. While local research protocols may require the banking of additional volumes of blood, this should not exceed 40 ml at admission. Further details can be found in the Laboratory Manual and sampling SOP's. The blood sample will be drawn from an arterial or (central) venous catheter placed as a part of standard care where possible. In other cases patients will need to undergo a blood drawl. No more than 2 venepunctures will take place. Whenever possible, blood draws will be combined with those of routine clinical care.
The additional blood draws performed for purposes of the study will not exceed 40 ml upon presentation; 75 ml during the acute clinical course (only ICU stratum), and no more than 30 ml over a 2-year follow up period.

In the ICU stratum, more extended sampling will be performed in a subset of patients. Cross – sectional sampling at follow-up will be performed in at 6 months in the admission and ICU strata and at other time points in those subjects undergoing MR investigations (Table 1)
Sampling kits will be provided to sites.

5.4.1. Sampling kits: These will be in separate biohazard bags for the biomarker, genetic and advanced haemostasis samples. All sample tubes will be colour-coded. The sample tube colours will be finalized once current tendering processes are completed, and details will be specified in site study manuals. *(Sampling kits will be provided to centres by the Univ of Pecs (UP), which will lead the biomarker workpackage)**:***

1. Sample collection and processing form.
2. Serum separator tube (1x9 ml) and the corresponding needle – (in a separate smaller bag).
3. Cryovials (8x1.2 ml) –labelled with barcodes – (in a separate smaller bag).
4. Sterile disposable plastic Pasteur pipette– graduated (with 0.25 ml graduations) up to 1.5 ml.
5. Potassium EDTA tubes (2x4.9 ml; red cap) – stickered with barcodes – and the corresponding needle – (in a separate smaller bag).

Table 1: Planned sampling points for biomarkers. Figures in columns represent the number of subjects expected to provide samples at each time point, allowing for mortality and loss to follow up. Figures in brackets represent provisional amount of the drawn blood in milliliters.

Time point	Admission/ Presentation	Day 1	Day 3	Day 4	Day 5	2-3 wk	3 m	6 m	12 m	24 m
**ER Stratum (1800)**										

Biomarkers	1800 (9)					600 (9)	200 (9)			
Genetics	1800 (10)									
**Admission stratum (1800)**										
Biomarkers	1800 (9)							1200 (9)	300 (9)	250 (9)
Genetics	1800 (10)									
**ICU stratum (1800)**										
Biomarkers	1800 (9)	1000 (9)	250 (9)	250 (9)	250 (9)	600 (9)		1200 (9)	300 (9)	250 (9)
Genetics	1800 (10)									

<u>Processing of samples for protein ***biomarker*** sampling</u>

Protein biomarker sampling planned in case of each of the 5400 subjects at time points specified in table 1; For each sample 9 ml of blood will be collected into a serum separator tube, centrifuged after 45±15 minutes of coagulation at room temperature, at 4000 rpm for 10 minutes and aliquoted as 8x0.5 ml serum into the barcoded 1.2 ml cryovials. Aliquots should be deepfrozen at -80°C within 3 hours.

If deep freezing is not directly possible for logistic reasons, samples may be stored at -20°C non-frost-free freezer for a maximum of 48 hours before being transferred to -80°C. Samples will be transferred to the central laboratory at the University of Pecs at regular intervals.

6. Genetic studies

For ***genetic*** studies, collection of 2x4.9 ml blood is required in all subjects upon enrolment. Samples of full blood will be collected in potassium EDTA tubes and stored at -80°C ASAP (at least within 6 hours). The two samples should be stored in two different racks (provided by UP) one should stay at the site the other shipped to the central facility in Pecs. Transfer to Pecs will be in batches is at regular interval. We recommend duplicate sampling in order to have a reserve sample available in case any sample gets lost during transport or that DNA extraction process may not be optimal.

7. Neuro-imaging

All acute and at least one follow up brain CT scan performed between day 2 and 7 that are done for clinical care will be collected and uploaded into the CENTER-TBI neuro-imaging repository. CT scans performed as part of clinical care will follow standard clinical practice of the hospital. This will generally include a follow up scan in all patients treated surgically performed within 24hrs of surgery. It is recommended to perform a 3D-volumetric CT with a multi-detector row scanner (32 rows or better).

During upload, the images will be de-identified and de-faced and will only be coded by the assigned Globally Unique Personal Identifier (GUPI) code. All images will be read and coded by central reviewers at Icometrix in accordance with the neuro-imaging TBI CDE's.

No additional CT scans will be performed for study purposes.

8. Outcomes

All outcome measures will be obtained from the patient if they are cognitively able supplemented as appropriate by information from a caregiver or other proxy. Assessments will be administered by telephone/postal questionnaire/web-based questionnaire and face-to-face visits. In order to maximise the number of subjects in whom outcome data are obtained face-to-face visits may be conducted within the local study site, home or other residential/health care setting as appropriate. Where subjects are resident within a long-term rehabilitation care facility some assessments and neuropsychological evaluations (such as the JFK coma recovery scale – revised) may be available from the clinical record. A detailed overview of times of outcome assessments is provided in table 2.

A pre-specified neuropsychological evaluation will be performed in all strata at 6 months after injury and longitudinal at various time points in the three strata up to 24 months after injury (focus on more early outcome assessments in ER stratum and later assessments in Admission and ICU strata).

Two main types of patient follow-up are planned depending on whether only questionnaires are being used or whether a neuropsychological assessment is being conducted (Table 2). The neuropsychological assessment involves face-to-face contact. Travel expenses of patients will be reimbursed according to local site policy. Assessment that only involves questionnaires/ interviews will be conducted by a mixture of telephone follow-up and postal/ web-based questionnaires. If it is more convenient these questionnaire assessments will be completed as part of a visit.

Outcome assessment will include functional outcome (as assessed by the GOSE), heath related quality of life, and patient questionnaires. The preferred method of assessing the GOSE is by interview, and postal/web-based questionnaires will also be options. The face-to-face visits will include formal neuropsychological testing, including paper and pencil tests and the CANTAB test battery, which is language independent and therefore admirably suited for a multinational study. Hardware and software for the CANTAB assessments will be provided by the project organization to sites free of charge. A detailed overview of the instruments used in the various assessments is summarized in table 3. Where applicable, license fees will be covered by the coordinator.

Table 2: Timing of follow up assessments. Numbers at follow up are estimated to be lower than the size of the original cohort, to allow for mortality and for anticipated loss of follow up.

**Time point**		**2-3 wk**	**3 m**	**6 m**	**12 m**	**24 m**
**ER Stratum (1800)**						
Outcome	Neuropsych	600*	600*	1150		
	Questionnaire	1400	1300	1250		
**Admission stratum (1800)**						
Outcome	Neuropsych			1200	300*	250*
	Questionnaire		1450	1300	1200	250
**ICU stratum (1800)**						
	Neuropsych			1200	300*	250*

Outcome	Questionnaire		1450	1300	1200	250

*only in patients undergoing MR studies.

Formal neuropsychological testing will only be performed in patients considered "testable". Assessment of "testability" will be based upon the Galveston Orientation and Amnesia Test (GOAT). Patients with a GOAT ≤ 65 will be considered untestable and in these patients assessment will consist of the JFK coma recovery scale.

Table 1 Outcome Assessment - Instruments and Time Requirements

**Questionnaire Follow-up***	
Telephone interview or postal questionnaire/web based completion/personal interview	Time: 30 min
Participant Questionnaire Part A	10
GOSE	10
SF-12v2	5
QOLIBRI-OS	5
Postal questionnaire/web based completion/personal interview	Time 40 min
QOLIBRI	12
Post-traumatic Stress Disorder (PCL-5)	5
Brief Symptom Inventory (BSI-18)	6
Rivermead Post Concussion Questionnaire	5
SF36v2	12

**Neuropsychology Follow-up***	
Neuropsychology face to face visit	Time: 102 min
GOAT	5
Testable patients:	
RAVLT	15
TMT	7
WAIS-IV Processing Speed Index	10
CANTAB	60
10 meter walk and timed up and go	5
Untestable patients (if GOAT ≤ 65):	
JFK Coma Recovery Scale –Revised	

*For times see table 2

Tests will only be administered by trained study personnel. In case any clinically relevant problems are detected during outcome assessments, the research personnel will notify the medical staff according to local clinical policies and procedures. Any concerns related to a study participant will be discussed with the PI or other senior clinical members of the research team to ensure appropriate arrangements for patient treatment or follow up are in place. In non-urgent cases a letter may be sent to the patients GP outlining these concerns, which will also be copied to the relevant hospital department.

9. Extended studies

Sites have been given the opportunity to contribute to more extended data collection in the following domains:

- MR imaging
- Extended coagulation and biomarker studies
- High resolution ICU monitoring
- Electrocorticographic monitoring
- Continuous EEG monitoring

The selection of sites is determined by expression of interest and logistic considerations.
In addition to data that are collected specifically as part of CENTER-TBI, we will also record additional data that are available at individual centres. None of these data will be mandated as part of CENTER-TBI, and acquisition will depend on local clinical judgment, and will only be collected if part of routine clinical management or another ethically approved study with appropriate consent. Such data could include (but not be limited to) electroencephalography (EEG), cerebral microdialysis, brain oxygen monitoring, and other imaging studies. These data will be used in combined analyses to address the goals of precision medicine and comparative effectiveness research.

9.1 MR imaging

Healthy Controls: nine healthy controls will be recruited within each centre that contributes to the MR data collection. Following informed consent these subjects will undergo the same MR imaging sequences as patients in order to standardize the imaging protocols. Healthy volunteers will be reimbursed for their travel and time.

Centre's participating in the MR data collection arm will undertake MR scans of the brain locally,, using a 3 Tesla scanning, some sites will also undertake ultra-early MR scans (<72 hours post TBI). As the MR protocols are constantly undergoing improvement to reduce imaging times, it is inappropriate to specify exact sequences to be used at each time point. The time points for obtaining MR images and number of patients targeted are specified in table 4. Patients will be cared for according to hospital standards and are instructed to lie still in the scanner, but not to fall asleep. They will be given appropriate hearing protection to maximize patient comfort. Total scan time is estimated at around 45 minutes, and every effort will be made not to exceed a 60 min scan time.
We will ensure that scanning is halted if patients exhibit any significant discomfort. During the study, the patient will be escorted by a suitably trained staff member. Sequences used will include, but are not confined to:

- sagittal 3D T2
- sagittal 3D SPGR/MPRAGE T1
- sagittal 3D T2 FLAIR
- axial 3D SWI

- DTI
- b0 image reversed phase encoding
- fieldmap
- resting state fMRI

Table 4: timing of MR scans

Time point	Ultra Early (<72 hrs post TBI)	2-3 wk	3 m	6 m	12 m	24 m
**ER Stratum (1800)**						
MRI	200	600	250*			
**Admission stratum(1800)**						
MRI	200	600#		600@	300$	150$
**ICU stratum (1800)**						
MRI	200	600#		600@	300$	150$

*We will target patients with abnormalities on previous MR study (patients who have a normal MR at 2-3 weeks will <u>not</u> be invited to return for imaging at 3 months)
#We will attempt to target patients who have ultra-early MR imaging at <72 hours, but recognize that most of the 600 patients imaged at this time point will not have had ultra-early MR imaging.
@We will try to target patients who have had MR images at 2-3 weeks as far as possible for follow up (6 month) imaging
$We will only recruit patients who have had MR imaging at 6 months for late follow up imaging (12 and 24 months)

It is anticipated that all sites participating in the MR acquisition studies will perform these in consecutive patients to the extent that this is logistically possible. Selection for MR studies will therefore not be based on arbitrary selection of individual patients but rather on site selection.

The imaging protocols will be standardized as much as possible and central quality control of uploaded images will be performed by Icometrix. Standardization between centers will be further enhanced by the use of phantoms and by studying nine healthy control subjects per site.

Patients imaged early (<72 hrs after admission) will be longitudinally followed up. In the ER stratum, all patients (n=600) will undergo MR studies at 2-3 weeks after injury; those with abnormalities will be followed up at 3 months. Where logistically possible, we aim to obtain an early MR (<72 hours after injury) in approximately 200 patients. In this stratum, a maximum of 3 MR investigations will be performed per patient.
Patients admitted to the hospital (ward or ICU) will receive an MRI between week 2-4, and after 6 months post-injury. Where logistically possible, we aim to obtain early MR studies (<72 hours after presentation) in approximately 200 patients. Cohorts of the admission and ICU strata will be followed up with longitudinal MR studies at 12 and 24 months. Thus, patients in these strata will undergo at most 5 MR examinations.

All patients undergoing MR studies at follow up will also have blood drawn for biomarker analyses as described in section 5.

9.2 Extended hemostatis studies

Complementary to routine hemostasis tests performed by the local laboratories at sites, approximately 20 centers will perform extended studies for detailed coagulation profiling. This will include central assays for coagulation profiling as well as local points for testing with TEG, ROTEM and multiplate assays. Selection of sites will depend on expressed interest and logistic considerations. We aim to target sites that will be involved in high resolution ICU data collection (section 9.3) as these will in general have existing high level research infrastructures.

9.2.1 Central hemostasis investigations

Sampling for detailed coagulation profiling will involve the collection of 1x2.7 ml blood into a potassium EDTA and 1x10 ml + 1x5 ml blood into two sodium-citrate tubes in case on enrollment in 600/600 subjects from the admission and the ICU strata. In the ICU stratum, further samples will be obtained post-operatively in all patients treated surgically and on day 1.

9.2.1.1. Sampling kits: These will be in separate biohazard bags for the biomarker, genetic and advanced haemostasis samples. All sample tubes will be colour-coded. The sample tube colours will be finalized once current tendering processes are completed, and details will be specified in site study manuals.

Sampling kits will be provided to centres by the Univ ofPecs (UP), which will elad the biomarker package:

1. Sodium Citrate tubes (1x10 ml & 1x5 ml and the corresponding needle – (in a separate smaller bag).
2. Cryovials (7x1.2 ml) – stickered with barcodes – (in a separate smaller bag).
3. Sterile disposable plastic Pasteur pipette – graduated (with 0.25 ml graduations) up to 1.5 ml.
4. Those hemostasis sampling kits which are designated for enrollment time points also contain (in a separate smaller bag):
   - Potassium EDTA tube (1x2.7 ml.
   - Cryovials (2x1.2 ml) – stickered with barcodes.
   - Sterile disposable plastic Pasteur – graduated (with 0.25 ml graduations) up to 1.5 ml – for aliquoting EDTA plasma.

9.2.1.2 Sample processing:

In each case the tubes will be centrifuged at room temperature at 4000 rpm for 10 minutes ASAP and then aliquotted (7x1 ml citrate plasma aliquots) and 2x0.5 ml EDTA plasma aliquots (in case of enrollment time points only) into the barcoded 1.2 ml cryovials. Sampling kits will be provided.

Put the cryovials into the subsequent empty spaces of a cardboard freezer box (provided by University of Pecs) and a -80°C freezer ASAP.
Fill in the central hemostasis sample collection and processing form. Samples will be transferred to the central laboratory at the University of Pecs at regular intervals. From there, samples will be transported to the Universities of Köln and Bonn for further analyses.

9.2.1.3 Central assays:

<u>University of Köln</u>:
Fibrinogen (Factor 1)
Factor V
Factor VIII
Factor XIII (Fibrinase)
d-dimer
Protein C
Activated Protein C
vWF:Ricof (vWF-Reduction)

<u>University of Bonn</u>:
TAT-Complex (Thrombingeneration)
ETP (Thrombinpotential)
F1+ F2 (Prothrombin cleavage frag)
Thrombomodulin
t-PA
a2-AntiPlasman Activity
PAI-1
TAFI + PAP complexes

9.2.2 TEG, ROTEM and multiplate assays

<u>9.2.2.1 Requirements from the sites:</u>

Availability of ROTEM/TEG

<u>9.2.2.2 Sampling process:</u>
Samples for TEG or ROTEM are taken from unmodified blood by venous blood sampling or from arterial catheters. The amount needed for testing is 3 ml. If a regular tube is used a standard time of 15 minutes is recommended to start processing for TEG/ROTEM. If a citrate tube is used, a much larger time window for processing (up to 2 hours) may be permitted.

<u>9.2.2.3 Point of care analysis:</u>
The clotting within the device should be allowed to continue for at least 1 hour. Both the TEG and ROTEM devices will yield a graphic description of the clot formation and lysis. This graphic representation needs to be uploaded into the CENTER-TBI database, either digitally or scanned as pdf. Derived parameters should be entered into the CRF.

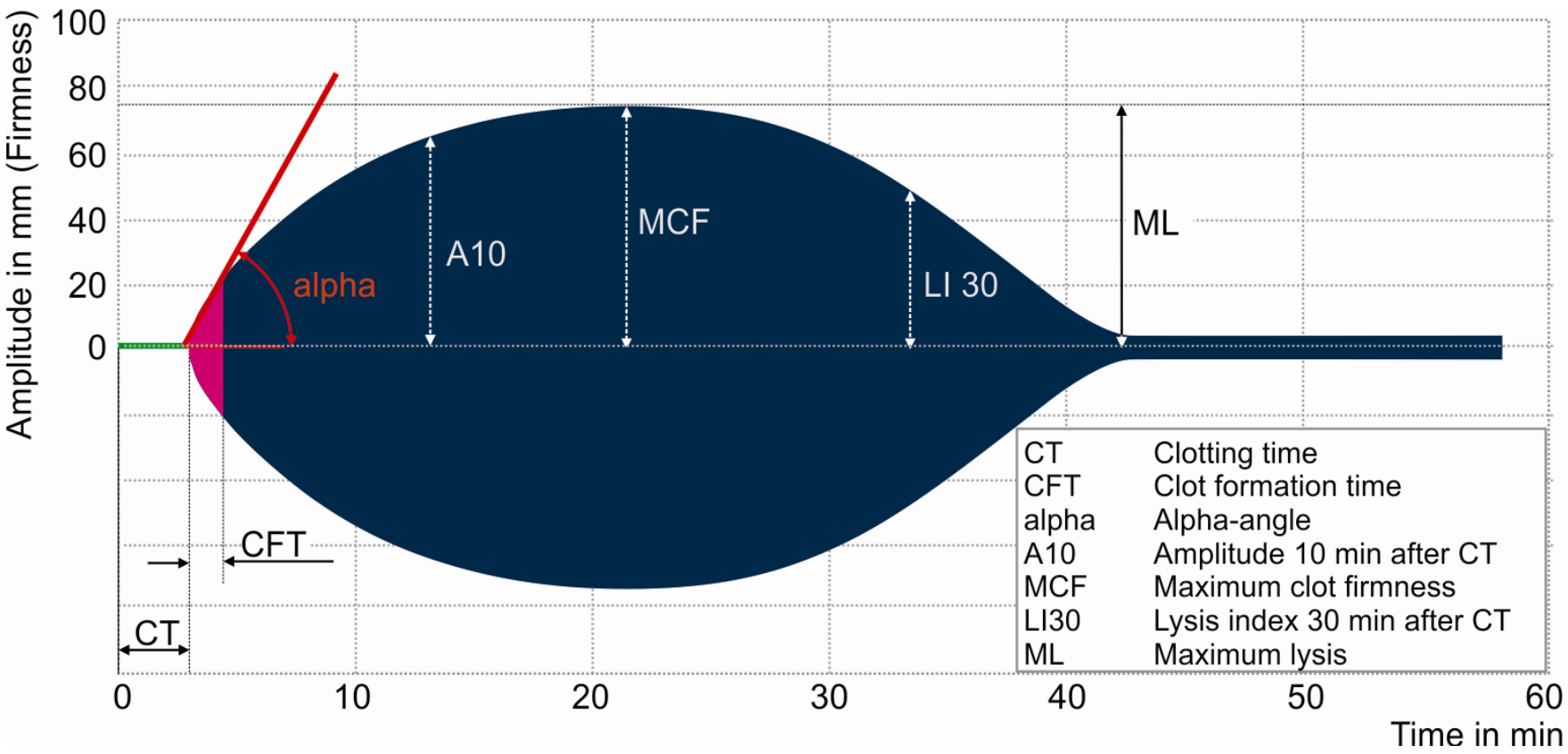

Fig: Example of a normal TEG/ROTEM

A detailed overview of the time points for extended data collection is provided in table 5.

Time point	Enrolment	Post-op	Day 1
ER Stratum (n=1800)			

Haemostasis (central)				
Haemostasis (local)	Clinical	1800 (6ml)		
	Extended	600 (6ml)		
**Admission stratum (n=1800)**				
Haemostasis (central)		600 (16.7ml)		
Haemostasis (local)	Clinical	1800 (6ml)		
	Extended	600 (6ml)		
**ICU stratum (n=1800)**				
Haemostasis (central)		600 (16.7ml)	300 (14ml)	600 (15ml)
Haemostasis (local)	Clinical	1800 (6ml)	300 (6ml)	
	Extended	600 (6ml)	300 (6ml)	600 (6ml)

Table 5 Timing of extended coagulation profiling

Figures in columns represent the number of subjects expected to provide samples at each time point. Figures in brackets represent provisional amount of the drawn blood in millilitres.
In each stratum, patients will have coagulation studies as per normal clinical practice, whilst n=600 will have local extended haemostasis testing (ROTEM and multiplate). It is estimated 50% of ICU patients will undergo an operation prior to or following admission, therefore 300 of the 600 with haemostasis testing completed on enrolment will have both central haemostasis assays collected and local extended haemostasis testing done post operatively.

9.3 High resolution ICU monitoring

Approximately 20 centers will be selected for participation in the high resolution ICU monitoring (HR-ICU). Selection of sites will be based on expression of interest and logistic considerations. Standardization of acquisition of data will be facilitated by providing all selected sites with the ICM+ software (free of charge) and we will facilitate implementation of the Moberg device for standardized acquisition of data.

All ICU centres will contribute hourly physiological parameters (blood pressures, ICP) along with therapeutic intensity levels in all patients in whom ICP monitoring is clinically indicated. The HR-ICU centers will additionally collect ICP, invasive blood pressure and ECG waveforms at sampling frequencies greater than 50Hz in these patients (sites may also wish to contribute other parameters at different time resolutions such as brain tissue oxygenation or microdialysis, but these are not mandated). The data, which will be anonymous and identified only by a CENTER-TBI reference number, will be converted into a standard format and uploaded offline to a central repository in Cambridge which will be securely mirrored. The ICM+ software will be provided and used for data collection and formatting.

Additionally, tablet computers with web-access will be supplied for nurses to upload basic event annotation in real time at the bedside. Sites will provide internet connectivity with either Wi-Fi or 3G (or better) for this tablet. The web interface will submit this data (again anonymously, with a CENTER-TBI reference number) which will be collected in Cambridge and subsequently merged with the other data in collaboration with INCF.

9.4 Electrocorticographic (EcoG) monitoring

ECoG monitoring is performed in many sites for detection of cortical spreading depolarizations, a putative pathomechanism of secondary excitotoxic injury, and is also a more sensitive technique than scalp EEG for seizure detection. The ECoG monitoring itself is not part of the CENTER-TBI protocol and will be subject to separate IRB approval when required by local IRB regulations. Approximately 10-12 centers participating in high resolution ICU monitoring will perform ECoG monitoring in consecutive patients according to local protocols. We expect to recruit ~100 patients overall for this substudy.

In brief, ECoG recordings are obtained from linear electrode strips (Ad-Tech Medical Instruments Corp., Racine, WI, USA) placed surgically on the brain surface and exited in a straight line at the craniotomy margin, as for tunnelized probes. Where applicable, a strip will be placed on viable peri-contusional cortex at highest risk for secondary injury, preferably along a single gyrus. A self-adhesive ground electrode will be placed on the skin and a subdermal needle electrode will be placed under the scalp as a reference. ECoG data will be recorded with the Moberg ICU monitor in a parallel and time-locked manner with other high-resolution variables. Continuous recordings are then obtained in the ICU for the full duration that high-resolution variables (ICP, blood pressure) are collected. At the end of monitoring, the electrode strip will be removed by gentle traction at the bedside under sterile conditions.

9.5 Continuous EEG monitoring

The Moberg system will be used for EEG recording and upload. Electrode placement for continuous EEG monitoring will follow the 10-20 system, and will use a minimum of 8 electrodes featuring a bipolar chain of electrodes (the optimal number is currently undergoing refinement). The EEG electrodes will be applied using either collodion based or needle electrodes (depending on local practice). Application should be as early as possible during the course of the patient's stay in the ICU, allowing for logistic considerations, and the aim should be to retain the electrodes in place for 5-7 days. EEG data will be acquired at a sampling rate of 50 Hz or greater, and uploaded to the high resolution ICU repository along with the other ICU data, using the Moberg device. These patients will also need to have a small amount of additional clinical data recorded about clinical evidence of seizures. No specific funding is currently available from CENTER-TBI to support EEG monitoring; consequently this will only be undertaken in centres that are participating in the high resolution ICU data collection where local resources allow this – this will include the ECoG centres (see **9.4** above). If additional funds become available, the EEG substudy will be supported as feasible extended to other centres.

9.6 Other investigations

In patients undergoing bedside multi-modality monitoring or participating in other related research projects, results of such clinical or laboratory data may be included within the data collection for the core study. These data may include but are not limited to brain tissue oxygen, microdialysis, transcranial Doppler, near infrared spectroscopy, blood flow monitoring and microdialysis or cerebrospinal fluid (CSF) samples. These would only be obtained in those subjects who underwent multimodality monitoring or clinical microdialysis and/or placement of an external ventricular drain (EVD) as part of clinical care or other ongoing research projects. No specific diagnostic interventions will be performed as part of CENTER-TBI. In addition, centres may collect and store an aliquot of urine from a 24-hour collection to measure mediators and metabolites in patients recruited in the study.

10. Informed consent

10.1 Informed Consent Personnel

All study sites are experienced with recruiting TBI subjects, and will have obtained IRB approval to enrol patients into CENTER-TBI. The individuals responsible for identifying potential subjects, explaining the studies, answering questions, and obtaining informed consent will be appropriately trained.

10.2 Location and Privacy

Research staff will locate eligible patients in the hospital (emergency department, hospital wards, intensive care unit), explain the research study, review the consent form, ask the subject or surrogate if she/he voluntarily agrees to participate, and obtain consent.

If potential subjects are approached in the ED, patient care areas can be screened with curtains for privacy. All efforts will be made to ensure the patients confidentiality and privacy during the consenting process within the confines of the clinical area. If the potential subject approaches their time of discharge, then the research personnel will escort the subject and family to one of the privacy areas available in the hospital after discharge from the ED to discuss the study and conduct the informed consent process. The approach to potential subjects in the ED will not be made in such a way that it interferes with or delays the diagnosis and treatment process in the ED. Potential subjects will be given as much time as needed to read the informed consent document, discuss it with family members if they choose, and to ask questions of the research personnel.

10.3 Consent

Informed consent procedures will follow local and national requirements in all cases. We anticipate that many potential patients will not be able to consent themselves to participate in this project. The nature of mTBI means that some patients may lack capacity to decide to participate in this study especially at the earliest time point. It is important to try and include these patients to ensure that representative samples of patients are included to avoid bias in the study findings. Every step will be taken to ensure that a test of capacity is undertaken before a decision on a person's capacity to consent or not to consent to participation in research is taken. If the subject is not capable of self-consent, all efforts will be made to locate a legally acceptable representative to act on behalf of the subject.

When a legally acceptable representative (e.g. consultee/proxy) is identified, their opinion will be sought about the potential participant's wishes and feelings in relation to the project, and whether he or she would have wanted to take part in the study. A member of the research team will approach the consultee/proxy to explain the study and its implications. They will be provided with an information sheet, and given time to read it before having to decide if their friend/relative would wish to take part. If they are willing to provide advice on whether their relative/ friend would want to participate in the study, and agree, they will be required to sign a consultee/proxy declaration form. Members of the research team will be available to answer any questions, or concerns that the consultee/proxy may have both before recruitment and once they are enrolled in the study. Prospective subjects or their proxy will be given as much time as needed to consider study participation.

The patient information sheet and consent form will specify that the local PI be authorised to make contact with the subjects to book and arrange follow up, using a range of options (including telephone calls, text messaging, email, and conventional post), so as to offer patients a wide range of options for communication.

10. 4 Need for reconsent

Should the patient regain mental capacity after commencing this research protocol, consent shall be sought from the patient to request continued participation.

If the subject declines, continued participation will be stopped and the patient given the option to have already collected data removed from the study.

10.5 Withdrawal from study

Subjects are free to withdraw, or be withdrawn by their consultee/proxy if appropriate, at any point in the study, and they need not state a reason.

10.6 Declaration of Helsinki

The study will be performed in accordance with the guidelines of the Declaration of Helsinki adopted by the 18th World Medical Assembly in Helsinki, Finland in 1964 and amended by subsequent assemblies, the most recent being Fortaleza October 2013 as well as the applicable laws and regulations currently in force.

10.7 ICH Guidelines for Good Clinical Practice

Definition from EU Directive 2001/20/EC, article 1, clause 2:
"*Good clinical practice is a set of internationally recognised ethical and scientific quality requirements which must be observed for designing, conducting, recording and reporting clinical trials that involve the participation of human subjects."*

The study will be conducted to ensure compliance with this good practice and provide assurance that the rights, safety and well-being of trial subjects are protected, and that the results of the clinical trials are credible and accurate.
All study team members will have completed GCP training; a copy of each team member's certification will be included in the site file.

10.8 Data handling and record keeping

All recruited patients will be allocated a Global Unique Personal Identification number (GUPI) which will be linked locally to hospital identifiers. The local PI will ensure that patient confidentiality will be maintained at all times. All uploaded data will be de-identified prior to upload. While blood samples and clinical data will be linked, both sets of data will be kept confidential and anonymised beyond the initial stage of correlation for analysis.

All imaging and electronic data will be kept on individually password protected servers. The network is only accessible by persons with access rights, approved by the data controller. Clinical data will be stored in secure locations. Where applicable, information relevant to the patient's care will be made available to the physician responsible.

Data, including blood samples collected as part of this study will be shared in an anonymised form with collaborators from other EU states (this is part of a European Commission Framework7 funded program), and with selected collaborators in other countries who form part of an emerging **In**ternational **T**raumatic **B**rain **I**njury **R**esearch initiative (InTBIR; http://intbir.nih.gov/).

10.9 Ethical committee review

Before the start of the study or implementation of any amendment we will obtain approval of the study protocol, protocol amendments, informed consent forms and other relevant documents. All correspondence with the Research Ethics Committee (REC) will be retained in the Trial Master File/Investigator Site File.
Annual reports will be submitted to the IRBs in accordance with national requirements. It is the Principal Investigators responsibility to produce the annual reports as required.

11. Subject risks and benefits

11.1 Foreseeable risks and their minimization

No therapeutic interventions will take place in the context of the observational studies. Diagnostic interventions include blood sampling, outcome assessments and in selected sites MR imaging, HR ICU monitoring and extended blood sampling. The potential risks to the subject are minimal across all domains of data collection.

Clinical. Study data will be entered into eCRFs using secure, encrypted connections to the eCRF data. All data will be confidential and stored in locked areas to which only authorized study personnel have access. Records will be coded with a GUPI as early as possible so that names and other identifying information will not be linked to personal or sensitive data, in compliance with existing regulations. In addition, subjects and their families will be informed that participation is completely voluntary, that they may decline response to any questions they may find distressing, and that they may withdraw from the study at any time, all without jeopardizing medical treatment to which they are otherwise entitled.

Biospecimens. The blood sample will be drawn from an arterial or (central) venous catheter placed as a part of standard care for those patients consented while in the ICU. Those patients consented on the ward or emergency room will need to undergo a blood draw and may experience the discomfort associated with a needle stick and may suffer bruising at the site of the needle stick. This will be minimized as much as possible. No more than two venepuncture attempts will take place. Whenever possible, blood draws will be combined with those of routine clinical care. The additional blood draws performed for purposes of the study will not exceed 40 ml upon presentation, 75 ml during the acute clinical course (only ICU stratum), and no more than 30 ml over a 2-year follow up period.

Genetic Research. The highest degree of privacy protection is required with regard to genetic data, as a possibility exists that this information could affect one's ability to be insured, employed, future decisions regarding children, or family relationships. All data will be de-identified, and results only

coded by the GUPI code. All analyses will be performed centrally after completion of recruitment. The information linking the GUPI code to patient data will be retained locally at study sites, and will be stored in secure areas to which only authorized study personnel have access. Given the delay between sample collection and genotyping, the research (rather than clinical) standards used for genotyping, and the enormous variation in the facilities available for counselling and follow up, we have elected not to make incidental genetic results available to participants.

Neuroimaging. Subjects at selected sites will undergo brain imaging using 3 Tesla MR scanners at 2 weeks and 6 months post-injury. No exogenous contrast agents and no sedation will be used solely for purposes of the study. The MRI procedures are non-invasive and painless. The MRI does, however, require the subject to lie still with the head and part of the body confined in a tunnel-like device for a considerable length of time (total scan time of at most 60 minutes). If the subject finds it uncomfortable to lie still in the MRI scanner for the duration the scan, they will be asked by the scan operator if they would like breaks between sequences.

Contraindications for the MRI examination include those who have cardiac pacemakers, neural pacemakers, surgical clips in the brain or blood vessels, surgically implanted steel plates, screws or pins, cochlear implants, intrauterine devices, or metal objects in their body, especially in the eye. Subjects will be required to remove all ferromagnetic items (e.g. keys, phones, credit cards, belts, loose change, and others) before entering the MRI examination room. Claustrophobia may also preclude successful MR imaging. Careful screening will prevent such individuals from participating in this study, as well as preventing the introduction of any ferromagnetic objects into the scanner room. Dental fillings do not present a problem.

During scanning precautions will be maintained so that specific absorption rates (SAR) will be less than 8 watts per kilogram in any 1 gram of tissue. This is the estimated power required to raise the temperature 1 degree centigrade. The maximum dB/dt will be set at 20T/sec for > 120usec or 200T/sec for < 12usec. These levels are well below peripheral nerve stimulation threshold in humans, both children and adults. In rare cases, subjects may still experience some peripheral nerve stimulation during portions of the MRI procedure. These experiences are transient and harmless. MRI participants will be instructed prior to examination to refrain from skin-on-skin contact of their extremities (e.g. clasping hands or legs) to further reduce this risk. The MRI will produce loud noises during image acquisition. The decibel intensity of these noises is not considered harmful. Subjects will be provided with earplugs and noise-cancelling headphones/earpads/earplugs to minimize discomfort.

Where subjects are conscious, they will always be in communication with the MRI technologist and will be given a sensor that triggers an alarm. If the subject indicates at any point that they have a desire to stop the procedure, the exam will be terminated immediately and without any penalties to the subject in any way.

If any unexpected findings are identified that may be clinically significant, we require that local PIs follow local policy for review, reporting and counselling. Where the data are from a patient, and pertain to TBI, we recommend that the clinician caring for the patient should be made aware of the findings. Non-TBI related findings (e.g. incidental developmental abnormalities, or a intracranial tumour discovered on MR) in patients or incidental findings in healthy controls should be dealt with based on locally agreed protocols – in many centres the arrangement is that the participant is counselled by the Site PI and recommended to seek medical care from their primary care physician.

Outcomes. Some of the questionnaire assessments deal with issues such as emotional distress, and these have all been used in previous studies. There are no known risks carried by any of the assessments, including items concerning self-harm. This will be explained to participants both orally and in the consent document. Further, only trained study personnel will administer such interviews

and questionnaires. All participants will be given information concerning local sources of help for common forms of distress after TBI. If a participant gives any indication that they might self-harm, it will be documented in the research record and the research personnel will notify the medical staff according to local clinical policies and procedures.

11.2 Potential benefits of proposed research

There is no direct benefit to study participants other than by enhanced contacts and more detailed study assessments. The results will be directly relevant to society in general and to future patients who suffer TBI (see section 11.3).

CENTER-TBI subjects will undergo comprehensive neuropsychological, psychological and health-related quality of life testing and brain imaging. These data will be made available to clinicians caring for the patient, as judged appropriate by the local PI. The Study Coordinators will not mandate disclosure of these results to patients or their families, since we recognise that wide variations exist in local practice and custom. However, where local practice dictates that these data to be made available to patients and/or their families, the decision to provide such data will be the responsibility of the local PI. These procedures are not part of the standard of care for mild TBI. Allowing for variations in practice, and recognising that local PIs may come to different implementation protocols for return of data to patients, we anticipate that where such disclosure is permitted and planned, subjects will have access to the results of their research MRI results within 2 weeks of their respective 2 week and 6 month timepoints. If requested, subjects will receive a CD of their conventional MR imaging data and a viewer application tool. Upon request, subjects will be provided the results of their outcome tests after completion of their 12-month outcomes, as to minimize the feedback and undue influence of test results on the subjective perception from the research subjects during the study. All participants may share their study information with their care providers as they choose. Investigators will also be available for consultation with subject’s care providers to interpret these findings. All MRI and outcomes data provided to subjects will be stripped of Study ID.

11.3 Benefits and impact of the CENTER-TBI project to patients, health care professionals and policy makers.

Patients: TBI is not limited by any borders. The need for European action is dictated by national and regional differences in resource, treatment approaches and healthcare delivery, which impact on outcome. These inequalities in treatment provision and outcome are not small: Analysis of clinical trial data shows 3.8 fold differences in risk adjusted outcomes across Europe (Lingsma et al 2011), and a recent EU report (https://webgate.ec.europa.eu/idb/) recognised that 100,000 lives could be saved annually if injury mortality rates across Europe could be reduced to the lowest observed national rate. *CENTER-TBI will provide robust guidelines on best clinical practice, ensuring that every EU citizen obtains the best possible care, regardless of country or region of domicile. This will improve outcome and quality of life for individual patients.* We will also develop accessible information for patients and families, empowering them as partners in their own care. This will include information on early and reliable outcome prediction (providing hope and decreasing unrealistic expectations).
Various approaches will be adapted to enhance visibility and interaction with patient groups; these include an open website, press releases, establishment of a public information platform and use of social media. TBI is the commonest cause of deaths in hospital trauma attendances hence we would anticipate our CER findings to save ~20,000 EU lives per annum in a predominately economically active population, and reduce disability in survivors.

Health Care Professionals: We anticipate that our study will transform characterization of TBI, and improve detection and understanding of disease processes, mirroring the recommendations of the National Academy of Science (NAS) on the importance of developing a new taxonomy in the context of Precision Medicine. The expected impact of CENTER-TBI is displayed in figure 3.2, adapted from the NAS report. We expect that improved disease characterization and identification of best practices will lead to therapies that are better targeted and more individually oriented (Precision Medicine).Knowledge gained from the CER analyses will be integrated with systematic reviews of the existing literature to produce improved and harmonized clinical guidelines, facilitating constant improvement by the clinical neurotrauma community.

Policymakers: Insight into current epidemiological patterns of TBI across Member States will inform prevention campaigns, targeted to needs at national levels. Our focus on the impact of systems of care and organizational aspects of care delivery could yield substantial benefits: for example introduction of the UK NICE Guidelines for TBI management was associated with a 12% reduction in TBI mortality (Fuller et al 2011). More efficient and targeted care and improved outcome will reduce costs. New performance indicators and improved prognostic models will facilitate benchmarking and assessments of quality of care.

In summary, the CENTER-TBI project will contribute towards the overall goals of InTBIR, by identifying more effective and efficient treatment provision, thus improving outcome and reducing costs. The science in the project will provide novel information on disease processes, treatment, outcome, and prognosis in TBI, identifying new therapeutic targets and therapies; while the CENTER-TBI repositories will ensure opportunities for legacy research. Thus, the project has the potential to improve current health care and its delivery at both population and individual levels, deliver early scientific advances that could improve the care of patients with TBI, and provide a rich investment for future biomedical research.

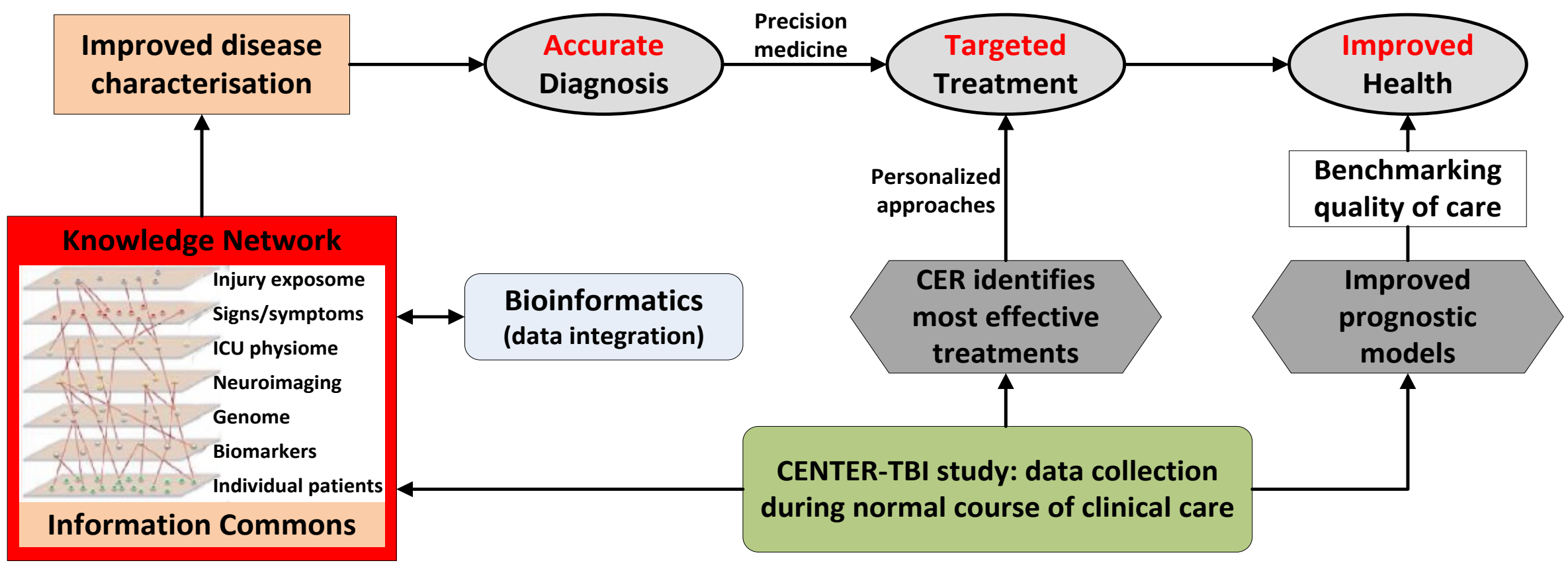

Figure 3.2: The CENTER-TBI participants represent a knowledge network that will analyze the clinical database and associated biorepositories. This highly granular "information commons", aided by novel bioinformatic approaches, will improve disease characterization, resulting in a new taxonomy of TBI (Precision Medicine). Clinical data will be subjected to CER analysis, both directly, and after refinement with precision medicine to identify more effective and targeted therapies. The increased data inputs will improve prognostic accuracy, allowing better benchmarking of care (Adapted from National Research Council)

11.4 Adverse events

No structured adverse event reporting will be implemented, as this is an observational study without any therapeutic intervention. However, we will capture any serious complication which may occur during the clinical course in the CRF.

12. Data Management and Compliance

Clinical monitoring. Data collection for each timepoint and Core type must be completed accurately and to schedule. Data monitoring will utilize both on-line monitoring of web entry forms and source data verification at the site level to optimize efficiencies and reduce data discrepancies. Furthermore, the study staff and staff of the study CRO will conduct site visits to monitor protocol compliance, complete written monitoring reports, and deliver findings to the Coordinators. Visits will be performed at a minimum of 1 visit at each site, and further targeted to performance and recruitment and protocol (clinical, biospecimen, neuro-imaging and outcome). Protocol processes, including enrolment practices, data collection, imaging, and biospecimen collection procedures, will be assessed over the course of the study period. Site visits will be conducted to review and adjudicate subject records in accordance with CENTER-TBI monitoring procedures. Some records will receive a targeted review that may include items such as informed consents, eligibility, inclusion/exclusion criteria, protocol-specific biospecimens collection, neuroimaging procedures, and outcomes administration and scoring standards. The study staff will review results from monitoring visits and regularly scheduled data checks to identify trends and problems, and will share these results with the Executive Committee on a regular basis.

Data storage. Prior to upload to the study database, all acquired data will be stored locally. Imaging data will be stored on the centre's digital image archive system, and all other data on a secure computer, access to which must comply with all local requirements for data accesses and security. These data will be periodically uploaded to the study database using the GUPI system described below, using a secure link.

Clinical database. At study enrolment, the system will generate study identifiers to reference patients. The StudyID will be the following format:

XX (two character country identifier) - YYY (three character site identifier) - 9999 (four digit patient identifier)-99 (two character check digit)

The first patient enrolled at a site will have the number 1001. Numbers 0001-1000 are reserved for test data and training. The check digits are added to ensure that a number is not mis-keyed and an incorrect patient selected for data entry.

The system will also create a Generated Unique Patient Identifier (GUPI). In the "Patient Add" process, the system will have a local function that will collect elements that uniquely identify a patient (full name at birth, date of birth, location information) and create a computer generated code (for example: e8ea7a8d1e93e8764a84a0f3df4644de). The generated code is a "one-way hash" which means that entered data will always generate the same code, but the code cannot be reversed to generate the data. This one-way hash will be sent to the server and a complete random GUPI will be assigned. It will be a characters string (for example LPYUQR) that will be stored in the patient record.

The one-way hash will be generated on the local computer and not stored anywhere. The GUPI will be assigned randomly, and will not be traceable to the patient. No identifying data will ever leave the local computer.

MRI scans will have an additional label at the end to distinguish the timepoint and whether the scan is a patient, phantom, or control. The additional labels are as follows: (XX-XXX-9999_2WK for 2-week

MRI, _6MO for 6-month MRI, _PHA for phantom, and _CTL for control). Clinical data will be entered into eCRFs via a web-based portal to the secure, clinical database. Registry data will be anonymised by a randomly generated code.

Automated data integrity monitoring. All clinical data will be entered into electronic Case Report Forms (eCRFs) and managed by the QuesGen data management platform which will be developed in collaboration with INCF. As data is entered into each form, the system will run data validation checks that include conditionally required data, validation across fields, and validation requirements based on subject type. If any validation check fails, the user is alerted immediately that the data does not meet QA criteria and the issue can be addressed and corrected at that point. If a data element fails a validation check, yet the value entered is correct, the user can enter an exception to the problem and provide a notation as to why the out-of-range data is actually correct. Data validation checks include:

- **Date/time value checks:** all dates and times entered into the database are checked to ensure that events recorded are accurate and in sequence.
- **Range value checks:** all numeric, non-date fields have range values specified to minimize data entry errors.
- **Selection lists:** all categorical data fields have predetermined drop-down lists, check boxes, or resettable radio buttons instead of free text to ensure accuracy.
- **Logic checks:** data fields from different sections of the eCRF will be compared to pass logical integrity
- **Required fields:** the eCRF will be programmed to require input into fields when appropriate to minimize missing information.
- **Score calculation** will be performed and programmed into eCRFs for tests and measures with numerical score summations or norming to avoid mathematical errors by the examiner. All automated scoring computations will be fully documented and validated by QuesGen and the Clinical Core, and must pass User Acceptance Testing.
- **Electronic data audits** will be automated in the QuesGen database through a series of pre-determined queries against the study database at regular intervals. These queries will be designed for the CRO and study staff to monitor data quality and completeness and identify protocol variations/deviations/violations.
- **Data audits against source documents**, where available, will be conducted in approximately 10% of subjects.

All investigators and designated study personnel will have unique and confidential password access to the QuesGen database. All access to the database and to study data will be logged in an audit trail and monitored. Any indication of inappropriate access will be reported immediately to the study coordinators. Investigators will have access to their data at any time.

The database system will also provide checks for form completion based on the subject type. Validation rules will establish when forms for a particular subject should be entered, and any missing forms can be tracked by the Study Site and study management immediate follow-up. Once subject forms are marked complete, a dataset for sharing can be created. The QuesGen platform stores the exact dataset that is shared for future reference and also tracks information about when the data was shared and the dataset recipient.

Due dates for eCRF completion windows are set by the study management. The QuesGen system will automatically generate reminders to complete eCRFs for enrolled patients. Monthly reports of enrollment, timeliness of eCRF completion and error correction will be monitored and adjudicated by the CRO.

Integration with analytics platforms. All de-identified electronic study data in the CENTER-TBI database will be stored securely in the European data space under supervision of INCF for the duration of subject enrolment and follow-up and for a period afterwards for data analysis and preparation of publications. We estimate that the analysis and publication period will last for several years after the conclusion of subject enrolment.

Together with QuesGen Systems, INCF will ensure that data standards are established for the data model e.g. conformity of field formats, field codes and names to ensure consistency across all datasets. Any approved changes will be fully documented with dataset updates to maintain data quality and accuracy. INCF will be responsible for importing cleaned datasets to other analytic platforms as determined by the coordinators.

13. Clinical protocol maintenance

13.1 Protocol modifications

Investigators wishing to modify the Clinical Protocol will submit proposed changes to the study management for discussion. Upon approval, the proposed modifications will be communicated to all sites. The proposed modifications will be communicated to all sites for revision of their respective operational procedures and IRB protocols.

13.2 Protocol deviations

Protocol compliance and study performance will be monitoring by the study staff and CRO using the study reports and dashboards provided by QuesGen Systems. Protocol deviations need to be reported to the Coordinators, REC and CI as appropriate. In the event that a consistent pattern of poor performance (e.g. not enrolling allotted amount of patients per quarter, not achieving adequate follow-up rate across all timepoints) or inadequate compliance (e.g. insufficient blood draw amount, CT or MR imaging, CRF completion without errors, full outcomes battery completion, or any timepoint completed outside the approved window) is detected, the responsible site investigator will be notified and required to present a plan for improvement and a time line for accomplishing this to the study staff. Failure to meet objectives specified in this plan may result in termination of study participation or assignment of another investigator.

14. Publication

All main multicentre publications that result from the CENTER-TBI Study utilizing data from substantially all participating centres will include in the authorship designation: “on behalf of the CENTER-TBI participants and investigators”. All scientific participants and all principle investigators of the centres will be listed as appendix to the manuscript, resulting in a qualification by PubMed as contributing author. For such publications, the coordinators will make available to the Principal Investigator such data and information in a form reasonably deemed necessary by the coordinators to permit the Principal Investigator to contribute, in accordance with generally recognized standards for academic publications, to any such publication. The Principal Investigator will cooperate promptly with the coordinator’s requests for review and comment of any proposed multi-centre publication.

Local investigators have the right to publish or present the results of the sites activities conducted in the context of CENTER-TBI. However, it is recognized that the study is part of a multicentre study and that any publication by the site of its results of the study should not be made before the first multi-centre publication. Sites are entitled to publish research that includes study data, but is unrelated to the CENTER-TBI study at all times. All proposed publications should be submitted in draft format to the Management Committee at least 45 days prior to any submission of work for a publication, to ascertain that no patentable subject matter or confidential information is disclosed therein. All publications should acknowledge the funding source as follows:
"Data used in preparation of this manuscript were obtained in the context of CENTER-TBI, a large collaborative project funded by the European Commission Framework 7 program (grant 602150)."

15. Insurance and liability

The local site retains all responsibility, medical and otherwise, to provide the best care for their patients.
Prior to the commencement of the Study, the Sponsor shall secure insurance coverage to cover its liability in relation to subjects in compliance with article 29 of the Belgian Law of May 7, 2004 concerning Experiments on Humans.

References

Brain Trauma Foundation. American Association of Neurological Surgeons; Congress of Neurological Surgeons. Guidelines for the management of severe traumatic brain injury. J Neurotrauma. 2007; 24:S1–106.

Fuller G, Bouamra O, Woodford M, Jenks T, Patel H, Coats TJ, Oakley P, Mendelow AD, Pigott T, Hutchinson PJ, Lecky F. Temporal trends in head injury outcomes from 2003 to 2009 in England and Wales. Br J Neurosurg. 2011 Jun; 25(3):414-21. Epub 2011 Apr 22.

Institute of Medicine. Initial National Priorities for Comparative Effectiveness Research. June 2009 http://www.iom.edu/Reports/2009/ComparativeEffectivenessResearchPriorities.aspx

Lingsma HF, Roozenbeek B, Li B, Lu J, Weir J, Butcher I, Marmarou A, Murray GD, Maas AI, Steyerberg EW. Large between-center differences in outcome after moderate and severe traumatic brain injury in the international mission on prognosis and clinical trial design in traumatic brain injury (IMPACT) study. Neurosurgery. Mar;68(3):601-7; discussion 607-8. 2011.

Maas AI, Stocchetti N, Bullock R. Moderate and severe traumatic brain injury in adults. Lancet Neurol. 2008; 7(8):728–41

Maas AI, Menon DK, Lingsma HF, Pineda JA, Sandel ME, Manley GT. Re-orientation of clinical research in traumatic brain injury: report of an international workshop on comparative eff ectiveness research. J Neurotrauma 2012; 29: 32–46.

Masel BE, DeWitt DS Traumatic brain injury: a disease process, not an event. J Neurotrauma. Aug;27(8):1529-40. 2010.

National Institute for Health and Care Excellence. Head injury: Triage, assessment, investigation and early management of head injury in children, young people and adults. 2014

National Research Council (US) Committee on A Framework for Developing a New Taxonomy of Disease.
Toward Precision Medicine. Washington (DC): National Academies Press (US); 2011.

Rosenfeld JV , Maas AI, Bragge P, Morganti-Kossmann MC, et al. Early management of severe traumatic
brain injury. Lancet. 2012;380:1088-98
