# Supplementary material for: The CBI-M framework for characterization of traumatic brain injury: Evaluation of performance using data from the CENTER-TBI and TRACK-TBI prospective cohort studies
Source: PLoS Med. 2026 Oct 20;23(10):e1004945. doi: 10.1371/journal.pmed.1004945 (PMC13681731; doi:10.1371/journal.pmed.1004945)
Supplement: Supplementary file 17 [file pmed.1004945.s017.pdf]

Transforming Research and Clinical Knowledge in Traumatic Brain Injury

Clinical Protocol

January 18, 2018

Version 18

Transforming Research and Clinical Knowledge in Traumatic Brain Injury (TRACK-TBI)
Clinical Protocol

Table of Contents

1. Leadership, Sites and Contacts 4
   1.1. Leadership 4
   1.2. TRACK-TBI Principal Investigators 4
   1.3. TRACK-TBI Steering Committee Members 5
2. Study Overview 6
3. Structure and Governance 7
   3.1. Steering Committee 7
   3.2. Executive Committee 7
   3.3. Innovative Distributed Cores Model 8
   3.4. Public-Private Partnership Resources 9
   3.5. Scientific Advisory Board 9
   3.6. Community Advisory Board 9
4. Objectives and Specific Aims 10
   4.1. Specific Aim 1 10
   4.2. Specific Aim 2 10
   4.3. Specific Aim 3 13
   4.4. Specific Aim 4 16
5. Study Design and Sample Size 19
   5.1. Subject Groups 19
   5.2. Assessment Cohorts 20
   5.3. Patient Cohort Selection for MRI 20
   5.4. Protocol Grid 22
   5.5. Milestone Plan 23
6. Subject Eligibility 24
   6.1. Inclusion/Exclusion Criteria 25
7. Subject Procedures by Core 26
   7.1. Clinical 26
   7.2. Biospecimens 26
   7.3. Neuroimaging 26
   7.4. Outcomes 27
8. Subject Recruitment and Screening 30
   8.1. Subject Identification 30
   8.2. Screening Process 30
   8.3. Participation Requirements 30
   8.4. Subject Compensation 31
9. Informed Consent 32
   9.1. Informed Consent Personnel 32
   9.2. Location and Privacy 32
   9.3. Competency Screening 32
   9.4. Language and Literacy 33
   9.5. Need for Reconsent 33
   9.6. Storage of Consent Documents 33
   9.7. Waiver of Consent 33
10. Subject Risks and Benefits 34
   10.1. Foreseeable Risks by Core 34
   10.2. Protections Against Subject Risks 35

10.3. Certificate of Confidentiality 36
10.4. Potential Benefits of Proposed Research 36
11. Subject Compliance and Retention 38
12. Data Management and Compliance 39
12.1. Clinical 40
12.2. Biospecimens 41
12.3. Neuroimaging 42
13. Adverse Events 43
14. Data Sharing 44
14.1. Internal Data Sharing 44
14.2. External Data Sharing 44
15. Clinical Protocol Maintenance 45
15.1. Protocol Modifications 45
15.2. Protocol Deviations 45
16. TRACK-TBI LONGITUDINAL STUDY 46
17. Reference List 47

TRACK-TBI Clinical Protocol Appendices

(All appendices can be found in the "Clinical Protocol Appendices" folder on Dropbox unless otherwise noted)
Appendix 1: Pediatric Protocol (see "Pediatric Core" on Dropbox)
Appendix 2: Screening and Consent Forms
2.1: Screening Log
2.2: Informed Consent Form
Appendix 3: Clinical Case Report Forms
Appendix 4: Biospecimens Full Protocol (see "TRACK-TBI Biospecimen SOP" in "Biospecimens Core" on Dropbox)
Appendix 5: Neuroimaging Full Protocol (see "TRACK-TBI MRI Manual" in "Imaging Core" on Dropbox)
Appendix 6: Outcomes Full Protocol
6.1: Adult Outcome Measures (see "Outcome Assessment SOP" in SOP folder under "Outcomes Core" on Dropbox)
6.2: Pediatric Outcome Measures (see "Outcomes Folder" in "Pediatric Core" on Dropbox)
Appendix 7: Policy and Procedure for Revisions to the TRACK-TBI Manual of Operating Procedures
Appendix 8 v 3: Control Enrollment Site Targets and Procedures
Appendix 9: Site Contact Lists
Appendix 10: Data Dictionary for Emergency Department and Hospital CRF MOP
Appendix 11: Guidance for continued TRACK-TBI sub-study enrollment/follow up after final TRACK-TBI enrollment
Appendix 12: TRACK-TBI LONG SOP

1. LEADERSHIP, SITES AND CONTACTS

1.1 LEADERSHIP

TRACK-TBI is a large and complex project. Its institutional and public-private partnership is comprised of 18 clinical sites, 7 Cores, for a total of nearly 50 collaborating institutions, corporations, and philanthropy. From inception, we recognized that a clear and comprehensive governance and leadership plan would be essential to the success of the scientific and administrative aspects of the project. The multi-PI plan presented here emphasizes excellence, inclusiveness, collaboration, and efficiency.

Governance will implemented by the PIs, advised by a Steering Committee, to promulgate critical policy and strategic decisions, executed by an Executive Committee, through the work of a model of distributed Cores of scientific and administrative expertise (Administrative, Clinical, Biospecimens, Informatics, Neuroimaging, Outcomes, Biostatistics/Comparative Effectiveness Research). Each of the 7 PIs, and a specially appointed Informatics expert, serve as the Core Leaders, sometimes in conjunction with a Core Co-Leader who has complementary expertise. At each of the 18 Study Sites, we have appointed a Study Site Leader, who works with 1 or more Co-Investigators at that site, selected for their mix of strengths across the Cores. The Leadership will also benefit from the input of a Scientific Advisory Board and a Community Advisory Board, as to strategic research participation and planning, and dissemination of TRACK- TBI scientific findings.

1.2 TRACK-TBI PRINCIPAL INVESTIGATORS (EXECUTIVE COMMITTEE)

Name	Role	Institution
Geoffrey Manley, MD, PhD	Contact PI + Administrative Core Leader + Study Site Leader	University of California, San Francisco
Claudia Robertson, MD	PI + Clinical Core Leader + Study Site Leader	Baylor College of Medicine
David Okonkwo, MD, PhD	PI + Clinical Core Leader + Study Site Leader	University of Pittsburgh
Ramon Diaz-Arrastia, MD	PI + Biospecimens Core Leader	Center for Neuroscience and Regenerative Medicine, USUHS
Nancy Temkin, PhD	PI + Biostatistical/CER Core Leader	University of Washington
Pratik Mukherjee, MD, PhD	PI + Neuroimaging Core Leader	University of California, San Francisco
Joseph Giacino, PhD	PI + Outcomes Core Leader	Spaulding Rehab Hospital Harvard Medical School
Rotating Steering Committee Member		

1.3 TRACK-TBI STEERING COMMITTEE MEMBERS

Name	Role	Institution
Principal Investigators (7)	See above	See above
Ann-Christine Duhaime, MD	Clinical Core Co-Leader (Pediatrics) + Site Leader	Harvard Medical School-MGH
Dana Goldman, PhD	Biostatistical/CER Core Co-Leader	Univ. Southern California
Arthur Toga, PhD	Informatics Core Leader	Univ. Calif. Los Angeles
Kevin Smith, MSIS	Informatics Core Co-Leader	Univ. Michigan
Opeolu Adeoye, MD	Study Site Leader	Univ. Cincinnati
Neeraj Badjatia, MD	Study Site Leader	Univ. Maryland
Randall Chesnut, MD	Study Site Leader	Univ. Washington
Mitchell Cohen, MD	Study Site Leader	Denver Health Medical Center
Gregory Hawryluk, MD, PhD	Study Site Leader	University of Utah Health Care
Gillian Hotz, PhD	Study Site Leader	Univ. Miami
Christopher Madden, MD	Study Site Leader	Univ. Texas South Western
Michael McCrea, PhD, ABPP-CN	Study Site Leader	Medical College of Wisconsin
Randall Merchant, PhD	Study Site Leader	Virginia Commonwealth Univ.
Uzma Samadani, MD PhD	Study Site Leader	Hennepin County Medical Center
David Wright, MD	Study Site Leader	Emory University
Mateo Ziu, MD	Study Site Leader	Seton Brain & Spine Institute Austin
Andrew IR Maas, MD	Member + PI of CENTER-TBI/InTBIR	Antwerp University Hospital, Edegem, Belgium
David Menon, PhD	Member + Co-PI of CENTER-TBI/InTBIR	University of Cambridge, Cambridge, UK
Isabelle Gagnon, MSc PhD	Member + PI Canadian Institutes of Health Research (CIHR) InTBIR Team Grant	McGill Univ. Health Centre, Montreal, Canada
Ryan Kitagawa, MD	Study Site Leader	UTHSC – Houston<br>Baylor College of Medicine

2. STUDY OVERVIEW

Effective treatment of traumatic brain injury (TBI) remains one of the greatest unmet needs in public health. Each year in the United States, at least 1.7 million people suffer TBI; it is a contributing factor in a third of all injury-related US deaths. An estimated 3.2 to 5.3 million people live with the long-term physical, cognitive, and psychological health disabilities of TBI, with annual direct and indirect costs estimated at over $60 billion.[1] Recent efforts have increased our understanding of the pathophysiology of TBI; however, these advances have failed to translate into a single successful clinical trial or treatment.[2] These failures are largely attributable to the fact that TBI classification approaches are blunt and have not changed in more than 3 decades. TBI patients are divided into the crude categories of mild, moderate, and severe, using the Glasgow Coma Scale (GCS)[3], outcomes are measured using the equally crude Glasgow Outcome Scale-Extended (GOSE).[4] These symptoms-based categories do not permit mechanistic targeting for clinical trials. Clinical research has also been underpowered, hampered by lack of data standardization, and with limited multidisciplinary collaboration. Workshops coordinated by the National Institute of Neurological Disorders and Stroke (NINDS), Department of Defense (DOD), and the National Institute on Disability and Rehabilitation Research (NIDRR) since 2007 identified the urgent need for improved TBI classification using more accurate diagnostic and outcome tools (beyond the GCS and GOSE), along with a standardized approach to data collection. A multidisciplinary effort was launched to develop TBI Common Data Elements (TBI-CDEs). Domains included clinical data, imaging, biospecimens, and outcomes.[5-8]

In 2009, the NINDS-funded, multicenter Transforming Research and Clinical Knowledge in Traumatic Brain Injury Pilot study (TRACK-TBI Pilot - PI, Manley) implemented and helped to refine the TBI-CDEs, and created a prototype of the TBI Information Commons proposed here. This effort validated the feasibility of the TBI-CDEs and collected detailed clinical data on 650 subjects across the injury spectrum, along with CT/MRI imaging, blood biospecimens, and detailed outcomes. It also established an infrastructure of integrated databases, imaging repositories, biosample repositories, and multicenter expertise. The TRACK-TBI Pilot dataset is the first to populate the Federal Interagency Traumatic Brain Injury Research (FITBIR) repository. Among the early findings of TRACK-TBI Pilot data: Co-Investigator (Co-I), Yuh et al[9] identified early MRI abnormalities in ~30% of subjects with mild TBI and a normal CT scan. These structural MRI abnormalities, in the first-ever standardized reporting of imaging features employing TBI-CDEs, were predictive of unfavorable outcome at 3 months. This work represents a significant step toward improved stratification of heterogeneous patient subgroups within the traditional “mild TBI/Concussion” population. Additionally, we validated a blood-based glial proteomic biomarker that reliably detects the presence and severity of brain injury seen on CT scan.[10] Using TBI-CDE outcome measures examining multiple domains revealed that subjects with unfavorable outcomes could be separated into groups that screened positive for PTSD versus those with cognitive disability. While this improves precision of outcome assessment beyond GOSE, gaps in the TBI-CDE outcome battery were identified with respect to more severely injured patients, leading us to propose the flexible battery in this application. TRACK-TBI Pilot data also permitted comparison of emergency department disposition decisions in mild TBI patients. Co-I Adeoye et al[11] found that although hospital admission usually resulted after a finding of CT abnormalities, no difference in 6-month outcome was observed regardless of whether the patient was admitted to hospital. Site-specific factors also influenced disposition, highlighting the opportunity to examine the variability in TBI clinical practice that influences outcome and costs.

These results demonstrate the significance of a fully integrated TBI Information Commons that will:

- Improve TBI classification/taxonomy for targeted clinical treatment trials.
- Improve TBI outcome assessments, such that the size and costs of clinical trials can be reduced.
- Identify the health and economic impact of mild TBI patient disposition.
- Create a legacy database with analytic tools and resources to support TBI research.

3. STRUCTURE AND GOVERNANCE

The TRACK-TBI team, in collaboration with expert public-private partners, in a Team Science approach, is now prepared to enroll 3668 subjects across 18 sites. Shattering the silos that traditionally separate research and clinical practices, our team includes acute-care clinicians, neuroscientists, rehabilitation specialists, health economists, and informaticists. The effort and resources of these highly qualified investigators, combined with those of our government and industry collaborators, have created a uniquely synergistic public-private partnership. The TBI Information Commons will create a high quality, widely accessible, and fully integrated TBI-CDE dataset compatible with FITBIR and the International TBI Research Initiative (InTBIR). Collaboration within InTBIR will provide opportunities to strengthen research approaches, and importantly, provide a global platform to connect TBI's best scientists. The expected outcome is an international resource, statistically powered to identify new diagnostic and prognostic markers and refine outcome assessments, which will lead to successful clinical treatment trials.

TRACK-TBI's multiple-PI governance champions inclusiveness, collaboration, excellence, and efficiency. The PIs will share strategic planning, management, and oversight, advised by a Steering Committee, with day-to-day functions managed by the Contact PI and an Executive Committee. An innovative, distributed core model empowers our researchers to generate and execute initiatives. A nimble administration will assure coordination, oversee fiscal matters, and maximize synergies through public-private partnerships.

3.1 STEERING COMMITTEE

The Contact PI will serve as first Chair of the Steering Committee (chair will thereafter rotate) and will be the NIH's primary point of contact for the project. The PIs bear ultimate responsibility for the project, working collaboratively with and advised by, a Steering Committee composed of the PIs (who also lead our Cores in most cases, described below), Core Leaders,18 Study Site Leaders, and 3 international members. The Steering Committee is charged with strategic planning for research, and overseeing site operations and personnel. The Steering Committee will make policy decisions related to key operations. The Steering Committee's Data Use Committee will serve as arbiter for the use of TRACK-TBI's data and biospecimens, according to the Data Use Policy and IP Agreements conforming to FITBIR policies. As well, it will synthesize contributions of the Scientific and Community Advisory Boards to strategic research participation and planning. The Steering Committee will meet via weekly teleconferences, as well as at periodic national/international meetings.

3.2 EXECUTIVE COMMITTEE (EC)

The EC, composed of the PIs, with a rotating member of the Steering Committee (initial member Dr. Pancioli), will have oversight of day-to-day TRACK-TBI operations. The EC will be led by the Contact PI and supported by the Administrative Core. The Contact PI will coordinate communication among PIs, including establishing meeting schedules and agendas. The EC will make and execute final decisions as to research priorities,

supervise execution of studies, identify external collaborative private and public partners, ensure sound fiscal management, and review financial resource allocation. It will monitor data quality and progress toward completion of the Specific Aims through its Data Acquisition and Quality Committee (DAQC). The EC will also participate in InTBIR's International Scientific Advisory Committee (ISAC). Upon notification of award, the EC will meet in-person, followed by weekly teleconferences during which PIs will provide updates on progress toward launch. Post-launch, the EC will monitor Core functions with ongoing review of enrollment targets and compliance with all clinical, data management, and regulatory/ethical protocols. Should key personnel need to be replaced, the EC will make recommendations to the Contact PI, who will immediately alert the NIH.

3.3 INNOVATIVE DISTRIBUTED CORES MODEL

Administrative Core (AC). Dr. Manley and his administrative staff will have overall scientific and financial responsibility. The AC will oversee subcontracts, prepare/monitor budgets, report to NIH Program staff regarding timeline and milestone achievement, submit progress reports, and plan/facilitate meetings and teleconferences, including distributing and archiving agendas and minutes.

Clinical Core (CC). Drs. Robertson and Okonkwo will lead the CC, responsible for direct oversight of the clinical research sites, reporting to the EC. The CC will develop the recruitment plan and organize training meetings as well as approve the design of electronic Case Report Forms (eCRFs) and the Manual of Operations and Procedures (MOP). It will approve regulatory documents, track enrollment, and organize the collection of clinical and biomarker data. Detailed CRFs can be found in Appendix 3.

Biospecimens Core (BC). Dr. Diaz-Arrastia will lead the BC Core, responsible for direct oversight of protocols for biospecimen sample collection and transport to the central biorepository at UP for storage and analysis. The BC will have primary oversight of Specific Aim 2, Subaims 2.2 and 2.3. Detailed protocols and training procedures can be found in Appendix 4.

Informatics Core (IC). Dr. Toga will lead the IC, which will oversee TRACK-TBI Information Commons data flow. Responsibilities of the IC include the integration and harmonization of the multimodal data collected across each Core, ensuring that data comply with NINDS/FITBIR standards, and managing the timely transfer of data between the TRACK-TBI Information Commons hub and the FITBIR and InTBIR Platforms (tranSMART, One Mind/INCF portal, LONI). Co-Leader Kevin Smith will manage the tranSMART and One Mind/INCF portal. The IC will also deploy critical imaging and data processing resources rapidly to our clinical and research collaborators and to the public.

Neuroimaging Core (NC). The NC, led by Dr. Mukherjee, is responsible for standardization of imaging data acquisition, imaging quality, and analytics (Specific Aim 1; Specific Aim 2, Subaim 2.1). The NC will provide coding and adjudication for all TRACK-TBI neuroimages according to the NIH-CDE criteria. Detailed CT and MR imaging protocols can be found in Appendix 5.

Outcomes Core (OC). Dr. Giacino will lead the OC, responsible for design, collection, and evaluation of a flexible outcome assessment battery comprised of TBI-CDEs that capture a broad range of outcomes at all levels of TBI severity and across all phases of recovery. The OC will oversee site training for collection of outcome data, and have primary protocol oversight for Specific Aim 3. Detailed outcomes administration protocols and the full measures can be found in Appendix 6.

Biostatistical and Comparative Effectiveness Research Core (BCERC). Co-led by Dr. Temkin (Biostatistics) and Dr. Goldman (CER), the BCERC is responsible for design and analysis of data captured across all Aims. Dr. Temkin will coordinate biostatistical analyses for all Aims, and Dr. Goldman will analyze the direct impact of treatments, tests, and practice patterns using health economic and clinical metrics (Specific Aim 4).

3.4 PUBLIC-PRIVATE PARTNERSHIP RESOURCES

Both financial and in-kind resources will be provided through public-private partnerships supporting computer infrastructure, diagnostics, data management, and informatics, through such partners as One Mind for Research (OMFR), QuesGen Systems, Laboratory of NeuroImaging (LONI), GE, Siemens, Banyan Biomarkers, Myriad RBM, DePuy Synthes, a Johnson&Johnson company, Abbott Laboratories; tranSMART Foundation, and Thomson Reuters, in close collaboration with the University of Michigan, Neuroscience Information Framework (NIF), International Neuroinformatics Coordinating Facility (INCF), American College of Radiology Imaging Network (ACRIN), and FITBIR. By leveraging existing data management, biospecimen management (UCSF), image management and informatics initiatives (QuesGen, LONI, tranSMART, OMFR Portal, FITBIR), the TBI Information Commons ensures the reliable, harmonized, and secure collection and curation, followed by dissemination, of TBI-CDE-compliant heterogeneous data, enabling integration and analysis at cell, organ, and systems levels.

3.5 SCIENTIFIC ADVISORY BOARD (SAB)

The SAB is composed of distinguished individuals with expertise in the fields of: Neurosurgery and TBI, Alzheimer's Disease, Genetics, Proteomics/Systems Biology, Imaging/Outcomes, Translational Science, Information Technology/Computer Science, Precision Medicine, Quality CER, and TBI Philanthropy. The SAB will advise on strategies for new research priorities, and identify complementary funding sources. It will meet yearly, or more frequently by request, with circulated agendas and minutes.

3.6 COMMUNITY ADVISORY BOARD (CAB)

Our multi-stakeholder CAB is the essential ambassador to community engagement for participation in research. Their efforts in community sensitization will result in TRACK-TBI achieving timely accrual targets, retaining study subjects, and designing and facilitating appropriate dissemination routes for research findings. With the CAB, we will evolve the design of our Web portal, using the ONE MIND/INCF Platform. Our CAB members are leaders of the CDC, One Mind for Research, the NCAA, and the Brain Injury Association of America. The CAB will teleconference, according to circulated agendas, documented with minutes.

4. OBJECTIVES AND SPECIFIC AIMS

Effective treatment of traumatic brain injury (TBI) remains one of the greatest unmet needs in public health. After 3 decades of failed clinical trials, a new approach is needed. Our proposal establishes a public-private partnership of experienced TBI investigators, and philanthropic and industry collaborators, who share a mission to accelerate TBI research. TRACK-TBI will create a large, high quality database that integrates clinical, imaging, proteomic, genomic, and outcome biomarkers to establish more precise methods for TBI diagnosis and prognosis, refine outcome assessment, and compare the effectiveness and costs of TBI care.

We hypothesize that this approach will permit investigators to better characterize and stratify patients, allow meaningful comparisons of treatments and outcomes, and improve the next generation of clinical trials. We have built on the TRACK-TBI Pilot study (NCT01565551) and our team's precompetitive collaboration, forged by participation in the TBI Common Data Elements project (TBI-CDE) and InTBIRHaving provided the index dataset for FITBIR, we now propose the following Specific Aims:

4.1 SPECIFIC AIM 1

To create a widely accessible, comprehensive TBI Information Commons that integrates clinical, imaging, proteomic, genomic, and outcome biomarkers from subjects across the age and injury spectra, and provides analytic tools and resources to support TBI research.

Multi-disciplinary teams across 18 sites will enroll 3668 subjects of all ages across the injury spectrum of concussion to coma. The existing TRACK-TBI Pilot informatics platform will be expanded to facilitate clinical trial management, analytics, CER, and data sharing. Features of the Information Commons include integrated data and biospecimen management, as well as neuroimaging and informatics platforms, to support the entire information lifecycle (collection, management, curation, analysis, and sharing) accessible via a web portal to facilitate internal project communication as well as external community engagement. Curation in conformance with FITBIR policies ensures that collected data can be integrated and aggregated with InTBIR-affiliated projects and other NIH and DOD studies. Data will be transported into FITBIR, yielding an open-source resource for current and future TBI research and international collaboration.

Data Analysis. We propose various statistical methods to address our Specific Aims, and the data will be openly available to test hypotheses not yet formulated. With a fixed sample size of 3000 per the NINDS RFA, analyses should consider the magnitude of effect sizes that can be detected or the precision of parameter estimates possible for any proposed hypothesis. For context, comparing 2 groups in the full cohort using a t-test, the detectable effect sizes are < 0.18 (small). For comparing binary outcomes such as mortality, this translates to a difference of < 9 percentage points. For the CA Cohort alone, detectable effect sizes are < 0.24 (small), and for the CA + MRI/CA+MRI-HDFT Cohort, they are < 0.39 (medium). We will implement a best practice approach to statistical analyses where the analysis plan for each question is defined a priori, including specification of the target participants. Where data mining is proposed, adjustments for multiple comparisons will be made.

4.2 SPECIFIC AIM 2

To validate imaging, proteomic, and genetic biomarkers that will improve classification of TBI, permit appropriate selection and stratification of patients for clinical trials, and contribute to the development of a new taxonomy for TBI. We hypothesize that validated imaging, proteomic, and genetic biomarkers will permit improved patient classification, beyond traditional categories of mild, moderate and severe TBI.

CT and MR imaging are universally used to assess brain disorders. However, the full clinical significance of imaging abnormalities has not been firmly established for TBI.[13] We have recently demonstrated the utility of qualitative TBI-CDE imaging biomarkers for prognosis in mild TBI,[9] but their prognostic significance in more severe TBI and long-term outcome remains unknown. Quantitative imaging biomarkers have the potential to further improve diagnosis and prognosis. These include computer-aided lesion analysis, regional brain

volumetrics, white matter microstructure and structural connectivity from diffusion tensor imaging (DTI), and functional connectivity (FC) from resting state functional MRI (R-fMRI). We have developed novel software that performs immediate quantitative, objective measurements of key parameters of the admission head CT.[19] We have demonstrated that these quantitative CT (qCT) parameters are superior to qualitative patho-anatomic features for prediction of 6-month GOSE.[20] Quantitative MRI measurements of regional brain volumes and cortical thickness are useful biomarkers in large multicenter studies of neurodegenerative disease such as Alzheimer's disease.[21] We have previously described progressive atrophy of the hippocampus and amygdala during the 1-12 month interval after TBI,[22] but large-scale studies are needed to determine whether regional brain volume and cortical thickness changes are related to cognitive and behavioral impairments. Given the reported association of both hippocampal and amygdala volumes with PTSD,[23-26] we aim to validate these regional volumetric measurements as biomarkers of PTSD. The large sample size and longitudinal design of the proposed study will help resolve the controversy over whether atrophy of the hippocampus and amygdala leads to PTSD or if pre-existing small hippocampal and amygdala volumes predispose to PTSD.[26] Numerous single-center studies using DTI demonstrate that injury to the microstructural integrity of white matter tracts can explain much of the cognitive and behavioral sequelae of TBI.[27] We have shown that the global load of microstructural injury on DTI is associated with impaired cognitive processing speed,28 and that microstructural integrity of the uncinate fasciculus (UF) correlates with memory performance while microstructural integrity of the anterior corona radiata (ACR) is related to visuospatial attention.[29] This is especially significant because processing speed impacts many areas of cognition, while memory and attention are the 2 cognitive domains most often impaired in mild TBI.[30,31] Our preliminary data also reveal that early reduction in white matter microstructural integrity correlates with poor verbal memory at 12-month follow-up. Disruption of the structural connectivity of the brain also leads to alterations in its functional connectivity, defined as the temporal synchrony of neural signals such as the blood oxygenation level-dependent effect in R-fMRI. We recently analyzed R-fMRI in a cohort of 51 mild TBI patients at 1-month post-injury and compared them with 45 matched controls. Altered FC of mild TBI patients versus controls was found bilaterally in the default mode network (DMN), executive control network (ECN), and the salience network.[32] Although R-fMRI results from single-center TBI studies are promising,[33-40] large, multicenter validation studies are needed.

Validated proteomic biomarkers have been critical to progress in a broad range of clinical conditions; their absence in the TBI field is a major limitation. The most widely studied blood-based biomarkers are neuron-specific enolase (NSE), glial protein S100a, myelin basic protein (MBP), glial fibrillary acidic protein (GFAP), and ubiquitin-C-terminal hydrolase 1 (UCHL1).[41-46] Recent work from the TRACK-TBI Pilot confirms that GFAP provides information regarding CT abnormalities and persistent disability after TBI.[10] Elevation of additional biomarkers has been identified, including mediators of the innate immune response,[47,48] coagulation, and endothelial activation.[49] Inflammatory biomarkers are associated with cerebral hypoperfusion[47] and elevations in intracranial pressure, making them attractive surrogate markers for therapies such as hypertonic saline.[49] Another emerging area is autoimmune response biomarkers. Brain-directed autoimmunity is a well-established pathogenic phenomenon in neurological disorders such as paraneoplastic syndrome, Alzheimer's disease, stroke, epilepsy, and spinal cord injury.[50-55] In TBI, autoimmunity has been examined in a limited way and focused on autoantibodies against preselected antigens such as MBP, S100a, glutamate receptors, and pituitary proteins.[56-61] Our recent analysis of biospecimens from TRACK-TBI Pilot found significantly elevated anti-GFAP antibody in patients with a TBI history. This is an appealing marker due its subacute nature and ability to provide an objective marker for prior TBI, a negative predictor for outcome.

Recent work from the Human Genome Project has made feasible a new approach to identify potential therapeutic targets in human TBI. "Mendelian randomization" takes advantage of natural variability in outcome after similar traumatic insults.[62] Allelic association analysis is used to determine whether inheritance of certain genetic variants results in worse functional outcome. To date, published allelic association studies have implicated common variants in approximately 20 genes that play a role in outcome after TBI. While these studies are promising, all have small to modest sample sizes, and findings have been independently reproduced for only 3 genes (APOE, BDNF, and ANKK1—the latter from a single laboratory). Genome-wide

levels of significance have not been achieved, raising the likelihood of false positives. Sufficiently powered studies are required to confirm allelic association between gene variants and TBI outcome, which would be powerful evidence of the role of certain molecular pathways in TBI pathophysiology in humans.[62]

Subaim 2.1. To establish prognostic imaging biomarkers for TBI based on pathoanatomic analysis of CT and MRI, quantitative MR volumetrics, diffusion tensor imaging (DTI), and resting state functional MRI (RfMRI).

- **Hypothesis 2.1.1.** Patho-anatomic analysis of early CT and MRI scans performed by a neuroradiologist using CDE criteria are predictive of poor outcome at 6 months after TBI, as measured by GOSE and domain scores (or pediatric equivalents) (Aim 3.1). Moreover, quantitative analysis of these scans to measure mass effect, hematoma volume, and edema volume further improves outcome prediction at 6 months.
- **Hypothesis 2.1.2.** Post-traumatic cerebral atrophy, detected using quantitative volumetrics of serial MRI, is associated with global outcome after TBI, as measured by GOSE and domain scores. Regional atrophy in the prefrontal cortex will be associated with executive function, while hippocampal and amygdala atrophy will both be associated with PTSD. However, baseline hippocampal and amygdala volumes at 2 weeks after TBI will not predict development of PTSD, supporting the view that post-traumatic atrophy leads to PTSD.
- **Hypothesis 2.1.3.** DTI measures of fractional anisotropy (FA) in the corpus callosum at 2 weeks after TBI predict cognitive processing speed and global outcome at 6 months (measured by GOSE and domain scores). Additionally, FA of UF and ACR at 2 weeks predict memory and attention deficits at 6 months.
- **Hypothesis 2.1.4.** Reduced R-fMRI FC of the DMN, and increased FC of the salience network, at 2 weeks after TBI predict worse global outcome at 6 months as measured by GOSE and domain scores (Aim 3.1).

Subaim 2.2. To identify blood-based biomarkers that will provide additional diagnostic and prognostic information with which to identify TBI phenotypes that can be targeted by specific therapies.

- **Hypothesis 2.2.1.** A combination of blood-based proteomic biomarkers measured within the first day of injury (GFAP-BDP, UCH-L1, and S100B) are predictive of intracranial pathology on CT and MRI scans with a high level of sensitivity and specificity (AUC > 0.8), and are predictive of unfavorable outcome at 6 months.
- **Hypothesis 2.2.2.** Persistent elevation in the subacute and chronic period of a subset of inflammatory and systemic response biomarkers in blood is associated with unfavorable outcome 6 months after injury.
- **Hypothesis 2.2.3.** Blood levels of anti-GFAP-BDP or anti-pituitary 16K antigen auto-antibody in the chronic stage after injury are associated with unfavorable outcome.

Subaim 2.3. To identify common polymorphisms in candidate genes associated with outcome after TBI, and to elucidate causal molecular mechanisms of injury, response, and repair.

- **Hypothesis 2.3.** APOE, BDNF, and ANKK1 variants will be associated with poor outcome after TBI with genome-wide level of significance ($p \leq 10^{-7}$).

Subaim 2.4. To construct a multidimensional TBI classification system incorporating data from multiple domains that will define homogeneous classes of patients suitable for clinical trial inclusion.

- **Hypothesis 2.4.** A new taxonomy of TBI that includes imaging, proteomic and genetic data will be more precise than the GCS-based categories of mild, moderate, and severe TBI.

Methods - Neuroimaging. Pathoanatomical analyses of scans will be performed according to TBI-CDE for neuroimaging[63] on scans from all sites by Co-I Dr. Yuh, a board-certified neuroradiologist, who will be blinded to all data. A second neuroradiologist, PI Dr. Mukherjee, will read a subset of the CT and MR scans to establish inter-rater reliability of the pathoanatomic interpretations. We will apply quantitative CT (qCT) methods and innovative new software developed by a corporate partner (GE Global Research) for computer-aided analysis of lesions on structural MRI.[19,20] Volumes from cortical and subcortical structures and cortical thicknesses will be measured for all of the regions of the Desikan-Killiany atlas[64] from FreeSurfer analysis of 3D T1-weighted images.[65] Parametric maps of fractional anisotropy (FA), mean diffusivity (MD), axial diffusivity (AD), and radial diffusivity (RD) will be computed for each scan and then registered to the FMRIB58 template in the standard MNI1[52] atlas space using the automated tract-based spatial statistics (TBSS) processing pipeline.[66] Regional measurements of DTI parameters will be made in 30 major white matter tracts throughout the brain using

pre-defined regions of interest from the Johns Hopkins University White Matter Atlas.[67] After motion correction, bandpass temporal filtering, smoothing, regression of nuisance signals, and registration to MNI1[52] atlas space, seed voxel correlation analysis of FC in the DMN, ECN, and salience network will be performed on the R-fMRI acquisition, and regional cortical and subcortical FC measurements will be obtained.[32] All quantitative data from computer-aided lesion analysis, volumetrics, DTI, and R-fMRI measurements will be uploaded as metadata for each scan to the TRACK-TBI Information Commons.

Methods – Biospecimens. Blood specimens will be collected at specified time points and processed for plasma, serum, and DNA isolation according to TBI-CDE biospecimen protocol; these will be deposited in the TRACK-TBI Biospecimen Repository. Our corporate partner will assay the GFAP, UCHL1, and S100a biomarkers. We will use multiplex bead-array immunoassays to measure over 80 biomarkers of inflammation, innate immunity, coagulation, and endothelial activation. These studies will be performed in collaboration with our corporate partners who have pioneered multiplex bead-arrays immunoassays using the Luminex platform, and have been widely used in large clinical trials of neurodegenerative disease, cancer, and atherosclerosis. The HumanMap v.2.0 antigens panel contains most of the biomarkers that have been investigated in small TBI studies, as well as additional proteins that have been implicated in neuroprotection and neurorecovery after TBI. Co-I Dr. Wang will examine the prevalence and temporal profile of anti-GFAP and anti-pituitary antibody (17-kDa autoantigen) in acute and chronic time points after TBI. We will correlate autoantibody levels to prior history of TBI, inflammatory marker response, and to patient outcome.

Data Analysis. Subaims 2.1 to 2.3 will use a common statistical methodology. Regression methods appropriate to the outcome (e.g. logistic regression for binary outcomes) will be used to construct predictive models and to estimate which imaging parameters and biomarkers are most strongly associated with which outcomes, after accounting for the commonly available predictors such as GCS and those used in the CRASH[98] and IMPACT[99] models. Sensitivity, specificity, likelihood ratios, and corrections will be made for multiple comparisons. Area under the Receiver Operating Characteristic curve will be used to assess prognostic strength. To develop a TBI taxonomy (Subaim 2.4), we will initially use factor analysis methodology. Newer, unsupervised learning methods such as neural networks, clustering methods, or network topology will be explored to see if they provide a more useful classification.

Expected Outcomes. TRACK-TBI will validate these promising biomarkers in a large cohort of TBI patients. By combining conventional and state-of-the-art CT and MR neuroimaging techniques with information from proteomic biomarkers, genetic markers, and clinical parameters, we will construct a comprehensive, multidimensional classification of the traumatized brain across a wide spectrum of TBI severity and pathologic mechanisms. An important deliverable will be the creation of a carefully curated Biomarkers Repository which will be available to other investigators for discovery of novel proteomic, metabolomic, or genomic biomarkers. Our deliverable of a new TBI taxonomy squarely meets the NAS's challenge[100] to create large datasets that drive the development of disease taxonomy defined by intrinsic biology in addition to traditional physical "signs and symptoms."

Potential Limitations. Although we will adopt well-established and effective protocols from ADNI for harmonizing structural MRI across centers, such standards for DTI and R-fMRI are still in evolution. However, recent multicenter studies[101,102] have shown that adequate inter-site reproducibility is possible for both modalities and these standardization procedures. It is likely that useful biomarkers remain to be discovered. The aim of TRACK-TBI will be to overcome the limitations of previous studies by creating a large, carefully provenanced and curated Biomarkers Repository. However, we believe such discovery work is best supported through investigator-initiated mechanisms rather than through a multicenter cooperative agreement. If, by 2016, progress in the field indicates that novel biomarkers are more promising than those proposed here, the Steering Committee may consider a reallocation of resources to pursue assay of these new biomarkers.

4.3 SPECIFIC AIM 3

To evaluate a flexible outcome assessment battery in adult patients comprising a broad range of TBI-CDEs that enables assessment of multiple outcome domains across all phases of recovery and at all levels of TBI severity.

Measuring TBI outcome is important for clinical trials, prognostic studies, and longitudinal studies of recovery or deterioration. Outcome measures need to be sensitive to treatment effects, associated with prognostic variables, and be sufficiently specific to reflect small but important differences in TBI sequelae. TBI can affect physical, cognitive, emotional, and social domains of function. Cognitive impairment is the signature feature of TBI and is closely related to the biological severity of the brain injury. Because most measures of cognition require engagement in the testing process, the most impaired patients are typically excluded from formal assessment, significantly biasing study samples. The GOSE is the most commonly used outcome measure in adult TBI research, but its outcome categories are both broad and non-specific, with observed scores being affected by cognitive impairment, mental health disorders, and access to community resources. Data from the TRACK-TBI Pilot showed the association between GOSE outcome strata and impairment on measures assessing cognitive and psychological domains. Although cognitive dysfunction was clearly a more prevalent driver of impairment in patients with lower GOSE scores (3-4), psychological distress emerged as a key contributor to impairment in patients with higher GOSE scores (5-7). The CDEs cover a broad array of TBI consequences. We will test a flexible outcome assessment battery, comprised of CDEs, that is sensitive to injury-related changes in multiple domains of function and is suitable for assessment at all levels of TBI severity across all stages of recovery. We will also address the concern that most cognitive measures, including those recommended as CDEs, must be administered in-person, which increases both cost and loss to follow-up. The TRACK-TBI infrastructure affords the opportunity to explore the effectiveness of the Brief Test of Adult Cognition by Telephone (BTACT), a battery of tests for cognition that can be administered remotely but that has not yet been used extensively in TBI research. Using empirical data reduction techniques common in psychometrics, we will identify factors that emerge from the proposed outcome battery that are specific to the various outcome domains assessed. Increased sensitivity and specificity of these outcome measures, particularly at the upper and lower limits of injury severity, will provide more granular outcome data and support the design of more efficient studies with potentially smaller sample sizes. Moreover, relating the improved TBI classification system developed in Aim 2.4 to this multifactorial outcome battery will help identify patients most likely to benefit from specific interventions and enable better detection of treatment effects.

For pediatric patients, an outcome battery consistent of measures recommended by the Common Data Elements Pediatric Outcomes Group and the NIH Toolbox will be used and results merged with other injury and host factors by similar computational methodology used for adult patients, as noted below.

Subaim 3.1. To improve the granularity and breadth of TBI outcomes using a flexible outcome assessment battery that enables basic neurocognitive assessment in subjects too impaired to undergo standard neuropsychological testing, and comprehensive assessment of cognition, functional status, mental health, social participation, and quality of life in those cognitively intact enough to provide valid results.

- **Hypothesis 3.1.1.** In those too impaired for standard neuropsychological testing at 2 weeks post-injury, performance on the Coma Recovery Scale-Revised (CRS-R) and the Confusion Assessment Protocol Cognitive Impairment subscale (CAP CI) will be associated with cognitive function measured using the Comprehensive Assessment Battery at 6 months.
- **Hypothesis 3.1.2.** In persons who are oriented and able to undergo the Comprehensive Assessment Battery, outcomes will be multifactorial with at least one cognitive and one mental health factor.

Subaim 3.2. To determine the efficiency of a flexible outcome assessment battery, as compared with the GOSE, in reducing sample sizes needed to detect differences between groups.

- **Hypothesis 3.2.1.** The in-person flexible outcome battery will require less than half the sample size to have the same power as the GOSE in detecting expected differences between groups.

- **Hypothesis 3.2.2.** The telephone-administered battery will allow at least a 25% decrease in sample size, as compared with the GOSE, for detecting differences between groups.

Subaim 3.3. To identify specific TBI phenotypes amenable to targeted interventions, by relating patient classification factors (Subaim 2.4) to different outcome factor scores (Subaim 3.1).

- **Hypothesis 3.3.1.** MRI abnormalities in those with GCS 13-15 and the APOE allele will be most strongly associated with the cognitive outcome factor while the ANKK1 allele and loss of volume in the amygdala will be most strongly associated with the mental health factor.

Methods. The details of the outcome assessments will depend on the assessment that the subject is enrolled into (see Section 5.2 for more details). Subjects enrolled in the Comprehensive (CA) and Comprehensive with MRI (CA+MRI)/CA+MRI-HDFT Cohorts (n=1800) will be assessed with a battery that directly relates to the TBI-CDE v.2.0 Core, Basic, and Supplemental outcome measures (as published on the NINDS CDE Web site), at 2 weeks, 3 months, 6 months, and 12 months post-injury. The 2-week, 6-month, and 12-month assessments will be conducted in-person while the 3-month assessment will be administered by telephone. At 6 months, the CA Cohort will undergo telephone administration in addition to in-person administration of the outcome battery. The Comprehensive Assessment Battery includes measures of cognition (e.g. attention, memory, information processing speed, executive functions), mood, depression, anxiety, posttraumatic stress symptoms, functional status, social participation, and subjective well-being. Subjects enrolled in the “Brief Assessment” Cohort (BA Cohort) (n=1200) will undergo telephone-based evaluation on the GOSE at 2 weeks, 3, 6, and 12 months post-injury in accord with measures in the TBI-CDE v.2.0 Core. The CA Cohort will include 300 matched controls with extracranial injuries who will be assessed in the same manner and on the same schedule as the subjects with TBI. To improve sensitivity to the full range of TBI sequelae we have incorporated additional measures designed to extend the evaluation to the lower and upper levels of function. For subjects unable to complete the Comprehensive Assessment Battery due to confusion or disturbance in consciousness, we will administer an abbreviated battery comprised of the CRS-R and CAP CI, which will enable quantitative assessment of basic elements of cognition and behavior. Subjects who are sufficiently cognitively intact will receive both performance-based cognitive assessment and self-report questionnaires addressing psychological health.

Data Analysis. Subaim 3.1.1. We will define categories for those unable to complete the Comprehensive Assessment Battery by using the CAP Cognitive Impairment score. If the CAP CI score is <18, or cannot be obtained, outcome categories will be based on the CRS-R score. This will provide an ordered cognitive outcome for those too impaired to undergo the Comprehensive Assessment Battery. We will use Spearman rho to assess the strength of association with cognitive scores at 6 months, when most will be able to take the full battery. **Subaim 3.1.2.** Subjects who complete the Comprehensive Assessment Battery will be included in a principal components analysis (PCA). There must generally be about 10 cases for each variable included in a PCA; with well over 1,000 cases having taken the Comprehensive Assessment Battery we will be able to include results from each test in the battery. Varimax rotation will be used to minimize the number of variables loading on a factor and maximize interpretability of factors. Factors with an eigenvalue >1 will be interpretable as a domain of interest. **Subaim 3.2.** Effect sizes, defined as a difference in means divided by the common standard deviation, will be calculated for the comparison between various groups (i.e., GCS 3-8 [severe TBI] vs. GCS 13-15 [mild TBI] and GCS 3-8 vs. controls) that would be expected to differ on particular factor scores (e.g. cognition). Effect sizes[18] will be calculated for the GOSE alone, the in-person cognitive factor score and the BTACT total score. If the effect size for the cognitive factor score is over 1.4 times that for the GOSE, using the factor score as an outcome in a trial would allow a 50% decrease in sample size compared to using GOSE. Similarly, an effect size 1.15 times greater allows a 25% decrease. **Subaim 3.3**. We will use regression methods to predict outcomes from the patient classifications identified in **Subaim 2.4**. We will also explore other novel methods, such as neural networks, machine learning, and structural equation modeling.

Expected Outcomes. A validated flexible outcome battery comprised of a broad range of CDEs staged to estimate level of functioning in different domains will provide a platform to obtain both cross-sectional and

longitudinal data across the entire spectrum of TBI severity. The sensitive and reliable factor scores will allow clinical trials with greatly reduced sample sizes. A valid telephone-based assessment of cognition will improve efficiency for long-term follow-up. Coupling a TBI injury classification system to a multi-domain outcome assessment battery will provide a rich description of TBI sequelae, identify distinct TBI phenotypes, and facilitate development of more targeted and efficient clinical trials.

Potential Limitations. Successful completion of this Aim depends on the capacity of the measures selected to capture domain-specific functional status and subjects' willingness to comply with a comprehensive assessment battery that requires serial in-person assessments during the first year post-injury. Although in-person assessment is difficult and costly, consortium members have demonstrated the ability to attain high follow-up rates under these circumstances and both consortium commitment and the capitated payments will facilitate successful completion of this Aim. This effort also informs future parallel efforts for pediatric patients.

4.4 SPECIFIC AIM 4

To determine which tests, treatments, and services are effective and appropriate for which TBI patients, and use this evidence to recommend practices that offer the best value.

Little is known about the relative effectiveness or cost implications of different approaches to managing TBI, and practice variability is extensive. Capturing data on tests, treatments, and services with associated health outcomes and costs provides a unique opportunity to answer questions about which practices improve which patient's outcomes and at what cost.

One controversial example is deciding whether ED patients with mild TBI should be discharged or admitted and, if admitted, to what level of care. Prior work from TRACK-TBI Pilot demonstrated inconsistency in ED disposition between and within sites, even after adjusting for severity.[11] The lack of consistency in disposition, and uncertain association between disposition and outcome show that current risk-stratification tools are inadequate. This has implications for costs, as unnecessary treatments contribute to ED backlogs and improper resource allocation.[103-105] A related example arises after discharge from the ED. It is unknown how follow up to identify TBI's treatable sequelae may impact patient outcomes. Yet another example is the variability in treatment decisions for the increasing number of elderly patients who are taking antiplatelet agents at the time of TBI. Antiplatelet therapy may affect the progression of intracranial hemorrhage and increase the risk of morbidity and mortality in TBI patients. Many clinicians order platelet transfusion to reverse antiplatelet therapy, however it is unknown whether transfusion results in better (or even improved) TBI outcomes.[106]

Specific Aim 4 will identify which patients benefit from costly, often invasive care, and those who may be safely and effectively managed with less intensive options. We will use health economics and CER methods to evaluate which clinical practices improve both short- and long-term clinical outcomes and quality of life, limit unnecessary costs, and improve economic outcomes such as productivity (e.g. return to work vs. absenteeism). We will apply these to the 3 clinical examples described above, all of which lack an evidence base, span the ED, ICU, and follow-up settings, and illustrate how this dataset can improve the effectiveness of treatment and management of TBI across the care continuum.

Subaim 4.1. To identify patients currently admitted to an ICU who could be safely and effectively cared for in a floor bed or discharged home with outpatient management, and to estimate the health and economic impact of changing the management of these patients.

- **Hypothesis 4.1.1.** Among patients with mild TBI, there are factors measurable at ED presentation that characterize those not at risk of deterioration, who may be admitted to a lower level of care, such as a floor bed or observation unit, and those who may be safely discharged.
- **Hypothesis 4.1.2.** Changing the ED disposition from ICU to floor or from floor to discharge reduces overall health care costs without worsening patient health or economic outcomes.

Subaim 4.2. To determine whether routine follow-up improves TBI outcomes and minimizes economic burden.

- **Hypothesis 4.2.1.** Patients discharged from the ED who are followed up to identify treatable sequelae of TBI have improved health outcomes compared with those who are not followed up.
- **Hypothesis 4.2.2.** The costs of follow up are lower than the economic losses arising from failure to identify and mitigate adverse sequelae of TBI.

Subaim 4.3. To assess variability in management of patients taking antiplatelet agents at the time of TBI, and the effect of management on progression of intracranial hemorrhage, need for craniotomy, and outcome.

- **Hypothesis 4.3.1.** Patients taking antiplatelet agents at the time of injury are at increased risk of developing intracranial hematoma requiring neurosurgical intervention compared with those not on antiplatelet agents.
- **Hypothesis 4.3.2.** Platelet transfusion for patients taking antiplatelet agents at the time of injury does not reduce the risk of developing an intracranial hematoma or improve TBI outcomes.

Methods. Different questions are relevant to different patients: Subaim 4.1 will include patients presenting to an ED with mild TBI (GCS 13-15); Subaim 4.2 will include patients discharged from the ED; Subaim 4.3 will include patients on antiplatelet agents at time of injury, and suitably matched controls who were not on antiplatelet agents pre-injury. When appropriate we will pool data with the InTBIR project. We will measure the relative benefits of alternative approaches to health care delivery with multivariate analysis. Let Y_i represent some health or economic outcome of a TBI patient i treated at site s. We will relate outcomes to treatment using the following Equation:

$$Y_{is} = \delta_s + \pi T_{is} + \omega_X X_{it} + \epsilon_{ijst}$$

Here, T_{is} is a measure of the treatment of interest and the parameter $\boldsymbol{\pi}$ indicates the impact of that treatment on outcomes. If we hypothesize that treatment T improves outcomes, we would expect $\pi \geq 0$. Other covariates are site fixed-effects δ_s that capture potentially confounding time-invariant heterogeneity across sites, and individual patient characteristics X_{it}. These will include demographics as well as detailed clinical, biomarker, and imaging information that could be correlated with health or socioeconomic status.

This model generalizes to many different specifications. Outcomes in TBI can be binary, categorical, or continuous, and statistical models will be selected appropriately (including linear regression, logistic regression, or survival analysis). These can include acute (e.g., ICH expansion) or chronic (e.g., depression) outcomes, which we will model separately, as acute outcomes usually require more intensive care. Beyond clinical outcomes, we will collect information on health and economic outcomes including mortality, readmission, health care costs, Quality-Adjusted Life Years (QALYs), and productivity.

A challenge to identifying the causal effect of health care is that when patients are assigned to treatment according to expected clinical benefit, estimates of treatment efficacy can be biased. For example, sicker patients are more likely to be admitted, so simply comparing outcomes between admitted and discharged patients could generate misleading results unless severity is considered. We will apply multiple techniques widely used in CER and health economics to identify the causal effect of different treatments on outcomes, namely propensity scoring, case/control matching, and instrumental variable analysis.

For **Subaim 4.1** in applying the Equation to the relationship between ED disposition and subsequent events we would treat T_{is} as a binary variable equal to 1 if the patient were admitted and 0 otherwise. If mild TBI patients were no worse off from discharge we would expect $\pi = 0$ if we used health measures as the outcome variable (Hypothesis 4.1.1) but we would expect $\pi < 0$ if total health care costs were the outcome (Hypothesis 4.1.2).

The causal pathway between patient characteristics and outcomes is confounded by the greater likelihood of admission for sicker patients. One approach to addressing this is a 2-stage matching estimator. First, we will develop propensity scores, which predict the probability that a patient is discharged. Then we will ascertain whether discharge impacts the probability of an adverse outcome adjusted for propensity. Thus, we compare outcomes between admitted and discharged patients who are otherwise similar at the time of disposition.
To model the cost effectiveness of ED disposition decisions, we will compute the overall health care costs, productivity loss (or gain), and the QALYs associated with various decision-making thresholds. We will use estimates of the value of a QALY from the economics literature to determine how the relative value of different disposition options—ranging from discharge to ICU admission—changes as the probability of adverse outcome increases. We expect that the final predictive models, combined with evaluation of economic impact, will inform the construction of decision aids that can guide best practices for disposition decision making.

For **Subaims 4.2 and 4.3 w**e can apply the same approach to our other clinical applications, using T_{is} as a measure of follow up (4.2) or prior antiplatelet use, with or without platelet transfusion (4.3). Importantly, we can use models with different specifications for treatment, and/or include different treatment combinations in the same model, and evaluate which are most strongly associated with outcomes. For example, the timing, frequency, and specialty involved in follow up may impact health and economic outcomes differently. International comparisons will provide added variation in practice to inform modeling; both the care and outcomes of TBI patients have been shown to differ significantly between the US and Europe.[107-108]

Expected Outcomes. We will show the direct impact of treatments, tests, and practice patterns on patients' health over short and longer time periods, and assess the implications for health care costs and other economic outcomes (e.g. productivity gains). This will inform medical decision-making and identify knowledge gaps where new approaches are needed to improve outcomes or contain costs. We will also assess the value of novel biological and imaging markers and treatment approaches developed during the course of this work. In addition, this approach will be used to begin to explore a number of widely recognized gaps in evidence related to optimizing current TBI management. By identifying which types of patients and injuries respond most to specific treatments with fewest risks, we can begin to fill these gaps. Examples include determining the relative risks and benefits of parenchymal monitors vs. external ventricular drains for patients with specific injury types, comparing different indications and agents for hyperosmolar therapy, and quantifying differences in recovery duration and follow up needs for pediatric patients compared with adults.

Potential Limitations. Observational cohort data must be treated carefully to allow causal relationships to be uncovered. We propose rigorous statistical techniques to account for such issues as confounding by severity or indication in order to identify causal effects. Nonetheless, the possibility remains that conclusions may be weakened if variables go unmeasured or heterogeneity is unexplained. This project will produce the most comprehensive TBI dataset ever available, and so we expect to mitigate such concerns.

We expect that achievement of these Specific Aims will advance our understanding of TBI, improve clinical trial design, lead to more effective patient-specific treatments, and improve outcome after TBI.

5. STUDY DESIGN AND SAMPLE SIZE

5.1 SUBJECT GROUPS

A total of 2,700 TBI patients will be enrolled evenly across 3 clinical groups, differentiated by clinical care path:

1. Patients evaluated in the ED and discharged (ED)
    a. After May 5, 2016 patients in this clinical care path will only be enrolled as controls and as TBI subjects at the sites participating in the HDFT imaging protocol.
2. Patients admitted to the hospital, but not to ICU (ADM)
3. Patients admitted to the ICU (ICU)

An additional 100 patients per clinical group (n=300) with extracranial trauma but no TBI will be enrolled as controls for a total enrollment of 3000 patients. This stratification plan into 3 clinical groups, developed and adopted by InTBIR working groups, facilitates CER analyses and is not constrained by traditional differentiation into "Mild/Moderate/Severe" TBI. Data collection is dependent on the clinical care path (ED, ADM, ICU) and requirements of each Aim. Patients in each group will be stratified into 3 cohorts that define the extent of data to be collected. Cohort distribution, data components, and time points appear in the Clinical Protocol Grid and Flexible Outcome Assessment Table.

The Controls will be adult orthopedic trauma patients who meet the following criteria:

1. An Abbreviated Injury Score of ≤4 (not life threatening) for their extremity and/or pelvis injury and/or rib fracture.
2. Meet the same inclusion and exclusion criteria as the TBI subjects (Section 6.1) except that the criterion of having undergone a CT or MRI in the ED for suspected head injury does not apply. TBI will be ruled out for the current injury by interviewing potential controls about LOC, disturbance of consciousness, and PTA/RA.
3. Each site will be provided a plan in Appendix 8 for the number of controls to target according to age and gender distributions derived from the TBI Cohort.
4. Controls will be enrolled into the CA-MRI cohort for follow-up (Section 5.4) and drop to CA at 2-weeks if unable to complete the MRI visit.

An additional 200 participants will be enrolled as Healthy Controls at select TRACK-TBI sites. These Healthy Controls will not contribute to the overall study enrollment projection as only a subset of TRACK-TBI data will be collected at one time point. Specifically, Healthy Controls will contribute a baseline blood sample, past injury history using the Ohio State University TBI Identification Method Interview Form, and past medical history. No follow ups will be conducted with Healthy Controls. Healthy Control blood samples will be analyzed and compared with blood samples collected from participants with TBI.

Healthy Controls will meet the following criteria:

Inclusion criteria:

1. Age 18-100
2. No history of traumatic brain injury or concussion <12 months ago
3. Ability to obtain informed consent

Exclusion criteria:

1. History of TBI <12 months ago
2. Any traumatic injury causing polytrauma in the last 12 months

In mid-July 2018, TRACK-TBI completed its projected enrollment of 3000 participants study-wide. Enrollment will continue in order to fulfill the enrollment goals of the sub-studies within TRACK-TBI (e.g., HDFT, SDII, Abbott, i-STAT, etc.). Therefore, we are increasing the overall enrollment projection to 3668 subjects. The additional 668 subjects to the overall enrollment projection will be enrolled across all 18 TRACK-TBI sites on a rolling basis.

5.2 ASSESSMENT COHORTS

1. Brief Assessment (BA) Cohort

- **n=1200, 400 each for ED, ADM, and ICU Groups**
- Demographic and full clinical course data
- Blood draw for serum, plasma, DNA and RNA on Day 1 (< 24 hours of injury)
- Repeat blood draw for serum within 3-6 hours of the Day 1 baseline collection (optional for sites to include this component)
- Clinical brain CT scan from Day 1 acquired as part of hospital course
- Outcome data collected via structured telephone interview at 2 weeks, 3, 6, and 12 months using NIH TBI-CDEs v.2.0 Core outcome measures as published on the NINDS CDE website

*Patients in the BA cohort will not be enrolled until a directive has been issued by the Executive Committee to the study sites.

2. Comprehensive Assessment (CA) Cohort

- **n=1200, 300 subjects + 100 controls each for ED, ADM, and ICU Groups**
- Demographic and full standard clinical course data
- High density daily clinical data for ADM and ICU Groups
- Blood draw for serum, plasma, RNA, and DNA on Day 1 (< 24 hours of injury)
- Repeat blood draw for serum and plasma within 3-6 hours of the Day 1 baseline collection (optional for sites to include this component)
- Blood draw for serum, plasma and RNA on Day 3 (48-72 hrs) and 5 (96-120 hrs) for ADM and ICU
- Collection of cerebrospinal fluid on days 1 through 5 (optional for sites to include this component)
- All clinical brain CT scans and MRIs acquired as part of hospital course
- Blood draw for serum, plasma and RNA at 2 weeks and 6 months
- Outcome data collected via structured in-person interview at 2 weeks, 6, and 12 months and at 3 months via structured telephone interview using NIH TBI-CDEs v.2.0 Core, Basic, and Supplemental outcome measures

3. Comprehensive Assessment + MRI (CA+MRI)/CA+MRI-HDFT Cohort

- **n=600, 200 each for ED, ADM, and ICU Groups**
- Demographic and full standard clinical course data
- High density daily clinical data for ADM and ICU Groups
- Blood draw for serum, plasma, RNA, and DNA on Day 1 (< 24 hours of injury)
- Repeat blood draw for serum and plasma within 3-6 hours of the Day 1 baseline collection (optional for sites to include this component)
- Blood draw for serum, plasma, and RNA on Day 3 (48-72 hrs) and 5 (96-120 hrs) for ADM and ICU
- Collection of cerebrospinal fluid on days 1 through 5 (optional for sites to include this component)
- All clinical head CT scans and MRIs acquired as part of hospital course
- Blood draw for serum, plasma and RNA at 2 weeks and 6 months
- 3T research MRI acquired at 2 weeks and 6 months
- Outcome data collected via structured in-person interview at 2 weeks, 6, and 12 months and at 3 month via structured telephone interview using NIH TBI-CDEs v.2.0 Core, Basic, and Supplemental outcome measures

5.3 PATIENT COHORT SELECTION FOR MRI

The goal is to enroll a minimum of 600 patients in the Phase 1 CA+MRI cohort that have completed both the 2-week and 6-month visits. After that goal is met sites with a Siemens scanner participating in the high diffusion fiber tracking (HDFT) protocol will continue to enroll into this imaging Phase 2 protocol. Based on the TRACK-TBI Pilot study and others in the field, the 2-week MRI is a challenge for both the TBI patient schedule as well as hospital research resources including scheduling, personnel time, and resources. As we seek to enroll a total

of 1800 patients in the Comprehensive Assessment, an achievement of 33% with MRI completed at 2 weeks and 6 months was set as a feasible initial threshold. Given the resource value of the MRI and the known challenges for its completion all patients approached for the study will be enrolled initially into the CA+MRI/CA+MRI-HDFT group. Patients who are unable to complete the MRI at 2 weeks due to contraindications, scheduling, or loss to follow-up will be placed in the CA cohort with all follow-up timepoints identical to the CA+MRI cohort. Completion rates of 2-week and 6-month MRI will be assessed quarterly by the Clinical Core. Should TRACK-TBI be on a trajectory to exceed the initial threshold of 33% MRI completion rate, the Clinical Core will evaluate pacing, enrollment strategies, and potential resource needs for an increased total number of MRIs achieved, and report such recommendations to the Steering and Executive Committees for appropriate modifications to the enrollment target.

5.4 CLINICAL PROTOCOL GRID

BA* n=1200	CA n=1200	CA+MRI/ HDFT n=600	Procedure	Admission	Hospital	2W	3M**	6M	12M
**ED: ED Discharge Group (900 TBI patients + 100 controls)**									
♦	♦	♦	Admission Data	X					
♦	♦	♦	Blood (DNA, Biomarkers)	X (optional repeat @ 3-6h)					
	♦	♦	Blood (Biomarkers)			X		X	
♦	♦	♦	Clinical Brain CT (and MRI)	X					
		♦	3T Research Brain MRI			X		X	
♦			Outcomes: Telephone			X	X	X	X
	♦	♦	Outcomes: Full Battery			X	X	X	X
**ADM: Hospital Admit Group (900 TBI patients + 100 controls)**									
♦	♦	♦	Admission Data	X	X				
	♦	♦	Daily Clinical Data	X	X (daily)				
♦	♦	♦	Blood (DNA, Biomarkers)	X (optional repeat @ 3-6h)					
	♦	♦	Blood (Biomarkers)		X (day 3,5)	X		X	
♦	♦	♦	Clinical Brain CT (and MRI)	X	X (all)				
		♦	3T Research Brain MRI			X		X	
♦			Outcomes: Telephone			X	X	X	X
	♦	♦	Outcomes: Full Battery			X	X	X	X

BA* n=1200	CA n=1200	CA+MRI n=600	Procedure	Admission	Hospital	2W	3M**	6M	12M
**ICU: ICU Group (900 TBI patients + 100 controls)**									
♦	♦	♦	Admission Data	X	X				
	♦	♦	Daily Clinical Data	X	X (daily)				
	♦	♦	High Resolution ICU Data	X	X (daily)				
♦	♦	♦	Blood (DNA, Biomarkers)	X (optional repeat @ 3-6h)					
	♦	♦	Blood (Biomarkers)		X (day 3,5)	X		X	
	♦	♦	CSF (Biomarkers, optional)		X (days 1-7)				
♦	♦	♦	Clinical Brain CT (and MRI)	X	X (all)				
		♦	3T Research Brain MRI			X		X	
♦			Outcomes: Telephone			X	X	X	X
	♦	♦	Outcomes: Full Battery			X	X	X	X

*Patients in the BA cohort will not be enrolled until a directive has been issued by the Executive Committee to the study sites.

** For the CA and CA+MRI/CA+MRI-HDFT cohorts the 3M timepoint will only be by telephone. The 6M time point will include the BTACT.

5.5 MILESTONE PLAN

	Year 1			Year 2			Year 3			Year 4	Total
**Group**	**CA+MRI**	**CA**	**N**	**CA+MRI**	**CA**	**N**	**CA**	**BA**	**N**	**BA**	**N**
**ED**	150	87	237	50	58	108	155	100	255	300	900
**ADM**	150	87	237	50	58	108	155	100	255	300	900
**ICU**	150	87	237	50	58	108	155	100	255	300	900
**Controls**	0	99	99	0	66	66	135	0	135	0	300
**Total**	450	360	810	150	240	390	600	300	900	900	3000

Our target follow-up rate for the 12-month duration of the study is 80%. The Clinical Core will monitor this rate at the overall study level as well as the individual site level. While this rate is ambitious, it was achieved in the COBRIT trial, in which several TRACK-TBI sites participated (UW, UPMC, UTSW, MST, VCU) and this experience can be effectively translated to the wider TRACK-TBI effort. No age limits will be applied to the study. Women and minorities will be included. Assent for children will be documented as required by local institutions.

TRACK-TBI Milestone Plan completed in mid-July 2018. Overall enrollment projection will be increased to 3668 subjects to allow for continued enrollment into the TRACK-TBI sub-studies.

6. SUBJECT ELIGIBILITY

We will enroll adult patients of all ages presenting to the Emergency Department (ED) with a history of acute TBI as per American Congress of Rehabilitation Medicine (**ACRM) Criteria**, in which the patient has sustained a traumatically induced* physiological disruption of brain function, as manifested by ≥ one of the following:

- Any period of loss of consciousness (LOC)
- Any loss of memory for events (e.g. amnesia) immediately before or after the accident
- Any alteration of mental state at the time of the accident (feeling dazed, disoriented, and/or confused)
- Focal neurologic deficits that may or may not be permanent

* Traumatically induced includes the head being struck, the head striking an object, or the brain undergoing an acceleration/deceleration movement (e.g. whiplash) without direct external trauma to the head.

<u>Possible question for LOC</u>

Did you have a period of time after the event when *you* were completely unconscious. That means you had no ability to think, speak or move and were completely unaware of the world around you.

<u>Possible question for PTA</u>

Was there a period of time after the injury for which you have no memory? If so, how long did it take for your memory to return to normal or become consistent (e.g. who you saw, conversations, what you ate, etc. (Walk them through the post-injury events if and as necessary).

<u>Possible questions for alteration in consciousness (AOC):</u>

Right after the event, did you feel dazed or confused or in a fog? Did you have trouble knowing where you were or what happened to you? Did you keep asking the same question over and over? Did you insist you could do things that you could or should not?

<u>Suggested prioritization for LOC, and for more severe cases, AOC:</u>
1st- EMS run report
2nd- Witness report
3rd- ED records (if positive)
4th- Participant

<u>Suggested prioritization for PTA, for milder cases AOC:</u>
1st- Participant
2nd- EMS run report
3rd- Witness report
4th- ED or hospital records (if positive)

In general:

LOC: Subject's recall is unreliable unless the subject explicitly states that a witness informed the subject that the subject was knocked out cold.

PTA: Use the participant report for mild cases. Use EMS or hospital medical records for more severe cases

AOC: should be recorded as "positive" if present in any of these sources. Only record AOC as negative if available sources (especially the participant) state it did not occur.

6.1 INCLUSION/EXCLUSION CRITERIA

Criterion	Data Source	Comments
**Inclusion Criteria**		
1. Age 0-100	Chart	
2. Documented/verified TBI (ACRM Criteria)	Chart, Interview	
3. Injury occurred < 24 hours ago	Chart, Interview	
4. Acute brain CT for clinical care	Chart	Subject must have brain CT scan
5. Visual acuity/hearing adequate for testing	Chart, Interview	
6. Fluency in English or Spanish	Chart, Interview	Based on Test battery or personnel availability
7. Ability to provide informed consent	Interview	□ Patient □ Surrogate □ Waiver
**Exclusion Criteria**		
1. Significant polytrauma that would interfere with follow-up and outcome assessment	Chart	Significant body trauma may confound TBI outcomes testing.
2. Prisoners or patients in custody	Chart, Interview	
3. Pregnancy in female subjects	Chart, Interview	
4. Patients on psychiatric hold (e.g. 5150, 5250)	Chart	
5. Major debilitating baseline mental health disorders (e.g. schizophrenia or bipolar disorder) that would interfere with follow-up and the validity of outcome assessment	Chart, Interview	Debilitating psychiatric disorders can significantly impact the reliability of follow up and/or pose difficulties in attributing to index TBI.
6. Major debilitating neurological disease (e.g. stroke, CVA, dementia, tumor) impairing baseline awareness, cognition, or validity of follow-up and outcome assessment	Chart, Interview	Documented debilitating baseline cognitive impairment will confound outcome assessment in addition to not being fully consentable.
7. Significant history of pre-existing conditions that would interfere with follow-up and outcome assessment (e.g. substance abuse, alcoholism, HIV/AIDS, major transmittable diseases that may interfere with consent, end-stage cancers, learning disabilities, developmental disorders)	Chart, Interview	
8. Contraindications to MRI (for CA+MR/CA+MRI-HDFT cohort)	MRI Screening	
9. Low likelihood of follow-up (e.g. participant or family indicating low interest, residence in another state or country, homelessness or lack of reliable contacts	Interview	
10. Current participant in an interventional trial (e.g drug, device, behavioral)	Chart, Interview	Exception to co-enrollment exclusion is made for sites participating in Resuscitation Outcomes Consortium Prehospital Tranexamic Acid for TBI Study.
11. Penetrating TBI	Chart	
12. Spinal cord injury with ASIA score of C or worse	Chart	

7. SUBJECT PROCEDURES BY CORE

7.1 CLINICAL

The following broad categories of clinical data variable types will be collected from all enrolled patients through medical record and personal interview:

- Baseline demographics e.g. age, gender, race, ethnicity, handedness
- Baseline socioeconomics e.g. education, employment, living situation, types of support
- Baseline medical history by system including substance abuse and prior TBI, and medications
- Mechanism of injury, location, and surrounding circumstances
- Pre-hospital clinical course variables e.g. vital signs, transport times, GCS score
- Brain CT report including presence of skull fracture and intracranial abnormalities
- Emergency department clinical course e.g. vital signs, GCS, fluids, labs, toxicology, complications
- Hospital admission clinical course e.g. daily vital signs, GCS, fluids, labs, complications, medications*
- For ICU patients: continuous physiologic data from high resolution ICU monitors (e.g. Moberg monitors)
- Hospital surgeries and neuromonitoring
- Hospital daily therapeutic intensity level for ICU patients with neuromonitoring
- Admit and discharge dates and times throughout full clinical course
- Abbreviated Injury Scale (AIS) score and Injury Severity Score (ISS)
- Discharge destination and acute care outcome evaluation

*For non-TRACK-TBI participants (i.e., participants enrolled after the final TRACK-TBI subject) who are admitted to the hospital but do not fall within the TRACK-TBI "severe" criteria (see Appendix 11 "Guidance for continued TRACK-TBI sub-study enrollment/follow up after final TRACK-TBI enrollment" document in the Clinical Protocol folder on Dropbox for severity inclusion criteria), data entry will no longer include the following CRFs: Scheduled and Daily Meds, Vitals, and Labs. These CRFs will continue to be required for severe hospital and ICU admit participants.

7.2 BIOSPECIMENS

Patients from all cohorts (CA+MRI/CA+MRI-HDFT, CA, BA) will have up to 21.0 ml of blood drawn <24 hours of injury and a repeat sample of 18.0 ml obtained 3 to 6 hours after the initial blood draw.

- Tubes consist of 6.0 ml EDTA (plasma and DNA), 6.0 ml red top (serum), 2.5 ml Paxgene (RNA).
- CA and CA+MRI patients admitted to the hospital floor and ICU will have additional blood draws of 15.0 ml per day on days 3 and 5 to allow for analysis of serial biomarkers. For pediatric patients, weight-based blood volumes will be obtained.
- CA and CA+MRI patients will have 15.0 ml of blood drawn at the time of the 2-week and 6-month 3T research MRI to correlate biomarkers with neuroimaging results. For pediatric patients, weight-based blood volumes will be obtained.
- Whole blood will be processed for serum, plasma, RNA, and DNA. Serum and plasma will be stored in 500µl aliquots for future analyses.
- *This is a site-specific protocol item.* At some sites (subject to local IRB approval), cerebrospinal fluid (CSF) will be drawn for patients with ventricular catheters. The CSF collection protocol is detailed in Appendix F of the Biospecimens Full Protocol
- *This is a site-specific protocol item.* At some sites (subject to local IRB approval), in the case of death, the subject's next of kin will be contacted to request donation of the brain for banking, validation studies of imaging and biomarker findings, and further research. See Appendix 11.

7.3 NEUROIMAGING

CT or initial MRI will be obtained as part of clinical care. 3T MRI will be obtained at 2 weeks and 6 months from CA+MRI subjects only. All initial and follow-up brain CT scans, and any brain MRI scans that are done for clinical care and their reports will be collected. Images will be read and coded by the Neuroimaging Core radiologist in accordance to the Neuroimaging TBI-CDEs. Sites with a Siemens scanner participating in the HDFT protocol will enroll new TBI patients into the CA+MRI/CA+MRI-HDFT cohort. Non-HDFT sites will finish out their 6 month

imaging visits on the Phase 1 protocol. All control subjects will complete their 2 week and 6 month imaging visits on the Phase 1 protocol. Sites with the capability to collect the Phase 1 3T MRI on inpatients may continue to enroll into the CA+MRI cohort. The Phase 1 protocol can be completed at 2 weeks on patients who are still in the hospital who presented with a moderate-severe TBI (ED Arrival GCS 3-12). The follow up scan at 6 months will be on the Phase 1 protocol as well.

7.4 OUTCOMES

All outcome measures will be obtained from the patient, or if cognitively unable, the caregiver. The BA cohort will undergo telephone assessment with the GOSE and the phone battery at 2 weeks, 3, 6, and 12 months. The CA+MRI/CA+MRI-HDFT and CA cohorts will undergo in-person outcomes testing with the flexible battery at 2 weeks, 6 and 12 months, as well as the phone battery at 3 months. Pediatric patients will undergo comparable Pediatric Outcomes Battery (Appendix 6.2).

Flexible outcomes battery framework. The proposed flexible outcome assessment battery is designed to assess multiple outcome domains across all phases of recovery in patients at all levels of TBI severity. The battery comprises the original Core CDE measures (TBI-CDE Version 1.0) administered in the TRACK-TBI Pilot, and additional Basic and Supplemental CDEs (TBI-CDE Version 2.0) measures that further assess psychological health and cognition. The combined TRACK-TBI Pilot and additional supplemental measures from TBI-CDE Version 2.0 constitute the **Comprehensive Assessment Battery**. Patients who are too impaired to take the Comprehensive Assessment Battery will undergo assessment on the **Abbreviated Battery**, which consists of standardized measures of basic neurobehavioral (e.g. Coma Recovery Scale-Revised [CRSR]) and cognitive (e.g. Confusion Assessment Protocol [CAP]) function.

FLEXIBLE OUTCOMES BATTERY FRAMEWORK: DECISION WORKFLOW

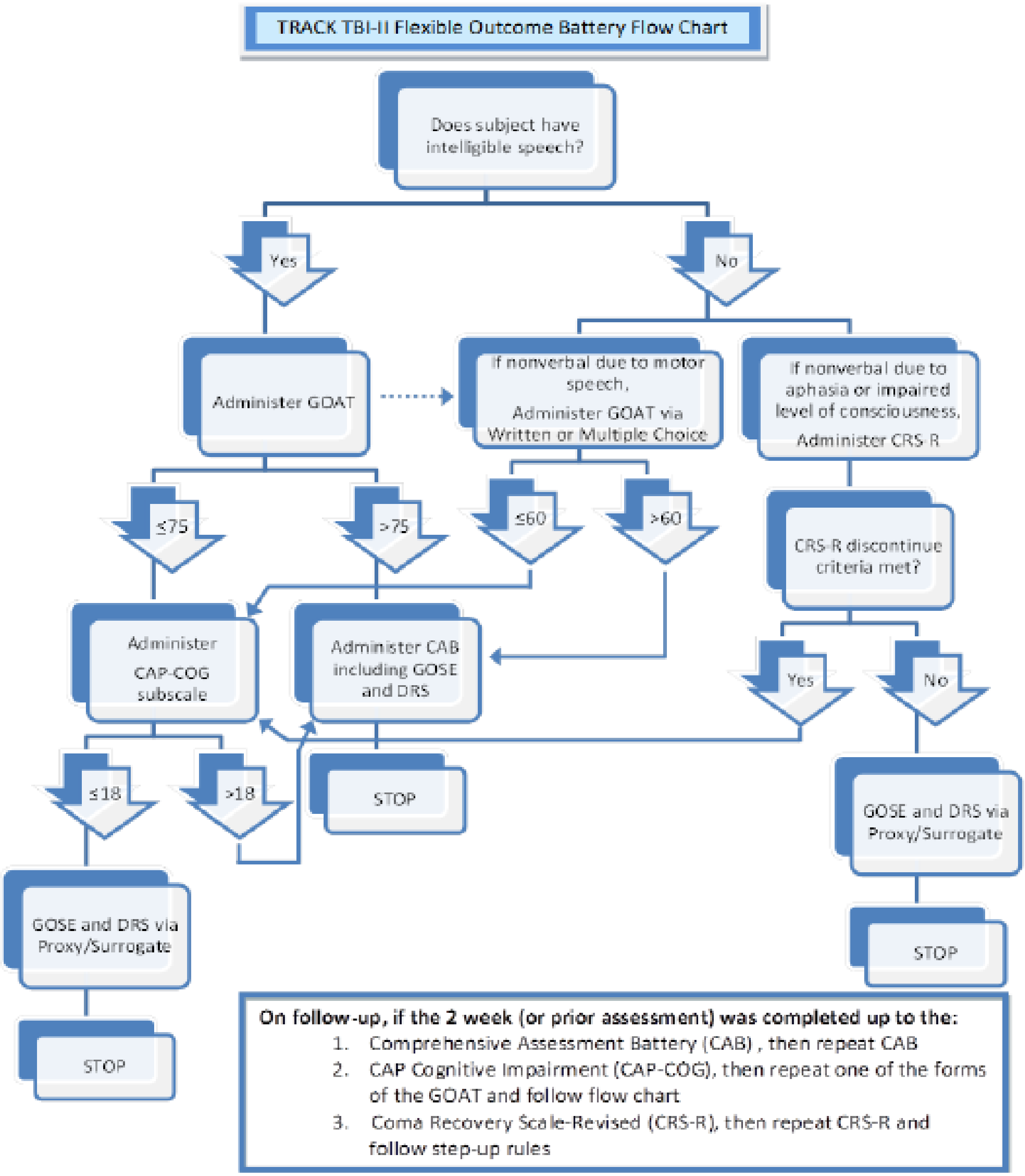

FLEXIBLE OUTCOMES BATTERY FRAMEWORK: MEASURES LIST

Domain	Outcome Measure	Estimated Completion Time	Comprehensive Assessment (CA) Cohort	Brief Assessment (BA) Cohort
**Screening Protocol (5-7 minutes)**				
Screening	• Assessment of speech intelligibility<br>• Galveston Orientation and Amnesia Test (Standard, Written, and Modified GOAT)	2m<br>5m	2W, then as needed	N/A
**Abbreviated Battery (60-85 minutes- includes screening)**				
Participant/ Surrogate Interviews	• Sections:<br>• Demographic Variables<br>• Vocational History<br>• Pre-morbid medical history<br>• Prior TBI screen<br>• Alcohol Use Disorders Identification Test (AUDIT-C)<br>• 3-Item Drug Use Interview	10 min	2W, 3M (T), 6M, 12M	N/A
Consciousness and Basic Cognition	• Confusion Assessment Protocol (CAP)<br>• Coma Recovery Scale Revised (CRS-R)	15m<br>15-30m	2W, 6M, 12M	N/A
Global Outcome	• Glasgow Outcome Scale Extended (GOSE)<br>• Expanded Disability Rating Scale Post-Acute Interview (E-DRS-PI)	8m<br>5-15m	2W, 3M (T), 6M, 12M	GOSE: 2W (T), 3M (T), 6M (T), 12M (T)
**Comprehensive Assessment Battery (136-148 minutes- includes screening; excludes BTACT)**				
Global Outcome	• Glasgow Outcome Scale Extended (GOSE)<br>• Expanded Disability Rating Scale Post-Acute Interview (E-DRS-PI)	8m<br>5-15m	2W, 3M (T), 6M, 12M	N/A
Participant/ Surrogate Interviews	• Sections:<br>• Demographic Variables<br>• Vocational History<br>• Pre-morbid medical history<br>• Prior TBI screen<br>• Alcohol Use Disorders Identification Test (AUDIT-C)<br>• 3-Item Drug Use Interview	15 min	2W, 3M (T), 6M, 12M	N/A
Cognition	• Rey Auditory Verbal Learning Test II (RAVLT)<br>• Trail Making Test (TMT)<br>• Wechsler Adult Intelligence Scale IV Processing Speed Index (WAIS-IV PSI)<br>• NIH Toolbox Cognitive Battery<br>• Brief Test of Adult Cognition by Telephone (BTACT)	15m<br>5m<br>4m<br>30m<br>20m	2W, 6M, 12M<br>------------------------<br>6M (T)	N/A
Post-Concussive/TBI-Related Symptoms	• Rivermead Post-Concussion Questionnaire (RPQ)<br>• Participant Reported Outcome Measurement Information System Pain Intensity and Interference Instruments (PROMIS-PAIN)<br>• Insomnia Severity Index	6m<br>5m<br>3m	2W, 3M (T), 6M, 12M	N/A
Participation and Quality of Life (QoL)	• Quality of Life After Brain Injury- Overall Scale (Qolibri-OS)<br>• Mayo-Portland Adaptability Inventory- (MPAI4-PART)<br>• Satisfaction With Life Scale (SWLS)<br>• SF-12 Version 2	5m<br>2m<br>3m<br>3m	2W, 3M (T), 6M, 12M	N/A
Psychological Health	• PTSD Checklist (PCL-5)<br>• Brief Symptom Inventory 18 (BSI18)<br>• Participant Health Questionnaire- 9 (PHQ-9)<br>• Columbia Suicide Severity Rating Scale (C-SSRS)*<br>(*Only required if ≥1 on the PHQ-9 or ≥2 on the BSI-18)	6m<br>6m<br>5m<br>5m	2W, 3M (T), 6M, 12M	N/A

Key: (T) = Testing conducted by telephone; (W) = Weeks; (M) = Months; 1 = measures translated into Spanish

8. SUBJECT RECRUITMENT AND SCREENING

8.1 SUBJECT IDENTIFICATION

Study personnel will identify potential subjects in the ED, hospital and ICU during "peak hours" as appropriate for their study site through conferring with medical records, trauma logs and triage notes as well as on-duty doctors and nurses to identify potential subjects. Many of the inclusion/exclusion criteria can be evaluated by a review of the potential subject's medical records, such as mechanism of injury, extent of non-head injuries, prior medical history, and prior clinical visits at the center of care. As all eligible patients must receive an acute clinical brain CT due to external force trauma to the head, the ideal place to begin screening is scanning for acute scheduled CT brain studies in the radiology department. When a potential subject is identified and has been screened against the primary set of inclusion/exclusion criteria, they will be approached about the study.

8.2 SCREENING PROCESS

Due to the vulnerability of the subjects and the complexity of the protocol, we envision a three stage screening process. These stages are: 1) review of medical records and test results to determine eligibility, 2) subject completion of a screening evaluation to determine competency to provide informed consent, and 3) subject interview to present and discuss this research participation opportunity. Only after all three of these phases have been completed would the subject be asked to participate and provide formal signed informed consent.

Prior to enrolling a subject, the research personnel will screen the subject for competency to provide informed consent. This is necessary because TBI may result in a period of posttraumatic amnesia (PTA) characterized by confusion, disorientation, and impaired memory for ongoing events. The Galveston Orientation and Amnesia Test (GOAT) will be used as the standard assessment instrument for this screening. A score of 75 or greater on the GOAT would indicate that the subject is competent to provide informed consent. If the subject scores < 75 on the GOAT, then consent must be provided by a Legally Authorized Representative (LAR).

To accomplish the competency evaluation part of the screening process, the research personnel will approach the subject and introduce the study, explaining that the subject may be a candidate but that additional information is required to determine this. It takes approximately 5 minutes for the subject to answer the test questions contained in the GOAT. The research personnel will then score the test as described. If the subject qualifies and wishes to proceed, then the research personnel will move to the third stage of this process. If the subject is not interested in participation in the protocol, the subject will be thanked for their time and the data collected up to this point will be shredded per standard hospital protocol.

8.3 PARTICIPATION REQUIREMENTS

An important part of the screening and process is an interview with the subject, where the research personnel explains the project in detail, presents the consent forms, and responds to all patient questions and concerns. Key points that will be explained during this interview process are:

- Participation in the project is immediate and for all components (clinical, biospecimens, MRI, outcomes), unless contraindicated for MRI.
- Upon enrollment, data collection will begin in the hospital. Participation in follow-up activities must be completed within given time windows as specified but will be scheduled to accommodate the patient:
    1) For CA+MRI/CA+MRI-HDFT patients, the 2-week MRI must be completed at 14 days ± 4 days from the date of injury. Corresponding 2-week outcomes must be completed ± 3 days of the 2-week MRI.
    2) For CA and BA patients, 2-week outcomes must be completed 14 day ± 4 days from date of injury
    3) Outcomes at 3 months must be completed ± 7 days of 90 days from the date of injury.
    4) For CA+MRI/CA+MRI-HDFT patients, MRI at 6 months must be completed ± 14 days of 180 days from the date of injury, with corresponding 6-month outcomes ± 14 days of the 6-month MRI.
    5) For CA and BA patients, 6-month outcomes must be completed ± 14 days of 180 days from the date of injury.
    6) BTACT should be completed within ± 7 days of Outcomes (but not on the same day).

7) Outcomes at 12 months must be completed ± 30 days of 360 days from the date of injury.

- Compensation is provided to cover expenses the subject may incur due to participation (e.g. tolls for travel and parking). In order to disburse these funds, the subject must provide a social security number or other identification for tax purposes.
- All efforts should be made to schedule patient return within the specified window for each timepoint. Patients who are reached and scheduled but fall outside the window for any outcomes testing timepoint should still have their outcomes assessment completed in person, or over the phone at 3 months (refer to Section 15.2 for guidance). The number of days from date of injury, and the number of days outside of the exact 2-week, 3-month, 6-month, and 12-month window will be documented in the QuesGen database. Potential subjects will be given time to read the Consent Form(s) and to consult with family members who may be present or by phone. If the subject agrees to participate, then they will sign the appropriate forms. A copy of the form(s) will be given to the subject.

8.4 SUBJECT COMPENSATION

In addition to incurred travel costs to arrive at the testing center, subjects in the CA and CA+MRI/CA+MRI-HDFT cohorts will receive financial compensation in recognition of the extensive in-person and/or phone time required by the study. Individual sites have the ability to determine their own reimbursement rate per timepoint as approved by local IRB. The suggested compensation schedule to be given as follows:

Amount	2W MRI (CA+MRI)	2W Outcome (CA, CA+MRI)	3M Telephone Outcome (CA, CA+MRI)	6M MRI (CA+MRI)	6M Outcome (CA, CA+MRI)	12M Outcome (CA, CA+MRI)
$125.00	X	X		X	X	X
$75.00			X			

For the optional protocol at sites that collect blood at 3 to 6 hours following the baseline blood draw it is suggested that subjects be compensated in the amount of $50.

For BA patients, all outcomes will be phone based. Outcomes at 2 weeks, 3 months, 6 months, and 12 months will each have a suggested reimbursement rate of $75.00. Compensation will be disbursed at the end of each visit. Subjects must provide a social security number or other form of tax identification to receive these funds.

9. INFORMED CONSENT

9.1 INFORMED CONSENT PERSONNEL

The individuals responsible for identifying potential subjects, explaining the studies, answering questions, and obtaining informed consent will be study research personnel who are healthcare professionals, including MD, RN, Research Coordinators, and Research Associates (RAs). Qualifications for these positions include clinical experience with TBI patients, patient teaching skills related to home medication administration, excellent interpersonal and problem-solving skills, and knowledge of the clinical research process.

Based on sites local IRB policies, sites may include language in their informed consent which will ask patients if they wish to be contacted for future research after the completion of this study.
Suggested language:
In the future, other studies involving traumatic brain injury may become available. If you agree, then someone from the [site name] Neurosurgery team may contact you in the future about additional research that you may be interested in participating in. You agree to allow someone to contact you about research in the future.
_yes _no

9.2 LOCATION AND PRIVACY

Potential subjects will be approached in the ED, hospital wards, or ICU at each IRB-approved enrollment site. All sites have implemented electronic medical records in their hospitals and much of the screening process can be completed via utilizing these resources. Interested subjects are offered the opportunity to participate on-site during their emergency hospital visits or contacted by phone after hospital discharge.

If potential subjects are approached in the ED, patient care areas can be screened with curtains for privacy. If potential subjects are approached while still in this area, the curtains will be closed and the conversation will be conducted in soft tones to minimize the possibility of being overheard. If the potential subject approaches their time of discharge, then the research personnel will escort the subject and family to one of the privacy areas available in the hospital after discharge from the ED to discuss the study and conduct the informed consent process. The approach to potential subjects in the ED will not be made in such a way that it interferes with or delays the diagnosis and treatment process in the ED. Potential subjects will be given as much time as needed to read the informed consent document, discuss it with family members if they choose, and to ask questions of the research personnel.

9.3 COMPETENCY SCREENING

TBI often results in a period of posttraumatic amnesia (PTA) characterized by confusion, disorientation, and impaired memory for ongoing events. Thus TBI patients will be screened for competency using the Galveston Orientation and Amnesia Test (GOAT) to determine whether they are competent to provide informed consent or whether this must be done by a Legally Authorized Representative (LAR). This competency screening will be performed prior to inviting the subject to participate in the study and while the subject is in the ED or hospital.

The procedure for this competency screening will be:

1. The subject's ED medical record will be reviewed to determine whether the subject has been diagnosed with post-traumatic amnesia or other cognitive deficits.
2. The subject and family if present will be approached and informed about the study.
3. If subject and family agree, the GOAT will be administered according to standard procedures.
4. If the subject scores ≥ 75 on the GOAT, the subject will be deemed competent to provide informed consent. If the subject scores <75 then informed consent must be provided by a LAR or waiver of consent (site specific).

9.4 LANGUAGE AND LITERACY

Subjects should be fluent in English to be eligible for the study. Sites with research personnel fluent in Spanish may elect to enroll patients who speak Spanish as their primary language. The informed consent documents are available in both English and Spanish. Patients not fluent in English, or Spanish at certain sites, will be ineligible for the study.

9.5 NEED FOR RECONSENT

As this is a longitudinal study with multiple timepoints over the course of a year, and that the status of TBI patient cognition may change over this time course, it is likely that subjects may not recall all of the activities or procedures associated with each follow-up visit. To ensure that subjects are still willing to participate, the research personnel will review the Informed Consent document with the subject at the beginning of each follow-up visit.

In the event that a subject was determined incompetent to sign their informed consent document (i.e. GOAT score < 75 at time of screening/consent) but later demonstrates competency, then the subject will be asked if they wish to continue participation. If so, then the subject will be asked to sign the Informed Consent at that time. If they decline to do so, then they will be withdrawn from the protocol.

9.6 STORAGE OF CONSENT DOCUMENTS

Signed paper consent forms will be stored in a locked file cabinet located in the study office behind locked doors at each site. These documents will be stored for a minimum of 3 years after the conclusion of the study. These documents will be made available as needed for review for quality monitoring purposes.

9.7 WAIVER OF CONSENT

This is a site-specific protocol item. Sites may elect to enroll qualifying patients initially incapable of informed consent who have no legally authorized representative available for surrogate consent. This can be done under a “waiver of consent” rule in the emergency setting in order to procure and process the baseline blood sample for biomarker analysis within 24 hours of injury. Application to use the “waiver of consent” can be submitted by each individual site to their local IRB for approval.

Sample language as follows:

“If the subject is not capable of self-consent and there are no legally authorized representatives to sign in person or via fax, every effort will be made to follow-through with the subject as soon as s/he is capable of informed consent. However, in these situations, and for those 18 years and older, the 'Waiver of Consent' will be used to complete the CRF, to draw the blood within 24 hours of injury and to process the blood for separation of plasma (within 30 minutes of draw) or serum (within 1 hour of draw) because of the critical acute time element involved.

These scenarios are very common in the context of TBI and in the past have prevented much needed data from being collected from patients who are incapacitated from their injury and hospitalization, and/or who are under the influence of drugs or alcohol. We would like to be able to include everyone falling under the inclusion criteria, hence the waiver.”

10. SUBJECT RISKS AND BENEFITS

10.1 FORESEEABLE RISKS BY CORE

The potential risks to the subject are minimal across all domains of data collection. No data collected as part of this study will become part of the subject's medical record.

Clinical. The TBI event will already be part of the subject's medical record, so involvement in this study will not have any effect on obtaining care or coverage under insurance. The risks involve some degree of loss of privacy. This will be minimized as much as possible. All data will be confidential and stored in locked areas to which only authorized study personnel have access. Records will be coded with a Study ID as soon as the patient is enrolled so that names and other identifying information will not be linked to personal or sensitive data, in compliance with federal regulations of the Health Insurance Portability and Accountability Act (HIPAA). This Study ID is automatically generated by the QuesGen System as soon as the patient is entered into the database. In addition, subjects and their families will be informed that participation is completely voluntary, that they may decline response to any questions, and that they may withdraw from the study at any time, all without jeopardizing medical treatment to which they are otherwise entitled. Subjects and their families will not be required to answer any interview or assessment questions that they find distressing or sensitive in nature.

Biospecimens. The blood sample will be drawn from an arterial or central venous catheter placed as a part of standard care for those patients consented while in the ICU. Those patients consented on the ward or emergency room will need to undergo phlebotomy and may experience the discomfort associated with a needle stick and may suffer bruising at the site of the needle stick. The risks involve some degree of loss of privacy. This will be minimized as much as possible. No more than two venipuncture attempts will take place.

Genetic Research. There is a possibility that if the results of a research study involving genetic material were to become generally known this information could affect one's ability to be insured, employed, future decisions regarding children, or family relationships. As noted, all data will be de-identified and linked by Study ID. Data will be stored in locked areas to which only authorized study personnel have access.

Neuroimaging. CA+MRI subjects will undergo noninvasive brain imaging using FDA-approved 3 Tesla MR scanners at 2 weeks and 6 months post-injury. No exogenous contrast agents and no sedation will be used. The MRI procedures are noninvasive and painless. The MRI does, however, require the subject to lie still with the head and part of the body confined in a tunnel-like device for a considerable length of time (total scan time of approximately 60 minutes). The subject may find it uncomfortable to lie still in the MRI scanner for 1 hour. Therefore the subject will have breaks in between the data collection periods to reset the scanner and set up the parameters for the next set of images being collected, and also will have frequent communication with the experimenter. Contraindications for the MRI examination include those who have cardiac pacemakers, neural pacemakers, surgical clips in the brain or blood vessels, surgically implanted steel plates, screws or pins, cochlear implants, intrauterine devices, or metal objects in their body, especially in the eye. Subjects will be required to remove all ferromagnetic items (e.g. keys, phones, credit cards, belts, loose change, and others) before entering the MRI examination room. Claustrophobia may also preclude successful MR imaging. Careful screening will prevent such individuals from participating in this study, as well as preventing the introduction of any ferromagnetic objects into the scanner room. Dental fillings do not present a problem.

The FDA has set recommendations for exposure in MRI studies and the proposed 3T examinations satisfy those criteria. The guidelines from the Bureau of Radiological Health of the FDA will be followed in regard to specific absorption rate (SAR) of radiofrequency energy and time varying magnetic fields (dB/dt). Precautions will be maintained so that SAR will be less than 8 watts per kilogram in any 1 gram of tissue. This is the estimated power required to raise the temperature 1 degree centigrade. The maximum dB/dt will be set at 20T/sec for > 120usec or 200T/sec for < 12usec. These levels are well below peripheral nerve stimulation threshold in

humans, both children and adults. In rare cases, subjects may still experience some peripheral nerve stimulation during portions of the MRI procedure. These experiences are transient and harmless. MRI participants will be instructed prior to examination to refrain from skin-on-skin contact of their extremities (e.g. clasping hands or legs) to further reduce this risk. The MRI will produce loud noises during image acquisition. The decibel intensity of these noises is not considered harmful per FDA regulations. Subject will be provided with earplugs and noise-cancelling headphones/earpads to minimize discomfort.

Subjects will always be in communication with the MRI technologist and will be given a squeeze ball that triggers an alarm. If the subject indicates at any point that they have a desire to stop the procedure, the exam will be terminated immediately and without any penalties to the subject in any way.

If any unexpected findings are identified that may be clinically significant, the participant will be counseled by the Site PI and recommended to seek medical care from their primary care physician. Subjects and their families will be informed that participation is completely voluntary and that they may withdraw from the study at any time, all without jeopardizing medical treatment to which they are otherwise entitled.

Outcomes. Some of the questionnaires and interviews used in this research ask about personal and potentially sensitive information. This will be explained to subjects both orally and in the consent document. Further, only trained study personnel who are sensitive to these issues will administer such interviews and questionnaires. If a subject endorses any of the questions regarding suicide, it will be documented in the research record and the research personnel will notify the psychiatrist on call and follow their instructions.

10.2 PROTECTIONS AGAINST SUBJECT RISKS

Recruitment and Informed Consent. All study sites are experienced with recruiting TBI subjects. All sites have obtained IRB approval to enroll patients into TRACK-TBI. Research staff will locate eligible patients in the hospital (emergency department, hospital wards, intensive care unit), explain the research study, review the consent form, ask the subject or surrogate if s/he voluntarily agrees to participate, and obtain consent. Prospective subjects will be given as much time as needed to consider study participation. If the subject is not capable of self-consent, all efforts will be made to locate a legal surrogate to sign in person or via fax.

Clinical. The potential risk to subjects is minimal. We will take all necessary steps to reduce risk for all study participants. We will inform subjects of the potentially sensitive nature of some of the research questions and create an atmosphere of security and trust prior to collecting data. We carefully explain the steps taken to assure the confidentiality of all participant data. Subjects are always given permission to not answer questions with which they feel uncomfortable. In our experience with TRACK-TBI Pilot, with the establishment of rapport by a sensitive, experienced research team the majority of subjects welcome the opportunity to participate in research. Following initial consent, subjects will be reminded before every procedure that participation is completely voluntary, that they may decline to respond to any questions, and that they may withdraw from the study at any time without jeopardizing medical treatment to which they are otherwise entitled.

To protect confidentiality, no paper copies of study forms will include subjects' names, but instead will contain a Study ID as the identification key to match subjects over the repeated measures. The subject names will be entered into the QuesGen web-based eCRFs in order for study personnel to contact patients and conduct follow-up visits. However, only designated study site personnel will be able to view the subject name fields in QuesGen. All data communication between the QuesGen browser and secure servers is through an encrypted secure socket layer connection. Servers are located in a Statement on Auditing Standards-70 compliant data center behind a dedicated firewall. QuesGen has procedures in place for full compliance with Health Insurance Portability and Accountability Act security standards for protection of PHI. User password accounts are assigned according to user types and access roles which allow or restrict the viewing of any PHI fields. An algorithm is applied to each data element to determine if it should be considered PHI. The default determinations can be overridden if incorrectly classified as PHI. Administrative users can set up accounts for

users to only view the data or set filters that limit viewing of records according to their study site. Every data modification is tracked and all views and deletions are logged so that data tampering is not possible. Study sites that are not covered entities by their institution will be required to establish Business Associate Agreements with QuesGen Systems, Inc.

Study data will be entered into eCRFs using designated laptop and desktop study computers with secure, encrypted connections to the eCRF data. No PHI will be stored on the hard drives of any study computers. Study computers will have encrypted drives conforming to IT standards at each respective site. Study computers will be password-protected and securely maintained with virus protection software installed to automatically update and scan the drives. Only research personnel responsible for data entry or review will have password access for study computers. Paper copies of surveys will be filed by their Study ID in locked file cabinets behind locked doors at each study site. A list linking the Study ID and names will be kept in a separate locked file cabinet behind locked doors at each study site. Once the final outcome assessments are completed and checking for data quality monitoring purposes is completed, PHI for the subject will no longer be accessible to previously authorized personnel except the study site PI. At project conclusion, PHI data will be stored in a password protected PDF file and given to the site PI for long-term protected storage. The QuesGen Data Manager will then remove the PHI data from all server hard drives and all backup devices. QuesGen will not retain any copies of the PHI data long-term. These identifiers will then be accessible only by the study site PI and would be used at a later time only if it becomes necessary to contact the subject for additional studies or for regulatory purposes.

Biospecimens. For the blood draw, no more than two venipuncture attempts will be performed and specimens will be coded when the draw is complete.

Neuroimaging. As noted, the total scan time is approximately 60 minutes, but the subject will have breaks in between the collection periods as the radiologist resets the scanner and sets up the parameters for the next set of image sets being collected, and also will have frequent communication with the experimenter. Some patients find the loudness of the oscillating gradients during image acquisition to be discomforting, but the acoustical noise level is below FDA guidelines of 140 dB peak referenced to 20 micropascals. In addition all patients are provided with earplugs to reduce the noise. There is no evidence for long-term negative effects of MRI procedures on the body. The 3T scanner is an FDA-approved system.

Outcomes. An experienced outcomes team designated by the Outcomes Core will train all outcomes personnel for TRACK-TBI. Only trained study personnel sensitive to the inherent issues across cognitive, mental health, psychological, and quality of life domains will administer the outcome instruments for the study. Subjects will be re-introduced to the study at each outcomes time point for understanding and approval to continue participation. Subjects are informed that they are free to not answer any question that may be uncomfortable for them. If a subject endorses any of the questions regarding suicide, it will be documented in the research record and the research personnel will notify the Site PI and activate local suicidality protocols.

10.3 CERTIFICATE OF CONFIDENTIALITY

As an additional level of safeguard for study participants, we have received a Certificate of Confidentiality from the NIH for TRACK-TBI. Having this Certificate means that investigators and study personnel cannot be forced to disclose research information that might identify the subject in any federal, state, or local criminal, civil, administrative, legislative, or other proceedings. This is important because TBI patients are often involved in high-risk behaviors that result in their injuries.

10.4 POTENTIAL BENEFITS OF PROPOSED RESEARCH

There is no direct benefit to study participants. The results will be directly relevant to society in general and to future patients who suffer TBI. Tokens of thanks for study participants are especially important in longitudinal studies, where the burden on the respondent, even if small, tends to multiply over time. Each site will establish

a reimbursement schedule for each of the time-consuming components of the study, including MRI, telephone and in-person outcomes testing. Patient transportation costs incurred during commute to the research site will be reimbursed by standard mileage rates, or via taxi vouchers.

TRACK-TBI subjects will undergo extensive neuropsychological testing and brain imaging. These procedures are not part of the standard of care for mild TBI.

- All subjects will have access to the results of their research MRI results within 2 weeks of their respective 2-week and 6-month timepoints. If requested, subjects will receive a CD of their conventional MR imaging data and a viewer application tool. The study ID will be stripped from the MRI scan.

Release of outcomes testing results is a site-by-site issue to be addressed in accordance with local IRB and Risk Management policies with the following guidance:

- Information will be released only to the subject or the guardian
- Information will be released in the form of raw data with the name of the measure and the score without any interpretation
- A disclaimer statement must be included in the released records (i.e. "*These data are not meant to replace diagnostic testing/evaluation that would be ordered by a personal physician. We cannot interpret the data and provide recommendations as the data we collect is meant for research purposes only.")*
- Test record sheets should not be released under any circumstances (risk of copyright violation and test invalidation)
- Upon request, sites that agree to provide results to subjects can do so after completion of their 12-month outcomes, as to minimize the feedback and undue influence of test results on the subjective perception from the research subjects during the study.
- All participants may share their study information with their care providers or others as they choose. Investigators will also be available for consultation with subject's care providers to interpret these findings with the subject. All outcomes data provided to subjects will be stripped of Study ID.

11. SUBJECT COMPLIANCE AND RETENTION

We will monitor subject compliance with the observational portion of the protocol, and research personnel will maintain scheduled contact with the subjects and their family members to ensure on-going compliance through the 12-month study duration. Upon consent and enrollment, participants will be asked to provide multiple forms of contact information including phone, address, and email. Upon their permission, participants will be asked to provide one or more alternate contacts. The Clinical Core will maintain a schedule of contact to maximize the chance of successful contact and scheduling for follow-up timepoints as soon as their window of return opens for that timepoint. This will involve training Research Coordinators to monitor their own site progress, which will be supplemented by automated reminders of upcoming windows for follow-up generated by QuesGen Systems and emailed to each site coordinator weekly. Every effort will be made to schedule return visits at the subject's convenience, and multiple procedures for that time point will be scheduled on the same day. When subjects return for their follow-up they will be met by research personnel who will escort them to the various testing locations. Subjects will have full opportunity to ask any questions before, during, and at the end of the follow-up appointment. Site Coordinators will be encouraged to have relevant resource packets and materials for TBI assembled to provide to study patients.

In the event that a TRACK-TBI CA+MRI/CA+MRI-HDFT subject does not or cannot return for the 2-week MRI, every effort should be made to schedule participation in the in-person CA visits for outcomes and blood draws.

- Subjects initially enrolled into the TRACK-TBI CA+MRI/CA+MRI-HDFT cohort that do not complete the MRI but are able to complete the 2-week in-person outcomes assessment and blood draw will be reassigned to the CA cohort as their final cohort at 2-weeks and this will be documented in the QuesGen patient management form. The cohort does not change after 2-weeks.
- If the 2-week MRI is missed, subjects will not be asked to come back for a 6-month MRI.
- If the CA subject who has completed the 2-week visit doesn't or can't return for the 6 and 12-months visits then always attempt telephone interview with those tests that are suitable.
- If a CA subject can't be scheduled for the 2-week in-person visit then an attempt should be made to obtain the telephone assessment battery and the subject will be assigned to the CA cohort as their final cohort at 2-weeks and this will be documented in the QuesGen patient management form. The cohort does not change after 2-weeks.
- If a subject does not complete the 2-week in-person or telephone CA visit then the final cohort at 2-weeks remains as CA and it's reported as a missed milestone.
- If a CA subject misses the 2-week appointment entirely, the subject should still be contacted for their 3-month follow-up and a protocol deviation will be recorded for the 2-week timepoint. If 2-week/3-month/6-month or any combination of these visits are "Missed Milestone" then study staff will continue to attempt contact through the end of the 12 months. In the event that the subject has never shown up for in-person or via telephone for 2-week, 3-month 6-month or 12-month follow-up but self presents and contacts the study coordinator during the 3, 6 or 12-month windows then attempt to obtain the GOSE and then as much as the interview as possible over the phone.
- Assignment to the BA cohort is not being used for enrollment or as a drop-down at 2 weeks. Assignment to the BA cohort will be upon notification to sites by the Executive Committee at such time that a site has fulfilled their CA-MRI and CA enrollment quotas during the study year. It is possible that sites completing the CA-MRI and CA quotas will be asked to do additional CA-MRI or CA enrollments if other sites are not meeting the quotas.

In the even that a non-TRACK-TBI (i.e., participants enrolled after the final TRACK-TBI subject) CA+MRI-HDFT subject does not or cannot return for the 2-week MRI, but the subject is enrolled in one or more of the TRACK-TBI sub-studies (i.e., SDII, Abbott, i-STAT), the subject will drop to the CA cohort and every effort should be made to schedule participation in the in-person CA visits for outcomes and blood draws. If dropped to CA, please see above for directions on how to follow-up.

In the event that a non-TRACK-TBI CA+MRI-HDFT subject does not or cannot return for the 2-week MRI, and the subject is ***not*** enrolled in any of the TRACK-TBI sub-studies (i.e., SDII, Abbott, i-STAT), the subject will be withdrawn from the study by the Principal Investigator and will receive compensation only for the study activities in which they participate before being withdrawn. (see Appendix 11 "Guidance for continued TRACK-TBI sub-study enrollment/follow up after final TRACK-TBI enrollment" document in the Clinical Protocol folder on Dropbox).

12. DATA MANAGEMENT AND COMPLIANCE

Clinical monitoring. Data collection for each timepoint and Core type must be completed accurately and to schedule. Clinical monitoring will utilize both field monitoring and in-house monitoring/Quality Control (QC) staff at the site level to optimize efficiencies and reduce data discrepancies. Furthermore the Data Acquisition and Quality Committee (DAQC) under the Executive Committee will conduct site visits to monitor protocol compliance, complete written monitoring reports, and deliver findings to the Executive Committee. Visits will be performed at a minimum of 1 visit at each site per year, per Core and protocol (clinical, biospecimen, neuroimaging, and outcomes). Protocol processes, including enrollment practices, data collection, imaging, and biospecimens collection procedures, will be assessed over the course of the study period. Site visits will be conducted to review and adjudicate subject records in accordance with TRACK-TBI monitoring procedures. Some records will receive a targeted review that may include items such as informed consents, eligibility, inclusion/exclusion criteria, adverse events, serious adverse events, key safety and efficacy parameters, protocol-specific biospecimens collection, neuroimaging procedures, and outcomes administration and scoring standards. The DAQC will review results from monitoring visits and regularly scheduled data checks to identify trends and problems, and will share these results with the Executive Committee on a regular basis.

12.1 CLINICAL

Clinical database. Upon study enrollment, the subject will be entered onto the QuesGen System which will automatically generate a Study ID. This Study ID is not generated from personally identifiable information (PII), and is generated locally so that no PII is ever sent to FITBIR in the process. Study IDs will be coded in the format of [Site Code (2 digits)] – [Patient Number (4 digits)], e.g. XX-XXXX, with Site Codes from 01 to 18 for the 18 study sites, and beginning at Patient Number 1001 for the first patient enrolled, 1002 for the second patient enrolled, and increasing sequentially for each additional patient enrolled. MRI scans will have an additional label at the end to distinguish the timepoint and whether the scan is a patient, phantom, or control. The additional labels are as follows: (XX-XXXX-1 for 2-week MRI, -2 for 6-month MRI). Clinical data will be entered into eCRFs via a web-based portal to the secure, fully HIPAA-compliant QuesGen clinical database.

Site Codes are as follows:

Site Name	Site Code
BCM-TIRR /UTHSC	01
MGH-Spaulding	02
UCSF	03
Univ. of Cincinnati	04
Univ. of Maryland	05
Univ. of Miami	06
Univ. of Pittsburgh	07
Seton / UT Austin	08
UT Southwestern	09
Univ. of Washington	10
VCU	11
Univ. Pennsylvania	12
Emory	13
Medical College Wisconsin	14
Univ. Utah	15
Indiana Univ.	16
Hennepin	17
Univ. Colorado	18

Automated data integrity monitoring. All clinical data will be entered into electronic Case Report Forms (eCRFs) and managed by the QuesGen data management platform. As data is entered into each form, the system will run data validation checks that include conditionally required data, validation across fields, and validation requirements based on subject type. If any validation check fails, the user is alerted immediately that the data does not meet QA criteria and the issue can be addressed and corrected at that point. If a data element fails a validation check, yet the value entered is correct, the user can enter an exception to the problem and provide a notation as to why the out-of-range data is actually correct. These data validation checks match the FITBIR validation protocol:

- **Date/time value checks:** all dates and times entered into the database are checked to ensure that events recorded are accurate and in sequence.
- **Range value checks:** all numeric, non-date fields have range values specified to minimize data entry errors.
- **Selection lists:** all categorical data fields have predetermined drop-down lists, check boxes, or resettable radio buttons instead of free text to ensure accuracy.
- **Logic checks:** data fields from different sections of the eCRF will be compared to pass logical integrity
- **Required fields:** the eCRF will be programmed to require input into fields when appropriate to minimize missing information.
- **Score calculation** will be performed and programmed into eCRFs for tests and measures with numerical score summations or norming to avoid mathematical errors by the examiner. All automated scoring

computations will be fully documented and validated by QuesGen and the Clinical Core, and must pass User Acceptance Testing.

- **Electronic data audits** will be automated in the QuesGen database through a series of pre-determined queries against the study database at regular intervals. These queries will be designed for the Clinical Core to monitor data quality and completeness and identify protocol variations/deviations/violations.
- **Data audits against source documents**, where available, will be conducted prior to the final "lock" of each subject's data set. Errors found will be corrected at this time.

All investigators and designated study personnel will have unique and confidential password access to the QuesGen database. All access to the database and to study data will be logged in an audit trail and monitored. Any indication of inappropriate access will be reported immediately to the Clinical Core. Investigators may submit data request for access of specific variables to the Clinical Core for approval.

The QuesGen system will also provide checks for form completion based on the subject type. Validation rules will establish when forms for a particular subject should be entered, and any missing forms can be tracked by the Study Site and Clinical Core immediate follow-up. Once subject forms are marked complete, a dataset for sharing can be created. The QuesGen platform stores the exact dataset that is shared for future reference and also tracks information about when the data was shared and the dataset recipient.

Due dates for eCRF completion windows are set by the Clinical Core. Due dates for eCRF completion windows are set by the Clinical Core. The Subject and Presentation eCRFs need to be initiated as soon as possible following enrollment in order to assign subject IDs for the biospecimen vials. In general, every effort should be made to complete eCRFs within 2 business days of enrollments, inpatient stays, follow-up milestones and discharges. It is understood that some forms and fields within forms may not yet have complete information available to report (e.g., Hospital Admission/Discharge, AIS/ISS, Surgeries, Concomitant Medications, etc.). The QuesGen System will automatically generate reminders to complete eCRFs for enrolled patients. Monthly reports of enrollment, timeliness of eCRF completion and error correction will be monitored and adjudicated by the Clinical Core. Data validated and curated within QuesGen will be transferred to LONI for aggregation and harmonization and uploaded to tranSMART weekly, to support quality assurance activities and data analysis, as well as to LONI IDA. Data will be transmitted to FITBIR quarterly, per FITBIR policy.

Integration with analytics platforms. All de-identified electronic study data in the TRACK-TBI database will be maintained in secure storage by QuesGen Systems for the duration of subject enrollment and follow-up and for a period afterwards for data analysis and preparation of publications. We estimate that the analysis and publication period will last for several years after the conclusion of subject enrollment.

Together with QuesGen Systems, the Clinical Core will ensure that data standards are established for the data model e.g. conformity of field formats, field codes and names to ensure consistency across all datasets. After the initial approval of the data model and eCRFs, any proposed changes to the database will be reviewed by QuesGen and the Clinical Core for impact upon the existing data in the repository. Approved changes will be fully documented with dataset updates to maintain data quality and accuracy. After data adjudication and curation with the Informatics Core, Thomson Reuters, and other external partners, QuesGen Systems will be responsible for importing cleaned datasets to FITBIR on a quarterly basis as well as tranSMART and other analytic platforms as determined by the Informatics and Biostatistical Cores.

12.2 BIOSPECIMENS

Biospecimens collection. Study sites will collect, process, and ship blood biospecimens according to the NINDS TBI-CDE Biospecimens Protocol, to a central biorepository at University of Pittsburgh. Each site will batch and ship biospecimens to the central repository on a quarterly basis. The UP biorepository will aliquot and ship serum and plasma specimens to Abbott Laboratories to conduct research assays on potential diagnostic biomarkers for TBI. Formalized QC/QA policies for collection, processing and storage were developed and

validated for TRACK-TBI Pilot. Refer to the full Biospecimens Protocol (Appendix 4) for detailed information regarding control of collection supplies (disposables and reagents), identification (using Study ID) and labeling conventions, collection and processing methods, storage and retrieval, shipping and receiving, training, and security. Together these pre-analytic QC/QA policies minimize circumstances that could adversely affect scientific results, ensure the safety of research personnel, and aid in the efficient operation of the TRACK-TBI Biospecimen Repository. The Biospecimens Core will review the efficiency of existing processes and procedures on a quarterly basis.

Whole brain collection. When possible, sites will approach next of kin for donation of subject and control brains. We expect to collect <50 brains from individuals who die in both the acute and sub-acute time periods. The brains will be processed locally and shipped to the TBI Brain Bank at the Center for Neuroscience and Regenerative Medicine (CNRM) in Bethesda, MD (Dr. Dan Perl, Director). This will facilitate neuropathologic studies of subjects who have been characterized with MRI, proteomic, and genetic studies, providing further opportunity to validate imaging and biomarker results. See Appendix 11.

Biospecimen Repository. The TRACK-TBI Biospecimen Repository at University of Pittsburgh will ship biospecimens to relevant analytic partners, where genomic and proteomic analyses will be used to discover new TBI biomarkers. Raw and derived molecular data will be curated and uploaded to LONI for aggregation and harmonization before being transferred to FITBIR and to tranSMART for analysis.

12.3 NEUROIMAGING

Standardization of MR across sites. Imaging protocols will be standardized in collaboration with InTBIR. All study MR systems will initially be characterized with the Magphan® Quantitative Imaging Phantom (Phantom Laboratory, Salem, NY) designed to measure signal-to-noise ratio, object size scaling, and spatial distortion. The Magphan® Phantom has been extensively used for high-resolution structural brain imaging in numerous trials, including ADNI, as follows:

1. Serial imaging of the phantom will track scanner performance over the study enrollment period using an online MR Distortion and Image Quality service (ImageOwl, Salem, NY) which identifies scanner errors or defects and corrects for scaling errors and gradient non-linearity.
2. Standardization for diffusion MR imaging using an ice water phantom adopted by ACRIN for multi-site diffusion imaging trials.
3. Performance of fMRI will be assessed using the Biomedical Informatics Research Network (BIRN) phantom, with serial data analyzed to assess signal mean and standard deviations, temporal fluctuations, and drift.

Standardized MR protocols will maximize consistency among study sites and across vendor platforms. Within vendors the protocols will be identical and shared via manufacturer-generated tools (e.g. edx files, examcards). Across vendors, protocols will match spatial coverage, voxel dimensions, and primary contrast parameters (e.g. echo time, repetition time). A TRACK-TBI board-certified neuroradiologist will evaluate the test-retest scans from all sites to assure diagnostic image quality and for pathoanatomic analysis of structural MRI sequences.

Neuroimaging Repository. Neuroimages will be de-identified at each study site before uploading to the LONI repository as DICOM files, utilizing LONI's de-identification and transport protocol. Image data will then be passed into one of a series of modality-specific semi-automated quality assessment pipelines and evaluated by LONI for protocol conformance and quality. QA results will be provided to acquisition sites within 48 hours and the scan repeated if it does not meet QA criteria. Images passing QA will be sent into a modality-specific image analysis pipeline and the resulting processed images and measures will be returned to the neuroimaging repository. The existing image processing provenance collection method will ensure that derived images and data are properly annotated and preserved for future research. The curated raw images and quantifications will be uploaded to FITBIR and meta-data will be uploaded to tranSMART. Refer to the full Neuroimaging Protocol (Appendix 5) for detailed specifications.

Integration and analysis. The Neuroimaging Core will direct the coding of all TRACK-TBI neuroimages to NIH-CDE data standards. Final versions of the curated TBI-CDE compliant clinical and outcomes data, neuroimaging, and molecular measures will be integrated within the LONI repository, then validated and transformed using NINDS/FITBIR standards. Data will be exported to tranSMART to support a variety of statistical, bioinformatic and neuroinformatic analyses, along with a second level of QA possible through a deeper inspection of the integrated heterogeneous data modalities. Analytics systems in tranSMART and the LONI repository will support real-time inspection of data, hypothesis testing, data subsetting, and data exploration across studies.

13. ADVERSE EVENTS

Events may be categorized as Adverse Events (AEs) if the distress felt by the subject requires termination of testing or procedure (e.g. outcomes testing, MRI). Anticipated AEs in TRACK-TBI include:

- Excessive discomfort, pain, or bruising during venipuncture
- Claustrophobia or severe anxiety in the MRI
- Anxiety during outcomes administration due to sensitive material discussed
- Anxiety due to fear of legal discovery associated with high-risk/illegal behaviors during interview

Reporting Procedures. AEs will be documented in the AE section of the QuesGen database. Each Site PI will be informed on a weekly basis regarding the number and nature of the AEs at their site. The Clinical Core will review the number and nature of AEs at each site on a monthly basis. Given that each site must reach the 80% completion of follow-up milestone, if AEs exceed 10% of site enrollment the Clinical Core will contact the site to discuss potential methods for reduction of AE incidence.

Other Serious Events. Any other serious events that do not meet the above criteria will be reported to the Clinical Core within five working days. These AEs will be recorded for individual subjects during the 12-month study period. In addition to submission as required per site IRB regulations, AE data will be analyzed quarterly and reported in the quarterly reports submitted to the Executive Committee.

14. DATA SHARING

Data sharing and mutual collaboration among research teams to accelerate research in TBI is a fundamental tenet of the TRACK-TBI project and are core beliefs of its investigators. The TRACK-TBI database and repositories can only serve their intended purposes as a current and legacy resource for further research with a robust, transparent, and open-access data sharing plan. To ensure optimal use of the data and to prevent possible misuse, the TRACK-TBI Steering Committee has established policies for data sharing as well as policies for publication and publication credits for those who use TRACK-TBI data. Our policies harmonize with FITBIR requirements, as developed by the FITBIR Policy Committee (https://fitbir.nih.gov/jsp/about/policy.jsp). Specifically, 6 months after the end of the performance period of the grant, de-identified data will be made available to other researchers who have submitted data to FITBIR. Twelve months after the end of the grant or contract, data will be open to all qualified and approved researchers. We have also adopted policies to allow us to collaborate and share data throughout the study to advance knowledge in TBI.

To foster collaboration and accelerate research, we propose staged access to TRACK-TBI data, which will optimize reliability, promote best use of the data, encourage academic productivity and promote team science. Prior to closure of the performance period of the grant, TRACK-TBI participating sites, investigators, and our Public-Private Partners will have access to the data. We will also provide access to external investigators and potential Public-Private Partners who request such access of the TRACK-TBI Steering Committee and agree to the conditions of the TRACK-TBI Data Use Agreement.

14.1 INTERNAL DATA SHARING

In order to facilitate best use of the data and to streamline analyses and reporting during the study phase, we will implement the following:

1. The initial analysis for a research question that has been specified in the Specific Aims of the grant will be coordinated by the relevant Core Leader in collaboration with the Co-Investigators and the Biostatical and CER Core. All interested TRACK-TBI Investigators and Partners will be included.
2. Research questions not specified in the Specific Aims of the grant may be addressed by TRACK-TBI Investigators and Partners following internal submission of the TRACK-TBI Data Use Agreement, and approval granted by the TRACK-TBI Steering Committee. Content of requests to pursue new research questions will be made available to all TRACK-TBI investigators to promote transparency, prevent duplication of efforts, and promote collaboration with other interested TRACK-TBI investigators.

14.2 EXTERNAL DATA SHARING

Prior to the FITBIR-mandated date for data sharing, we will grant early access to external investigators and new Public-Private Partners who request such access of the TRACK-TBI Steering Committee. The request and Data Use Agreement will be confidentially reviewed by the Steering Committee for feasibility. Once approved, the applicant will be provided access to the requested data via the One Mind for Research (OMFR)-tranSMART platform. As with internal requests, content of requests to pursue new research questions will be made available to all TRACK-TBI investigators to promote transparency, prevent duplication of efforts, and promote collaboration. We will encourage such collaboration with external investigators to provide domain expertise and foster the development of new multidisciplinary teams. Any publications that emerge from use of the data are subject to the limited review and authorship acknowledgments set forth in a Data Use Agreement issued by the Coordinating Center at the University of California, San Francisco.

15. CLINICAL PROTOCOL MAINTENANCE

15.1 PROTOCOL MODIFICATIONS

Please refer to Appendix 7 for the procedure for revisions to the Protocol.

15.2 PROTOCOL DEVIATIONS

Protocol compliance and study performance will be monitoring by the Clinical Core using the study reports and dashboards provided by QuesGen Systems. Any protocol deviations should be reported and described in full under the research subject's "Subject → Protocol Deviations" tab within the QuesGen Database.

Protocol deviations may include:

Clinical

- Subject enrolled with unclear time of injury (for assessment of enrollment <24 hours)
- Baseline interview information missed on enrollment

Biospecimens

- Blood collected for the baseline sample outside of 24 hour window
- Blood processing times deviated from protocol
- Blood collection was missed at any timepoint
- Blood collected outside of the 2-week or 6-month window without prior approval for exception by the Executive Committee approval.

Neuroimaging

- MRI collected outside of the 2-week window
- MRI collected outside of the 6-month window without prior approval for exception by the Executive Committee.
- Certain MRI sequences were not completed or required separate visits to complete
- MRI missed at 6 months

Outcomes

- CA+MRI/CA+MRI-HDFT Cohort: MRI and Outcomes not completed within 3 days of each other
- Certain outcome measures were incomplete

In most major instances and especially concerning enrollment, MRI or outcomes administration dates, protocol deviations must be reported to the Executive Committee for approval before data collection can resume for the subject at the respective timepoint of deviation. Due to the time sensitivity of blood draws and processing, deviations can proceed at the local level but must be reported to the Clinical Core within 2 business days. Under circumstances in which the permissible window for outcome assessment cannot be met, with agreement from the subject, data collectors can request permission from the Executive Committee to complete the scheduled follow-up out-of-window. A protocol deviation will not need to be reported if permission is obtained in advance. Requests made to the Executive Committee to perform 6-month outcomes along with the MRI and blood draws outside of the window will also be considered exceptions and a protocol deviation will not need to be reported if permission is obtained in advance.

In the event that a consistent pattern of poor performance (e.g. not enrolling allotted amount of patients per quarter, not achieving at or above 80% follow-up rate across all timepoints) or inadequate compliance (e.g. insufficient blood draw amount, CT or MR imaging, CRF completion without errors, full outcomes battery completion, or any timepoint completed outside the approved window) is detected, the responsible site investigator will be notified and required to present a plan for improvement and a time line for accomplishing this to the Clinical Core. Failure to meet objectives specified in this plan may result in termination of the project or assignment of the project to another investigator.

16. TRACK-TBI LONGITUDINAL STUDY

The goal of the TRACK-TBI Longitudinal (TRACK-TBI LONG) study is to improve understanding of the long-range natural history of TBI by extending follow-up of the TRACK-TBI cohort beyond the first 12 months after injury. TRACK-TBI LONG is funded by the National Football League Scientific Advisory Board Funding Opportunity.

There are four Specific Aims:

- Specific Aim 1. Characterize the long-term effects of TBI in the TRACK-TBI cohort.
- Specific Aim 2. Characterize the relationship of imaging biomarkers to the long-term trajectory of neurocognitive/psychological function in TBI.
- Specific Aim 3. Characterize the relationship of proteomic biomarkers to the long-term trajectory of neurocognitive/psychological function in TBI.
- Specific Aim 4. Assess comprehensive clinical-pathological correlations of brain-injured TRACK-TBI participants (n=30) to determine TBI-associated structural and biological changes correlated with clinical and neuroradiological phenotyping.

For more information on the TRACK-TBI LONG study, please see Appendix 12 “TRACK-TBI LONG SOP” in the “Clinical Protocol Appendices” folder on Dropbox.

17. REFERENCES

1. Finkelstein E, Corso P, Miller T and Associates. The Incidence and Economic Burden of Injuries in the United States. New York (NY): Oxford University Press; 2006.
2. Maas AI, Roozenbeek B, Manley GT. Clinical trials in traumatic brain injury: past experience and current developments. Neurotherapeutics 2010 Jan;7(1):115-26. Review. NIHMS169841.
3. Teasdale G, Jennett B. Assessment and prognosis of coma after head injury. Acta Neurochir. 1976;34(1-4):45-55. PMID: 961490.
4. Wilson JT, Pettigrew LE, Teasdale GM. Structured interviews for the Glasgow Outcome Scale and the extended Glasgow Outcome Scale: guidelines for their use. J Neurotrauma. 1998 Aug;15(8):573-85. PMID: 9726257.
5. Maas AI, Harrison-Felix CL, Menon D, Adelson PD, Balkin T, Bullock R, Engel DC, Gordon W, Orman JL, Lew HL, Robertson C, Temkin N, Valadka A, Verfaellie M, Wainwright M, Wright DW, Schwab K. Common data elements for traumatic brain injury: recommendations from the interagency working group on demographics and clinical assessment. Arch Phys Med Rehabil. 2010 Nov;91(11):1641-9. PMID: 21044707.
6. Manley GT, Diaz-Arrastia R, Brophy M, Engel D, Goodman C, Gwinn K, Veenstra TD, Ling G, Ottens AK, Tortella F, Hayes RL. Common data elements for traumatic brain injury: recommendation from the biospecimens and biomarkers working group. Arch Phys Med Rehabil 2010 Nov;91:1667-72. PMID: 21044710.
7. Duhaime AC, Gean AD, Haacke EM, Hicks R, Wintermark M, Mukherjee P, Brody D, Latour L, Riedy G; Common Data Elements Neuroimaging Working Group Members, Pediatric Working Group Members. Common data elements in radiologic imaging of traumatic brain injury. Arch Phys Med Rehabil. 2010 Nov;91(11):1661-6. PMID: 21044709.
8. Whyte J, Vasterling J, Manley GT. Common data elements for research on traumatic brain injury and psychological health: current status and future development. Arch Phys Med Rehabil. 2010 Nov;91(11):1692-6. Review. PMID: 21044713.
9. Yuh EL, Mukherjee P, Lingsma HF, Yue JK, Ferguson AR, Gordon WA, Valadka AB, Schnyer DM, Okonkwo DO, Maas AI, Manley GT; TRACK-TBI Investigators. Magnetic resonance imaging improves 3- month outcome prediction in mild traumatic brain injury. Ann Neurol. 2012 Oct 11. PMID: 23224915.
10. Okonkwo DO, Yue JK, Puccio AM, Panczykowski D, Inoue T, McMahon PJ, Sorani MD, Yuh EL, Lingsma H, Maas A, Valadka A, Manley GT; TRACK-TBI Investigators. GFAP-BDP as an acute diagnostic marker in traumatic brain injury: results from the prospective TRACK-TBI study. J Neurotrauma. 2013 Mar 14. PMID: 23489259.
11. Ratcliff JJ, Adeoye O, Lindsell CJ, Hart KW, Pancioli AM, McMullan JT, Yue JK, Nishijima D, Gordon WA, Valadka AB, Okonkwo DO, Maas AIR, Manley G, and the TRACK-TBI Investigators. Emergency Department Disposition of the Mild Traumatic Brain Injured Patient: A Multicenter Prospective Cohort Study. Presented at the Society for Academic Emergency Medicine Annual Meeting, Atlanta, May 14-18 2013.
12. ACRM; Mild Traumatic Brain Injury Committee. (1993). Definition of mild traumatic brain injury. Journal of Head Trauma Rehabilitation, 8(3), 86-87.
13. Jagoda AS, Bazarian JJ, Bruns JJ Jr, Cantrill SV, Gean AD, Howard PK, Ghajar J, Riggio S, Wright DW, Wears RL, Bakshy A, Burgess P, Wald MM, Whitson RR; American College of Emergency Physicians; Centers for Disease Control and Prevention. Clinical Policy: neuroimaging and decisionmaking in adult mild traumatic brain injury in the acute setting. Ann Emergen Med. 2008 Dec;52(6):714-48. PMID: 19027497.
14. Jack CR Jr, Bernstein MA, Fox NC, et al. The Alzheimer's Disease Neuroimaging Initiative (ADNI): MRI methods. J Magn Reson Imaging. 2008 Apr;27(4):685-91. PMC2544629.
15. Gunter JL, Bernstein MA, Borowski BJ, Ward CP, Britson PJ, Felmlee JP, Schuff N, Weiner M, Jack CR. Measurement of MRI scanner performance with the ADNI phantom. Med Phys. 2009 June; 36(6): 2193–2205. PMC2754942.
16. Chenevert TL, Galbán CJ, Ivancevic MK, Rohrer SE, Londy FJ, Kwee TC, Meyer CR, Johnson TD, Rehemtulla A, Ross BD. Diffusion coefficient measurement using a temperature-controlled fluid for quality control in

multicenter studies. J Magn Reson Imaging. 2011 Oct;34(4):983-7. PMC3177164.
17. Friedman L, Glover GH. Report on a multicenter fMRI quality assurance protocol. J Magn Reson Imaging. 2006 Jun;23(6):827-39. PMID: 16649196.
18. Cohen J (1988) Statistical power analysis for the behavioral sciences; second edition. Hillsdale, New Jersey: Lawrence Erlbaum Associates, Publishers.
19. Yuh EL, Gean AD, Manley GT, Callen AL, Wintermark M. Computer-aided assessment of head computed tomography (CT) studies in patients with suspected traumatic brain injury. J Neurotrauma 2008; 25(10): 1163-1172. PMID: 18986221.
20. Yuh EL, Cooper SR, Ferguson AR, Manley GT. Quantitative CT improves outcome prediction in acute traumatic brain injury. J Neurotrauma 2012b; 29(5):735-746. PMC3303103.
21. Schuff N, Woerner N, Boreta L, Kornfield T, Shaw LM, Trojanowski JQ, Thompson PM, Jack CR Jr, Weiner MW; Alzheimer's Disease Neuroimaging Initiative. MRI of hippocampal volume loss in early Alzheimer's disease in relation to ApoE genotype and biomarkers. Brain 2009; 132:1067-1077. PMC2668943.
22. Venugopal S, Sampat M, LaHue S, et al. Mild traumatic brain injury causes reduction in hippocampal volumes over time that correlates with patient-reported cognitive impairment but does not correlate with verbal memory function. Brain Inj 2012; 26(4-5):782-783.
23. Woon FL, Hedges DW. Amygdala volume in adults with posttraumatic stress disorder: a meta-analysis. J Neuropsychiatry Clin Neurosci 2009; 21:5-12. PMID: 19359446.
24. Karl A, Schaefer M, Malta LS, Dorfel D, Rohleder N, Werner A. A meta-analysis of structural brain abnormalities in PTSD. Neurosci Biobehav Rev 2006; 30:1004-1031. PMID: 16730374.
25. Woon FL, Sood S, Hedges DW. Hippocampal volume deficits associated with exposure to psychological trauma and posttraumatic stress disorder in adults: a meta-analysis. Prog Neuropsychopharmacol Biol Psychiatry 2010; 34:1181-1188. PMID: 20600466.
26. Morey RA, Gold AL, LaBar KS, Beall SK, Brown VM, Haswell CC, Nasser JD, Wagner HR, McCarthy G; Mid-Atlantic MIRECC Workgroup. Amygdala volume changes in posttraumatic stress disorder in a large case-controlled veterans group. Arch Gen Psychiatry 2012; 69:1169-1178. PMID: 23117638.
27. Niogi SN, Mukherjee P. Diffusion tensor imaging of mild traumatic brain injury. J Head Trauma Rehabil. 2010; 25:241-255. PMID: 20611043.
28. Niogi SN, Mukherjee P, Ghajar J, Johnson C, Kolster RA, Sarkar R, Lee H, Meeker M, Zimmerman RD, Manley GT, McCandliss BD. Extent of microstructural white matter injury in postconcussive syndrome correlates with impaired cognitive reaction time: a 3T diffusion tensor imaging study of mild traumatic brain injury. AJNR Am J Neuroradiol 2008; 29:967-973. PMID: 18272556.
29. Niogi SN, Mukherjee P, Ghajar J, Johnson CE, Kolster R, Lee H, Suh M, Zimmerman RD, Manley GT, McCandliss BD. Structural dissociation of attentional control and memory in adults with and without mild traumatic brain injury. Brain 2008; 131:3209-3221. PMID: 18952679.
30. Halterman CI, Langan J, Drew A, Rodriguez E, Osternig LR, Chou LS, van Donkelaar P. Tracking the recovery of visuospatial attention deficits in mild traumatic brain injury. Brain 2006; 129:747-753. PMID: 16330498.
31. Lundin A, de Boussard C, Edman G, Borg J. Symptoms and disability until 3 months after mild TBI. Brain Inj 2006;20:799-806. PMID: 17060147.
32. Li YO, LaHue S, Chen R, Cooper SC, Yue J, Manley GT, Mukherjee P. Resting state fMRI reveals altered functional connectivity in cortical and subcortical networks one month after mild traumatic brain injury. Proc Int Soc Magn Reson Med 2012; 1599.
33. Hillary FG, Slocomb J, Hills EC, Fitzpatrick NM, Medaglia JD, Wang J, Good DC, Wylie GR. Changes in resting connectivity during recovery from severe traumatic brain injury. Int J Psychophysiol 2011; 82:115-123. PMID: 21473890.
34. Johnson B, Zhang K, Gay M, Horovitz S, Hallett M, Sebastianelli W, Slobounov S. Alteration of brain default network in subacute phase of injury in concussed individuals: resting-state fMRI study. Neuroimage 2012; 59: 511-518. PMC3196274.
35. Kasahara M, Menon DK, Salmond CH, Outtrim JG, Taylor Tavares JV, Carpenter TA, Pickard JD, Sahakian BJ, Stamatakis EA. Altered functional connectivity in the motor network after traumatic brain injury.

Neurology 2010; 75:168-176. PMC2905928.
36. Marquez de la Plata CD, Garces J, Shokri Kojori E, Grinnan J, Krishnan K, Pidikiti R, Spence J, Devous MD Sr, Moore C, McColl R, Madden C, Diaz-Arrastia R. Deficits in functional connectivity of hippocampal and frontal lobe circuits after traumatic axonal injury. Arch Neurol 2011; 68:74-84. PMC3100186.
37. Mayer AR, Mannell MV, Ling J, Gasparovic C, Yeo RA. Functional connectivity in mild traumatic brain injury. Hum Brain Mapp 2011; 32:1825-35. PMC3204375.
38. Nakamura T, Hillary FG, Biswal BB. Resting network plasticity following brain injury. PLoS ONE 2009;4:e8220. PMC2788622.
39. Sharp DJ, Beckmann CF, Greenwood R, Kinnunen KM, Bonnelle V, De Boissezon X, Powell JH, Counsell SJ, Patel MC, Leech R. Default mode network functional and structural connectivity after traumatic brain injury. Brain 2011; 134:2233-2247. PMID: 21841202.
40. Tang L, Ge Y, Sodickson DK, Miles L, Zhou Y, Reaume J, Grossman RI. Thalamic resting-state functional networks: disruption in patients with mild traumatic brain injury. Radiology 2011; 260:831-840. PMC3157002.
41. Vos PE, Jacobs B, Andriessen TM, Lamers KJ, Borm GF, Beems T, Edwards M, Rosmalen CF, Vissers JL. GFAP and S100B are biomarkers of traumatic brain injury: an observational cohort study. Neurology 2010;75:1786-1793. PMID: 21079180.
42. Vos PE, Lamers KJ, Hendriks JC, van Haaren M, Beems T, Zimmerman C, van Geel W, de Reus H, Biert J, Verbeek MM. Glial and neuronal proteins in serum predict outcome after severe traumatic brain injury. Neurology 2004;62:1303-1310. PMID: 15111666.
43. Mondello S, Papa L, Buki A, Bullock MR, Czeiter E, Tortella FC, Wang KK, Hayes RL. Neuronal and glial markers are differently associated with computed tomography findings and outcome in patients with severe traumatic brain injury: a case control study. Crit Care 2011;15:R156. PMC3219030.
44. Metting ZZ, Wilcza NN, Rodiger LAL, Schaaf JMJ, van der Naalt JJ. GFAP and S100B in the acute phase of mild traumatic brain injury. Neurology 2012; 78, 1428-1433. PMID: 22517109.
45. Papa L, Lewis L, Falk J, Silvestri S, Giordano P, Brophy GM, Demery J, Dixit NK, Ferguson I, Liu MC, Mo J, Akinyi L, Schmid K, Robertson CS, Mondello S, Tortella FC, Hayes RL, Wang KKW. Serum levels of ubiquitin C-terminal hydrolase distinguish mild traumatic brain injury from trauma controls and are elevated in mild and moderate traumatic brain injury patients with intracranial lesions and neurosurgical intervention. J Trauma Acute Care Surg 2012 May;72(5):1335-1344. PMID: 22673263.
46. Papa L, Lewis L, Falk J, Zhang Z, Silvestri S, Giordano P, Brophy GM, Demery J, Dixit NK, Ferguson I, Liu MC, Mo J, Akinyi L, Schmid K, Robertson CS, Mondello S, Tortella FC, Hayes RL, Wang KK. Elevated levels of serum glial fibrillary acidic protein breakdown products in mild and moderate traumatic brain injury are associated with intracranial lesions and neurosurgical intervention. Ann Emerg Med. 2012 Jun;59(6):471-83. PMID: 22071014.
47. Stein DM, Lindel AL, Murdock KR, Kufera JA, Menaker J, Scalea TM. Use of serum biomarkers to predict secondary insults following severe traumatic brain injury. *Shock* 2012;37:563-568. PMID: 22552017.
48. Hergenroeder GW, Moore AN, McCoy JP Jr, Samsel L, Ward NH 3rd, Clifton GL, Dash PK. Serum IL-6: a candidate biomarker for intracranial pressure elevation following isolated traumatic brain injury. *J Neuroinflammation* 2010;7:19. PMC2853529.
49. Rhind SG, Crnko NT, Baker AJ, Morrison LJ, Shek PN, Scarpelini S, Rizoli SB. Prehospital resuscitation with hypertonic saline-dextran modulates inflammatory, coagulation and endothelial activation marker profiles in severe traumatic brain injured patients. *J Neuroinflammation* 2010 Jan 18;7:5. PMC2819256.
50. Dambinova SA, Khounteev GA, Izykenova GA, Zavolokov IG, Ilyukhina AY, Skoromets AA. Blood test detecting autoantibodies to N-methyl-D-aspartate neuroreceptors for evaluation of patients with transient ischemic attack and stroke. *Clin Chem* 2003;49:1752-1762. PMID: 14500616.
51. Colasanti T, Barbati C, Rosano G, Malorni W, Ortona E. Autoantibodies in patients with Alzheimer's disease: pathogenetic role and potential use as biomarkers of disease progression. *Autoimmun Rev* 2010;9:807-811. PMID: 20656067.
52. D'Andrea MR. Add Alzheimer's disease to the list of autoimmune diseases. *Med Hypotheses* 2005;64:458-463. PMID: 15617848.
53. Ankeny DP, Lucin KM, Sanders VM, McGaughy VM, Popovich PG. Spinal cord injury triggers systemic autoimmunity: evidence for chronic B lymphocyte activation and lupus-like autoantibody synthesis. *J*

Neurochem 2006;99:1073-1087. PMID: 17081140.

54. Popovich PG, Stokes BT, Whitacre CC. Concept of autoimmunity following spinal cord injury: possible roles for T lymphocytes in the traumatized central nervous system. *J Neurosci Res* 1996;45:349-363. PMID: 8872895.
55. Lancaster E, Dalmau J. Neuronal autoantigens--pathogenesis, associated disorders and antibody testing. *Nat Rev Neurol* 2012;8:380-390. PMID: 22710628.
56. Ponomarenko NA, Durova OM, Vorobiev II, Belogurov AA, Telegin GB, Suchkov SV, Misikov VK, Morse HC 3rd, Gabibov AG. Catalytic activity of autoantibodies toward myelin basic protein correlates with the scores on the multiple sclerosis expanded disability status scale. *Immunol Lett* 2006;103:45-50. PMID: 16297986.
57. Hedegaard CJ, Chen N, Sellebjerg F, Sorensen PS, Leslie RG, Bendtzen K, Nielsen CH. Autoantibodies to myelin basic protein (MBP) in healthy individuals and in patients with multiple sclerosis: a role in regulating cytokine responses to MBP. *Immunology* 2009;128:e451-e461. PMC2753924.
58. Cox AL, Coles AJ, Nortje J, Bradley PG, Chatfield DA, Thompson SJ, Menon DK. An investigation of auto-reactivity after head injury. *J Neuroimmunol* 2006;174:180-186. PMID: 16519951.
59. Goryunova AV, Bazarnaya NA, Sorokina EG, Semenova NY, Globa OV, Semenova ZhB, Pinelis VG, Roshal' LM, Maslova Ol. Glutamate receptor autoantibody concentrations in children with chronic post-traumatic headache. *Neurosci Behav Physiol* 2007;37:761-764. PMID: 17922239.
60. Tanriverdi F, De Bellis A, Bizzarro A, Sinisi AA, Bellastella G, Pane E, Bellastella A, Unluhizarci K, Selcuklu A, Casanueva FF, Kelestimur F. Antipituitary antibodies after traumatic brain injury: is head trauma-induced pituitary dysfunction associated with autoimmunity? *Eur J Endocrinol* 2008;159:7-13. PMID: 18463108.
61. Tanriverdi F, De Bellis A, Battaglia M, Bellastella G, Bizzarro A, Sinisi AA, Bellastella A, Unluhizarci K, Selcuklu A, Casanueva FF, Kelestimur F. Investigation of antihypothalamus and antipituitary antibodies in amateur boxers: is chronic repetitive head trauma-induced pituitary dysfunction associated with autoimmunity? *Eur J Endocrinol* 2010;162:861-867. PMID: 20176736.
62. Diaz-Arrastia R, Baxter VK. Genetic factors in outcome after traumatic brain injury: what the human genome project can teach us about brain trauma. *J Head Trauma Rehabil* 2006;21:361-374. PMID: 16915011.
63. Haacke EM, Duhaime AC, Gean AD, Riedy G, Wintermark M, Mukherjee P, Brody DL, DeGraba T, Duncan TD, Elovic E, Hurley R, Latour L, Smirniotopoulos JG, Smith DH. Common data elements in radiologic imaging of traumatic brain injury. J Magn Reson Imaging 2010; 32:516-43. PMID: 20815050.
64. Desikan RS, Ségonne F, Fischl B, Quinn BT, Dickerson BC, Blacker D, Buckner RL, Dale AM, Maguire RP, Hyman BT, Albert MS, Killiany RJ. An automated labeling system for subdividing the human cerebral cortex on MRI scans into gyral based regions of interest. Neuroimage 2006; 31:968-980. PMID: 16530430.
65. Fischl B. FreeSurfer. Neuroimage 2012; 62(2):774-781. PMID: 22248573.
66. Smith SM, Jenkinson M, Johansen-Berg H, Rueckert D, Nichols TE, Mackay CE, Watkins KE, Ciccarelli O, Cader MZ, Matthews PM, Behrens TE. Tract-based spatial statistics: voxelwise analysis of multi- subject diffusion data. Neuroimage 2006 Jul 15;31(4):1487-1505. PMID: 16624579.
67. Mori S, Oishi K, Jiang H, Jiang L, Li X, Akhter K, Hua K, Faria AV, Mahmood A, Woods R, Toga AW, Pike GB, Neto PR, Evans A, Zhang J, Huang H, Miller MI, van Zijl P, Mazziotta J. Stereotaxic white matter atlas based on diffusion tensor imaging in an ICBM template. Neuroimage 2008; 40:570-582. PMC2478641.
68. Teasdale GM, Nicoll JAR, Murray G, Fiddes M. Association of apolipoprotein E polymorphism with outcome after head injury. *Lancet* 1997;350:1069-1071. PMID: 10213549.
69. Jordan BD, Relkin NR, Ravdin LD, Jacobs AR, Bennett A, Gandy S. Apolipoprotein E e4 associated with chronic traumatic brain injury in boxing. *J Am Med Assc* 1997;278:136-140. PMID: 9214529.
70. Crawford FC, Vanderploeg RD, Freeman MJ, Singh S, Waisman M, Michaels L, Abdullah L, Warden D, Lipsky R, Salazar A, Mullan MJ. APOE genotype influences acquisition and recall following traumatic brain injury. *Neurology* 2002;58:1115-1118. PMID: 11940706.
71. Sundstrom A, Marklund P, Nilsson LG, Cruts M, Adolfsson R, Van Broeckhoven C, Nyberg L. APOE influences on neuropsychological function after mild head injury. *Neurology* 2004;62:1963-1966. PMID: 15184597.

72. Teasdale GM, Murray GD, Nicoll JA. The association between APOE e4, age, and outcome after head injury: a prospective cohort study. *Brain* 2005;128:2556-2561. PMID: 16033781.
73. Liaquat I, Dunn LT, Nicoll JA, Teasdale GM, Norrie JD. Effect of apolipoprotein E genotype on hematoma volume after trauma. *J Neurosurg* 2002;96:90-96. PMID: 11795256.
74. Ariza M, Pueyo R, Matarin Mdel M, Junque C, Mataro M, Clemente I, Moral P, Poca MA, Garnacho A, Sahuquillo J. Influence of APOE polymorphism on cognitive and behavioural outcome in moderate and severe traumatic brain injury. *J Neurol Neurosurg Psychiatry* 2006;77:1191-1193. PMC2077553.
75. Wagner AK, Miller MA, Scanlon J, Ren D, Kochanek PM, Conley YP. Adenosine A1 receptor gene variants associated with post-traumatic seizures after severe TBI. *Epilepsy Res* 2010;90:259-272. PMC2933564.
76. Ariza M, Matarin MD, Junque C, Mataro M, Clemente I, Moral P, Antonia Poca M, Carnacho A, Sahuquillo J. Influence of Angiotensin-converting enzyme polymorphism on neuropsychological subacute performance in moderate and severe traumatic brain injury. *J Neuropsychiatry Clin Neurosci* 2006;18:39-44. PMID: 16525069.
77. McAllister TW, Rhodes CH, Flashman LA, McDonald BC, Belloni D, Saykin AJ. Effect of the dopamine D2 receptor T allele on response latency after mild traumatic brain injury. *Am J Psychiatry* 2005;162:1749-1751. PMID: 16135640.
78. McAllister TW, Flashman LA, Harker Rhodes C, Tyler AL, Moore JH, Saykin AJ, McDonald BC, Tosteson TD, Tsongalis GJ. Single nucleotide polymorphisms in ANKK1 and the dopamine D2 receptor gene affect cognitive outcome shortly after traumatic brain injury: a replication and extension study. *Brain Inj* 2008;22:705-714. PMC3169811.
79. Hoh NZ, Wagner AK, Alexander SA, Clark RB, Beers SR, Okonkwo DO, Ren D, Conley YP. BCL2 genotypes: functional and neurobehavioral outcomes after severe traumatic brain injury. *J Neurotrauma* 2010;27:1413-1427. PMC2967822.
80. McAllister TW, Tyler AL, Flashman LA, Rhodes CH, McDonald BC, Saykin AJ, Tosteson TD, Tsongalis GJ, Moore JH. Polymorphisms in the brain-derived neurotrophic factor gene influence memory and processing speed one month after brain injury. *J Neurotrauma* 2012;29:1111-1118. PMC3325555.
81. Rostami E, Krueger F, Zoubak S, Dal Monte O, Raymont V, Pardini M, Hodgkinson CA, Goldman D, Risling M, Grafman J. BDNF polymorphism predicts general intelligence after penetrating traumatic brain injury. *PLoS One* 2011;6:e27389. PMC3210804.
82. Krueger F, Pardini M, Huey ED, Raymont V, Solomon J, Lipsky RH, Hodgkinson CA, Goldman D, Grafman J. The role of the Met66 brain-derived neurotrophic factor allele in the recovery of executive functioning after combat-related traumatic brain injury. *J Neurosci* 2011;31:598-606. PMC3195417.
83. Stam AH, Luijckx GJ, Poll-The BT, Ginjaar IB, Frants RR, Haan J, Ferrari MD, Terwindt GM, van den Maagdenberg AM. Early seizures and cerebral oedema after trivial head trauma associated with the CACNA1A S218L mutation. *J Neurol Neurosurg Psychiatry* 2009;80:1125-1129. PMID: 19520699.
84. Kors EE, Terwindt GM, Vermeulen FL, Fitzsimons RB, Jardine PE, Heywood P, Love S, van den Maagdenberg AM, Hann J, Frants RR, Ferrari MD.. Delayed cerebral edema and fatal coma after minor head trauma: role of the CACNA1A calcium channel subunit gene and relationship with familial hemiplegic migraine. *Ann Neurol* 2001;49:753-760. PMID: 11409427.
85. Lipsky RH, Sparling MB, Ryan LM, Xu K, Salazar AM, Goldman D, Warden DL. Association of COMT Val158Met genotype with executive functioning following traumatic brain injury. *J Neuropsychiatry Clin Neurosci* 2005;17:465-471. PMID: 16387984.
86. Wagner AK, Ren D, Conley YP, Ma X, Kerr ME, Zafonte RD, Puccio AM, Mario DW, Dixon CE. Sex and genetic associations with cerebrospinal fluid dopamine and metabolite production after severe traumatic brain injury. *J Neurosurg* 2007;106:538-547. PMID: 17432702.
87. Robertson CS, Gopinath SP, Valadka AB, Van M, Swank PR, Goodman JC. Variants of the endothelial nitric oxide gene and cerebral blood flow after severe traumatic brain injury. *J Neurotrauma* 2011;28:727- 737. PMC3088960.
88. Tanriverdi T, Uzan M, Sanus GZ, Baykara O, Is M, Ozkara C, Buyra N. Lack of association between the IL1A gene (-889) polymorphism and outcome after head injury. *Surg Neurol* 2006;65:7-10. PMID: 16378839.
89. Uzan M, Tanriverdi T, Baykara O, Kafadar A, Sanus GZ, Tureci E, Ozkara C, Uysal O, Buyra N. Association

between interleukin-1 beta (IL-1beta) gene polymorphism and outcome after head injury: an early report. *Acta Neurochir (Wien)* 2005;147:715-720. PMID: 15891809.
90. Hadjigeorgiou GM, Paterakis K, Dardiotis E, Dardioti M, Aggelakis K, Tasiou A, Xiromerisiou G, Komnos A, Zintzaras E, Scarmeas N, Papadimitrious A, Karantanas A. IL-1RN and IL-1B gene polymorphisms and cerebral hemorrhagic events after traumatic brain injury. *Neurology* 2005;65:1077-1082. PMID: 16217062.
91. Dalla Libera AL, Regner A, de PJ, Centenaro L, Martins TT, Simon D. IL-6 polymorphism associated with fatal outcome in patients with severe traumatic brain injury. *Brain Inj* 2011;25:365-369. PMID: 21314275.
92. Pardini M, Krueger F, Hodgkinson C, Raymont V, Ferrier C, Goldman D, Strenziok M, Guida S, Grafman J. Prefrontal cortex lesions and MAO-A modulate aggression in penetrating traumatic brain injury. *Neurology* 2011;76:1038-1045. PMC3068009.
93. Johnson VE, Stewart W, Graham DI, Stewart JE, Praestgaard AH, Smith DH. A neprilysin polymorphism and amyloid-beta plaques after traumatic brain injury. *J Neurotrauma* 2009;26:1197-1202. PMC2850253.
94. Chuang PY, Conley YP, Poloyac SM, Okonkwo DO, Ren D, Sherwood PR, Hravnak M, Alexander SA. Neuroglobin genetic polymorphisms and their relationship to functional outcomes after traumatic brain injury. *J Neurotrauma* 2010;27:999-1006. PMC2943497.
95. Martinez-Lucas P, Moreno-Cuesta J, Garcia-Olmo DC, Sanchez-Sanchez F, Escribano-Martinez J, del Pozo AC, Lizan-Garcia M, Garcia-Olmo D. Relationship between the Arg72Pro polymorphism of p53 and outcome for patients with traumatic brain injury. *Intensive Care Med* 2005;31:1168-1173. PMID: 16007417.
96. Sarnaik AA, Conley YP, Okonkwo DO, Barr TL, Fink EL, Szabo C, Kochanek PM, Clark RS. Influence of PARP-1 polymorphisms in patients after traumatic brain injury. *J Neurotrauma* 2010;27:465-471. PMC2867630.
97. Bales JW, Garringer J, Conley YP, Puccio AM, Okonkwo DO, Dixon CE. Association between the PPP3CC gene, coding for the calcineurin gamma catalytic subunit, and severity of traumatic brain injury in humans. *J Neurotrauma* 2011;28:A101.
98. MRC CRASH Trial Collaborators, Perel P, Arango M, Clayton T, Edwards P, Komolafe E, Poccock S, Roberts I, Shakur H, Steyerberg E, Yutthakasemsunt S. Predicting outcome after traumatic brain injury: practical prognostic models based on large cohort of international patients. BMJ. 2008 Feb;336(7641):425-9. PMC2249681.
99. Steyerberg EW, Mushkudiani N, Perel P, Butcher I, Lu J, McHugh GS, Murray GD, Marmarou A, Roberts I, Habbema JD, Maas AI. Predicting outcome after traumatic brain injury: development and international validation of prognostic scores based on admission characteristics. PLoS Med. 2008 Aug 5;5(8):e165. PMC2494563.
100. Toward Precision Medicine: Building a Knowledge Network for Biomedical Research and a New Taxonomy of Disease. National Research Council (US) Committee on A Framework for Developing a New Taxonomy of Disease. Washington (DC): National Academies Press (US); 2011.
101. Biswal BB, Mennes M, Zuo XN, Gohel S, Kelly C, Smith SM, Beckmann CF, Adelstein JS, Buckner RL, Colcombe S, Dogonowski AM, Ernst M, Fair D, Hampson M, Hoptman MJ, Hyde JS, Kivinilemi VJ, Kotter R, Li SJ, Lin CP, Lowe MJ, Mackay C, Madden DJ, Madsen KH, Margulies DS, Mayberg HS, McMahon K, Monk CS, Mostofsky SH, Nagel BJ, Pekar JJ, Peltier SJ, Petersen SE, Riedl V, Rombouts SA, Rypma B, Schlaggar BL, Schnidt S, Seidler RD, Siegle GJ, SOrg C, Teng GJ, Veijola J, Villringer A, Walter M, Wang L, Weng XC, Whitfield-Garbrieli S, Williamson P, Sindischberger C, Zang YF, Zhang HY, Castellanos FX, Milham MP. Toward discovery science of human brain function. Proc Natl Acad Sci U S A. 2010; 107:4734-4739. PMC2842060.
102. Fox RJ, Sakaie K, Lee JC, Debbins JP, Liu Y, Arnold DL, Melhem ER, Smith CH, Philips MD, Lowe M, Fisher E. A validation study of multicenter diffusion tensor imaging: reliability of fractional anisotropy and diffusivity values. AJNR Am J Neuroradiol 2012; 33:695-700. PMID: 22173748.
103. Terris J, Leman P, O'Connor N, Wood R. Making an IMPACT on emergency department flow: improving patient processing assisted by consultant at triage. Emerg Med J. 2004 Sep; 21(5):537-41.
104. Pitts SR. Higher-complexity ED billing codes--sicker patients, more intensive practice, or improper payments? N Engl J Med. 2012;367(26):2465-7. PMID: 23268662.

105. Pitts SR, Pines JM, Handrigan MT, Kellermann AL. National trends in emergency department occupancy, 2001 to 2008: effect of inpatient admissions versus emergency department practice intensity. Ann Emerg Med. 2012;60(6):679-686. PMID: 22727201.
106. McMillian WD, Rogers RB: Management of prehospital antiplatelet and anticoagulant therapy in traumatic head injury: A review. J Trauma 2009; 66:942-950. PMID: 19276776.
107. Lingsma HF, Roozenbeek B, Li B, Lu J, Weir J, Butcher I, Marmarou A, Murray GD, Maas AI, Steyerberg EW. Large between-center differences in outcome after moderate and severe traumatic brain injury in the international mission on prognosis and clinical trial design in traumatic brain injury (IMPACT) study. Neurosurgery. 2011;68(3):601-7; discussion 607-8. PMID: 21311293.
108. Hukkelhoven CW, Steyerberg EW, Farace E, Habbema JD, Marshall LF, Maas AI. Regional differences in patient characteristics, case management, and outcomes in traumatic brain injury: experience from the tirilazad trials. J Neurosurg. 2002;97(3):549-57. PMID: 12296638.
